# PlanarFold: a coarse-grained molecular dynamics model of RNA in two-dimensional space

**DOI:** 10.1038/s41467-026-74729-y

**Published:** 2026-06-26

**Authors:** Lan Xiang, Yi Xue

**Affiliations:** 1 Beijing Frontier Research Center for Biological Structure; School of Life Sciences, Tsinghua University, Beijing, China; 2 State Key Laboratory of Complex, Severe, and Rare Diseases, Beijing, China

**Keywords:** RNA, Molecular modelling, Computational models

## Abstract

RNAs serve versatile functional roles by virtue of their structures and dynamics. RNA computational models are typically tailored to either perform structural modeling or solve a specific class of folding problems. Here, we present PlanarFold, a coarse-grained RNA model that integrates molecular dynamics simulation in two-dimensional space with dynamic programming to explore the diverse dynamic behaviours of RNAs, achieving a speedup of more than four orders of magnitude compared with all-atom molecular dynamics models. We demonstrate that, at the secondary structure level, PlanarFold quantitatively reproduces experimental results across diverse scenarios, including the native secondary structures, thermodynamics and kinetics, mechanical properties, and co-transcriptional and de novo folding pathways. The conformational dynamics revealed by PlanarFold can provide mechanistic insight into how RNAs perform or lose functions, and offer potential targets for mutagenesis and therapeutics design, as well as guide the development of RNA-based devices.

## Abstract (web summary)

Modelling the dynamics of RNA molecules can shed light on their function. Here, the authors present PlanarFold, a fast, coarse-grained model of RNA secondary structure dynamics that can reproduces experimental measurements.

## Introduction

RNA molecules have attracted growing interest as their roles expanded from genetic messengers to catalytic agents, regulatory machineries, aptamers, etc^1,2^. The functional diversity of RNAs is tightly linked to their specific secondary and tertiary structures, which are typically formed hierarchically^3,4^, as well as to their conformational dynamics. Occasionally, secondary structures may come into play solely^5,6^. The simple four-character (A, U, C, and G) primary sequences of RNAs generally encode more rugged energy landscapes compared to protein sequences^4,7,8^, with abundant local minima separated by energy barriers predominantly from secondary interactions, underpinning the delicate and intricate structural and dynamic properties of RNA molecules.

The in-depth understanding of RNA conformational dynamics can offer essential insights into the mechanisms by which regulatory RNAs execute their biological functions^9^, but the high flexibility of RNA poses significant challenges to the high-resolution structure determination through X-ray crystallography or cryogenic electron microscopy. Nuclear magnetic resonance (NMR) spectroscopy, despite its capability to probe dynamics, can only solve the structure of small RNAs (typically less than 50 nt). Moreover, the heterogeneous dynamic ensembles, formed either post-transcriptionally or co-transcriptionally, resist dissection by most bulk methods that obscure individual molecular details^10^. Recently, single-molecule technologies such as optical tweezers (OT) and atomic force microscopy have been utilized to explore the mechanical properties and folding process of RNAs under applied force^11^. Nevertheless, the mechanisms by which free RNA molecules fold into their native structures without external force manipulation remain largely unresolved. A high-efficiency computational framework that can resolve such heterogeneous dynamics and reconstruct the underlying energy landscape is therefore urgently needed.

Over the past two decades, dozens of computational models have been developed to elucidate RNA structure and/or dynamics^12^, including conventional ab initio and template-based methods^13,14^, as well as the emerging AI-based models^15^. Coarse-grained (CG) strategy^16^, which has been widely adopted in these molecular models, simplifies complex molecular structures by representing residues or helices as reduced “beads” or “sticks”. The level of coarse-graining varies across different RNA CG models, ranging from representing one nucleotide with multiple beads—such as 6 beads (3dRNA^17^ and Martini^18^), 5 beads (simRNA^19^ and oxRNA^20^), and 3 beads (TOPRNA^21^ and iFold^22^)—to a single bead (YUP^23^ and NAST^24^), and even further simplification by replacing entire helix and loop motifs with vertices and edges (RAGTOP^25^). Despite these advances, existing CG models still need improvements, such as a unified framework for integrating the structure prediction with the characterization of dynamic properties, along with high conformational sampling efficiency when folding complex and sizable RNAs.

In this work, we developed PlanarFold, a CG model that evolves over time on a two-dimensional plane, and employed it to study the structure, thermodynamics, kinetics, mechanics, and folding pathways of RNA, all conducted at the secondary structure level. This model demonstrated performance in predicting RNA secondary structure on par with state-of-the-art methods based on free energy or deep learning, as validated on both cross-sequence and cross-family tests. Aside from predicting the native structure, PlanarFold effectively captured alternative states and their corresponding free energy differences across diverse conformational transitions. With a computational speedup of more than four orders of magnitude, PlanarFold reproduced the experimental exchange rates of these conformational transitions across timescales ranging from sub-milliseconds to minutes. By incorporating steered molecular dynamics (MD) into PlanarFold, we successfully recapitulated the unfolding/refolding intermediates and mechanical features of multiple RNAs, as observed in OT experiments. Furthermore, PlanarFold is capable of investigating the co-transcriptional folding and has accurately predicted the end products and intermediates along the process. Finally, PlanarFold was employed to depict the de novo folding pathways of two large, complex RNAs.

## Results

### Model representation, force field design, and sampling strategy

**Fig. 1:**
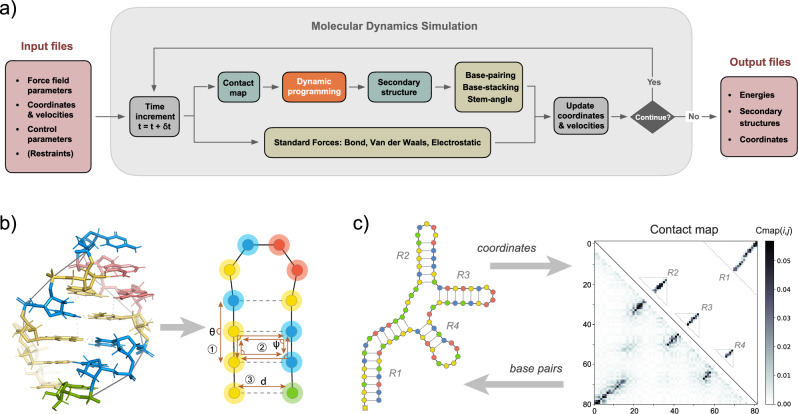
The sampling pipeline and coarse-grained representation of PlanarFold. **a** The MD pipeline of PlanarFold. By incorporating dynamic programming (DP), three potential terms (base-pairing, base-stacking, and stem-angle) are selectively applied to residues in the stem region. The instantaneous coordinates from MD is utilized by DP to determine the secondary structure at each integration step. The trajectory of secondary structures, along with their corresponding energies and base pairs, are recorded over the course of the simulation. **b** The 3D structure of an RNA hairpin (PDB ID: 1ZIH [10.2210/pdb1ZIH/pdb]) and its corresponding 2D planar representation, with nucleotides A, U, C, and G colored red, green, blue, and yellow, respectively. The arrows labeled 1, 2, and 3 indicate stem angle, base stacking, and base pairing terms, respectively. The variables θ, ψ, and d denote the angle of stem angle term, the angle of base stacking term, and the distance of base pairing term, respectively. **c** The instantaneous 2D coordinates (left) are used to calculate the contact map matrix Cmap(i,j) (right) between res[i] and res[j] (for |i - j| > 4). On the lower-left triangle of the map, all inter-residue contacts are displayed; on the upper-right triangle, only close contacts (with a cutoff of 14.0 Å) are shown. The color bar represents the values of Cmap(i,j) in Å^−2^. The contact map is partitioned into isolated helix regions (R1–R4), which are subjected to dynamic programming to determine the optimal base pairs and thus the secondary structure of the current MD frame (see Methods and Supplementary Methods).

The formation of RNA secondary structure precedes, and thus largely separates from, that of tertiary structure; in practice, RNA secondary structures can be effectively depicted in a two-dimensional plane without the need for 3D representation. On the basis of these two observations, we developed a two-dimensional coarse-grained MD model, named PlanarFold (Fig. 1a), to study RNA secondary structure and, in particular, RNA dynamics with exceptional conformational sampling efficiency. In this model, each nucleotide is represented by a single bead (Fig. 1b), evolving under a force field governed by Newton’s laws but with coordinates confined to the 2D plane. PlanarFold comprises three essential components: the CG representation of RNA molecules, the potential function, and the sampling strategy. Each component is elaborated below.

To investigate large-scale base-pair rearrangements in complex RNAs, the atomic details of nucleotides are sacrificed by representing each nucleotide as a single bead, distinguishing only nucleotide types: A, U, C, and G (Fig. 1b). By setting the model at the one-bead-per-nucleotide level, whose complexity lies between the multiple-bead model and the helix-as-stick model, PlanarFold is capable of studying the formation and disruption of secondary structures and related dynamics within a minimalistic framework. In contrast, nearly all existing models are inadequate for capturing the kinetic process of secondary structure folding. Specifically, single-bead models, such as YUP and NAST, rely on predefined secondary structures, while most multiple-bead models (e.g., SimRNA) depend on Monte Carlo sampling, making both of them unreliable for reproducing temporal progression. Although HiRE-RNA^26^, an MD-based model that features 6 or 7 beads per nucleotide, supports de novo folding of RNA, it has only been tested on relatively simple RNAs and is not publicly available.

To monitor the time-resolved dynamics of RNA, we designed a physics-based potential function for PlanarFold that is evaluated at each timestep and is differentiable within a fixed secondary structure configuration, making it fully compatible with an MD sampling scheme. PlanarFold’s potential function comprises six energy terms: bond, stem angle, base pairing, base stacking, van der Waals interaction, and electrostatic interaction.

1 $${E}_{{{\rm{potential}}}}={k}_{{{\rm{scaling}}}}\cdot ({E}_{{{\rm{bond}}}}+{E}_{{{\rm{angle}}}}+{E}_{{{\rm{stacking}}}}+{E}_{{{\rm{pairing}}}}+{E}_{{{\rm{vdW}}}}+{E}_{{{\rm{elec}}}})$$

The most distinctive feature of PlanarFold’s force field is that three terms in the potential—stem angle, base pairing, and base stacking (Fig. 1a, b)—are not applied to all residues but are selectively applied to residues identified to form stems. Specifically, these stems are not predefined by the user but are determined in real-time by a dynamic programming (DP) algorithm. We integrated a Nussinov algorithm-based DP approach into the MD framework (Fig. 1a and Supplementary Fig. 1). Importantly, instead of maximizing the number of base pairs (dependent solely on sequence) as the objective function, we maximize a score based on base-pair-dependent weights and on the proximity between residues, where proximity is derived from the instantaneous coordinates at each MD timestep (Fig. 1c, see “Methods”). The implementation of DP is proven to be crucial for PlanarFold, as it resolves the dilemma between adopting the minimalistic single-bead coarse-graining and the consequent lack of explicit definitions of base pairing and stacking. Our modified Nussinov algorithm obviates the branching scenario in DP by dividing the contact map matrix into sub-matrices. These sub-matrices represent individual helical regions and are processed independently (Fig. 1c; see “Methods”).

### Optimizing force field parameter for RNA secondary structure prediction

To faithfully capture the dynamic behaviours of RNA, the force field must reliably distinguish between low-energy conformations (native and near-native) and high-energy ones (partially folded and misfolded). Therefore, we systematically optimized the force field parameters of PlanarFold to maximize its ability to identify the native state from a large pool of candidate secondary structures, and subsequently validated the refined force field by applying it to predict the secondary structure of unseen RNAs. There are three major steps involved: first, the curation of high-quality RNA datasets from PDB structures; second, the generation of a planar conformation pool for each RNA; third, the parameter optimization aimed at selecting native or near-native structures from the decoys.

We sought to benchmark PlanarFold against several existing RNA secondary structure prediction methods, including traditional methods (RNAstructure^27^, mfold^28^, etc.) and deep-learning (DL)-based ones (SPOT-RNA^29^, MXfold2^30^, UFold^31^, etc.). For an unbiased comparison, we first gathered all the RNA sequences that have been used as training data for SPOT-RNA, MXfold2, and UFold, and merged them into a large dataset (approximately 40,000 sequences, Supplementary Table 1), termed DL-nonRedundant. From this dataset, we selected 216 RNAs with high-resolution PDB structures as the training set (TR). Then we carefully assembled a testing set of newly released, high-resolution RNA PDB structure. These RNAs were further filtered by removing those with ≥80% sequence similarity to the DL-nonRedundant dataset (Supplementary Fig. 2a), yielding a final set of 58 RNAs (TS) that can serve as an independent cross-sequence testing dataset for these DL-based predictors. It is important to note that PlanarFold is specifically designed to investigate RNA secondary structure dynamics in the plane; thus, tertiary interactions (especially long-range pseudoknots and kissing loops) are beyond the scope of this study due to geometric constraints.

Since PlanarFold employs a sample-and-select strategy, a representative and comprehensive conformation pool is required for each RNA. Although PlanarFold is capable of sampling alternative conformations by itself, its sampling efficiency is considerably lower compared to other secondary structure predictors, especially for long RNAs. Therefore, we combined restraint-free sampling with restrained sampling to generate decoys for both TR and TS, with the aid of RNAstructure (see “Methods” and Supplementary Fig. 3). For most RNAs, the conformation pools we constructed indeed encompass their native or near-native states (Supplementary Fig. 4a–c), with the best-sampled conformations reaching a median (average) F1-score of 1.0 (0.97) and 0.94 (0.88) in the TR and TS datasets, respectively.

Based on these vast conformation pools, we utilized a genetic algorithm (GA) to optimize the parameters of PlanarFold, aiming to maximize the median F1-score of RNA secondary structure prediction in the TR dataset. To minimize the risk of overfitting, our optimization procedure involves only five parameters, with each one controlling the relative contribution of electrostatics, vdW interaction, base pairing, base stacking, and stem angle to the total potential energy, respectively (see “Methods”). The rationality and robustness of our GA optimization are collectively corroborated by the approach to the maxima in the 5-parameter hyperspace (Supplementary Fig. 5), the consistency of fourfold cross-validation (Supplementary Fig. 6a–d), and the convergence of parameter sets from multiple independent optimizations (Supplementary Fig. 6e).

**Fig. 2:**
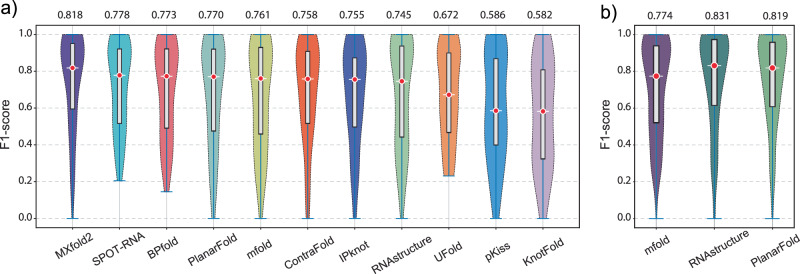
PlanarFold predicts RNA secondary structures. **a** Prediction performance of 11 RNA secondary structure prediction programs on the cross-sequence dataset (TS, *n* = 58 RNAs, Supplementary Fig. 2 and Supplementary Table 2). The blue whisker spans full data range. The median F1-scores are indicated by red spheres, with the corresponding values labeled above. The gray boxes represent the 25th percentile to 75th percentile. **b** Prediction performance of mfold, RNAstructure, and PlanarFold on the cross-family dataset (CrossFam1 and CrossFam2 combined, *n* = 67 RNAs for each subset, Supplementary Fig. 7). The average/median F1-scores for mfold, RNAstructure, and PlanarFold are 0.774/0.710, 0.831/0.750, and 0.819/0.753, respectively.

The optimized parameters exhibited satisfactory performance on the TS dataset, with the median (average) F1-score increasing from 0.620 (0.588) to 0.770 (0.681) (Supplementary Fig. 4d, e). Among the eleven tools evaluated, MXfold2, SPOT-RNA, and BPfold^32^—all deep learning-based—outperformed PlanarFold, achieving median F1-score of 0.818, 0.778 (*p*-value = 0.02, one-tailed paired *t*-test), and 0.773 (*p*-value = 0.004), respectively (Fig. 2a and Supplementary Table 2). The leading performance of MXfold2 could probably be credited to its implementation of thermodynamics regularization^30^, a strategy that integrates Turner’s nearest-neighbor free energy parameters^33^ into a DL network to minimize overfitting, and to a testing dataset lacking extensive tertiary and unnested interactions (pseudoknots). In the same vein, the incorporation of 36 base stacking thermodynamic parameters (Supplementary Table 3) into the base stacking energy term of PlanarFold has also benefited its holistic scoring even before GA optimization. With the fewest neural network parameters (~ 47,000) among the five DL-based predictors (Supplementary Table 4), MXfold2 bears the lightest overfitting on our dataset. In contrast, using a plain potential energy function of 6 terms with only 45 parameters in total (Supplementary Table 5), PlanarFold achieved a highly sequence-independent prediction performance (Supplementary Fig. 7a), which is comparable to or even better than more complex DL-based methods and traditional methods. Cross-sequence validation is common practice in the field; nonetheless, the risk of RNA homolog leakage exists in such tests^34^. To further evaluate PlanarFold’s ability to predict unseen RNA families under a more stringent condition, we rearranged the datasets using a homology-based split according to Rfam^35^ families and retrained the model (Supplementary Fig. 7b). Overall, PlanarFold achieved performance comparable to RNAstructure (*p*-value = 0.112) and mfold (*p*-value = 0.316) in cross-family prediction (Fig. 2b), demonstrating its generalization capability across RNA families.

Of note, conformer energies showed a preliminary correlation with F1-scores even prior to GA optimization (Supplementary Fig. 4c). After parameter optimization, PlanarFold energy exhibited a better correlation with F1-score than RNAstructure energy did for most RNAs (42 of 58, see Supplementary Fig. 8), demonstrating the impressive capacity of PlanarFold’s force field to distinguish the misfolded from the native. Furthermore, PlanarFold is capable of sampling multiple conformations. The top-3 and top-10 ensembles of PlanarFold improved the F1-score of TS dataset from 0.771 to 0.835 and 0.892, respectively (Supplementary Fig. 9), markedly higher than the best single-output predictors (MXfold2: 0.818).

### PlanarFold recapitulates the thermodynamics and kinetics

**Fig. 3:**
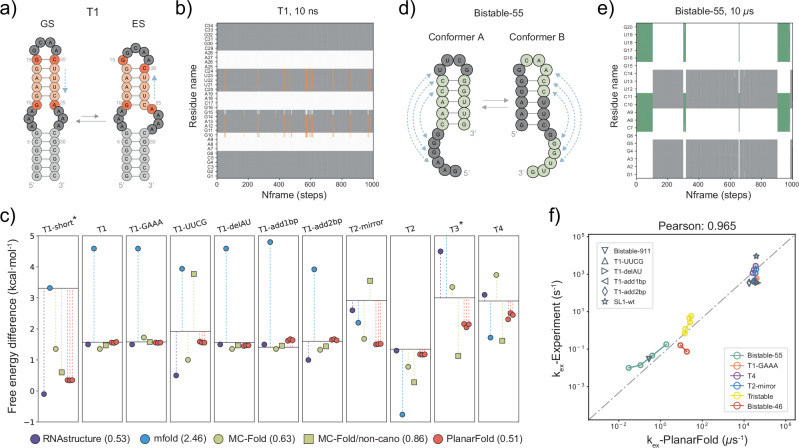
PlanarFold recapitulates RNA thermodynamics and kinetics. **a** The 1-nt register reshuffling within T1 hairpin, with the ground state (GS) and excited state (ES) labeled. **b** A 10-ns simulation trajectory of T1 displaying the secondary structure evolvement. Colored regions represent paired bases, with orange regions indicating the formation of the upper stem of ES. Unshaded regions indicate unpaired bases. See “Trajectory presentation” in Methods section for full details. **c** The performance of four software tools in predicting the free energy differences between the ES and GS of 11 hairpins undergoing base-pair reshuffling (Supplementary Table 7). Solid horizontal lines denote the experimental values measured by NMR. Predictions using MC-Fold with or without considering non-canonical base pairs are denoted by olive-colored circles or squares, respectively. Predictions from three independent PlanarFold simulations are shown as red circles. The RMSEs between the predicted and experimental free energy differences are indicated in parentheses (T1-short and T3, marked with an asterisk, are plotted but excluded from RMSE calculation due to large experimental uncertainties). **d** A bistable RNA (bistable-55) undergoing a strand-replacement (5bp-to-5bp) conformational transition. **e** A 10-µs simulation trajectory of bistable-55 displaying the secondary structure evolvement. **f** The exchange rates spanning six orders of magnitude for the tested RNAs with diverse transition modes (Supplementary Table 10). RNAs simulated at multiple temperatures are represented by circles connected by lines, and those at a single temperature by gray markers with different shapes. The fitted linear relationship is 1 µs (PlanarFold time) = 0.047 s (real time), with a Pearson correlation coefficient of 0.965.

The thermodynamic parameters of RNA, such as free energy, enthalpy, and entropy, determine how RNA molecules fold into their functional structures and undergo conformational exchanges, while the kinetics of RNA specifies the rates of those processes^36^. In the field of RNA dynamics, it is common practice to depict various conformational transitions of RNAs using a planar representation^37^. We speculate that these thermodynamics primarily governed by secondary structure changes may also be recapitulated by a 2D simulation model. Given that PlanarFold is able to sample diverse conformations, here we further examined its ability to estimate the thermodynamics of several experimentally verified RNA conformational transitions. NMR is one of the most powerful experimental tools to investigate RNA dynamics, and its recent advances have enabled unambiguous characterizations of some conformational exchanges^38^, such as the 1-nt register reshuffling of hairpin RNAs^39^ (Fig. 3a). Hence, we first benchmarked PlanarFold’s performance on characterizing these hairpin RNAs, and then assessed its efficacy for predicting more diverse conformational changes.

We compared PlanarFold’s prediction performance on thermodynamics with that of three conventional methods on 11 hairpin RNAs displaying μs-ms timescale base-pair reshuffling (Supplementary Fig. 10a). Since the parameters optimized by GA only stipulate the relative energy level of conformers, we fine-tuned the absolute energy difference of PlanarFold by fitting a single global scaling factor (Eq. (1), see “Methods”) to NMR-derived thermodynamic data^39^. In PlanarFold, the population of different states can be counted from a long, equilibrated trajectory (Fig. 3b), and can be further adjusted to match the corresponding experimental populations by tuning this factor that uniformly rescales the strength of all six potential terms. As only one parameter is fitted, a limited set of high-quality NMR measurements suffices for calibration—a strategy analogous to that used by RNAstructure, whose nearest-neighbor thermodynamic parameters were fitted using limited UV-melting experiments^33^. Ultimately, we chose a value of 0.33 for this scaling factor based on the thermodynamics of T1 RNA (Supplementary Table 6 and Supplementary Fig. 10b, see “Methods”). Importantly, such rescaling preserves the relative energies among conformers, thereby maintaining PlanarFold’s secondary structure prediction performance.

PlanarFold and RNAstructure demonstrated superior overall performance (Fig. 3c and Supplementary Table 7), with RMSE values of 0.51 and 0.53 kcal mol^−1^ for the 9 RNAs (excluding two RNAs, namely T1-short and T3, due to their large experimental measurement uncertainties^39^), respectively, followed by MC-Fold (RMSE = 0.63 kcal mol^−1^) and mfold (RMSE = 2.46 kcal mol^−1^). Notably, while mfold achieves secondary structure prediction accuracy comparable to RNAstructure, its poor thermodynamic prediction performance underscores the generalizability of PlanarFold in simultaneously resolving both structural and energetic features.

Following a temperature calibration procedure (see “Methods”), we investigated conformational exchanges across diverse transition modes at various temperatures. First, we tested the stem-loop 1 (SL1) of HIV, where a 2-nt register reshuffling of two adjacent base pairs occurs. PlanarFold predicted the free energy difference as 1.10 kcal mol^−1^, close to the experimental measurement^37^ (1.35 kcal mol^−1^; Supplementary Fig. 11a and Supplementary Table 8). Second, we studied three bistable RNAs^40–42^ harboring two major states that slowly interconvert through a global secondary structure rearrangement (Fig. 3d, e and Supplementary Fig. 11b–d). PlanarFold successfully reproduced the population ratios, despite the sampling challenges posed by the slow exchange dynamics. Third, we examined a tri-stable RNA that undergoes a nucleation-site-assisted transition (Supplementary Fig. 11e), and observed frequent exchanges between its two major states (A and B) but no detectable exchange with the third state (C), consistent with experimental observations^43^. Fourth, we simulated the stem-loop 3 (SL3) of 7SK RNA, which exhibits a 4-nt register reshuffling within its stem^44^ (Supplementary Fig. 11f). PlanarFold captured the two-state exchange within its P2 stem, although the ES population was underestimated (~ 3% by PlanarFold versus ~50% by NMR), which might be attributed to the effect of SL3’s complex dynamics. Interestingly, in addition to the experimentally verified slow exchange within P2 stem, we also identified a fast conformational exchange within its P3 stem.

PlanarFold can predict the impact of single point mutations on thermodynamics as well. We performed MD simulations for three P5abc constructs studied previously^45^: the wildtype and two mutants (G176A and U167C). In our simulations, the G176A mutation inverted the equilibrium between the ground state (GS) and the excited state (ES), whereas the U167C mutation abolished the ES, both in good agreement with the NMR experimental observations^45^ (Supplementary Fig. 12).

Encouraged by PlanarFold’s success in reproducing thermodynamics, we analyzed the kinetics of the cases tested above. Surprisingly, we observed excellent correlations between the overall exchange rates as well as their individual components (forward and backward rates) from PlanarFold simulations and NMR measurements (Fig. 3f, Supplementary Fig. 13, and Supplementary Table 10). PlanarFold’s reliable estimation of kinetic parameters across different transition modes spanning six orders of magnitude represents a notable achievement, since kinetics is determined by a continuum of transient transition states that remain elusive to experimental detection. Based on these results, the equivalent time of PlanarFold can be established: 20 µs of simulation approximately corresponds to 1 s (0.1–10 s, with a deviation of one order of magnitude) of motion in realistic time, suggesting a speedup of about 5 × 10^4^ times.

As a physics-based model, PlanarFold reproduced the thermodynamics and the more elusive kinetics that would be intractable for statistics-based CG models^16^. Here, we verify this by comparing PlanarFold with simRNA^19^, a statistics-based CG model. As expected, although simRNA could sample the GS and ES for those hairpin RNAs with register shifts, it tended to significantly overestimate the ES (Supplementary Fig. 14). Moreover, its predicted exchange rates correlate poorly with experimental ones (Supplementary Figs. 10 and 14).

### PlanarFold reproduces co-transcriptional folding pathways

In physiological conditions, RNA folding initiates as it is transcribed by RNA polymerase. During transcription, the partially transcribed strand often forms kinetically trapped intermediates, which can be persistent and serve as targets for several regulatory mechanisms^46^. Moreover, the progressive folding of nascent RNAs restricts the number of accessible folding pathways, presumably facilitating their rapid folding into the native structures^47^. Here, we tested PlanarFold’s ability to simulate co-transcriptional folding.

**Fig. 4:**
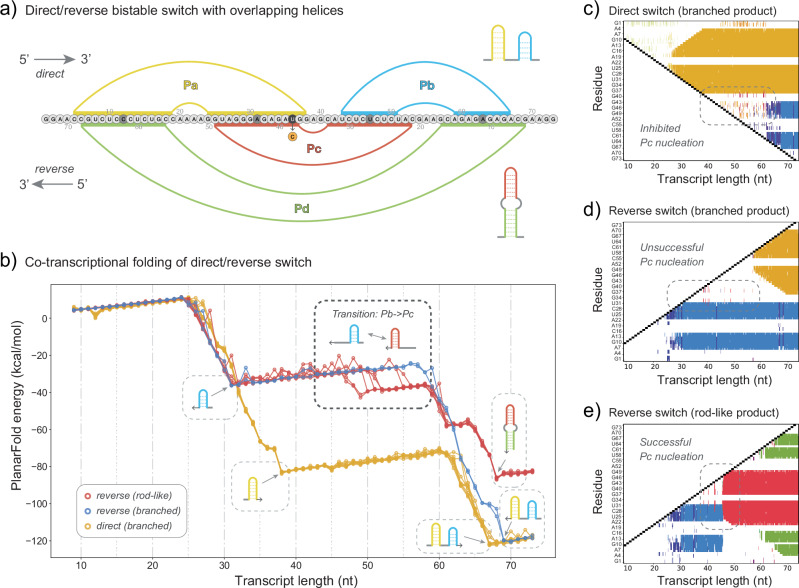
PlanarFold predicts the co-transcriptional folding of a pair of direct/reverse bistable switches. **a** The sequence of a pair of bistable switches in direct and reverse order, with their polarity and transcription direction labeled. The branched conformer contains helices Pa and Pb, while the rod-like conformer contains helices Pc and Pd. **b** The direct switch and reverse switch exhibit different folding pathways and end products during co-transcriptional folding. The transcription speed is 125 nt/µs. Ten trajectories are displayed for both switches. **c**, **d** 100% (40/40) of the direct switch and 15% (6/40) of the reverse switch fold into the branched conformer without helix replacement. Shaded and unshaded regions indicate paired and unpaired bases, respectively. **e** 85% (34/40) of the reverse switch fold into the rod-like conformer through the Pb-to-Pc transition occurring within the 45 to 57-nt window.

We investigated a pair of bistable RNA switches with sequences in direct and reverse order (referred to as the direct/reverse switch, respectively, hereafter). Experiments have shown^48^ that both switches can adopt two conformations with nearly identical free energies (branched and rod-like; Fig. 4a), but during co-transcriptional folding the direct switch yields exclusively the branched structure, whereas the reverse switch predominantly (~ 90%) forms the rod-like conformation. By adopting the equivalent time within the uncertainty range, the simulation speed of 125 nt/µs was chosen to match the transcription speed of ~250 nt/s for T7 RNA polymerase^48^ (Supplementary Table 11 and Supplementary Fig. 15). At this speed, the direct switch produced 100% branched conformers, while the reverse switch diverged into two folding pathways to yield predominantly rod-like conformers (Fig. 4b–e and Supplementary Table 11). Specifically, ~85% of the reverse switch in our simulations underwent a transition from hairpin Pb to Pc, employing a nucleation-site-assisted strand-replacing mechanism similar to the tri-stable RNA tested above (Supplementary Fig. 11e), and subsequently formed the rod-like conformation. A single-point mutation (U36C) disrupting the Pc-nucleation (Fig. 4a) decreased the rod-like population from 90 to 50% in the experiment, a trend reliably reproduced by PlanarFold (from 85 to 30%, Supplementary Fig. 15b).

Unlike most theoretical methods for predicting co-transcriptional folding^49,50^, PlanarFold, as an MD-based model, records intermediates and transient structures in single-molecule transcription events. In contrast to Kinefold^49^, which has facilitated the original design of direct/reverse switches and treats strand replacement as a one-step, abrupt transition with few intermediates (Supplementary Fig. 15g, h), PlanarFold captures the gradual replacement of old strands with nascent ones and occasional exchanges between competing strands (Pb and Pc, Fig. 4d, e). Such detailed trajectories allowed for more comprehensive analyses of strand replacement dynamics, including its dependence on both stability and accessibility. For instance, the nascent 3′ half of Pc largely remains unpaired in the reverse switch, favouring its attack on Pb, while the 3′ half and apical loop of Pc in the direct switch frequently pair with other loops, inhibiting Pc nucleation (Fig. 4c).

The validity of the co-transcriptional folding simulated by PlanarFold is further corroborated by another test on the *Escherichia coli* signal recognition particle (SRP). Without utilizing any experimental data, PlanarFold recapitulated all the key intermediates and strand replacement events in the SRP co-transcriptional folding pathway (Supplementary Fig. 16), which were detected by a recent method dependent on SHAPE experiments^51^. These results indicate that the dynamic ensemble of transcripts generated by PlanarFold could potentially be leveraged to design mutants and therapeutic drugs that modulate RNA functions by reshaping co-transcriptional folding pathways.

### PlanarFold captures the folding pathways and mechanical features

**Fig. 5:**
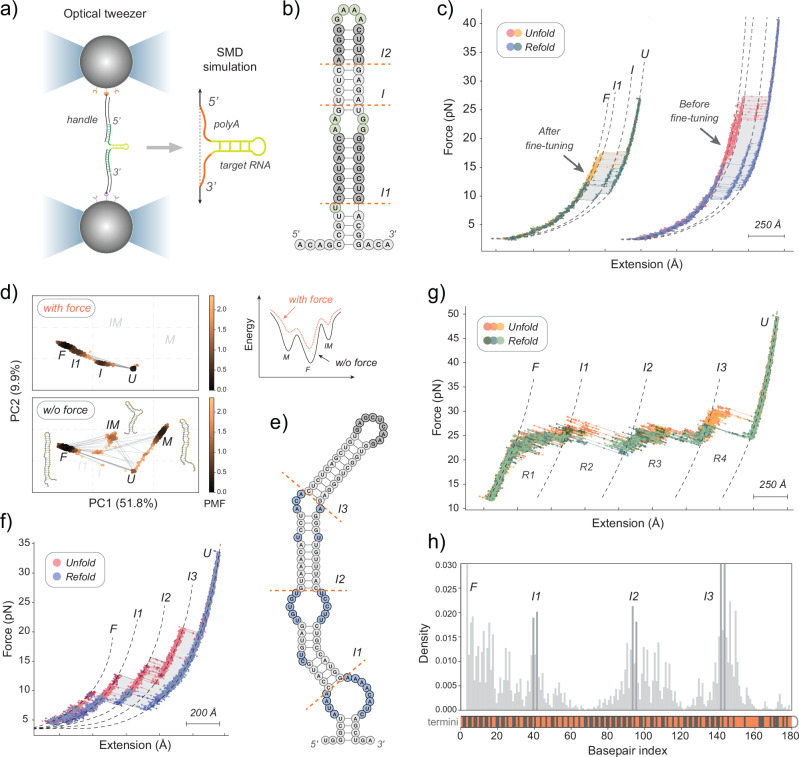
PlanarFold detects the intermediates characterized by optical tweezers. **a** The setup of the optical tweezers experiment and the molecule subjected to SMD simulation. For simplicity, instead of using kilobase-long RNA/DNA or DNA duplex handles (left), we added a 200-nt single-stranded RNA flanking poly(A) sequence to each terminus of the target RNA (right) to account for the effect of handle dynamics. **b** The sequence and secondary structure of P5ab, with three intermediates indicated (I1, I, I2). **c** Force-extension curves of P5ab before (ten unfolding and ten refolding curves, right) and after (five unfolding and five refolding curves, left) parameter fine-tuning. The extent of hysteresis is represented by the shaded area between unfolding and refolding curves. **d** Energy landscape of folding P5ab with and without external stretching force. Potential mean force (PMF) is in units of kcal mol^−1^. The folding starts at the unfolded state (U). In the lower panel, two kinetically trapped states are denoted as M and IM, and the representative structures of F, IM, and M are displayed. **e** The sequence and secondary structure of pri-miR-30c, with three intermediates indicated (I1, I2, I3). **f** Force-extension curves of pri-miR-30c (three unfolding and three refolding curves). **g** Force-extension curves of the 180-bp hairpin (three unfolding and three refolding curves), with three intermediates (I1, I2, I3) and four transition regions (R1–R4) labeled. **h** The occurrence of stalling sites (base pairs) during refolding and unfolding along the 180-bp hairpin, indicated by gray bars. The three intermediates I1, I2, and I3 are highlighted by dark gray bars. In the lower bar, 180 base pairs are indexed from the termini (left) to the apical loop (right) and are colored according to base pairing types: G-C/C-G as orange and A-U/U-A as gray.

RNAs typically fold hierarchically, with secondary structures forming first, followed by their assembly into higher-order conformations^4^. The latter process occurs on timescales of seconds to minutes for complex RNAs, allowing for measurement by various experimental techniques^52,53^. The faster secondary structure formation (microsecond to millisecond timescales), however, currently lacks effective experimental methods for per-residue resolution observation without external (mechanical, thermal, etc.) manipulation. OT, a single-molecule technique (Fig. 5a), have emerged as one of the most efficient and reliable tools for elucidating the mechanical folding and unfolding pathways of RNA^54,55^, capable of discerning co-existing pathways that are elusive to bulk methods. As an MD-based computational model, PlanarFold can offer a detailed, time-continuous view of RNA folding pathways. To validate the folding pathways explored by PlanarFold, we implemented steered MD (SMD) functionality to mimic the pulling and relaxing processes, and benchmarked the simulation results of several RNAs against their published OT measurements.

By virtue of PlanarFold’s enhanced sampling, we can approximate the OT experimental loading rates (~ 1–100 pN/s) relatively closely by using equivalent time, contrasting with other all-atom steered MD simulations^56,57^ that adopt loading rates 9–12 orders of magnitude higher (Supplementary Table 12). We initiated our test with a simple, short hairpin RNA, P5ab (Fig. 5b), which has been extensively benchmarked in prior OT experiments^58^. Previous OT studies have revealed three intermediates along P5ab’s unfolding and refolding pathways, two of which, I and I1, were successfully identified by PlanarFold (Fig. 5c, right). Discrepancies with the experimental data were also noted: the rupture forces were higher for unfolding (PlanarFold: 23–27 pN, experimental: 14–16 pN) while lower for refolding (PlanarFold: 10–15 pN, experimental: 13–15 pN), leading to stronger hysteresis; the hopping between the folded state (F) and completely unfolded state (U) was not observed; and the intermediates (I1, I) persisted longer. These discrepancies may stem from the unmatched loading rates, as higher rates have been reported to enlarge hysteresis^58^. The loading rates in PlanarFold’s simulations, while much closer to experimental values than all-atom simulations, remain 20-fold higher. Additionally, an oversimplified representation of the handle and inaccurate force parameters for the OT buffer may also account for the discrepancies, suggesting a need for fine-tuning the PlanarFold parameters to better mimic the OT experiment at higher loading rates (see “Methods”). Indeed, slightly weakening the strength of base pairing and partially shielding the charge improved agreement with the experimental data (Fig. 5c, left).

Despite the valuable insights into RNA folding pathways afforded by OT, whether these pathways, as probed under steering forces, diverge from those under unperturbed conditions remains an open question. Given P5ab’s structural simplicity and extensive OT characterization, we performed simulations of its de novo folding in the absence of the steering force to elucidate this question. One notable observation is that the initially fully unfolded P5ab molecule frequently fell into kinetic traps (M, IM) in the unperturbed folding landscape (Fig. 5d, bottom), which were nonetheless bypassed when external forces were applied (Fig. 5d, top), indicating that terminal tension reshaped the energy landscape of P5ab to favour pathways toward its native structure.

While both coarse-grained^59^ and theoretical models^60^ have been developed to characterize the mechanics of simple RNAs, they require either the native 3D structure or prior knowledge of mechanical features (such as ripping length and order) and thermodynamic data, rendering them unsuitable for ab initio prediction of RNA mechanics and folding pathways. To demonstrate that PlanarFold can simulate the mechanical unfolding and refolding of longer RNAs without any assumptions about their mechanics and thermodynamics, we benchmarked PlanarFold against a primary microRNA (pri-miR-30c), a 111-nt hairpin interspersed with five internal or bulge loops (Fig. 5e). In our simulations, PlanarFold recapitulated all three major intermediates validated by experiment^61^ (Fig. 5f). The identified checkpoints for unzipping and rezipping pri-miR-30c were large internal loops where continuous base stacking was disrupted.

Although it is intuitive and well-documented that folding tends to slow down near large internal loops, the determinants of folding behaviour within the RNA stem remain unclear. To investigate the specific determinants, we tested a 180-bp RNA hairpin free of any loops in the stem^62^. Strikingly, our SMD simulations captured all three intermediates probed by experiment, showing remarkable similarity with the experimental force-extension curve (FEC) pattern (Fig. 5g and Supplementary Fig. 17e–i). Upon inspection of the trajectories, the arresting sites were found to be preferentially located upstream of GC-rich regions (Fig. 5h), consistent with the enhanced stability of continuous G-C (or C-G) pairs through stronger base pairing and stacking interactions (Supplementary Table 3).

### De novo folding of large, complex RNAs

**Fig. 6:**
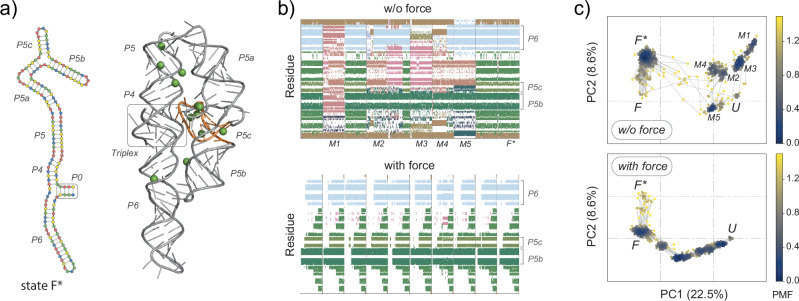
PlanarFold enables the de novo folding of P4–P6 domain from the group I intron. **a** The secondary and tertiary structures of P4–P6 domain (PDB ID: 1GID [10.2210/pdb1GID/pdb]). Magnesium ions are represented as green spheres. Due to the 2D representation, the native tertiary triplex (right) cannot form; instead, the 5' and 3' ends form an alternative helix P0 (left). We therefore consider both the experimentally observed structure (without P0, termed F) and its planar equivalent (with P0, termed F*) as native-like outcomes. **b** Ten repeats of P4–P6 de novo folding simulated without (top) and with (bottom) the external stretching force. Repeat trajectories are separated by vertical gray lines. Without steering force, 5 out of 10 trajectories fell into kinetic traps, resulting in 5 misfolded states (M1–M5). In contrast, under steering force, all 10 trajectories yielded native products (F or F*). Shaded and unshaded regions indicate paired and unpaired bases, respectively. **c** Folding energy landscape of the P4–P6 domain without and with the external stretching force. Potential mean force (PMF) is plotted in units of kcal mol^−1^.

Current computational models struggle to achieve de novo folding of large and complex RNAs^16^, both at the secondary and tertiary structure levels. Here, we demonstrate that PlanarFold can successfully fold the secondary structures of two large RNA systems: a well-studied domain of the group I intron ribozyme (P4–P6, Fig. 6a) and the aptamer domain of a Mg^2+^-sensing riboswitch (M-box, Supplementary Fig. 18a), starting from a linear conformation within a computationally affordable time frame. PlanarFold achieved a high success rate (50%) in de novo folding within 4 µs of simulation at 25 °C for both RNAs (Fig. 6b and Supplementary Fig. 18b). Building upon the foregoing finding that steering forces can suppress the formation of non-native structures when folding P5ab (Fig. 5d), we further validated that this observation also holds true for P4–P6 and M-box. Specifically, steering forces increased the refolding success rate to 100% for P4–P6 (Fig. 6b) and 80% for M-box (Supplementary Fig. 18b). Consistent with earlier observations on P5ab, the application of external stretching forces reshaped the energy landscapes of these complex RNAs as well, guiding them towards correct folding through a series of intermediates (Fig. 6c and Supplementary Fig. 18c).

Notably, these two RNAs stand out from our dataset as benchmarks for de novo folding, as they exhibited the ability to spontaneously anneal to and remain in near-native states during simulated annealing cycles (Supplementary Fig. 18d, e). These behaviours may reflect evolutionary optimization in some functional RNAs, such as P4–P6, a catalytic ribozyme, which serves to minimize folding time and avoid stable misfolded secondary structures^63^. According to PlanarFold’s approximation, the secondary structure of P4–P6 can form within several milliseconds at 25 °C, which is three orders of magnitude faster than its tertiary structure formation^52^ and is of the same order of magnitude as the formation of tRNA’s secondary structure (~ 2 ms, an estimation based on NMR measurements^64^). Notably, even for these two RNAs with unobstructed folding pathways, other coarse-grained models often fail to achieve correct de novo folding, becoming trapped in misfolded states (Supplementary Fig. 18f, g). In contrast, PlanarFold is able to fold long and complex RNAs from scratch without any secondary structure restraints. Although PlanarFold only operates at the secondary structure level, its success provides a foundation for future three-dimensional implementations to simulate the tertiary folding process of complex RNAs.

## Discussion

The secondary structure of RNA serves as the cornerstone of its folding dynamics, tertiary structure, and biological function. By integrating dynamic programming and dimension reduction into a single-bead-per-nucleotide representation, we devised a two-dimensional coarse-grained MD model, named PlanarFold. This MD model focuses on RNA dynamics at the secondary structure level. With a minimal set of parameters, PlanarFold demonstrates broad capacity to predict not only native RNA secondary structures, but, critically, also alternative conformations and their kinetics. Its fine temporal resolution and per-residue details enable the identification of short-lived intermediates and conformational switching events during transcription, including those that are elusive to OT. PlanarFold reproduces kinetic rates of diverse transitions across different temperatures with quantitative accuracy, underscoring its ability to discriminate between distinct transition modes and highlighting its potential for guiding the design of novel RNA-based devices.

The coarse-graining of one nucleotide into one single bead smooths the otherwise highly complex and rugged RNA energy landscape and dramatically accelerates conformational sampling. Meanwhile, the integration of dynamic programming ensures the stability of the RNA conformations through an explicit yet variable description of secondary structure, tackling a long-standing challenge confronted by coarse-grained MD model development. By incorporating DP, PlanarFold also circumvents the need for a complex form of empirical potential function to account for base pairing and base stacking interactions.

Despite PlanarFold’s success in reproducing multiple RNA characteristics, challenges still exist in broadening its general applicability. The intrinsic 2D nature of PlanarFold restricts its application to studying the formation of 3D structures and tertiary interactions, and limits its utility to investigating dynamics dominated by secondary structures. Due to the 2D geometric constraints of PlanarFold, many long-range pseudoknotted (or unnested) basepairs are also inaccessible in the planar representation. Therefore, PlanarFold is not suitable for the study of RNAs with high pseudoknot content. The RNAs benchmarked in this work either contain no pseudoknots or have a very low proportion (< 10%) of pseudoknotted base pairs. However, for short pseudoknot RNAs whose pseudoknots are planar-accessible, PlanarFold demonstrates better secondary structure prediction performance over traditional methods (Supplementary Fig. 19), suggesting the good generalization capability of PlanarFold’s force field. Nevertheless, as the dynamics of pseudoknots are more susceptible to the constraints of dimension reduction, PlanarFold remains primarily applicable to the dynamic study of nested (or non-crossing) secondary structures. The development of a 3D CGMD model dedicated to folding 3D RNA structures following a similar DP-assisted MD philosophy is currently underway.

Another limitation of our current model is that it neglects the sequence-dependent stability of various loops and non-canonical base pairs in both the dynamic programming framework and the potential function, primarily due to the scarcity of experimental thermodynamic data. Such data deficiency may explain the degraded performance of MC-Fold (RMSE = 0.86 kcal mol^−1^) in predicting thermodynamics when the energy contributions of non-canonical base pairs are considered (Fig. 3c and Supplementary Table 7). Leveraging available thermodynamic and statistical data, along with AI-driven fast quantum chemistry computations^32,65,66^, the energies of various loops and non-canonical base pairs might be estimated with higher accuracy. There are also two common hurdles for unified RNA model development: the heterogeneity of the experimental measurements and the lack of consideration for interacting molecules. The delicate energy landscapes and secondary structures of RNAs are highly susceptible to different experimental setups and a diverse array of external stimuli (e.g., ions, ligands, nucleic acids, and proteins)^9^.

As a proof-of-concept, we present PlanarFold as a versatile toolkit for investigating secondary structure-related features of RNA, with potential for future expansion to incorporate more functionalities, such as secondary structure prediction and dynamics characterization of circular RNA^67^, secondary structure visualization (Supplementary Fig. 20), incorporation of experimental restraints (e.g., SHAPE reactivities), and GPU-accelerated simulation of larger RNAs. We envisage that PlanarFold could promote the development of universal coarse-grained models for studying large-scale and slow RNA conformational changes, thereby advancing our knowledge of RNA dynamics-related phenomena.

## Methods

### Force field and dynamic programming

#### Force field functions and parameters

There are six potential function terms in PlanarFold’s force field (Eq. (1)). The details of the force field design and parameters are described in the Supplementary methods.

The bond term (Eq. (2)) is defined by a harmonic potential that depends on the inter-residue distance:

2 $${E}_{{{\rm{bond}}}}={k}_{{{\rm{bond}}}}\cdot {\left(r-{r}_{0}\right)}^{2}$$

where *r*_0_ and *k*_bond_ represent the equilibrium distance and spring constant, respectively.

The van der Waals term (Eq. (3)) and electrostatics term (Eq. (4)) adopt Lennard–Jones 12-6 potential and Debye–Hückel potential, respectively, to offer attraction and repulsion between non-bonded residues:

3 $${E}_{{{\rm{vdW}}}}={{\rm{\epsilon }}}\cdot \left\{{\left(\frac{\sigma }{r}\right)}^{12}-{\left(\frac{\sigma }{r}\right)}^{6}\right\}$$

4 $${E}_{{{\rm{elec}}}}=\frac{{{{\rm{Q}}}}^{2}}{r}\cdot {{{\rm{e}}}}^{-\frac{r}{{{\rm{\lambda }}}}}$$

For the vdW potential, *σ* represents the distance at which the potential is zero, while *ε* is the well depth; for the electrostatic potential, *Q* and *λ* denote the residue charge and the Debye length, respectively.

The base pairing, base stacking, and stem angle terms (Eqs. (5–7)), which are essentially harmonic restraints, are applied to maintain an ideal planar secondary structure geometry:

5 $${E}_{{{\rm{pairing}}}}={n}_{{{\rm{HB}}}}\left[i\right]\cdot \left[{k}_{{{\rm{pairing}}}}\cdot {\left(d-{d}_{0}\right)}^{2}+{p}_{{{\rm{pairing}}}}\right]$$

6 $${E}_{{{\rm{stacking}}}}={k}_{{{\rm{stk}}}}\cdot {k}_{{{\rm{stacking}}}}\left[i\right]\cdot \left[{\left(\psi -{\psi }_{0}\right)}^{2}+{p}_{{{\rm{stacking}}}}\right]$$

7 $${E}_{{{\rm{angle}}}}={k}_{{{\rm{angle}}}}\cdot {\left(\theta -{\theta }_{0}\right)}^{2}+{p}_{{{\rm{angle}}}}$$

where *k* and *p* are the spring constant and penalty/bonus of each potential. The variables d, ψ, and θ are indicated in Fig. 1b (right). In the base pairing potential, *n*_HB_ is the number of hydrogen bonds of the base pair and *d*_0_ represents the equilibrium distance. In the base stacking potential, *k*_stacking_ is assigned according to 36 Turner’s stacking parameters^33^ (Supplementary Table 3) and *ψ*_0_ is the equilibrium angle. In the stem angle potential, *θ*_0_ denotes the equilibrium angle. The values of all parameters are also listed in Supplementary Tables 3 and 5.

#### Dynamic programming

PlanarFold’s dynamic programming is a modified version of the classic Nussinov algorithm. The original Nussinov algorithm predicts RNA secondary structure by maximizing the base pair count, thus solely relying on the sequence. Its objective function, *S*(*i,j*), represents the maximum number of base pairs of an RNA sequence from residue *i* to residue *j*. If residue *i* and *j* can form a base pair, then

8 $${{\rm{S}}}\left(i,j\right)={{\rm{S}}}\left(i+1,j-1\right)+1$$

Otherwise,

9 $${{\rm{S}}}\left(i,j\right)=\max \big[{{\rm{S}}}\left(i+1,j\right),{{\rm{S}}}\left(i,j-1\right)\big]$$

If branching,

10 $${{\rm{S}}}\left(i,j\right)=\max \left[{{\rm{S}}}\left(i,k\right)+{{\rm{S}}}\left(k+1,j\right)\right],{{\rm{where\; i}}}\le {{\rm{k}}} < {{\rm{j}}}$$

In PlanarFold, the objective function *S*(*i,j*) depends not only on the sequence, but more importantly, on the contact map of the instantaneous coordinates. The objective function receives contributions from all base pairs:

$${{\rm{S}}}(i,j)=\max \left[\right.{{\rm{S}}}(i+1,j-1)+{{\rm{Cmap}}}(i,j),$$

$${{\rm{S}}}\left(i+1,j\right)+P,$$

11 $${{\rm{S}}}(i,j-1)+P\left]\right.$$

Compared with Nussinov’s version, PlanarFold’s DP replaced the base pair number (1 or 0) with the function Cmap(*i,j*), and introduced an extra penalty (P) for unpaired residues. For example, in a 3 × 3 internal loop, three non-canonical base pairs are allowed to form, yielding no unpaired residues and no penalty. However, in the case of a 3 × 4 internal loop or a 2-nt bulge loop (0 × 2), the presence of one or two unpaired residues results in one or two penalties, respectively. But importantly, the canonical base pairs only contribute to the *S*(i,j) in DP, but are not included in the secondary structure to calculate the potential energy (see Supplementary methods). The value of P was determined by matching the thermodynamics of hairpin T1 and T4 to experimental data; these two hairpins contain a symmetric internal loop and a 1-nt bulge loop, respectively. The resulting value is 0.02 (see below).

The function Cmap(*i,j*) depends on the instantaneous contact map:

12 $${{\rm{Cmap}}}\left(i,j\right)={{\rm{InvD}}}\left(i,j\right)\cdot {{\rm{BpFlag}}}\left(i,j\right)$$

InvD(*i,j*) is the reciprocal of the square of distance:

13 $${{\rm{InvD}}}\left(i,j\right)=\frac{1}{{{\rm{dis}}}{{{\rm{t}}}}^{2}\left(i,j\right)}$$

BpFlag(*i,j*) represents the weight for different types of base pairs: it takes values of 6, 4, 4, 2, and 1 for G-C, A-U, G-U, G-A, and other base pairs, respectively. These weights are approximately proportional to the number of hydrogen bonds involved in each base pair type. More specifically, the base pairs G-C, A-U, and G-U contain 3, 2, and 2 stable hydrogen bonds, respectively. Since PlanarFold is prone to pair any two opposite residues in the internal loop and bulge loop to avoid asymmetry and optimize the objective function *S*(i,j), we assigned 0.5 hydrogen bonds to all non-canonical base pairs except for G-A. The G-A base pair, being the most abundant non-canonical base pair^68^, usually adopts sheared or imino-bonded type, each containing two hydrogen bonds. Nevertheless, given its lower energetic stability compared to canonical pairs, we assigned it one hydrogen bond.

It is worth noting that PlanarFold eliminates the need to consider the scenario of branching as required by the original Nussinov algorithm. To achieve this, PlanarFold first partitions the contact map into independent regions (Fig. 1c), then employs its DP algorithm to search for the minimum sum of distances within each helix region. Detailed implementation of DP can be found in the Supplementary methods.

### Parameterization and secondary structure prediction

#### Collection of training and testing datasets

We collected a testing dataset with sequence similarity lower than 80% to the training data used for the three DL-based predictors (Supplementary Fig. 2). The specific procedure is detailed as follows. First, we collected all the datasets of SPOT-RNA, MXfold2, and UFold, and merged them into a single dataset named DL-nonRedundant (Supplementary Table 1). After removing identical sequences, we obtained about 40,000 RNA sequences in DL-nonRedundant, serving as the reference for sequence similarity filtering. Next, we collected the “solo RNAs” (length >10 nt and resolution higher than 4 Å) from RNAsolo [https://rnasolo.cs.put.poznan.pl/]^69^ database for cross-sequence validation. A sequence similarity filtering was then performed using CD-HIT-EST-2D^70^ to generate the RNAsolo-unique dataset. RNAsolo-unique is a diverse repository containing RNAs of various topologies, with half of them (211 of 418) featuring multi-branched topologies. On the other hand, we compiled a dataset named Rfam-unique from Rfam 14.9 using the same filtering procedure as in previous studies for cross-family validation^71^. Rfam-unique contains only 3 branched RNAs among 837 entries, with the vast majority of them belonging to microRNA families with short hairpin conformations, which is far too simple and unrepresentative for a cross-family test. Therefore, the cross-family comparison is conducted by re-splitting the testing dataset by Rfam families instead. Ultimately, 216 and 58 representative RNAs were compiled as our training (TR) dataset and testing (TS) dataset from DL-nonRedundant (SPOT-RNA’s PDB datasets) and RNAsolo-unique, respectively, with filtering criteria of high base pairing ratio (> 40%) and low unnested pairing ratio (< 10%) to exclude RNAs with extensive tertiary and unnested interactions. We manually verified all PDB structures and FASTA sequences of TR and TS RNAs, and cross-referenced them with original papers when necessary, to ensure accurate annotation of the secondary structures.

#### Generation of conformation pools

We constructed the conformation pools from two sources to ensure broad sampling. First, we generated 1000 unrestrained conformers for each RNA using 1000 unrestrained simulated annealing cycles (i.e., heating and cooling). Second, we employed Fold module in RNAstructure to generate representative secondary structures as restraints for the restrained conformational sampling of PlanarFold (Supplementary Fig. 3). All conformers were cooled to 0 K to eliminate geometric tension and thermal fluctuations, and the restraints were released when approaching the target secondary structure. In doing so, we collected the top 500 structures across four window sizes (Fold parameter: window = 1, 2, 3, and 4) to ensure a comprehensive conformation pool while keeping the computational cost manageable for subsequent MD simulations. Identical secondary structures sampled from different window sizes were merged. We generated 5 conformers under each secondary structure restraint using simulated annealing cycle, as PlanarFold can sample multiple conformers for a single secondary structure due to loop flexibility. Notably, for those long RNAs that were sampled in only “far-native” states (F1-score <0.2), manual addition of native conformers into the conformer pool did not improve their poor prediction performance, as these native conformers consistently scored significantly lower than their low-energy decoys. This phenomenon likely originates from dense protein-RNA interactions in these long RNAs (exemplified by a large ribosomal RNA, 8a22_4, in our testing dataset).

#### Parameter optimization for secondary structure prediction

The initial choice of force field parameters was guided by referencing existing RNA CG models or was determined through iterative manual adjustments based on MD trajectory analyses. Subsequently, we employed both genetic algorithm (GA) and Bayesian optimization for parameterization, and eventually chose one set of parameters from the GA-optimized candidates. The objective function of the GA optimization is the median F1-score of the TR dataset (216 RNAs). We chose the median F1-score over the average F1-score because it is less susceptible to outliers in the data, although using the average F1-score as the objective function yielded similar results. The F1-score for each RNA was calculated from the conformer with the lowest potential energy (using GA-optimized parameters) within its conformation pool. The potential energies were decomposed to enable independent rescaling of different terms (Eq. (1)) during GA optimization. The five optimized parameters can be classified into two types: those affecting the gradient calculation of potential function and those that do not. The former includes charge (*Q*), strength of base stacking (*k*_stk_), and epsilon of the vdW interaction (ε), while the latter includes penalty terms for base pairing (*p*_pairing_) and stem angle (*p*_angle_). Other parameters, such as bond strength (*k*_bond_), contribute marginally to F1-score improvements due to their small decomposed energies. To avoid unnecessarily expanding the parameter searching space during optimization, these parameters were fixed with manually selected values.

#### Evaluation of secondary structure predictors

The secondary structures for the native states were annotated by DSSR^72^ based on PDB files and were checked manually. Only stems, defined as a helix of at least two continuous canonical base pairs, were kept in the output secondary structures. Since PlanarFold predicts only canonical base pairs, all other non-canonical base pairs were also removed. All apical loops smaller than 4 nt were replaced with unpaired loops, as many previously annotated 2- and 3-nt apical loops either lack a canonical closing base pair or arise from missing residues in PDB files. To remove pseudoknots present in naturally occurring structures and those generated by various predictors (SPOT-RNA, UFold, pKiss, etc.), we used the bpRNA^68^ tool to annotate the pseudoknot-free secondary structure with the maximum number of base pairs. Our data filtering protocol requires that pseudoknotted base pairs constitute less than 10% of the total, ensuring their removal would have a negligible effect on our training and testing datasets. The webservers or packages of the predictors we benchmarked are listed above: MXfold2^30^ (available at https://ws.sato-lab.org/mxfold2/), SPOT-RNA^29^ (available at https://github.com/jaswindersingh2/SPOT-RNA), BPfold^32^ (available at https://github.com/heqin-zhu/BPfold), mfold^28^ (available at https://www.unafold.org/mfold/applications/rna-folding-form-v2.php), ContraFold^73^ (available at http://contra.stanford.edu/contrafold/), IPknot^74^ (available at https://ws.sato-lab.org/rtips/ipknot++/), RNAstructure^27^ (available at http://rna.urmc.rochester.edu/RNAstructure.html), UFold^31^ (available at https://github.com/uci-cbcl/UFold), pKiss^75^ (available at https://wwww.cebitec.uni-bielefeld.de/bibiserv.cebitec.uni-bielefeld.de/pkiss.html), KnotFold^76^ (available at https://github.com/gongtiansu/KnotFold).

### Thermodynamics and kinetics

#### Parameter rescaling for predicting free energy differences and temperature calibration

The free energy difference between two conformations can be derived from the Boltzmann distribution, as given in Eq. (14),

14 $$\Delta \Delta G=-{RT}\cdot {\mathrm{ln}}({{{\rm{p}}}}_{{{\rm{ES}}}}/{{{\rm{p}}}}_{{{\rm{GS}}}})$$

where *R* is the gas constant, and T is the temperature in Kelvin. The population of ES and GS is indicated by *p*_ES_ and *p*_GS_, respectively. In experiments, the free energy difference between GS and ES is obtained from their populations measured by NMR (or other methods). In MD simulation, the ratio of GS and ES can be determined from a long equilibrium simulation. The scaling factor rescales the conformer energies proportionally. A larger scaling factor (> 1) increases the absolute energy difference between GS and ES, while a smaller one (< 1) does the opposite. A value of 0.33 best reproduces the experimental populations of GS and ES for T1 RNA (Supplementary Fig. 10b).

In order to investigate the dynamics at various temperatures, we calibrated the temperature of PlanarFold based on NMR measurements of a hairpin RNA (T1-GAAA) at three temperatures (5, 10, and 15 °C). This calibration established a linear correlation between the experimental temperature and PlanarFold’s simulation temperature (Supplementary Fig. 10c and Supplementary Table 9), enabling reliable prediction of dynamics at various temperatures.

#### Evaluation of thermodynamics and kinetics

For RNAstructure and mfold, the free energies of ES and GS were predicted at the corresponding experimental temperatures (e.g., 283 or 298 K) by using their respective secondary structures as input. For MC-Fold, two types of prediction were made: considering non-canonical base pairs or not. To include non-canonical base pairs, one simply needs to change the restraint of “unpaired” (indicated as dots in the *dbn* file format) into “unconstrained”, allowing unpaired residues to either form non-canonical base pairs or remain unpaired. In PlanarFold, the free energy differences between two states were calculated using Boltzmann statistics, while the exchange rates were determined as: *k*_ex_ = *k*_forward_ + *k*_backward_. The forward (backward) rates, defined as the rates from state A to B (state B to A), were determined by dividing the number of forward (backward) transitions by the dwell time at state A (state B). The webservers we benchmarked are listed above: RNAstructure^27^ (Fold module, available at http://rna.urmc.rochester.edu/RNAstructure.html), mfold^28^ (available at https://www.unafold.org/mfold/applications/rna-folding-form-v2.php), MC-fold^77^ (available at https://major.iric.ca/MC-Fold/).

#### Trajectory presentation

We devised a scheme to monitor the secondary structure evolution along the MD trajectory (Fig. 3b and e, etc.). Each residue is colored according to the sum of its own index and its paired residue’s index, with unpaired residues assigned a value of 0. For example, in GS of T1 RNA, residue 11 is labeled 35 ( = 11 + 24), as it pairs with residue 24. Likewise, residue 24 is also labeled 35. The elegance of such a presentation is that residues in the same stem are identically labeled and colored. In this way, all five base pairs in the upper stem of T1-GS are labeled 35 (11 + 24, 12 + 23, etc.). For T1-ES, where the upper stem shifts by one nucleotide in register, the corresponding residues will be labeled 34 (10 + 24, 11 + 23, etc.). This coloring scheme allows immediate recognition of shifted regions, as exemplified in Fig. 3b. We applied this scheme to all the secondary structure trajectories in this study to effectively highlight conformational changes across all examined cases.

### Mechanics and folding pathway

#### Fine-tuning to match OT buffer conditions

The parameters refined in the thermodynamics section are based on free energy differences measured in NMR buffer, which usually differ from OT buffer. The OT buffer for P5ab consists of 250–300 mM NaCl or KCl and 3–20 mM MgCl_2_^58^, while the NMR buffer of 11 hairpins with register shifts contains 10 mM sodium phosphate and 0.01 mM EDTA^39^. Clearly, the OT buffer contains more monovalent ions and additional divalent ions, providing a more shielded environment for the RNA. Therefore, we partially shielded the residue charges, reducing their value from 2.93 to 1.0 kcal^1/2^ Å^1/2^ mol^−1/2^ (equivalent to changing from 0.16 to 0.055 point charge). In addition, the unzipping force is higher than the experimental value, which might indicate an overestimation of the base pairing interaction, so we decreased the strength of base pairing (*k*_pairing_) from 0.4 to 0.3 kcal mol^−1^ Å^−2^. The overestimation of the base pairing interaction might result from double counting of the base pairing interaction in PlanarFold, as PlanarFold’s base stacking strength is proportional to Turner stacking parameters, which have already considered both base stacking and base pairing interactions. Further independent optimization of the 36 Turner stacking parameters to separate the contribution of base stacking and base pairing may alleviate these issues, but has proven impractical due to the large parameter space and scarce data. Therefore, those fine-tuned parameters are employed for the following simulations of pri-miR-30c and the 180-bp hairpin as well, to match the higher pulling speeds (20-fold higher than their corresponding experimental speed).

#### Acceleration for SMD

If the target RNA is connected to a 200-nt flanking poly(A) sequence at both termini, the full construct for simulation will exceed 400 nt. The most computationally expensive part of PlanarFold is the determination of secondary structure by dynamic programming. Nonetheless, most computation time is wasted on the flanking poly(A) sequences, which are added only to reproduce the fluctuation of RNA/DNA duplex handles rather than to form any meaningful base pairing. To avoid unnecessary computations involving poly(A) handles forming base pairs, a restraining strategy was implemented in PlanarFold. We named this type of MD simulation “confined MD”, with a controlling parameter “*dpr*”. Confined MD requires pre-defined secondary structures, from which base pairs can be extracted. Only those prescribed base pairs can form and break during the MD simulation, according to a simple criterion of whether their distances are within the cutoff, thus circumventing the usage of dynamic programming. We employed this confinement strategy for nearly all constructs with 200-nt flanking handles to accelerate simulations, while RNAs free of handles (or with short handles) were simulated without such confinement. Furthermore, to better mimic the refolding process, which heavily depends on the nucleation of apical loops, we added a weak constant harmonic restraint (controlled by the parameter “*ntr*”) on the native apical loop of pri-miR-30c and the 180-bp hairpin. Removal of these restraints causes a significantly delayed nucleation of apical loops and the subsequent formation of numerous non-native apical loops and hairpins (Supplementary Fig. 17f–i).

#### SMD setups and post-processing

The simulated pulling or relaxing speed for P5ab, pri-miR-30c, and the 180-bp hairpin was set to 4000, 1000, and 1400 nm µs^−1^, respectively, to match the OT experiment speed as closely as possible, according to our equivalent time scale (i.e., 1 µs in simulation is equivalent to 5 × 10^4^ µs in reality). The exact experimental speed (20-fold slower than the current setting after equivalent time conversion) is computationally unaffordable at present but may become feasible with future GPU acceleration. The spring stiffness values for P5ab, pri-miR-30c, and the 180-bp hairpin were set to 0.038, 0.5, and 0.1 pN nm^−1^, respectively, based on values from either original literature or deduced values from their force-extension curve (FEC) data. The FECs in this study were typically averaged over a window of 100 or 1000 points to suppress noise.

#### Principal component analysis of PlanarFold conformations

Our principal component analysis (PCA) is based on the contact map Cmap(*i,j*) of each conformation (Fig. 1c, right). The top-right triangle of the contact map was flattened into a one-dimensional vector for PCA. The contact cutoff was set to 14 Å (corresponding to the default base pairing cutoff). However, it should be noted that these PMF values do not represent the true free energy differences between clusters (Figs. 5d and 6c), since sampling for such large conformational changes is non-ergodic even for PlanarFold.

#### Analysis of arresting sites of the 180-bp hairpin

As PlanarFold generated trajectories with explicitly recorded conformations, we could unambiguously locate the last (terminal) base pair that bears the steering force. Compared to fitting intermediates from the FEC, determining intermediates from trajectories proves more definitive (Fig. 5h).

#### Force-steered refolding of two large RNAs

To study refolding, we used constant-velocity SMD rather than constant-force mode. While constant force at ~15 pN hindered proper folding, gradually decreasing the extension allowed the force to drop smoothly from ~15 pN, enabling native structure formation.

### Reporting summary

Further information on research design is available in the Nature Portfolio Reporting Summary linked to this article.

## Supplementary information

Supplementary Information

Description of Additional Supplementary Files

Supplementary Data 1

Supplementary Data 2

Reporting Summary

Transparent Peer Review file

## Source data

Source Data

## Data Availability

The public datasets used for secondary structure benchmark are listed in Supplementary Fig. 2 and Supplementary Table 1, which are originally from the PDB [https://www.rcsb.org] or RNAsolo [https://rnasolo.cs.put.poznan.pl/archive]. The original datasets of SPOT-RNA^29^, MXfold2^30^, UFold^31^ listed in Supplementary Table 1 and Supplementary Fig. 2 are publicly available at [http://sparks-lab.org/jaswinder/server/SPOT-RNA/], [https://zenodo.org/records/4430150], and [https://github.com/uci-cbcl/UFold], respectively. The training dataset (TR, 218 RNAs) and testing dataset (TS, 58 RNAs) of PlanarFold are provided in Supplementary Data 1 and Supplementary Data 2, respectively. The detailed secondary structure prediction results are provided in Source Data. The following PDB structures were used in the figures, with their accession codes and DOI links: 1GID, 2QBZ, and 1ZIH. The various experimental data (optical tweezers, NMR, etc.) used for validation of PlanarFold were obtained from previously published studies. Specifically, the NMR experimental data for the 11 T1-related hairpin RNAs were obtained from ref. ^39^; the SL1 of HIV from ref. ^37^; the bistable RNAs from refs. ^40–42^; the tri-stable RNA from ref. ^43^; the SL3 of 7SK RNA from ref. ^44^; and the P5abc from ref. ^45^. The co-transcriptional folding experimental data of direct-reverse switch and SRP RNA were obtained from ref. ^48,51^. The optical tweezers experimental data of P5ab, pri-miR-30c, and 180-bp hairpin were obtained from refs. ^58,61,62^, respectively. The source data of trajectories and output files of the main figures are deposited on Zenodo [10.5281/zenodo.19985095]^78^. Other numerical data for the main figures and Supplementary Figs. and tables are provided in the Supplementary Information and Source Data. The parameters and reproduction procedures for benchmark tests of PlanarFold can be found in the Supplementary methods and Supplementary notes. More detailed instructions to use the software are described in the User Manual deposited on Zenodo [10.5281/zenodo.19397229]^79^ and GitHub [https://github.com/Lanxiang-thu/PlanarFold]. Source data are provided with this paper.

## Code availability

The standalone PlanarFold package, source codes, custom analysis scripts, and related benchmark setups are available at Github [https://github.com/Lanxiang-thu/PlanarFold] and Zenodo [10.5281/zenodo.19397229]^74^.
