# Supplementary material for: PlanarFold: a coarse-grained molecular dynamics model of RNA in two-dimensional space
Source: Nat Commun. 2026 Jun 26;17:7998. doi: 10.1038/s41467-026-74729-y (PMC13447859; doi:10.1038/s41467-026-74729-y)
Supplement: Supplementary file 1 [file 41467_2026_74729_MOESM1_ESM.pdf]

Supplementary Information

PlanarFold: a coarse-grained molecular dynamics model of RNA in two-dimensional space

Lan Xiang[1], Yi Xue*[1,2]

[1] Beijing Frontier Research Center for Biological Structure; School of Life Sciences, Tsinghua University, Beijing, 100084, China

[2] State Key Laboratory of Complex, Severe, and Rare Diseases, Beijing, 100084, China

Table of contents

Supplementary methods 4

Force field of PlanarFold 4

Molecular dynamics simulation: general parameters 6

MD simulations for predicting secondary structures 6

MD simulations for predicting free energy differences and kinetic rates 7

MD simulations for co-transcriptional folding 7

MD simulations for mechanical unfolding and refolding 8

Dynamic programming 10

Supplementary figures 12

Supplementary Figure 1. The framework of PlanarFold: MD incorporated with DP. 12

Supplementary Figure 2. Collecting training (TR) and testing (TS) datasets of PlanarFold. 13

Supplementary Figure 3. Pipeline for predicting RNA secondary structure using PlanarFold. 14

Supplementary Figure 4. Conformation pools sampled by PlanarFold for the TR and TS. 16

Supplementary Figure 5. Approaching local maximum after genetic algorithm optimization. 17

Supplementary Figure 6. Test of the robustness of GA optimization. 18

Supplementary Figure 7. Cross-family validation of secondary structure prediction. 19

Supplementary Figure 8. Correlation between F1-score and energy from PlanarFold and RNAstructure. 20

Supplementary Figure 9. Multiple prediction outputs of PlanarFold and RNAstructure. 22

Supplementary Figure 10. Thermodynamics benchmarking on short hairpins. 25

Supplementary Figure 11. Simulation results of transitions using PlanarFold and simRNA. 27

Supplementary Figure 12. Simulation results of three constructs of P5abc using PlanarFold. 28

Supplementary Figure 13. The forward and backward rates simulated by PlanarFold. 28

Supplementary Figure 14. Simulation results of transitions using PlanarFold and simRNA. 29

Supplementary Figure 15. Comparison of PlanarFold and KineFold. 30

Supplementary Figure 16. Co-transcriptional folding of E. coli SRP using PlanarFold. 33

Supplementary Figure 17. Benchmark on P5ab and a 180-bp hairpin. 34

Supplementary Figure 18. Benchmarking de novo folding on two RNAs using PlanarFold and simRNA. 36

Supplementary Figure 19. Secondary structure prediction of 15 pseudoknot RNAs. 38

Supplementary Figure 20. Plotting RNA secondary structure using different tools. 39

Supplementary Figure 21. Running time versus RNA length. 40

Supplementary tables 41

Supplementary Table 1. Datasets used for deep-learning based predictors. 41

Supplementary Table 2. F1-score of eleven predictors on testing dataset. 41

Supplementary Table 3. The base-stacking parameters for 36 bp-steps. 42

Supplementary Table 4. Number of parameters of different models. 42

Supplementary Table 5. Force field parameters of PlanarFold. 42

Supplementary Table 6. Fine-tuning energy difference by adjusting the force-scaling factor. 43

Supplementary Table 7. Predicted free energy differences of the hairpins with register shift. 43

Supplementary Table 8. Predicted free energy differences of transitions with diverse modes. 44

Supplementary Table 9. Temperature calibration based on T1-GAAA. 45

Supplementary Table 10. Kinetic rates of diverse transition modes across large timescale. 46

Supplementary Table 11. End products of co-transcriptional folding and renaturing folding. 47

Supplementary Table 12. Loading conditions of SMD simulations and optical tweezers experiments. 48

Supplementary Table 13. Sequences and secondary structures of benchmarked shuffling RNAs. 48

Supplementary Table 14. Sequences and secondary structures of benchmarked RNAs. 49

Supplementary notes 50

Supplementary references 58

Supplementary methods

Force field of PlanarFold

We chose C1' atom as the representative bead of the nucleotide, as we recognized that the (canonical) base pairing plane in a stem always aligns with C1' atoms, as C1' is directly connected to either a pyrimidine (for U, C) or a purine (A, G) ring. The C1'-C1' vector of a base pair is generally in the same plane as the pyrimidine-purine plane, giving it a relatively conserved distance (~ 10.5 Å in a stem). Of note, the C1'-C1' distance between two consecutive nucleotides has a converved distance of ~5.5 Å in the stem, but varies dramatically in loop regions (from ~4 Å to ~8 Å).

There are 6 potential terms in PlanarFold: bond, van der Waals, electrostatics, base-pairing, base-stacking, and stem-angle.

The initial choice of force field parameters (equilibrium values and force constants) was guided by referencing to all-atom force fields, existing RNA CG models, or determined through iterative manual adjustments based on geometry and trajectory stability. The force constant is adjusted to be neither too strong, which would trap the trajectory in misfolded structures easily, nor too weak, which would make any structure unstable. Subsequently, five parameters (ε, Q, $p_{pairing}$, k_{stk}, p_{angle}) are determined by genetic algorithm optimization in secondary structure prediction tasks to distinguish the misfolded from the native.

All the values of force field parameters are also listed in Supplementary Tables 5 and 3.

The bond term is a harmonic distance term (Eq. 2). All the bonds share the same equilibrium value (r_0) of 5.5 Å, which is the median length of C1' atom between two consecutive residues in 3D space. Although such a Euclidean distance is in 3D space, we try to keep the 2D geometry of the base-pair similar to their 3D geometry. All the bonds share the same force constant (k_{bond}) of 20.0 kcal mol^{-1} Å^{-2}, which is chosen to be slightly weaker than the bond length of most bonds in the all-atom force field to mimic a softer bond between two consecutive residues.

The van der Waals term employs Lennard-Jones 12-6 potential (Eq. 3). All the beads (A, U, C, and G) share the same parameters. The well depth (ε) is 1.1 kcal mol^{-1}. The σ is the distance at which potential is zero, which is set to 9.8 Å. The vdW (and electrostatic) interaction between two consecutive residues is excluded. The value is chosen such that the RNA single chain should basically maintain linear meanwhile also has the tendency to fold back to form base pairing: the upper limit of the distance between residue[i] and residue[i+2] is ~11.0 Å (which is two C1'-C1' bond length), and the lower limit of their distance is determined by parameter σ. The cutoff for vdW interaction is 13.5 Å. No cutoff tail correction or smoothing is employed.

The electrostatics term employs Debye-Hückel potential (Eq. 4), which is widely adopted in coarse-grained modeling to replace the long-range Coulomb force. Such potential is short-ranged by assuming a Debye length (λ) of 10 Å, by referring to a 3D RNA CG model (RACER[1]). The cutoff for electrostatic interaction is 20.0 Å (which is larger than vdW cutoff, to better consider the electrostatics of highly charged RNA molecules). All the beads share the same charge (Q) of -2.93 $kcal^{1/2}$ $mol^{-1/2}$ Å$^{1/2}$ (equivalent to 2.93/18.22 = 0.161 e). The charge might require fine-tuning given the ion concentration of the corresponding experiment condition. For instance, we decrease the net charge from -2.93 to -1.0 when simulating the buffer condition of optical tweezers experiment which uses a higher ion concentration than NMR buffer.

The base-pairing term is a harmonic distance term (Eq. 5) applied to base pairs. Along with other two terms (base-stacking and stem-angle), the base-pairing term is only applied to residues that are determined to be paired by dynamic programming (DP). All base pairs share the same equilibrium distance (d_0) of 10.0 Å. The median length of the C1' atom between two pairing residues is ~10.5 Å in 3D space. In PlanarFold, we chose a slightly short distance to better maintain the stability of the "stem" (defined as a helix comprised of at least two consecutive canonical base pairs). The force constant ($k_{pairing}$) is 0.4 kcal mol^{-1} $Å^{-2}$. To distinguish the different stability of different types of base pair, we assigned a factor, "nHB", for different base pairs. Specifically, nHB is 3, 2, and 2 for GC, AU, and GU base pair, respectively, which is determined by their corresponding hydrogen bond number. Since a harmonic energy term is always a positive (or zero) value, a suddenly formed base-pairing (and base-stacking, stem-angle) term will cause an increase in the energy (compared to unpaired state). So we introduced a penalty/bonus parameter ($p_{pairing}$) for base-pairing (base-stacking, and stem-angle) term to compensate for the energy increase. The base-pairing penalty/bonus ($p_{pairing}$) is 9.6 kcal mol^{-1}. Of note, a positive value of $p_{pairing}$ from genetic algorithm optimization result is against our initial assumption (i.e., a negative one), as it suggests a suddenly formed base pair will cause increase in the energy. However, this is a misconception. When the secondary structure changes during simulation, the energy contribution of forming a new base pair must be considered along with the other two suddenly applied terms (i.e. base-stacking and stem-angle). The base-stacking penalty/bonus ($p_{stacking}$) and stem-angle penalty/bonus (p_{angle}) are both negative values, which will compensate the positive contribution of base-pairing terms. Therefore, it is the total potential energy rather any individual potential term of a conformer that is used to determine the stability.

The base-stacking term is a harmonic angle term (Eq. 6) applied to two consecutive base pairs (also dubbed as bp-step). In 3D space, the stacking can happen between two close nitrogenous base planes. In the stem, two adjacent base pairs form stacking. For instance, for a bp-step of AU-CG (the sequence is 5'-AC…GU-3'), we applied 4 right angle terms (the equilibrium angle ψ_0 is 1.57079633 in radian, i.e., 90 in degree) to achieve the bp-step stacking such that each bp-step is in rectangle geometry on plane. They are A-U-C, U-A-C, A-C-G, and U-G-C (such 4 angles are also indicated in Fig.1a, the label 2). The reason why we use such a combination of angles to represent base stacking is that the base plane (adenine, guanine, pyrimidine, uracil) is completely missing in a single-bead coarse-grained model as PlanarFold. Therefore, we devised a bp-step dependent base-stacking term, as the compensation for missing nucleotide details. We used 36 Turner's helix parameters (Table.S3) for 36 types of bp-step as the force constant ($k_{stacking}$) of our base-stacking term, units in kcal mol^{-1} $radian^{-2}$. Original Turner's stacking parameters are for free energy calculation and their units are kcal mol^{-1}. We added another factor (k_{stk}) to rescale the strength of base-stacking potential to match better with other terms of PlanarFold. Although direct incorporation of Turner's stacking parameters into PlanarFold's base-stacking energy is not most ideal, as these parameters indicate the combined energy contribution of base-pairing and base-stacking, we speculate the contribution of constituent base-stacking energy is correlated (if not proportional) to Turner's stacking parameters. Optimizing these 36 Turner parameters by genetic algorithm has proven tricky for the large 36-dimension parameter space as parameter sets do not converge. The base-stacking penalty/bonus ($p_{stacking}$) is -3.0 $radian^2$.

The stem-angle term is a harmonic angle term (Eq. 7) applied to three consecutive bases in the stem. Therefore, the stem-angle term is only applied to stems longer than 2 bp. This term is employed to help maintain the linear shape of the stem, by using a uniform equilibrium angle (θ_0) of 180 degree. The force constant (k_{angle}) and penalty/bonus (p_{angle}) are 4.0 kcal $mol^{-1}\cdot radian^{-2}$ and -3.7 kcal mol^{-1}, respectively. This term is sequence-independent, as a geometric constraint for stems.

Molecular dynamics simulation: general parameters

We adopted the MD framework similar to *AMBER*[2], including the file formats, control variable nomenclature, and basic MD pipeline (modified velocity Verlet integration, etc.). Berendsen thermostat is applied to maintain temperature, with no stochasticity introduced. All simulation uses a temperature coupling constant of 0.005 ps (i.e. 5 fs) to control the temperature fluctuation within reasonable range. No pressure control (barostat) is applied. A timestep of 4 fs is recommended and is used for all simulations in this study. Verlet neighbor list is used to accelerate the calculation of non-bonded interactions. Translation and rotational momentum of the system are removed every 1000 steps. All beads (nucleotides) use a same mass of 321.45 g·mol^{-1}.

Detailed parameters for all the simulations conducted in this paper can be found in the user manual on Github (*https://github.com/Lanxiang-thu/PlanarFold*) and Zenodo (https://zenodo.org/records/19397229).

MD simulations for predicting secondary structures

PlanarFold's potential energy has been optimized to distinguish high energy (unfolded and misfolded) states and low energy (native and near-native) states using genetic algorithm. Using the default force field parameters (Supplementary Table 5), the energy of the conformations can be directly used to predict secondary structure, as the conformation with the lowest potential energy should share the highest similarity with native state. The similarity with the native secondary structure is commonly indicated by F1-score, with an F1 of 1.0 being identical to native state. PlanarFold employs a sample-and-select strategy, where conformation pool of diverse secondary structures should be generated at first, rendering it much less efficient in predicting secondary structure compared to traditional methods and deep-learning (DL) based methods.

To generate such diverse conformation pool, both restrained sampling and unrestrained sampling are resorted. For the unrestrained sampling, 1000 cycles of Heating-and-Cooling (H&C, similar to simulated annealing) simulation to sample 1000 conformers for each RNA are performed. In each H&C cycle, there are 4 stages: 1). the temperature increases from 0 K to 800 K in 50,000 steps; 2). cooled from 800K to 300 K in 50,000 steps; 3). cooled more slowly from 300K to 0 K in 100,000 steps; 4). eventually maintained at 0 K for 50,000 steps. The related parameters are temp0, tempi, and duration. For each complete H&C cycle, 250,000 steps of simulation is performed. As 1000 H&C cycle is required, the total simulation step is set to 250,000 x 1000 = 250,000,000. The conformer at last frame (0K) of each H&C cycle is extracted, yielding a total number of 1,000 randomly sampled conformers from a 1-μs simulation for each RNA. For the restrained sampling, the RNAstructure's *Fold* module is resorted to generate vast number of diverse secondary structures in the first place. To ensure that both near-native and diverse far-native conformations can be sampled while considering the computational burden for subsequent MD simulations, the top (up to) 500 structures at 4 different window sizes are sampled. The RNAstructure can be downloaded at *https://rna.urmc.rochester.edu/RNAstructure.html*. For short RNAs, the conformations sampled under option "window=0" should cover all the conformations sampled under option "window=1, 2, or 3", since the conformation space of small RNA is comparatively small. However, for larger RNAs (> 200 nt), the options other than "window=1" are necessary for generating a diverse conformation pool. To accelerate restrained MD, we apply confinement (dpr=1) to avoid the time-consuming dynamic programming given only the restrained secondary structure is desired. To avoid the trans-isomer, the restrained MD is arranged into two stages. The

first stage is the “Pre-equilibrium Stage”, where only long-range restraints are applied. The second stage is the “Production Stage”, using the coordinates from the restart file generated at the end of the first stage as the initial conformation to run H&C cycle. In production stage, 5 cycles of H&C are performed, yielding 5 conformers under each restraint.

In total, 5N conformer will be generated in restrained sampling, where N is the number of non-redundant secondary structure sampled by RNAstructure. Along with 1000 conformer generated using unrestrained sampling, 1000+5N conformations will be generated in total. The conformation with the lowest potential energy will be selected as the predicted potential “native” secondary structure.

MD simulations for predicting free energy differences and kinetic rates

There are several short hairpins (T1-series and bistable RNAs, etc) benchmarked for thermodynamics and kinetics. For RNAs with fast conformation transitions (T1-series), 1 μs of simulation is sufficient to get statistically significant free energy difference of ground state and excited state. While for RNAs with fast conformation transitions (bistable RNAs), 100-1000 μs of simulation should be sufficient. The free energy difference between two states is calculated following the Boltzmann distribution: $\Delta\Delta G(B\text{->}A) = \Delta G(A) - \Delta G(B) = -8.3145 \times Temp \times \ln(pA/pB) / 4184$, where the Temp is the temperature in unit Kelvin; pA and pB are the population of state A and state B, respectively; the value 4184 is the conversion coefficient from kcal to Joule.

For instance, in a 1 μs simulation of T1 at 283 Kelvin, 100,000 conformations are recorded, among which 93821 frames and 5855 frames are GS and ES, respectively. The GS and ES occupy ~99.7% of all sampled conformers, suggesting such simulation is valid for calculation. The transition GS-to-ES and ES-to-GS both happened 1899. The free energy difference between GS and ES will be 1.56 kcal/mol.

The forward (backward) rates, defined as the rates from state A to B (state B to A), were determined by dividing the number of forward (backward) transitions by the dwell time at state A (state B). Hence, the forward rate (GS->ES) is 1899/(pGS × Simulation time) = 2017.5 μs^{-1}. And the backward rate (ES->GS) is 1899/(pES × Simulation time) = 32329 μs^{-1}. The exchange rate is calculated as: $k_{ex} = k_{forward} + k_{backward}$. The exchange rate is 34346.5 μs^{-1}, which is 5 $\times 10^4$ times higher than experimental measurement (453 s^{-1}). For some RNAs, if two conformation share the some identical secondary structures, those common secondary structure are added as restraints to facilitate the sampling to conformers of interests.

MD simulations for co-transcriptional folding

PlanarFold is equipped to simulate the co-transcriptional folding of RNA by progressively adding nascent residues along the transcribed RNA chains. Currently, PlanarFold only supports adding 1 nucleotide each time to avoid crashes caused by overlapping with transcribed residues. With PlanarFold’s drastically increased sampling efficiency, it could capture the conformation transitions ranging from microseconds to second, using the equivalent simulation time. The transcription speed can be modulated by adjusting the simulation time accordingly.

Given the stochastic nature of MD simulations, several parallel simulations (10 to 40 in this study for each RNA) are conducted to better predict the co-transcriptional folding pathway and end products. PlanarFold records a continuum of transcripts in diverse conformations across simulation time. Generally, the transcription is initiated from 9 nt (starting from 5’). A circular initial

conformation of 9 nt is generated according to the sequence, with initial velocity randomly assigned using clock time as random seed. The initial velocities assigned are small, and will quickly be adjusted to the velocities that give rise to set temperature in very few integration steps as we apply strong temperature coupling constant (tautp = 0.005 ps) in PlanarFold. After estimation of the transcription speed, the simulation time period can be determined and the RNA transcripts will elongate with same transcription speed to yield the full length transcript.

To better mimic the transcription in vitro, usually catalyzed by T7 polymerase, the simulation time must be carefully calculated to match with the experimental transcription speed. Generally, the transcription speed of T7 polymerase is 200-400 nt/s. Hence, the life span of each transcript is 2.5-5.0 ms. Employing equivalent time, the simulation time for each transcript should be set to 50-100 ns. Of note, the equivalent time has an error of one order of magnitude. Therefore, any simulation time within the range 5 ns to 1000 ns is acceptable (Supplementary Table 11). Simulations at these two extremes may be tested both, to determine whether variation of transcription speed within this range could affect the dynamics or other characteristics of interest.

MD simulations for mechanical unfolding and refolding

We implemented steered MD (SMD) simulation into PlanarFold to mimic the force-steered pulling and relaxing by optical tweezer (OT) or atomic force microscopy (AFM). In vivo, the mechanical unfolding also occurs under the regulation of cellular machineries. Similar to co-transcriptional folding pathways, the mechanical unfolding or refolding pathways are diverse and require parallel simulations. Each simulation trajectory can be deemed as a record of a single molecule event. Currently, we have achieved the constant speed (c.v.) mode and constant force (c.f.) mode of SMD. The passive mode of steered can be readily achieved by setting the initial distance and final distance as the same in c.v. mode.

In experiment, the RNAs are connected to handles, either DNA duplex or DNA/RNA hybrid duplex ranging from 2 kb to 10 kb, which is not yet compatible with PlanarFold model. Therefore, polyA handles are generally added to the 5' terminus and 3' terminus of the RNA of interest, to account for the dynamic effect of handle fluctuation in OT experiments. Poly G, U, and C can be used as well. However, G and U have a higher change of forming base pair (GC/UA and GU/UG) than A, and C can form CG base pair, which is stronger than AU. Therefore, poly-A handles are recommended, with a length of 200 nt (or 10 nt).

There are several critical variables in OT experiment: trap stiffness, pulling/relaxing speed, force region, and acquisition frequency.

1). The trap stiffness (often denoted by κ), in optical tweezer experiments, is a measure of the restoring force exerted by the optical trap on a trapped particle when it is displaced from the equilibrium position. In MD simulation, the trap stiffness essentially equals to the force constant of the virtual spring, which are both the spring constant of harmonic potential. The apparent stiffness in OT experiment is the modulation of trap stiffness and handle stiffness. The handle stiffness should be reflected by the handles themselves, which is hard to simulate yet. Hence, the apparent stiffness is still hard to reach by PlanarFold yet. More detailed and systematic simulation of OT experiment might be achieved in the future. PlanarFold adopts the same SMD strategy of *Amber*: $f_{steering} = 2 \cdot k_{spring} \cdot (X(t) - X_{RNA})$, where X_{RNA} is the distance between 5' end and 3' end of the RNA, and X(t) is the equilibrium value of the virtual spring. X(t) is linearly interpolated between the initial distance (*dist_init*) and final distance (*dist_final*) set by the user, to attain the desired pulling or relaxing speed. And, k_{spring} is the spring constant (*stretch_spring*) of the virtual pulling spring. The experimental trap stiffness we want to simulate is 0.046 $pN \cdot nm^{-1}$ for P5ab. Therefore, according to

equation: $2 \cdot k_{spring} = 0.046$ pN·nm^{-1}, k_{spring} is 0.023 pN·nm^{-1}. Since the variable "stretch_spring" is in unit kcal·mol^{-1}·Å^{-2}, and 1 kcal·mol^{-1}·Å^{-2} converts to 69.5 pN·Å^{-1}. The k_{spring} is set to 0.000033 kcal·mol^{-1}·Å^{-2}.

2). Loading speed or pulling/relaxing speed is the moving speed of optical trap devices. Owing to the enhanced sampling of PlanarFold, experimental loading speed can be comparatively closely reached. In OT experiments, the loading speed is usually in the range of 10 nm·s^{-1} to 400 nm·s^{-1}, which translates to simulation speed of 10 nm·μs^{-1} to 400 nm·μs^{-1} in PlanarFold, by employing equivalent time. The experiment loading speed is 200 nm·s^{-1} for P5ab, therefore, the simulation speed is 200 nm·μs^{-1} (20-fold of experiment loading speed even after converting to equivalent time). In PlanarFold, the simulation speed is determined by several parameters (see User manual), which should be determined by the force range of interest.

3). Force region is the window where conformation transitions (unzipping or rezipping, etc.) of interest happen. For p5ab, the experimental measured ripping region is around 13-16 pN, and the force-extension curves (FEC) exhibit force generally ranging from 5 pN to 25 pN. Therefore, we set the final distance (*dist_final*) and initial distance (*dist_init*) to certain value such that their corresponding force be around 25 pN and 5 pN, respectively.

In the final state of refolding SMD simulation, we assume p5ab has completely folded into native state. Since folded p5ab has 4-nt and 3-nt dangling loop at its 5' and 3' terminus, respectively, the final distance X_{RNA} should be the distance 8 inter-residue interval: 3 for 4 nt 5' dangling loop, 2 for 3 nt 3' dangling loop, and 2 for a terminal base pair (whose distance is nearly two inter-residue interval). In PlanarFold, the distance of inter-residue interval is 5.5 Å (the equilibrium value of *Bond* potential), so we have the equation: $f_{steering}(\text{final}) = 2 \cdot k_{spring} \cdot (X(\text{final}) - X_{RNA})$. Then, X(final) is 1131 Å. Similarly, in the initial state of refolding SMD simulation, we assume p5ab has completely unfolded into native state. Since p5ab is 56 nt, we have the equation: $f_{steering}(\text{initial}) = 2 \cdot k_{spring} \cdot (X(\text{initial}) - X_{RNA})$ and, X(initial) is 5737 Å. Then, we determine the simulation steps: loading speeding = (*dist_final* - *dist_init*) / (*nstlim**dt*). More technical setup of each parameter should follow the User manual.

4). The acquisition frequency (or sampling frequency) refers to the rate at which the position (or other properties) of a trapped particle is measured, typically in Hertz (Hz). This parameter is crucial because it determines the temporal resolution of the experiment and affects the accuracy of force and displacement measurements. MD simulation can achieve incredibly high acquisition frequency, by setting the variable "*dumpfreq*" as small as possible. In here, we set *dumpfreq = 5000*, which translates to an acquisition frequency of 50 kHz. Such acquisition frequency is 10 times higher than experiment acquisition frequency (generally 4-5 kHz). Such high simulation sampling frequency guarantees the more precise states visualized in FEC.

For unfolding SMD simulation at the same loading condition, we simply switch the value of the final distance (*dist_final*) and initial distance (*dist_init*). Besides, the initial conformation is the native state, instead of the unfolded state. The native state conformation is acquired by performing restrained MD at first using the native secondary structure as restraint. It is worth mentioning that, in our previous tests, partially reducing the charge ("*charge*") from 2.93 to 1.0 and decreasing the strength of base pairing ("*bphm_k*") from 0.4 to 0.3 can better reproduce the OT experimental measurements. Such parameter fine-tuning is based on the buffer condition difference. The parameters we originally refined is based on free energy differences measured in NMR buffer, which usually differs from OT buffer. The OT buffer for p5ab consists of 250–300 mM NaCl or KCl and 3–20 mM $MgCl_2$, while the NMR buffer generally contains 10 mM sodium phosphate and 0.01 mM EDTA.

Dynamic programming

Dynamic programming is indispensable for our single-bead coarse-grained MD (CGMD) model to unambiguously describe base-pairing and base-stacking interactions. The missing physical representation of the nitrogenous base plane in a single-bead model complicates the explicit definition of base-pairing and base-stacking interactions. Several multi-bead coarse-grained models define a distance and angle dependent base-pairing and stacking term using beads on the base. For a single-bead model, if a distance dependent base-pairing term is defined, then a bead will form pairing interactions with several beads, which would far exceed the realistic occurrence of multiplets (such as triplet and quadruplet). Therefore, multiplets are forbidden in PlanarFold. Consequently, we stipulate that one bead can form only one base pairing at most. Intuitively, under such a base pairing potential, two interacting beads will approach as closely as possible (before the vdW interaction becomes repulsive). We stipulate that each residue can only form one base pair with another residue, and each residue should pair with its closest residue, which results in a classic dynamic programming (DP) problem. By resorting to Nussinov's DP, we have circumvented the usage of complex empirical functional forms of base-pairing and base-stacking potential like other models employed. In PlanarFold, DP determines the secondary structure (containing only nested, canonical base pairs) at each step based on instantaneous coordinates, and applies the base-pairing and base-stacking (and stem-angle) potential to the residues accordingly (Fig.1a and Supplementary Fig.1).

There are 7 steps related to PlanarFold's DP algorithm:

1. **Calculate the contact map**. The value of the contact map (Cmap) is decided according to equation 12 and 13. For an RNA of M nt, a contact map of Cmap(M, M) is assigned during preparation.

2. **Select the stem blocks according to contact map**. Only the upper-triangular part of the map is used for this process given the symmetry of the contact map. We employed a recursive algorithm to search for the neighboring contact of each contact to determine the independent "block". For each contact in the contact map, this algorithm searches its neighboring valid contacts recursively. Therefore, each recursive search will identify a continuous block of contacts, which suggests an individual helix region. As indicated in Fig.1b, there are 4 blocks, hence 4 helix regions (R1-R4) will be identified by this recursive search. By splitting the contact map into sub-matrices, the computationally heavy enumeration of bifurcated helices ("branching") is also avoided in dynamic programming. Moreover, independent calculation of each region is also designed to facilitate future GPU parallelization.

3. **Perform dynamic programming at each stem block**. The summation matrix (the objective function) is constructed according to equation 11 and backtracking is executed to give out the set of base pairs from the trace that yields the best solution. After that, base pairs in each helix region are trimmed into stems (as one stem block might contain several discontinuous stems).

In equation 11, any two residues can form base pairs, contributing to the Cmap with corresponding weights. Since our DP algorithm deals with segmented helices where all residues within cutoff are calculated, both bulge loops and internal loops contribute to the objective function S(i,j). Rather than incorporating numerous (up to hundreds of) loop parameters from the Turner's thermodynamic parameter set, we introduced a simple parameter termed "asymmetry penalty (*bulge_p*)" to distinguish the different stability of symmetric and asymmetric internal loops and bulge loops. We chose a value of 0.02 for the asymmetry penalty (*bulge_p*) to balance the

thermodynamics of T1 and T4, as large asymmetry penalty favors the GS of T1 but disfavors the GS of T4, and vice versa. PlanarFold's sampling around minor conformation changes heavily relies on DP, such that small adjustments to the DP-related parameters can significantly alter the dynamic ensemble. For instance, changing the bulge penalty from 0.02 to 0.005 reduces ES population of T4 RNA from 0.3% to trace levels (< 0.01%).

4. **Exclude parallel stem, trans-isomer, and pseudoknotted base pairs**. First, occasionally formed "parallel" stems are excluded, as all most naturally-occurring RNA helices are "anti-parallel". Second, "trans-isomer" is a phenomenon that occurs when two stems are in two opposite planar topologies, which is an issue only existing in planar structures (analogous to cis-trans isoform of carbon-carbon double bond) but not in 3D space. We devised a vector-based method to eliminate trans-isomer and pseudoknotted base pairs simultaneously. Third, since our model is designed to study secondary structure and its related dynamics, pseudoknotted base pairs are also prohibited. Of note, in the contact map, the pattern of pseudoknotted (un-nested) base pairs is no different from the pattern of nested base pairs. While in planar topology, many long-range pseudoknotted stems cannot be easily formed, restricted by the persistence length of RNA chain (5.5 Å in PlanarFold). However, for some short pseudoknot RNAs, their un-nested base pairs can form in our planar representation (Supplementary Fig.20). To permit the formation of pseudoknotted base pairs, the procedure of PK exclusion should be ignored by setting the control parameter iPK=1.

5. **Exclude multiplets and lone pairs**. If a residue is close to two other residues, it might will be identified to form two base pairs. Multiplets are prohibited as their unrealistically high occurrence in PlanarFold. Therefore, for those residues that form two base pairs, we keep the one of its two base pairs that has a larger Cmap value. If a residue forms more than two base pairs, notice information will be printed in the energy output file. Lone pairs are single base pairs without neighboring base pairs (i.e., with no adjacent base pairs on either side of the sequence), which is also excluded.

6. **Assign base-pairing, base-stacking, and stem-angle terms according to the final secondary structure**. The non-canonical base pairs are only used to calculate S(i,j) but not used to draw the final secondary structure. Due to the lack of their experimentally derived stacking and pairing parameters, but also their complexity and unpredictability in internal and bulge loops. For instance, all symmetric N×N (e.g. 1x1, 2x2) and asymmetric N×M (N<M, e.g, 1×2, 3×4) internal loop will form N non-canonical base pair if non-canonical base pairing is considered in PlanarFold. This might be true for some short symmetric internal loops such as 1×1 and 2×2. Nonetheless, based on PDB statistics, many larger symmetric N×N (N>2) and asymmetric N×M (N<M) internal loops do not form N continuous base pairing as short internal loops do. Instead, residues in those larger internal loop form intricate base pairs and stacks networks, and some residues are unpaired or facing outward the helix. Hence, only AU, GC, and GU base pairs are allowed to calculated base-pairing, base-stacking, and stem-angle potential terms, to avoid the aforementioned complications caused by considering non-canonical base pairs.

7. **Calculate base-pairing, base-stacking, and stem-angle terms.** Based on canonical base pairs determined by DP, several harmonic terms (distance and angle) are selectively applied to corresponding residues to maintain the current secondary structure.

Supplementary figures

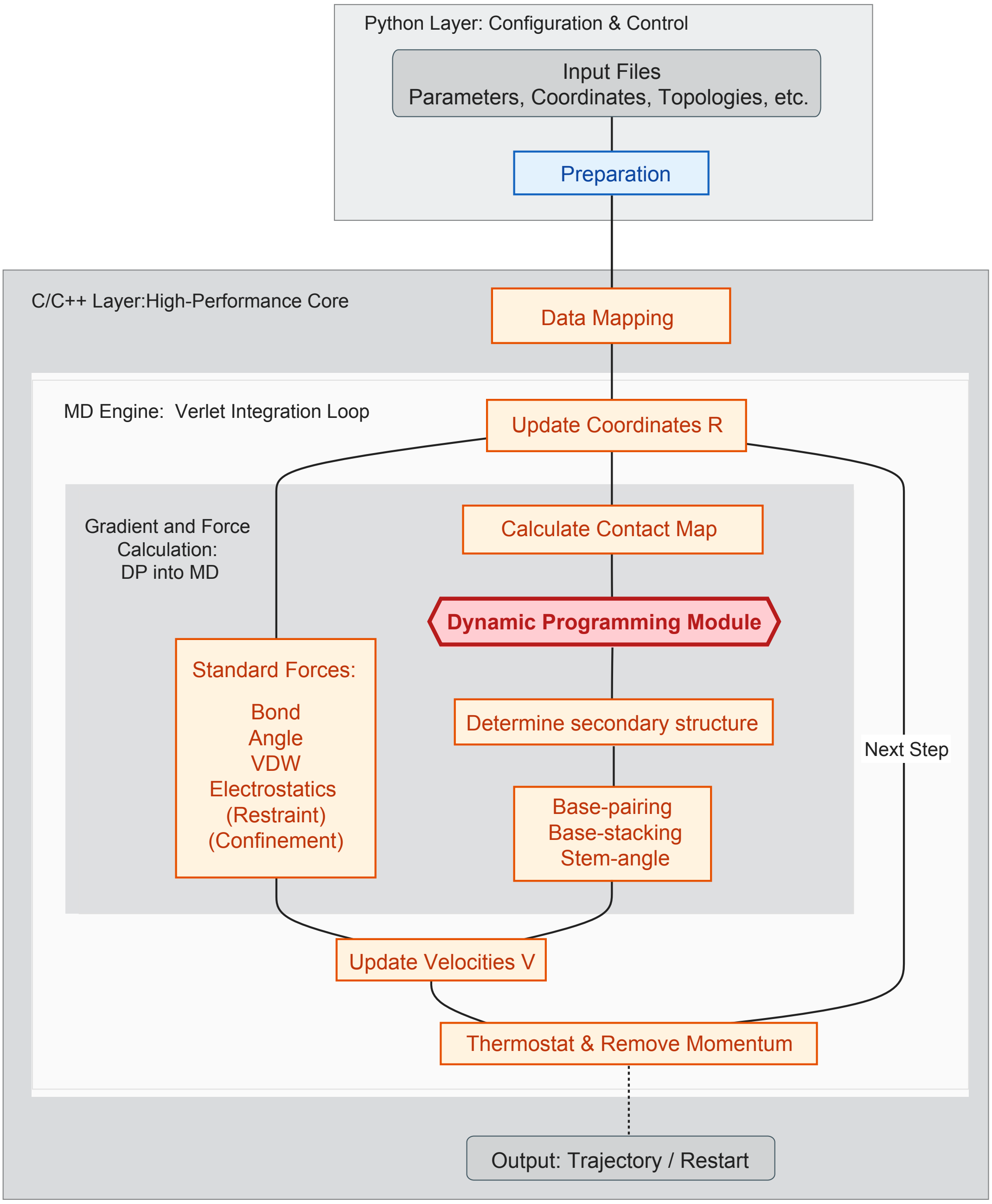

Supplementary Figure 1. The framework of PlanarFold: MD incorporated with DP.
The MD framework of PlanarFold follows AMBER's basic MD framework[2]. Different from conventional MD where potentials are applied to all residues, there are three potential terms (base-pairing, base-stacking, stem-angle) in PlanarFold only being applied to residues determined by DP to form stems. The DP determines the best secondary structure from the instantaneous coordinates at each timestep. PlanarFold does not require pre-defined secondary structure, underlying its capacity to investigate dynamic properties of various conformation transitions.

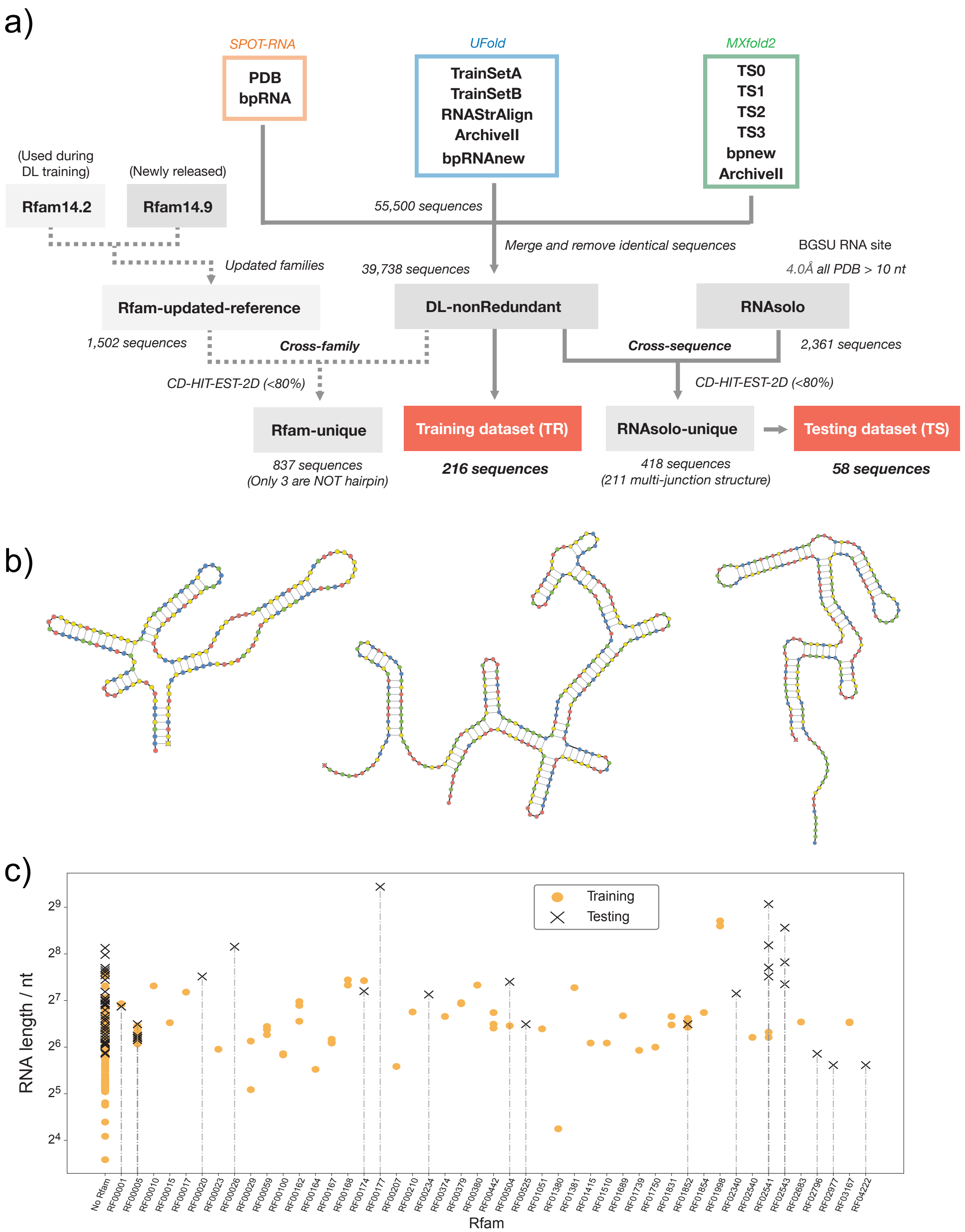

Supplementary Figure 2. Collecting training (TR) and testing (TS) datasets of PlanarFold. **a**. The pipeline of collecting the TR (216 RNAs) and TS (58 RNAs) datasets. The TR and TS datasets are obtained by filtering for RNAs with a high overall pairing ratio (>40%), a low unnested (pseudoknotted) pairing ratio (<10%), and diverse topologies. On the left, we originally tried to collect a cross-family dataset from Rfam14.9 following the pipeline of the previous study[3], but this attempt failed as the updated new families are predominantly simple microRNAs (834 out of 837

are short hairpins). On the right, we eventually collected a high-quality TR and TS dataset from PDB repository with manual re-check and annotation (Supplementary Table 1, and see *Methods*). Specifically, the TR dataset was curated from the PDB datasets used by SPOT-RNA, while the TS dataset was collected from *RNAsolo* repository. For comparison, the BPfold method utilizes the same datasets as SPOT-RNA and MXfold2. **b-c**. The diverse topologies and families covered by TR and TS. **b**. Three representative native structures with diverse topology (PDB ID from left to right: 3D0X [https://doi.org/10.2210/pdb3D0X/pdb], 8EA4 [https://doi.org/10.2210/pdb8EA4/pdb], 7DCO [https://doi.org/10.2210/pdb7DCO/pdb]). **c.** The families of TR and TS classified by Rfam.

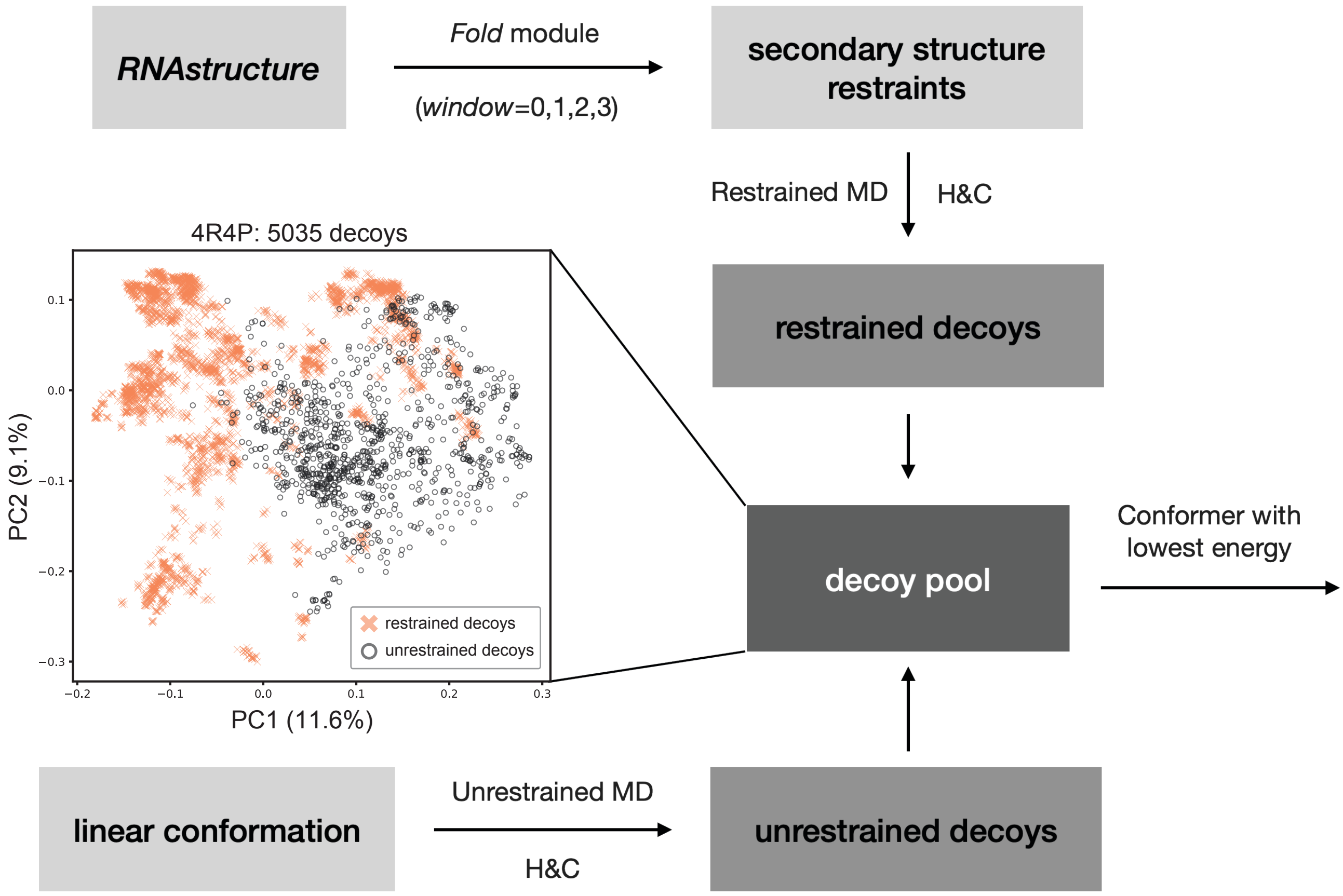

Supplementary Figure 3. Pipeline for predicting RNA secondary structure using PlanarFold. Secondary structures sampled using RNAstructure (*Fold* module) are imposed as restraints for PlanarFold simulation to generate a set of restrained decoys. Unrestrained MD simulations are also performed to sample more diverse conformations (especially the high energy states) to enrich the decoy pool. The decoy pool for a representative 186-nt RNA (PDB ID: 4R4P [https://doi.org/10.2210/pdb4R4P/pdb]) is displayed to justify the necessity of using both unrestrained decoys and restrained decoys for decoy pool. In the principal component analysis (PCA), the restrained and unrestrained decoys occupy distinct regions of conformational space, with only a small degree of overlap. For each RNA, the conformer with the lowest energy in its decoy pool is selected as the predicted native structure.

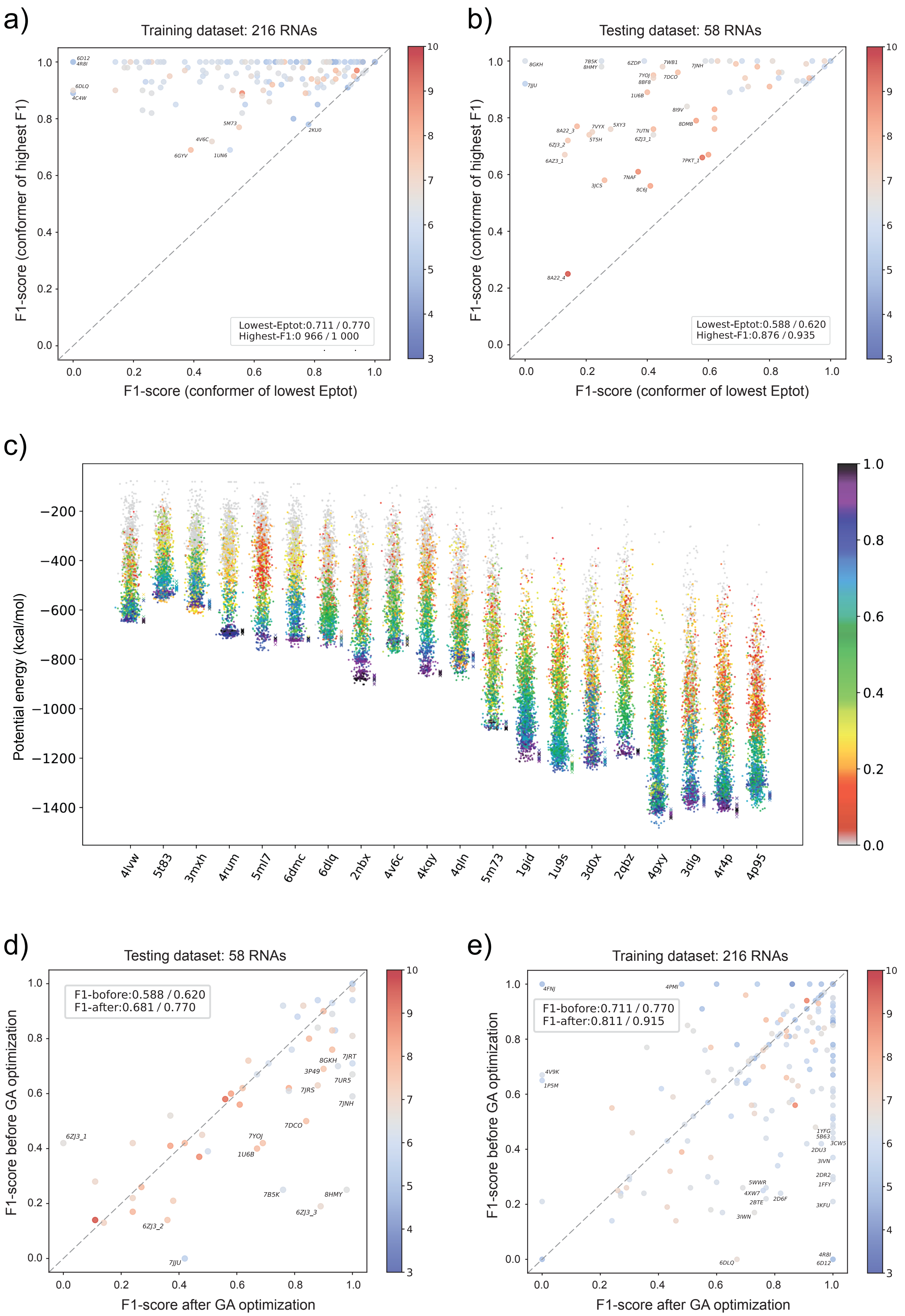
a)
Training dataset: 216 RNAs
F1-score (conformer of highest F1)
F1-score (conformer of lowest Eptot)
Lowest-Eptot:0.711 / 0.770
Highest-F1:0 966 / 1 000
b)
Testing dataset: 58 RNAs
F1-score (conformer of highest F1)
F1-score (conformer of lowest Eptot)
Lowest-Eptot:0.588 / 0.620
Highest-F1:0.876 / 0.935
c)
Potential energy (kcal/mol)
4lvw
5t83
3mxh
4rum
5ml7
6dmc
6dlq
2nbx
4v6c
4kqy
4qln
5m73
1gid
1u9s
3d0x
2qbz
4gxy
3dig
4r4p
4p95
d)
Testing dataset: 58 RNAs
F1-score before GA optimization
F1-score after GA optimization
F1-bofore:0.588 / 0.620
F1-after:0.681 / 0.770
e)
Training dataset: 216 RNAs
F1-score before GA optimization
F1-score after GA optimization
F1-bofore:0.711 / 0.770
F1-after:0.811 / 0.915

Supplementary Figure 4. Conformation pools sampled by PlanarFold for the TR and TS.
a. The sampled conformation pools contain native or near-native conformers. For each RNA in the TR (a) and TS (b) datasets, the scatter plots compare two metrics from its sampled conformational ensemble: the F1-score of the lowest-energy conformer (x-axis) and the highest F1-score among all conformers (y-axis). The two numbers in the legend indicate the average and median of F1-score. For example, in panel **a**, the conformation pool of 6D12 (at top-left corner) contains the native conformation, therefore the highest F1 score of its pool is 1.0. Nonetheless, the conformer with the lowest energy in the pool sampled for 6D12 shares no similarity with the native conformation. Consequently, its lowest-energy conformer has an F1-score of 0, placing 6D12 at the top-left corner of the plot (coordinates [0.0, 1.0]). Most RNAs sampled near-native conformers such that the median of highest F1-score reaches 1.0 and 0.935 for TR and TS respectively. Sampling performance on the TS set is lower than on the TR set, which is consistent with the TS set containing more large RNAs (the color bar indicates RNA length, in $\log_2$ scale). In panel **b**, a ribosomal RNA (PDB ID: 8A22_4 [https://doi.org/10.2210/pdb8A22/pdb]) only sampled the highest F1-score of 0.25, and it is the longest RNA in our dataset (695 nt). Adding the native conformation into its conformation pool will not change its prediction results, as its native secondary structure contains much less base-pairs and is less stable than its low-energy decoys. The reason why it adopts such “native” secondary structure (determined by cryo-EM) is that it was solved with ribosomal proteins in the ribosome complex, where RNA and protein interact intensively. This rationale may also explain the suboptimal sampling observed for several other large RNAs. Yet still, we followed the common collection criterion of RNA secondary structure evaluation studies, as filtering only orphan RNAs (without interaction with proteins, ligand, etc.) will largely reduce the already limited high-resolution RNA dataset. **c**. The conformation pool generated from restraint-free and restrained sampling. For each RNA, over thousands of conformers are generated for genetic algorithm optimization. Here, pools for several RNA are shown, ranked by RNA length from left to right. The color indicates F1-score of each conformer. The conformers include both near-native (purple and blue dots) and “far-native” states (gray and red dots). For comparison, the conformers restrained by native secondary structure are marked as crosses at the right of each column. In general, the lower potential energy (Eptot) a conformer has, the high similarity it shares with its native structure, showing good correlation with F1-score. For some RNAs, low potential energy decoys with low F1-score might exist (such as PDB ID: 3MXH [https://doi.org/10.2210/pdb3MXH/pdb]), and the genetic algorithm optimization is designed to address such cases by adjusting the energy function to favor near-native conformers over these low-energy, non-native decoys. **d-e**. The genetic algorithm optimization on TR and TS. The F1-score before and after GA optimization for each RNA on TS (**d**) and TR (**e**). The color bar indicates RNA length (in $\log_2$ scale). Most RNAs in TR and TS show improved F1-score, while several RNAs are sacrificed (with decreased F1-score, such as 6ZJ3_1 in TS and 4FNJ in TR. The average/median F1-score for TR improved from 0.711/0.770 to 0.811/0.915, and the average/median F1-score for TS improved from 0.588/0.620 to 0.681/0.770. The F1-score improvements on TR (0.100/0.145) and TS (0.093/0.150) are nearly the same, suggesting good generalization ability of the GA optimized parameters.

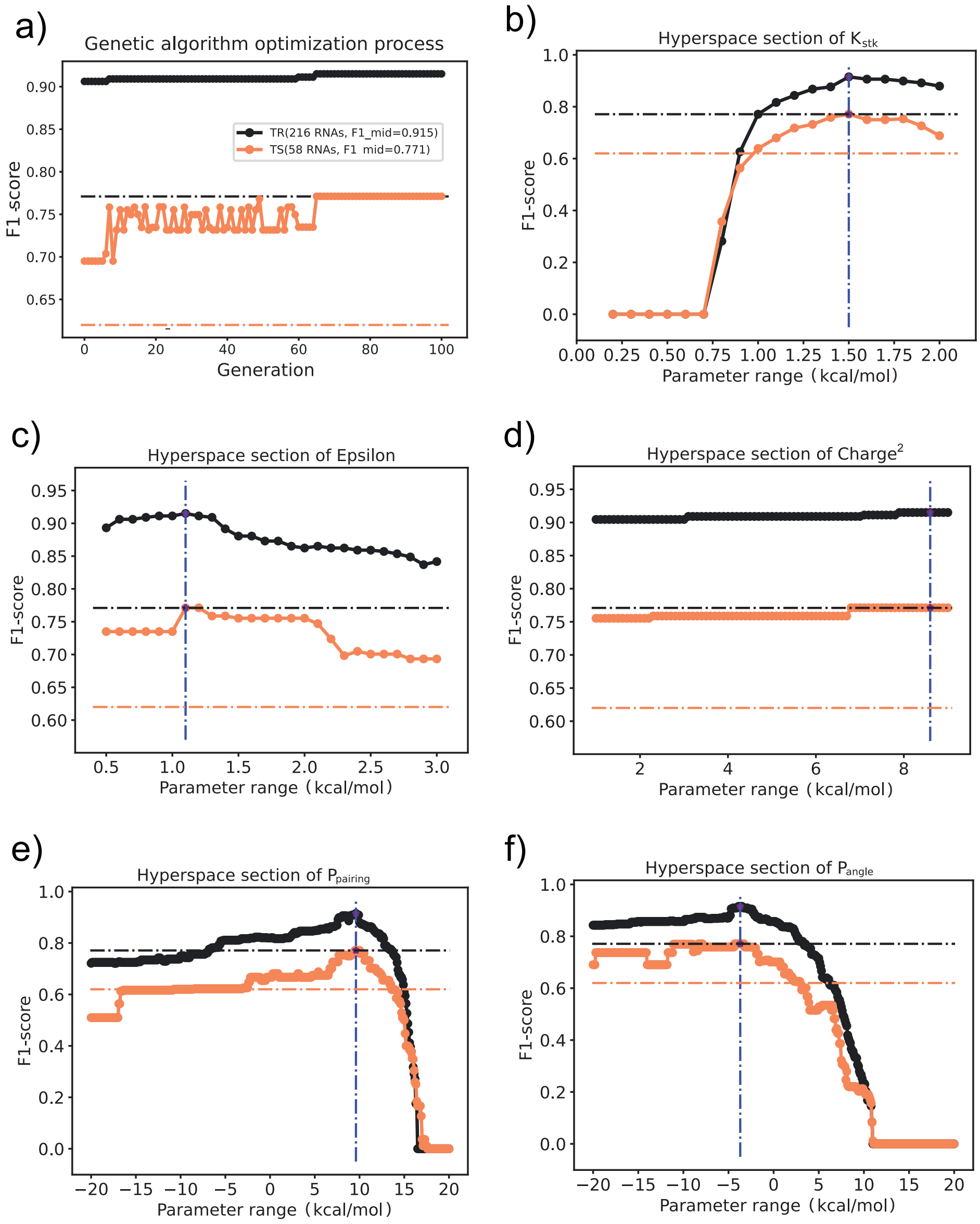

Supplementary Figure 5. Approaching local maximum after genetic algorithm optimization. **a**. The median F1-score for both TR and TS converges and remains stable after 100 generations of evolution. The parallel dashed lines indicate the median F1-scores before GA optimization for TR and TS respectively. We chose the final set of parameter and check the hyperspace section of the parameter k_{stk} (**b**), epsilon (**c**), charge^2 (**d**), $p_{pairing}$ (**e**), and p_{angle} (**f**). During GA optimization, each parameter can be chosen from values ranging from their lower bound to upper bound with a variation interval of 0.1. The vertical deep blue lines indicate the value of each parameter from the final parameter set: they are -3.7, 1.5, 1.1, 8.6, and 9.6 for k_{stk}, epsilon, charge^2, $p_{pairing}$, and p_{angle}, respectively. Each parameter resides at the peak within its one-dimensional projection, indicating that the final parameter set corresponds to at least a local maximum of the parameter hyperspace. We used *pygad* python package to perform GA optimization, with a population of 1000 for 1000 (or 100) generations of evolution. Parameter ranges were set for different reasons: the charge, epsilon, and k_{stk} are in a small positive range, as large values can destabilize the MD simulation. Meanwhile, $p_{pairing}$ and p_{angle} are penalties that affect scoring but not MD sampling and were therefore chosen from a large negative to positive range.

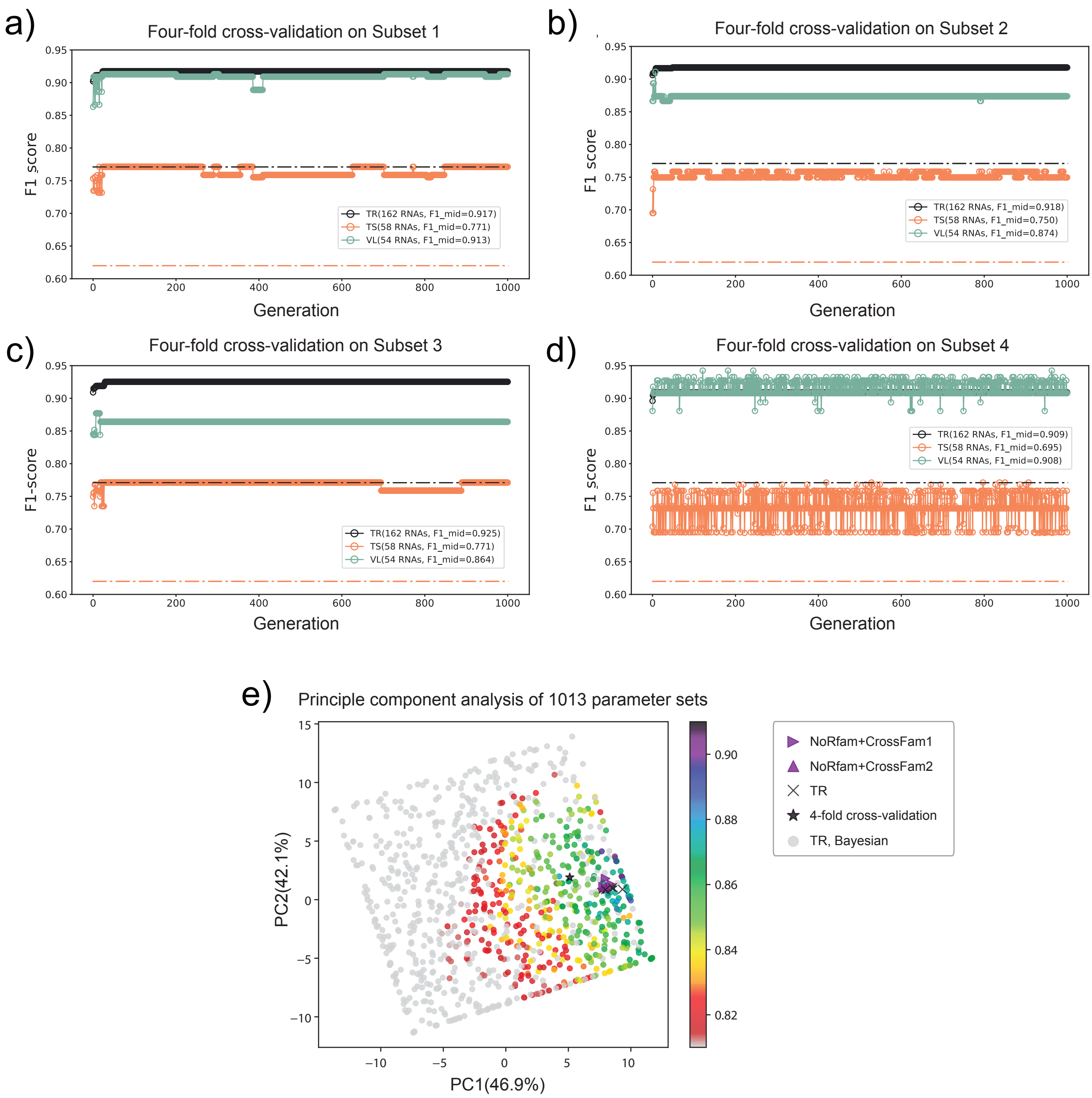

Supplementary Figure 6. Test of the robustness of GA optimization.

a-d. Four-fold cross-validation. For the 4-fold cross-validation, the 216 RNAs in TR were firstly arranged by length and then split into 4 subsets (54 RNAs each) to guarantee the similar length distribution of each subset. (**a-d**) The 1st, 2nd, 3rd, 4th subset was used as validation, and the other three were used as training. For subset 1-3, the training processes are stable (**a-c**), while the optimization for the fourth subset exhibited fluctuations (**d**), which can be explained by that the median of TS (58 RNAs) can vary as much as 0.07 under small changes of several RNAs (near median values). But the average F1-score is much more stable on subset 4 (data not shown, as the objective function here is the median instead of average F1-score). For those GA optimizations, we adopted a population number of 1000 evolving for 1000 generations. **e**. Principal component analysis (PCA) of parameter sets from Bayesian optimizations and GA optimizations. There are 1000 parameter sets from Bayesian optimization and 13 parameter sets from GA optimization, which are optimized on the identical parameter space (value range of each parameter). The color

bar indicates median F1-score of TR under each parameter set. In general, the data are spread out following the rainbow spectrum, suggesting the parameter hyperspace is smooth and harbors rare local maxima. For GA sets, 3 parameter sets are 3 independent parallel optimizations using all TR data for training (marked as crosses), while other 4 parameter sets are from 4-fold cross validation (marked as stars). Six parameter sets from cross-family validation are marked as triangles (the F1-scores are on CrossFam2 or CrossFam1 dataset, not on TS), either trained on NoRfam+CrossFam1 or NoRfam+CrossFam2. The parameter sets from genetic algorithm are the best sets at the end of optimization, while the parameter sets from Bayesian optimization are all the sets generated along the optimization process. The GA parameter sets converged except for one, which was from the cross validation using subset-4 as validation dataset (panel **d**). The 3 parameter sets trained on either NoRfam+CrossFam1 or NoRfam+CrossFam2 also converge with the parameter sets training on TR dataset, suggesting the robustness of GA optimization.

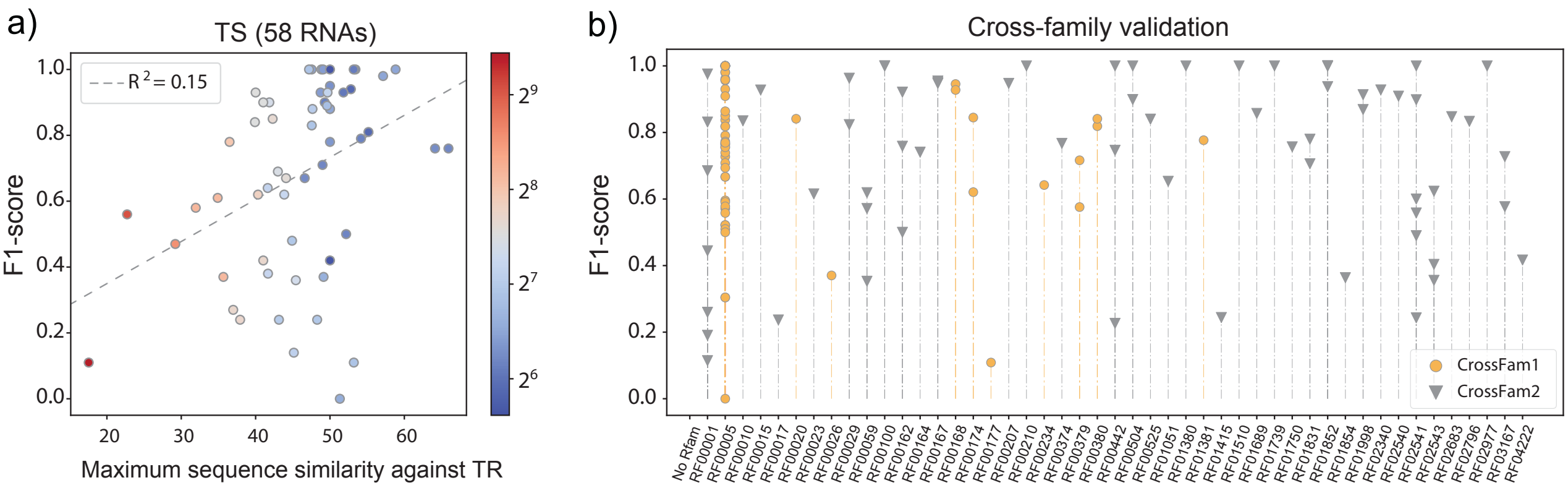

Supplementary Figure 7. Cross-family validation of secondary structure prediction.
a. The relationship between prediction performance and sequence similarity. The color bar indicates the RNA length. **b**. Rearrangement of RNAs from the existing TR+TS dataset and model retraining for cross-family validation. Based on Rfam family annotations (Supplementary Fig.2c), the 274 RNAs (TR+TS, 216+58) were rearranged into three datasets: NoRfam, CrossFam1, and CrossFam2. NoRfam contains 140 RNAs unclassifiable into existing Rfam families. The remaining 134 RNAs (belonging to 47 Rfam families) were split into CrossFam1 and CrossFam2, each comprising 67 RNAs, with no overlapping families between them. We then retrained the model using either NoRfam+CrossFam1 or NoRfam+CrossFam2 and tested it on CrossFam2 or CrossFam1, respectively. The resulting testing F1-scores for CrossFam1 and CrossFam2 reached 0.818 and 0.831, respectively. Deep learning-based models (e.g., SPOT-RNA, UFold) are not included in this cross-family comparison because many RNAs from CrossFam1 and CrossFam2 were part of their training data (Supplementary Fig.2c, orange dots).

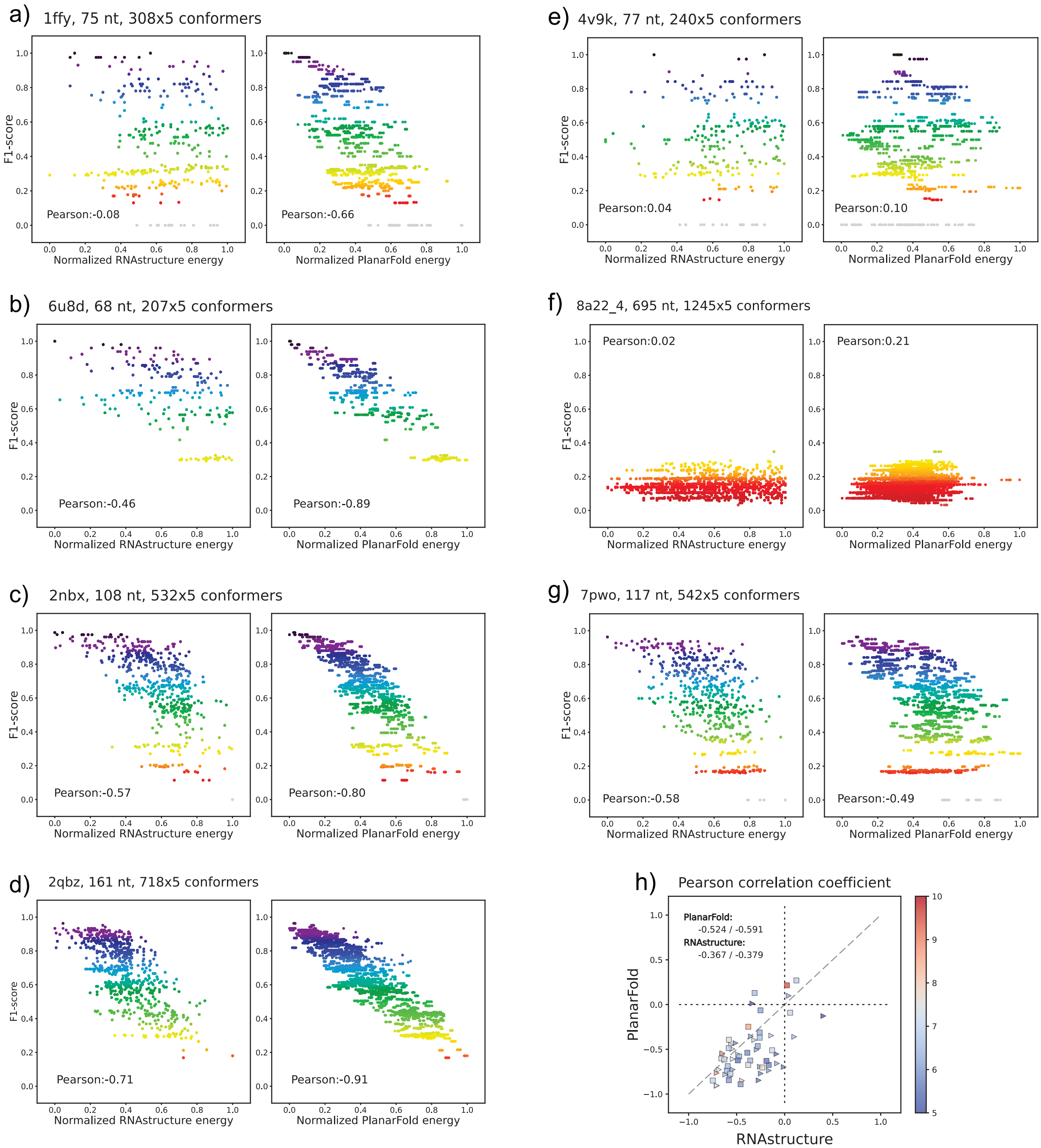

Supplementary Figure 8. Correlation between F1-score and energy from PlanarFold and RNAstructure.

Conformational pools for PlanarFold were generated using restraints from RNAstructure, with five conformers generated per restraint. Color and y-axis values both represent F1-score. The negative correlation indicates that lower energy corresponds to higher similarity with the native structure (higher F1-score). The parameter set of PlanarFold to generate those conformers is the GA optimized one. **a-c.** PlanarFold improved the correlation from no (Pearson < 0.3) or weak (0.3 < Pearson < 0.5) correlation to moderate (0.5 < Pearson < 0.8) or good (Pearson > 0.8) correlation. PlanarFold further improved the correlation from already fairly correlated scores (**c**). **e-f**. Both

PlanarFold and RNAstructure exhibited no correlation. **g**. PlanarFold energy shared slight worse correlation than RNAstructure energy. **h**. Distribution of Pearson correlation coefficients between energy and F1-score for PlanarFold and RNAstructure across all 58 RNAs. RNAs from the TR (28) and TS (30) sets are marked as triangles and squares, respectively. The average/median Pearson correlation efficient of PlanarFold and RNAstructure are -0.524/-0.591 and -0.367/-0.379 respectively.

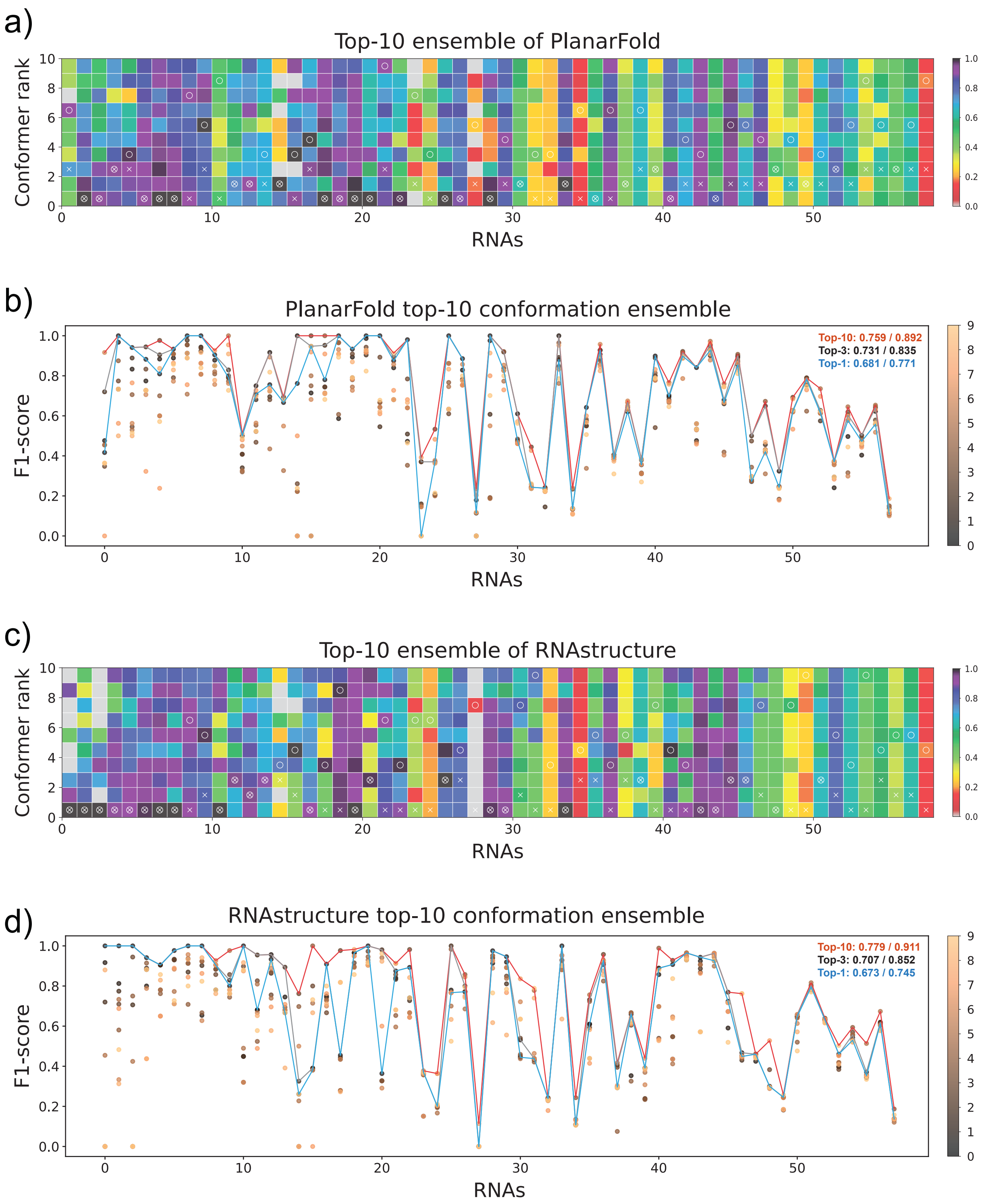

Supplementary Figure 9. Multiple prediction outputs of PlanarFold and RNAstructure. Both PlanarFold and RNAstructure can sample an ensemble of secondary structures, which may contain near-native conformations. **a**.**c**. The color bar indicates F1-score and the RNAs are ranked according to their length (from left to right), while the y-axis (conformer rank) indicates the top-10 conformers ranked by either PlanarFold energy (panel **a**) or RNAstructure energy (panel **c**) with conformer-1 being the energy minimum. The white circles and crosses indicate the conformers of

highest F1-score in top-3 and top-10 ensemble respectively. Longer RNAs tend to have lower F1-scores. **b**. The conformer of highest F1-score in top-3 and top-10 ensemble of PlanarFold elevates the average/median F1-score of TS dataset (58 RNAs) from the 0.681/0.771 of the single output (top-1) to 0.731/0.835 and 0.759/0.892 respectively. **d**. the conformer of highest F1-score in top-3 and top-10 ensemble of RNAstructure increases the average/median F1-score of TS dataset (58 RNAs) from the 0.673/0.745 of the single output (top-1) to 0.707/0.852 and 0.779/0.911 respectively.

a)

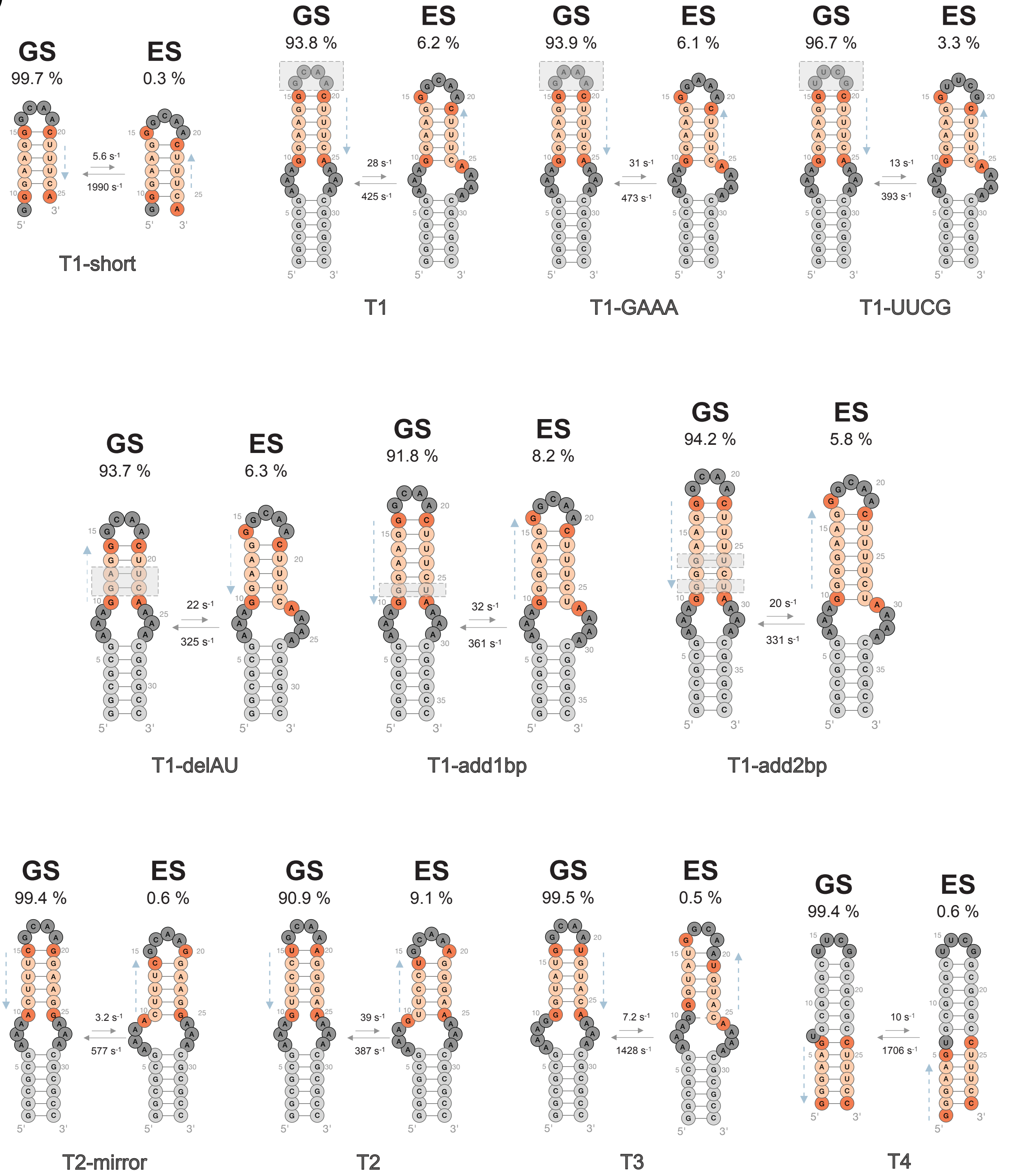
GS
99.7 %
ES
0.3 %
5.6 s-1
1990 s-1
T1-short
GS
93.8 %
ES
6.2 %
28 s-1
425 s-1
T1
GS
93.9 %
ES
6.1 %
31 s-1
473 s-1
T1-GAAA
GS
96.7 %
ES
3.3 %
13 s-1
393 s-1
T1-UUCG
GS
93.7 %
ES
6.3 %
22 s-1
325 s-1
T1-delAU
GS
91.8 %
ES
8.2 %
32 s-1
361 s-1
T1-add1bp
GS
94.2 %
ES
5.8 %
20 s-1
331 s-1
T1-add2bp
GS
99.4 %
ES
0.6 %
3.2 s-1
577 s-1
T2-mirror
GS
90.9 %
ES
9.1 %
39 s-1
387 s-1
T2
GS
99.5 %
ES
0.5 %
7.2 s-1
1428 s-1
T3
GS
99.4 %
ES
0.6 %
10 s-1
1706 s-1
T4

b)

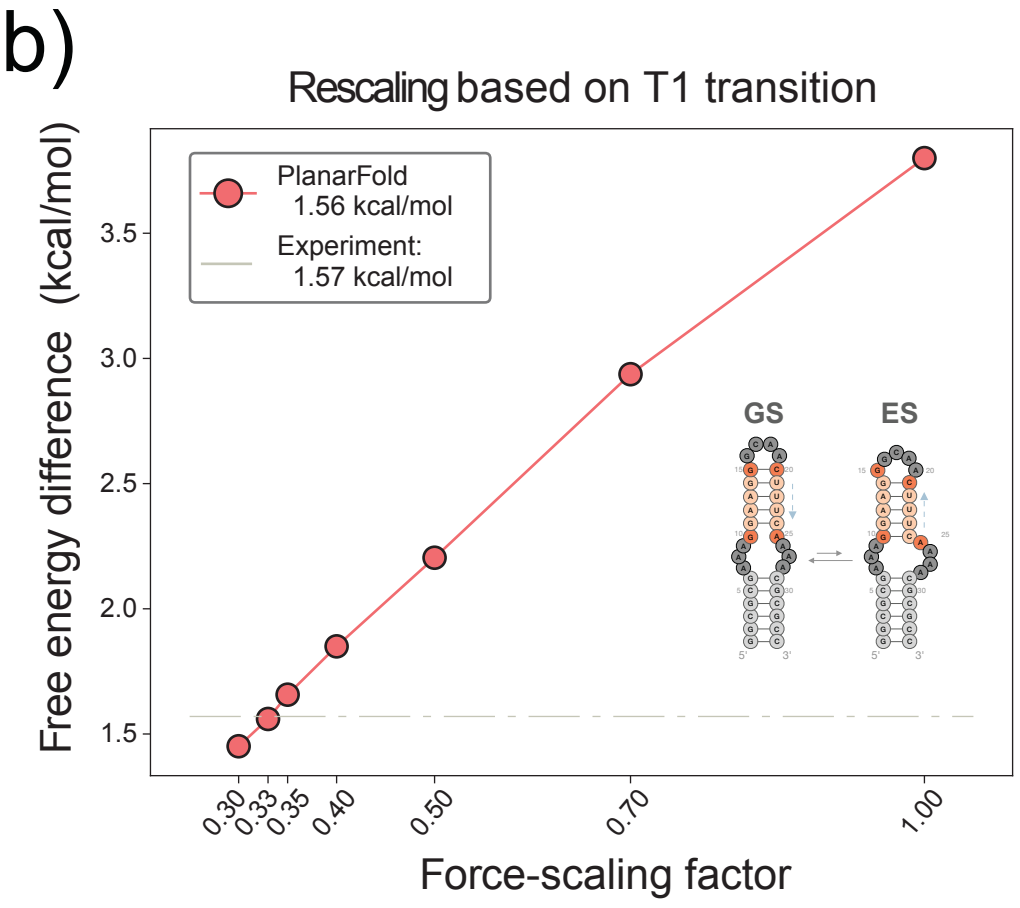
Rescaling based on T1 transition
PlanarFold
1.56 kcal/mol
Experiment:
1.57 kcal/mol
GS
ES
Free energy difference (kcal/mol)
Force-scaling factor
0.30
0.33
0.35
0.40
0.50
0.70
1.00

c)

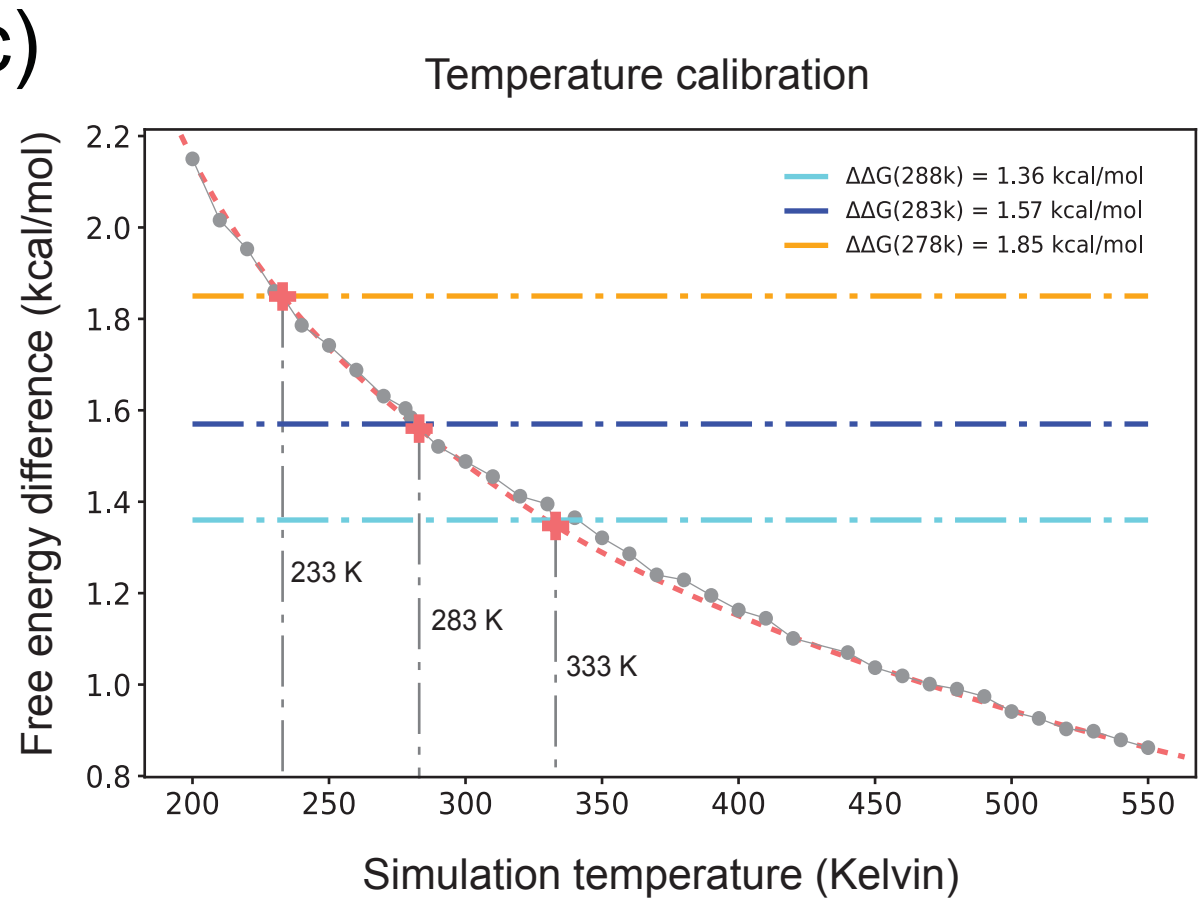
Temperature calibration
ΔΔG(288k) = 1.36 kcal/mol
ΔΔG(283k) = 1.57 kcal/mol
ΔΔG(278k) = 1.85 kcal/mol
233 K
283 K
333 K
Free energy difference (kcal/mol)
Simulation temperature (Kelvin)

Supplementary Figure 10. Thermodynamics benchmarking on short hairpins.

a. Structural characteristics of 11 hairpin RNAs exhibiting 1-nt or 2-nt register shifts. For each hairpin, the ground state (GS) and excited state (ES) are shown, with their experimentally determined populations and exchange rates labeled (data from previous study[4]). The internal loops in the ES of T1, T1-GAAA, T1-UUCG, T1-delAU, T1-add1bp, T1-add2bp, T2, T2-mirror are asymmetric (3x4), while the GS in these hairpins possess symmetric internal loop (3x3). For T3, both its GS and ES have asymmetric internal loop (5x4 and 3x4, respectively). For T4, its GS contains a 1-nt bulge loop, while its ES contains a 1x1 symmetric internal loop. For detailed descriptions, see the previous publication[2]. The bulge penalty was determined by balancing the thermodynamics of hairpin T1 and T4 with experiments, as the loop symmetry of their GS is opposite. **b**. Adjustment of absolute energy difference by tuning the force-scaling factor based on T1 (Supplementary Table 6). The original value of force-scaling factor is 1.0, indicating no re-scaling at all. As the force-scaling factor decreases, the population of ES increases and the free energy difference between GS and ES drops. Eventually, a value of 0.33 is chosen based on T1, which results in populations of 93% for its GS and 7% for its ES, closely matching their experimental measurements of 93.8% and 6.2%, respectively. **c**. Temperature calibration based on T1-GAAA (Supplementary Table 9). The temperature benchmark for 11 hairpins with register shifts was set at 283 K, corresponding to the NMR data collected at 10°C. To study thermodynamics and other characteristics at different temperatures, temperature calibration was performed by fitting the free energy differences of T1-GAAA measured at 278 K, 283 K, and 288 K. We conducted constant-temperature equilibrium simulations across a wide range of temperatures from 200 K to 550 K. The free energy differences simulated at 233 K, 283 K, and 333 K (marked as red crosses) closely match the experimental measurements at 278 K, 283 K, and 288 K (indicated by parallel lines), respectively. Consequently, a conversion relationship between PlanarFold's simulation temperatures and realistic temperatures can be established as: $T_{sim} = (T_{exp} - 283) \times 10 + 283$ (in Kelvin). For instance, to simulate an experiment performed at 288K, the simulation temperature in PlanarFold should be set to 333K, calculated as $T_{sim}(288K) = (288 - 283)\times 10 + 283 = 333$ (Kelvin).

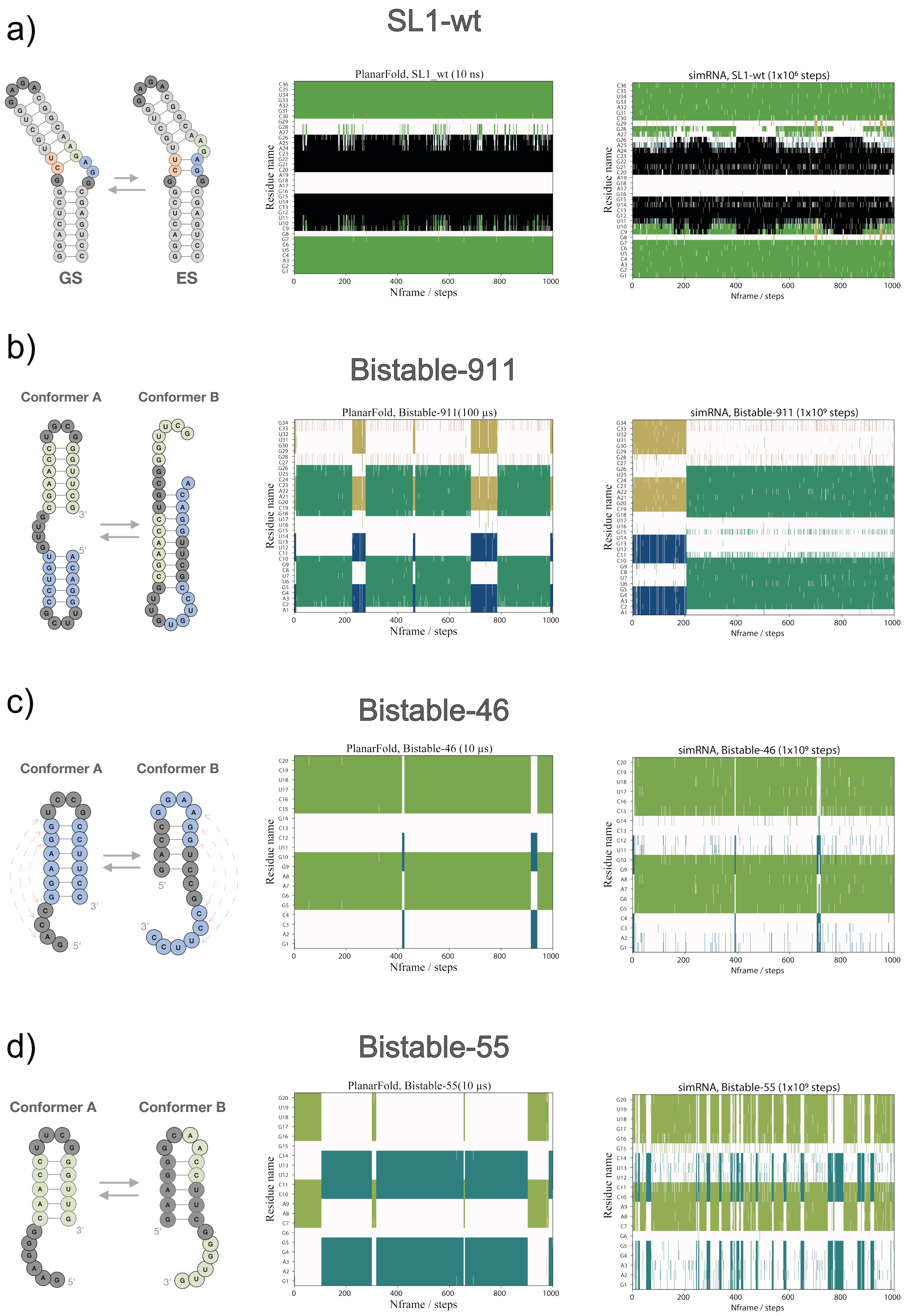

a)
SL1-wt
GS
ES
PlanarFold, SL1_wt (10 ns)
simRNA, SL1-wt (1x10⁶ steps)
Residue name
Nframe / steps
b)
Bistable-911
Conformer A
Conformer B
PlanarFold, Bistable-911(100 µs)
simRNA, Bistable-911 (1x10⁹ steps)
Residue name
Nframe / steps
c)
Bistable-46
Conformer A
Conformer B
PlanarFold, Bistable-46 (10 µs)
simRNA, Bistable-46 (1x10⁹ steps)
Residue name
Nframe / steps
d)
Bistable-55
Conformer A
Conformer B
PlanarFold, Bistable-55(10 µs)
simRNA, Bistable-55 (1x10⁹ steps)
Residue name
Nframe / steps

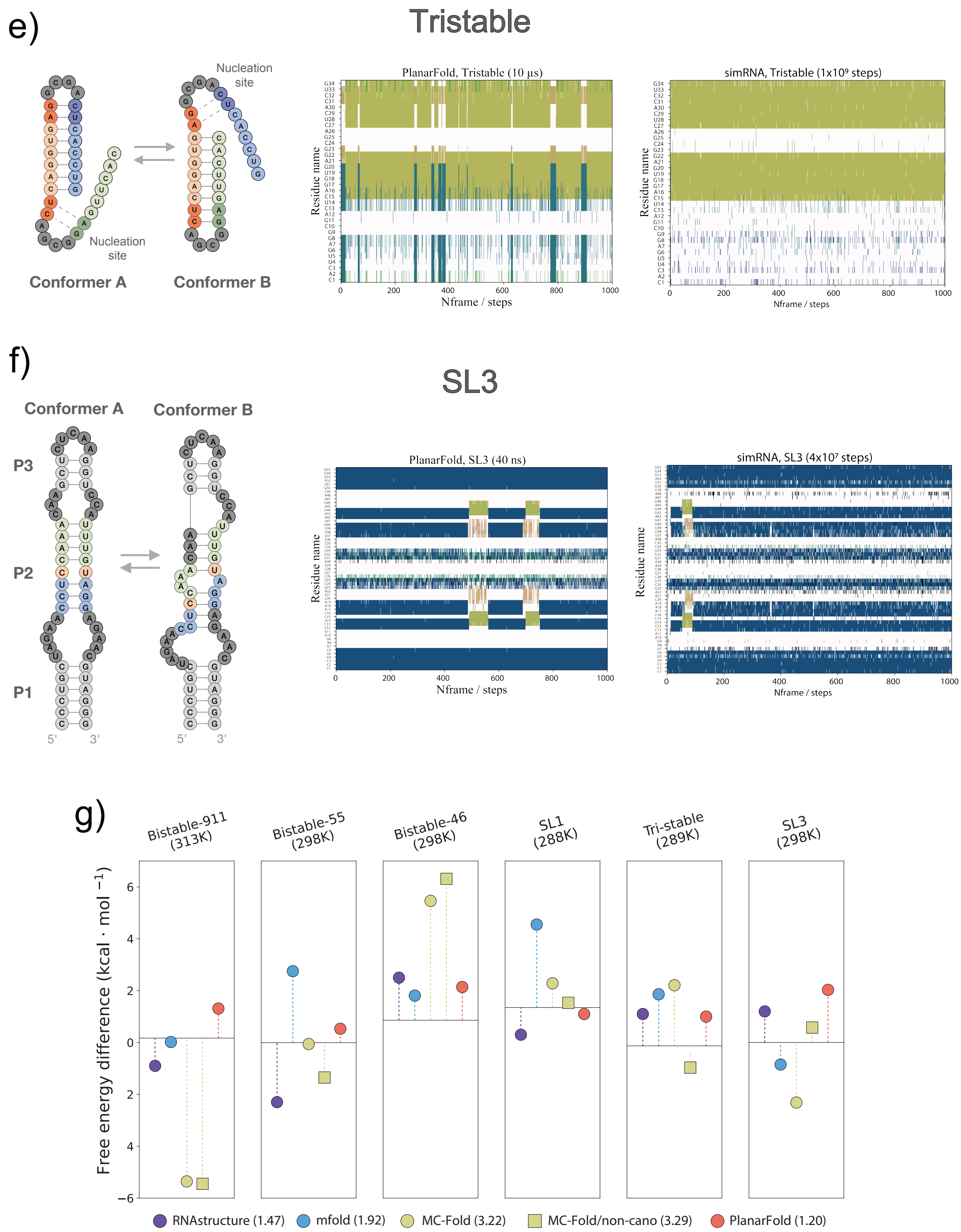

Supplementary Figure 11. Simulation results of transitions using PlanarFold and simRNA. **a-f**. For each RNA, the ground state and excited state are shown in the left column. The middle and right columns display the simulation results obtained using PlanarFold and simRNA, respectively. **f**. The P3 region of SL3 is predicted to undergo fast exchange, in addition to the slow dynamics of the 4-nt shift in the P2 region observed by others[5]. **g**. The performance of four software for predicting the free energy difference of transitions of diverse modes spanning 6 orders of magnitude (Supplementary Table 8). PlanarFold and RNAstructure perform best on those RNAs. When evaluated on a combined set that includes these RNAs and the 11 register-shift hairpins, PlanarFold performs best among the four tools. Of note, the free energy difference of those RNAs is more difficult to predict than that of 11 hairpins, leading to larger estimation variations.

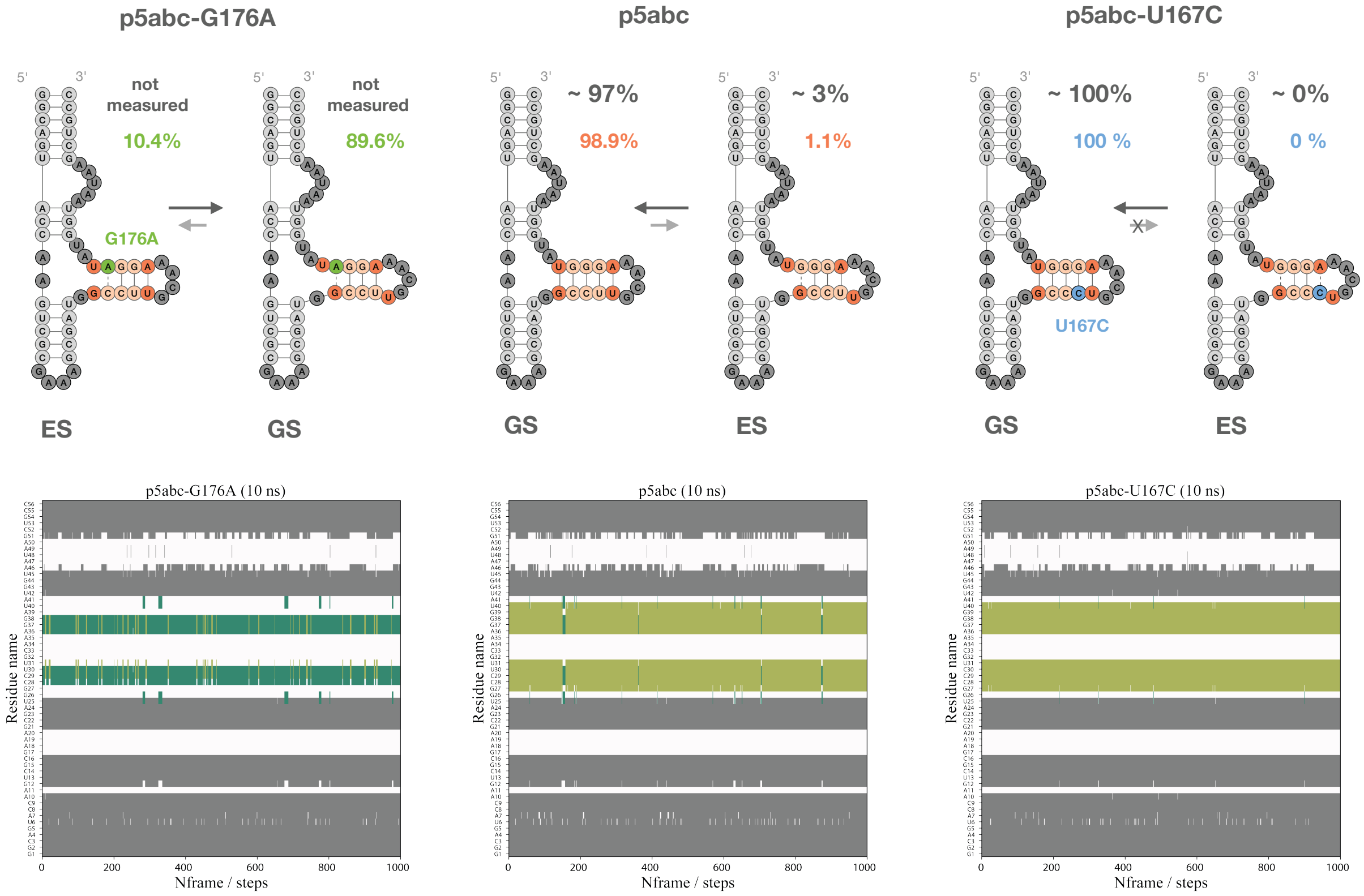

Supplementary Figure 12. Simulation results of three constructs of P5abc using PlanarFold. At the top, the ground states and excited states are indicated, along with their relative population measured by NMR (gray) and simulated by PlanarFold (colored). At the bottom, fragments of the 10-ns simulation trajectory for three constructs are shown, with the tetraloop and pentaloop conformation of p5c indicated as olive and green, respectively.

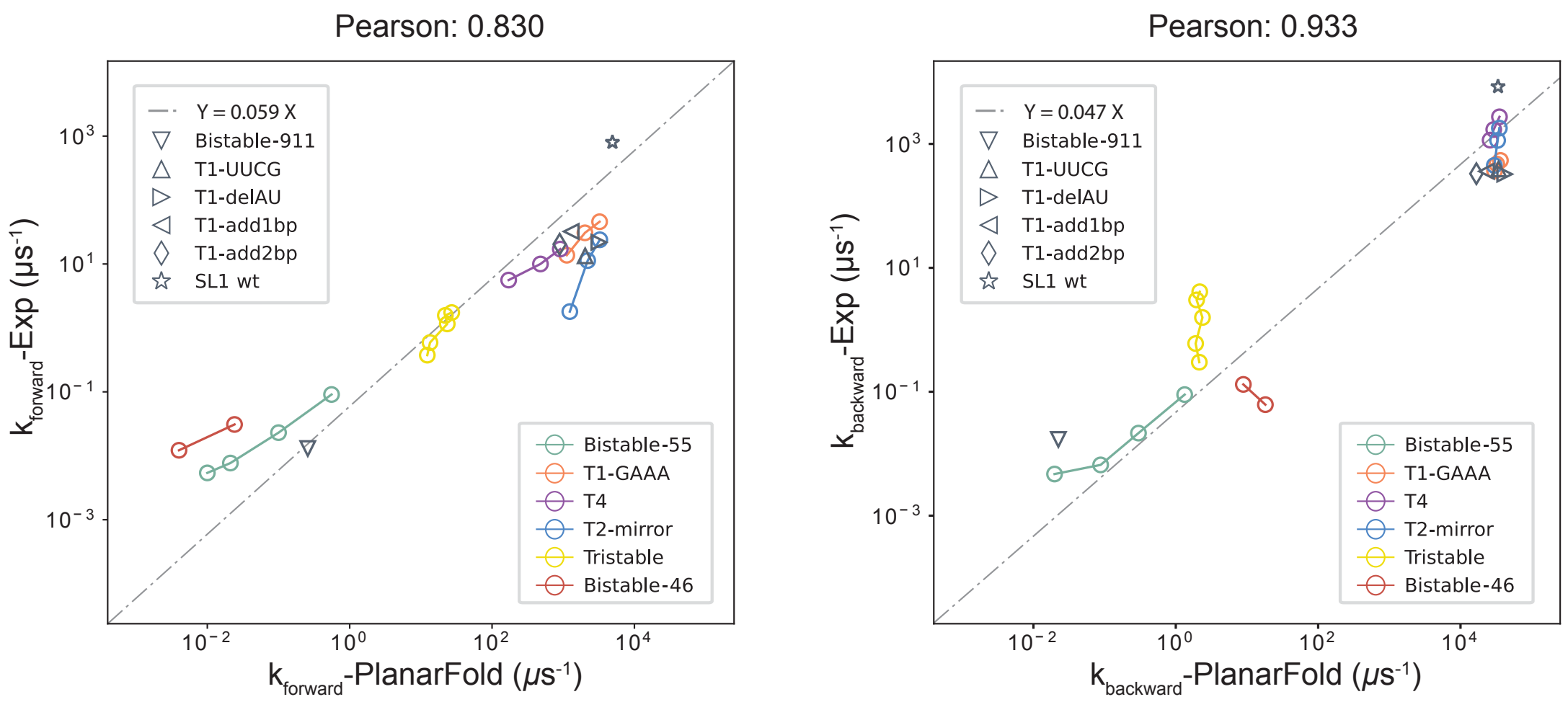

Supplementary Figure 13. The forward and backward rates simulated by PlanarFold. The correlation of the forward rates and backward rates with their corresponding experimental values (Supplementary Table 10). In general, both forward rates and backward rates agree well with NMR measurements (Pearson coefficient: 0.830 and 0.933, respectively). RNAs simulated at multiple temperatures are represented by circles connected by lines, and those at a single temperature by gray markers with different shapes.

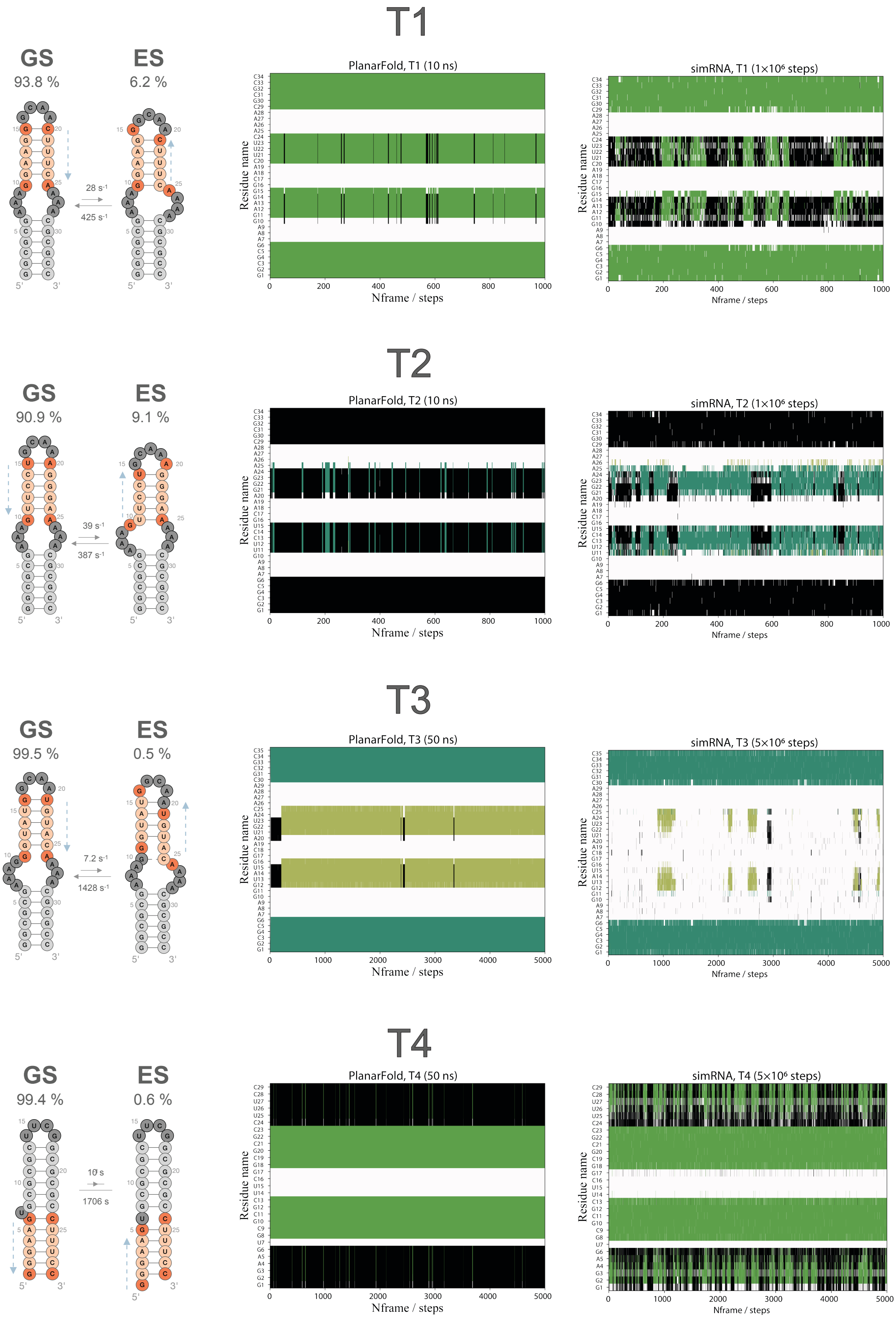

Supplementary Figure 14. Simulation results of transitions using PlanarFold and simRNA. In the left column, the ground state and excited state of each RNA are shown, with the positive and negative transition rates indicated (measured at 283K by NMR). In the middle and right column, the simulation results of PlanarFold and simRNA are shown, respectively. In contrast to PlanarFold, simRNA produced several discrepancies. For T1 and T4, the ES and GS were almost in 1:1 ratio. For T2, the ES was even more populated than its GS. For T3, neither its GS nor ES was stable, while PlanarFold could stabilize both states.

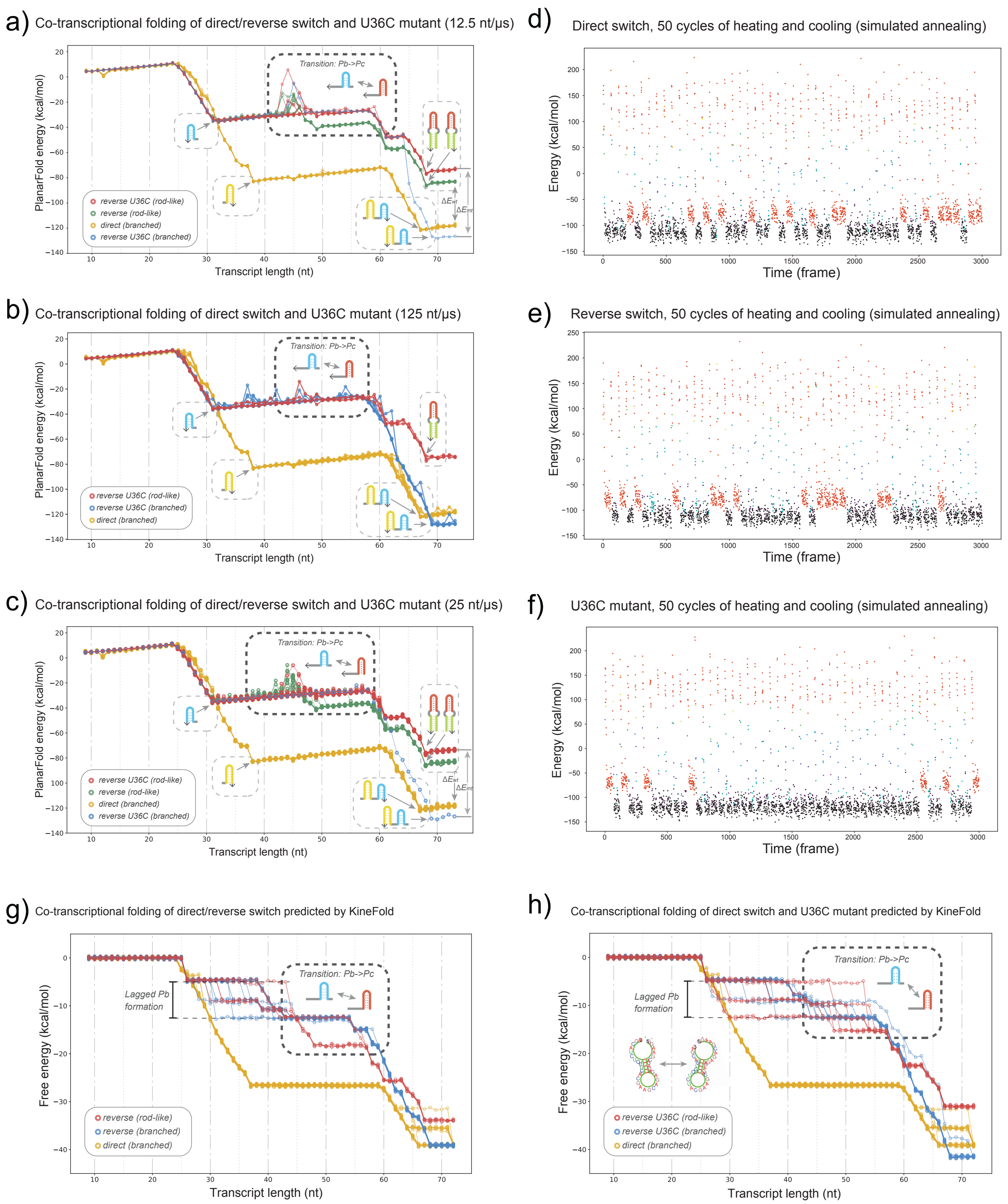

Supplementary Figure 15. Comparison of PlanarFold and KineFold.

a-c. Folding pathways of direct/reverse switch and U36C mutant under different simulation speed. **a**. Using the default equivalent time (20 μs in PlanarFold equals 1 s in reality), the experimental transcription speed of T7 polymerase (250 nt/s) translates to a simulation speed of 12.5 nt/μs. At this speed, the direct switch and reverse switch yielded divergent products: 100% branched conformers for the former and 100% rod-like conformers for the latter, both in good agreement with the experimental populations. The U36C mutant yielded 10% branched conformer and 90% rod-like conformer. Of note, the rod-like conformers for both reverse switch and U36C mutant are overestimated (100% and 90% in PlanarFold compared to 90% and 50% in experiment),

suggesting the simulated transcription speed might be slower than that in experiment, as it has been experimentally suggested that slower transcription speed might increase the population of rod-like conformer. Since the equivalent time has one order of magnitude of variation (Supplementary Table 10), we further investigated the simulation speeds to better match the experimental transcription speed as listed in Supplementary Table 11. **b**. At a simulation speed of 125 nt/μs (equivalent time ratio X=2), the direct switch, reverse switch and U36C mutant exhibited different folding pathways and end product during co-transcriptional folding. Specifically, 100% (40/40) of the direct switch and 70% (28/40 repeats) of the U36C mutant folded into branched conformer without helix replacement, while 30% (12/40) of the U36C mutant folded into rod-like conformer through Pb to Pc transition. The Pb-to-Pc transition window of the U36C mutant lacks the energy gradient between Pb-conformer and Pc-conformer, which was clearly observed in the reverse switch (Fig.4b), making U36C's Pb-to-Pc transition less favorable and producing fewer rod-like conformers compared to the reverse switch (30% versus 85%, at 125nt/μs). **c**. The co-transcriptional folding of 3 constructs at a lower transcription speed of 25 nt/μs (or 40 ns/nt, equivalent time ratio X=10). 100% (40/40) of the direct switch and 33% (13/40) of the U36C mutant folded into branched conformer, while 100% (40/40) of the reverse switch and 67% (27/40) of the U36C mutant folded into rod-like conformer. The Pb to Pc transition windows shifted forward, as a consequence of more sufficient time at lower transcription speed. Furthermore, the energy difference (ΔE_{mt}) of rod-like and branched conformer of U36C mutant is larger than the energy difference of the reverse/direct switch (ΔE_{wt}), where the energy of U36C branched conformer becomes lower and the energy of its rod-like conformer becomes higher, leading to the reduction of rod-like conformer population of U36C mutant during renaturing folding compared to experiment. **d-f**. The simulated annealing of three constructs. We performed 200 simulated annealing cycles for each construct to mimic the renaturing folding experiment, with 50 cycles shown for the direct switch (**d**), reverse switch (**e**), U36C mutant (**f**). The low-energy (-150 to -100 kcal mol^{-1}) black points indicate branched conformers, while the low-energy (-100 to -50 kcal mol^{-1}) red points indicate rod-like conformers. The branched-to-rod-like conformer ratios after 200 cycles are 121:79 for the direct switch, 141:59 for the reverse switch, and 182:18 for the U36C mutant. To ensure that RNA chains fold from a completely unfolding state at the beginning of each cycle, the temperature cycle was set as: 2000K, 600K, 533K, 600K, 2000K. The duration of each cycle is 120 ns. **g-h**. The co-transcriptional folding of three switches predicted by KineFold. **g**. 100%(20/20) of the direct switch and 30% (6/20 repeats) of the reverse switch folded into branched conformer without helix replacement, while 70%(14/20) of the reverse switch folded into rod-like conformer through Pb to Pc transition. Compared to PlanarFold, the folding pathways predicted by KineFold exhibited several unfavorable transitions (such as 2-nt register shift) within Pb, as Pb is comprised of tandem AU-GC repeats. Small random (Gaussian) variations were manually added to the free energy data to distinguish overlapping trajectories. **h**. 40% (8/20 repeats) of the U36C mutant folded into branched conformer, while 60% (12/20) of the reverse switch folded into rod-like conformer. Inset: KineFold predicted several energetically unfavorable transitions within Pb. Additionally, the native structure of Pb is challenging for KineFold to sample, resulting in lagged formation of Pb as indicated in panel **g-h**.

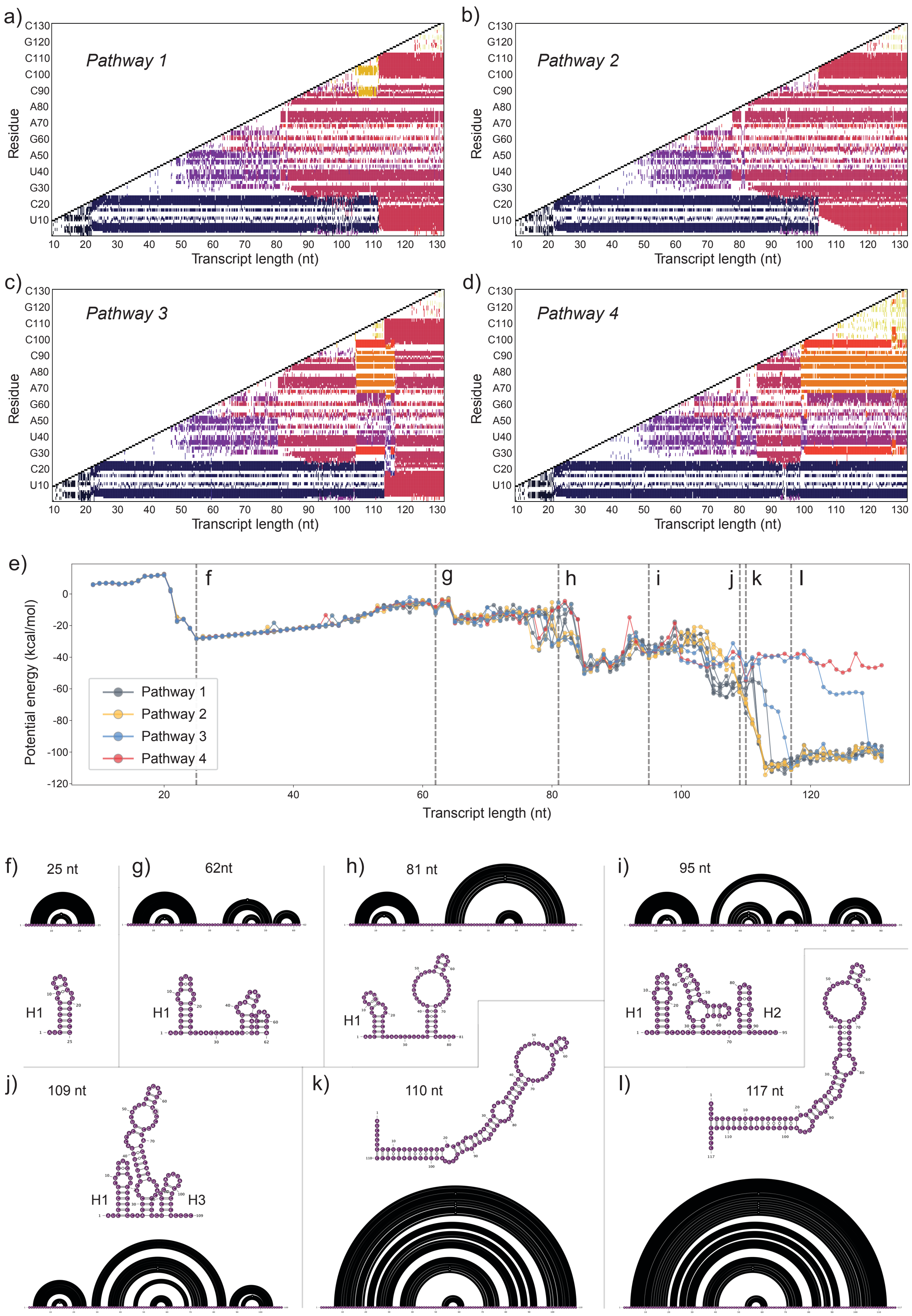
a)
Pathway 1
b)
Pathway 2
c)
Pathway 3
d)
Pathway 4
Residue
Transcript length (nt)
C130
G120
C110
C100
C90
A80
A70
G60
A50
U40
G30
C20
U10
e)
Potential energy (kcal/mol)
Transcript length (nt)
Pathway 1
Pathway 2
Pathway 3
Pathway 4
f
g
h
i
j
k
l
f)
25 nt
g)
62nt
h)
81 nt
i)
95 nt
H1
H2
j)
109 nt
H3
k)
110 nt
l)
117 nt

Supplementary Figure 16. Co-transcriptional folding of *E. coli* SRP using PlanarFold.
a-d. Evolution of secondary structures along four representative co-transcriptional folding pathways. The transcription rate of E. coli holo RNA polymerase is approximately 50–100 nt/s, equivalent to 10–20 ms per nucleotide. By adopting a transcription speed of 75 nt/s, each transcripts is elongated by 15 ms per nucleotide. Using the equivalent time (1 ms in reality corresponds to ~20 ns in PlanarFold simulation), the transcripts are elongated at a speed of 300 ns per nucleotide in PlanarFold. Four representative co-transcriptional folding pathways have been identified. In Pathway 1, the first intermediate hairpin (H1, panel **f**) forms in the beginning of the transcription (~ 20 nt), followed by the formation of part of the native base pairs around 80-90 nt, and then the third intermediate hairpin (H3) forms around 105 nt (panel **j**). After 110 nt, both H1 and H3 are gradually replaced with native base pairs. In pathway 2, H1 also forms and the native base pairs gradually forms and replaces H1 around 105 nt, without the appearance of H3. Pathway 1 and Pathway 2 correspond to the “Toehold” and “Early Toehold” pathway, respectively, suggested in previous work of R2D2. In both Pathway 3 and Pathway 4, the second intermediate hairpin (H2, panel **i**) forms and replaces the native base pairs around 100 nt. In Pathway 3, H2 is progressively replaced by native base pairs around 115 nt to form correct product. In Pathway 4, however, H2 persists till the end of transcription, giving rise to misfolded folding product. **e**. Potential energy profiles of transcripts during co-transcriptional folding. The folding pathways begin to diverge into 4 representative pathways around 100 nt. Across 12 replicate co-transcriptional folding simulations of SRP, Pathways 1, 2, 3, and 4 occurred 6, 3, 2, and 1 time(s), respectively. By approximately 110 nt, the intermediate hairpins H1 and H3 are gradually replaced by native base pairs, driving the system toward the low-energy native state. **f-l**. Representative secondary structures of seven transcription intermediates, analyzed as in prior studies. It is worth mentioning that, in the R2D2 method, the transcription is halted after 30 seconds and the mixture of transcripts of varying lengths is treated with the SHAPE reagent BzCN, which has a reaction half-life of 250 ms. Those treatments may cause structure rearrangement towards the lower energy conformation at each transcript length. However, our MD simulations capture the end products immediately after transcription, without re-equilibrium caused by subsequent treatment. Although PlanarFold is currently unable to derive a consensus conformation due to the divergence among folding pathways—a consequence of the experimentally inaccessible 30-second transcription halt (see R2D2 methods)—it successfully recapitulates all key intermediates and intermediate hairpins (H1, H2, and H3) detected by the R2D2 method. Diverse strand replacements are also unambiguously depicted in panel **a-d**. Notably, such agreement is achieved without utilizing any experimental data, such as SHAPE reactivities employed by R2D2. In contrast, KineFold failed to identify these features.

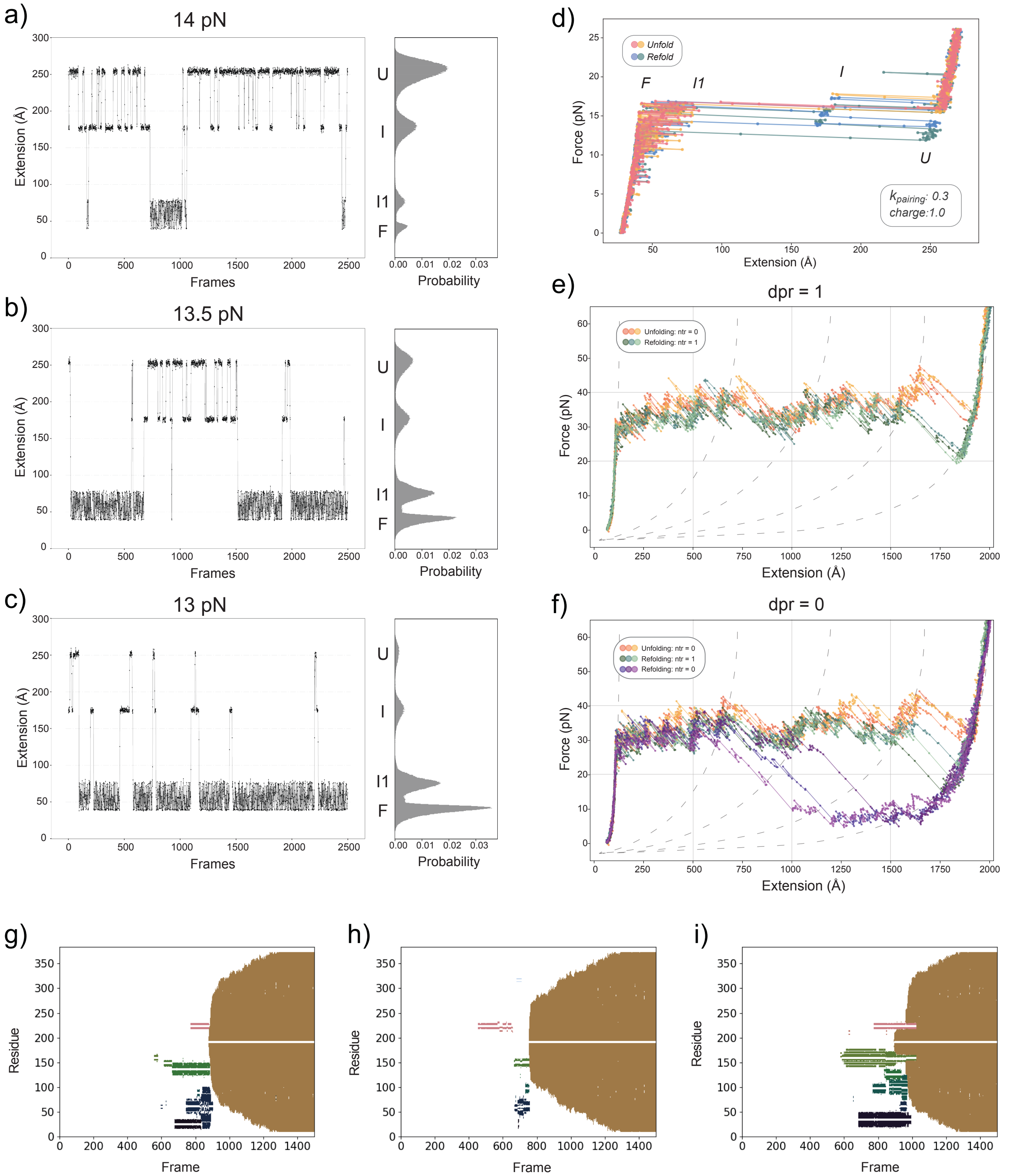

Supplementary Figure 17. Benchmark on P5ab and a 180-bp hairpin.

a-d. The hopping of P5ab in constant velocity (c.v., **d**) mode and in constant force (c.f., **a-c**) mode. The P5ab was kept at a constant force of 14 pN (**a**), 13.5 pN (**b**), and 13 pN (**c**). Extension-time curves and distributions of extensions are shown on the left and right, with an average bandwidth of 100 and 1 extension data, respectively. The fluctuation becomes weaker as the extension becomes smaller, because the fluctuating free loops becomes shorter, giving rise to narrower distributions at short extensions. The constant force mode of PlanarFold has not yet introduced the

variation of the feedback force, as the deviation is uncertain. **d**. The hopping of the entire P5ab hairpin was observed using the fine-tuned parameter, after removal of the 200-nt poly-A handles. The unfolded, folded, and intermediate states are labelled as U, F, and I and I1, respectively. **e-i**. Refolding and unfolding 180-bp hairpin at different simulation conditions. Force-extension curves of 180-bp hairpin (with 10-nt poly(A) handles) simulated at condition with (**e**) or without (**f**) native secondary structure for confinement. The confined MD (by setting: dpr = 1) accelerated the simulation by ~5.7 times for this construct. By comparing the orange and green curves in panel **e** and **f**, the confinement (dpr=1) nearly did not affect the unfolding process at all. By comparing the green refolding curves in panel **e** and **f**, the confinement did not dramatically affect the refolding process either, except for the nucleation process. For the refolding process, an extra restraint was applied to the native apical tetraloop (UUUU) to facilitate nucleation (green curves). Removing the confinement (by setting dpr=0) slightly affected the refolding process to yield less concurrent nucleation. Further removing the nucleation restraint (by setting ntr=0), the refolding was significantly lagged for confinement-free MD (panel **f**, purple curves). Without the confinement, misfolded hairpins (g-i) can form and compete with the native hairpin. **g-i.** The refolding process of 180-bp hairpin without any restraints or confinements. In the beginning, misfolded short hairpins, generally longer than 3 base pairs, were pervasive, which were subsequently replaced by the native 180-bp hairpin gradually. Most of those misfolded hairpins contain high proportion of GU base pairs (conformers not shown), which might be less likely to form, suggesting that the base pairing interaction for GU pairs in the model may be too strong.

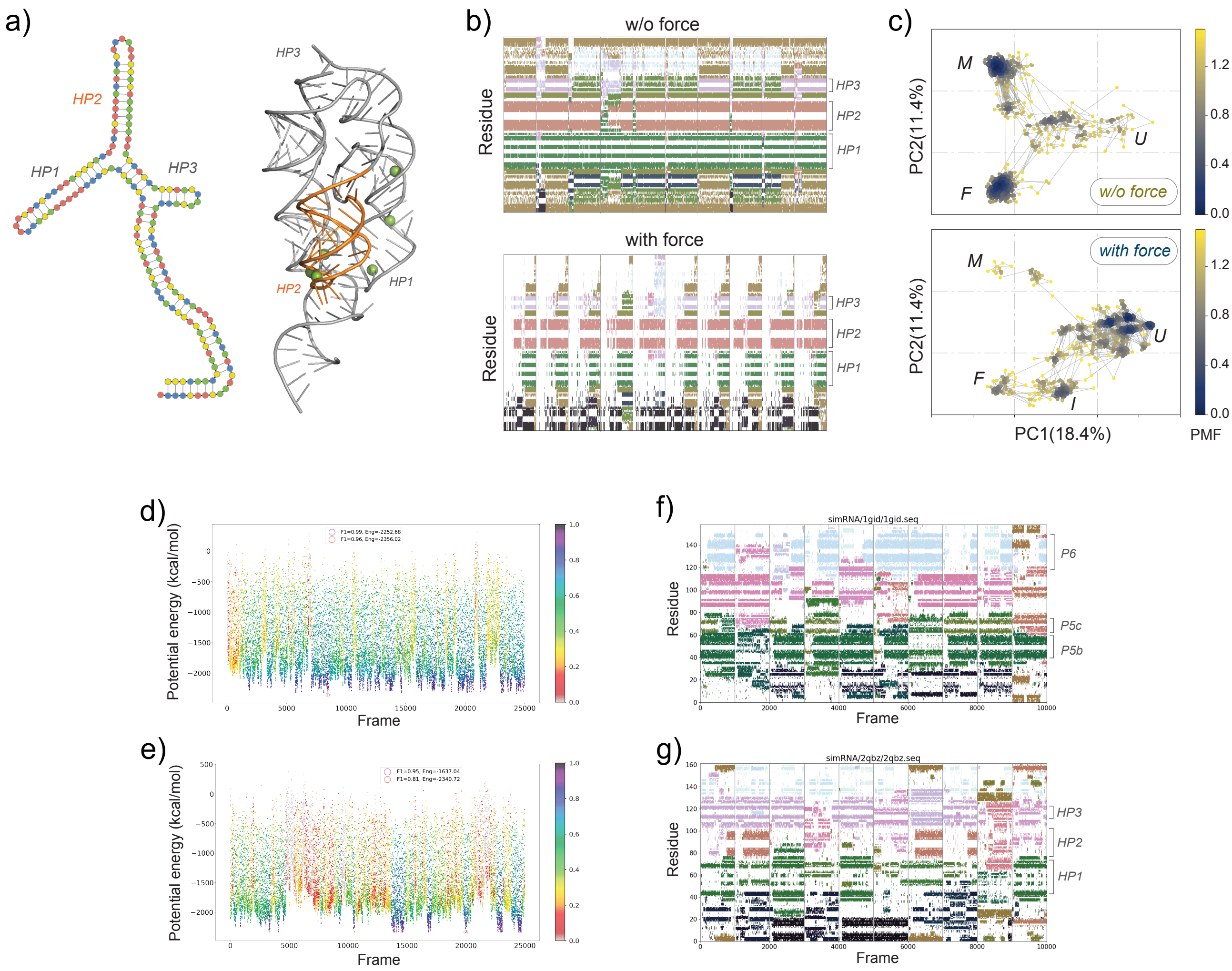

Supplementary Figure 18. Benchmarking *de novo* folding on two RNAs using PlanarFold and simRNA.

a-c. PlanarFold achieved successful *de novo* folding of large complex RNAs. **a**. The secondary and tertiary structure of a Mg^{2+}-sensing riboswitch aptamer domain M-box (PDB ID: 2QBZ [https://doi.org/10.2210/pdb2QBZ/pdb]). Magnesium ions are labeled as green spheres. Hairpin 3 (HP3) is absent from the deposited structure and was manually annotated according to the original paper. **b**. Ten repeats of M-box *de novo* folding were simulated under conditions with and without external stretching force. Repeat trajectories are separated by vertical gray lines. On the top, 5 out of 10 trajectories fell into kinetic traps. On the bottom, by gradually releasing the terminal tension, 8 out of 10 trajectories under steering force yielded native products (F). **c**. The energy landscape of folding the M-box domain with and without external force stretching. With stretching force, the misfolded pathways to state M were prohibited by 80% (4/5), and the folding pathway towards the native state changed dramatically, with the emergence of a frequently encountered intermediate state (I) and an intermediate hairpin at 5' end (**b**, bottom). Potential mean force (PMF) is in unit kcal mol^{-1}. **d-e**. The gradual annealing of two complex RNAs into near-native structure by rounds of heating and cooling: Group-I intron ribozyme domain, P4-P6 (**d**); Mg^{2+}-sensing riboswitch aptamer domain, M-box (**e**). These two RNAs can fold into near-native structure without external intervention, simply by heating and cooling, suggesting their native secondary structure might

easily form by nature. **f-g**. Simulation results of two complex RNAs using simRNA. The unrestrained *de novo* folding simulations were performed 10 times for each RNA with 1×10^7 Monte Carlo steps. **f**. Group-I intron ribozyme domain (P4-P6). The F1-score of the lowest-energy conformer is 0.469, at frame 8836. **g**. Mg^{2+}-sensing riboswitch aptamer domain (M-box). The F1-score of the lowest-energy conformer is 0.237, at frame 8695. Longer simulations (1×10^8 Monte Carlo steps) yielded similar results, suggesting sampling the near-native secondary structure for large RNAs without restraints is a challenge for simRNA. In comparison, PlanarFold sampled the native structure for both P4-P6 (F1-score = 1.00) and M-box (F1-score = 0.99), and the F1-score of the lowest-energy conformer sampled by PlanarFold is 0.90 and 0.88 for P4-P6 and M-box, respectively (**b** and Fig.6b).

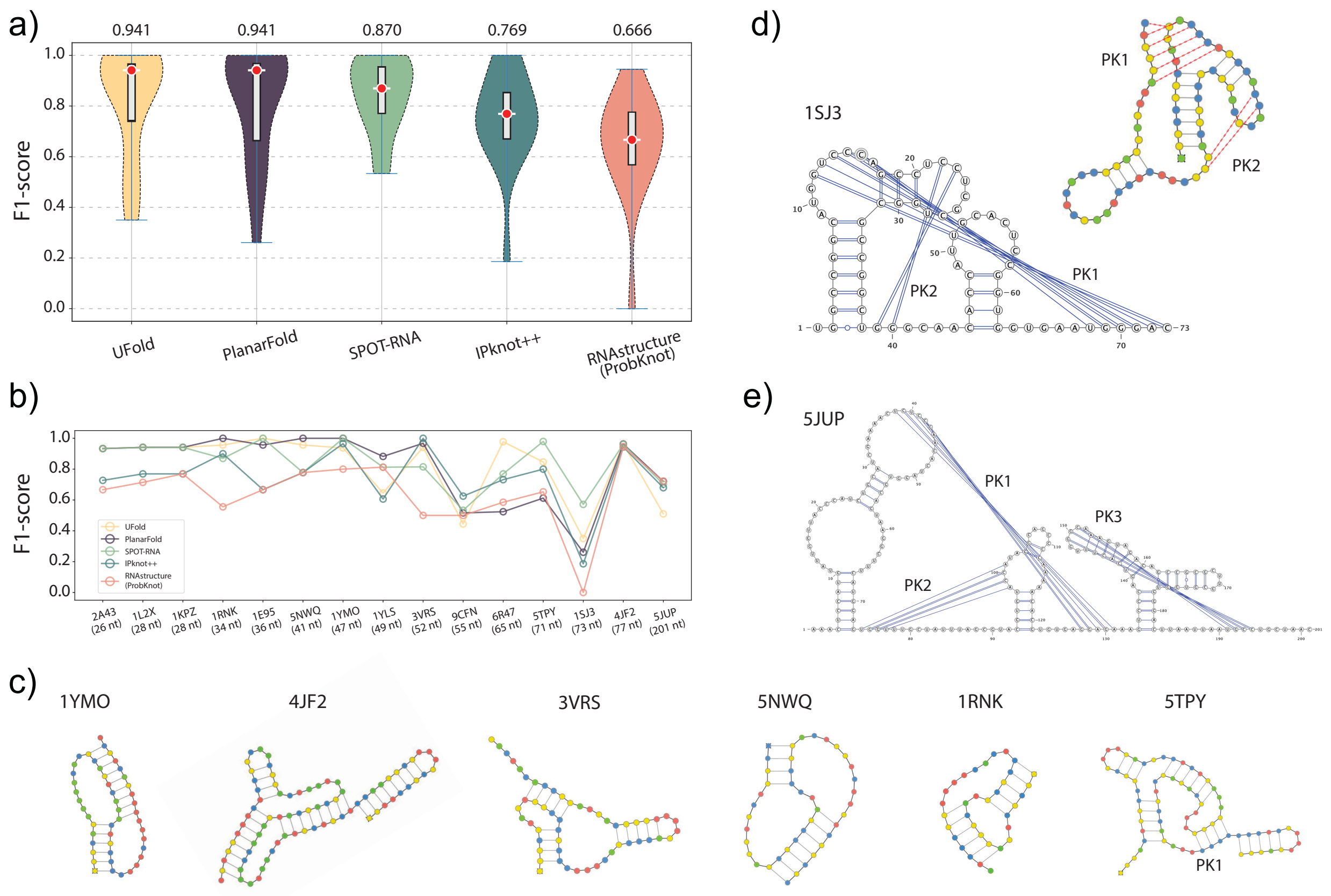

Supplementary Figure 19. Secondary structure prediction of 15 pseudoknot RNAs.
a. Comparison of five RNA secondary structure prediction programs on the 15 pseudoknot (PK) RNAs. The average/median F1-scores for UFold, PlanarFold, SPOT-RNA, IPknot++, and RNAstructure(ProbKnot) are 0.941/0.823, 0.941/0.815, 0.870/0.841, 0.769/0.743, and 0.667/0.644, respectively. For PlanarFold, 1000 simulated annealing cycles have been performed for each of these RNAs. For each RNA, the conformer with the lowest potential energy among its 1000 conformers is selected as the prediction results. For most RNAs, the secondary structure of lowest energy state is sampled several times, indicating robust sampling and reliable prediction. The inner gray boxes represent the 25th percentile to 75th percentile. The blue whisker spans full data range. **b**. The individual prediction results of 15 PK RNAs with un-nested base pair ratio ranging from 11% to 38%. Of note, among 15 PK RNAs in this dataset, 11 and 7 RNAs are already used as the training data for UFold and SPOT-RNA, respectively, which explains their good performance. UFold exhibited significant decreased prediction performance on 4 un-seen RNAs (1YLS, 9CFN, 1SJ3, 5JUP). In contrast, none of these RNAs are used to train PlanarFold since any RNA having a un-nested base pair ratio larger than 10% is excluded during dataset collection of our TR and TS. The formation of pseudoknotted base pairs are originally forbidden throughout the study (iPK=0) and is only allowed (iPK=1) in this PK-RNA benchmark. **c**. Short PK RNAs predicted by PlanarFold without any restraints. For 5TPY, its pseudoknotted stem (PK1) is only stable under restraint, and will deform when restraints are removed. **d**. For 1SJ3, neither its PK1 (kissing loop) nor PK2 can be simulated in a plane, restricted by the persistence length of RNA single-stranded chain (5.5 Å in PlanarFold). Of note, most kissing loops (e.g. in tRNAs and purine riboswitches) cannot be simulated by PlanarFold. **e**. For 5JUP, its PK2 can form spontaneously and PK1 can form only under strong restraints, while PK3 is impossible to form.

a)

c)

b)

d)

e)

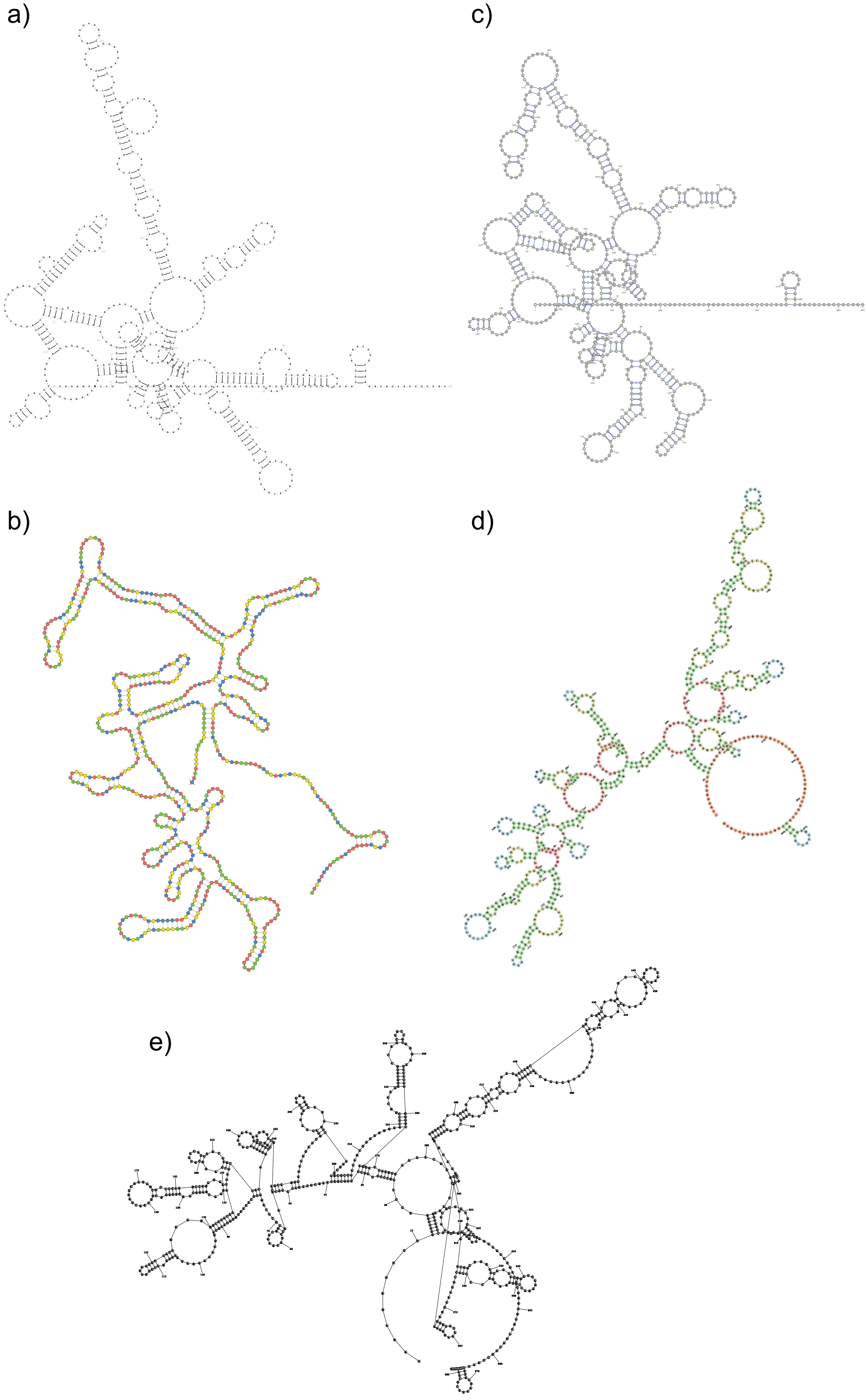

Supplementary Figure 20. Plotting RNA secondary structure using different tools.

The secondary structure of 8A22_4 drawn by: **a.** R2DT[6], **b.** PlanarFold, **c**. Varna[7], **d**. Forna[8], and **e.** RNAstructure[9] (*Draw* module). The overlapping between subdomains is avoided in PlanarFold.

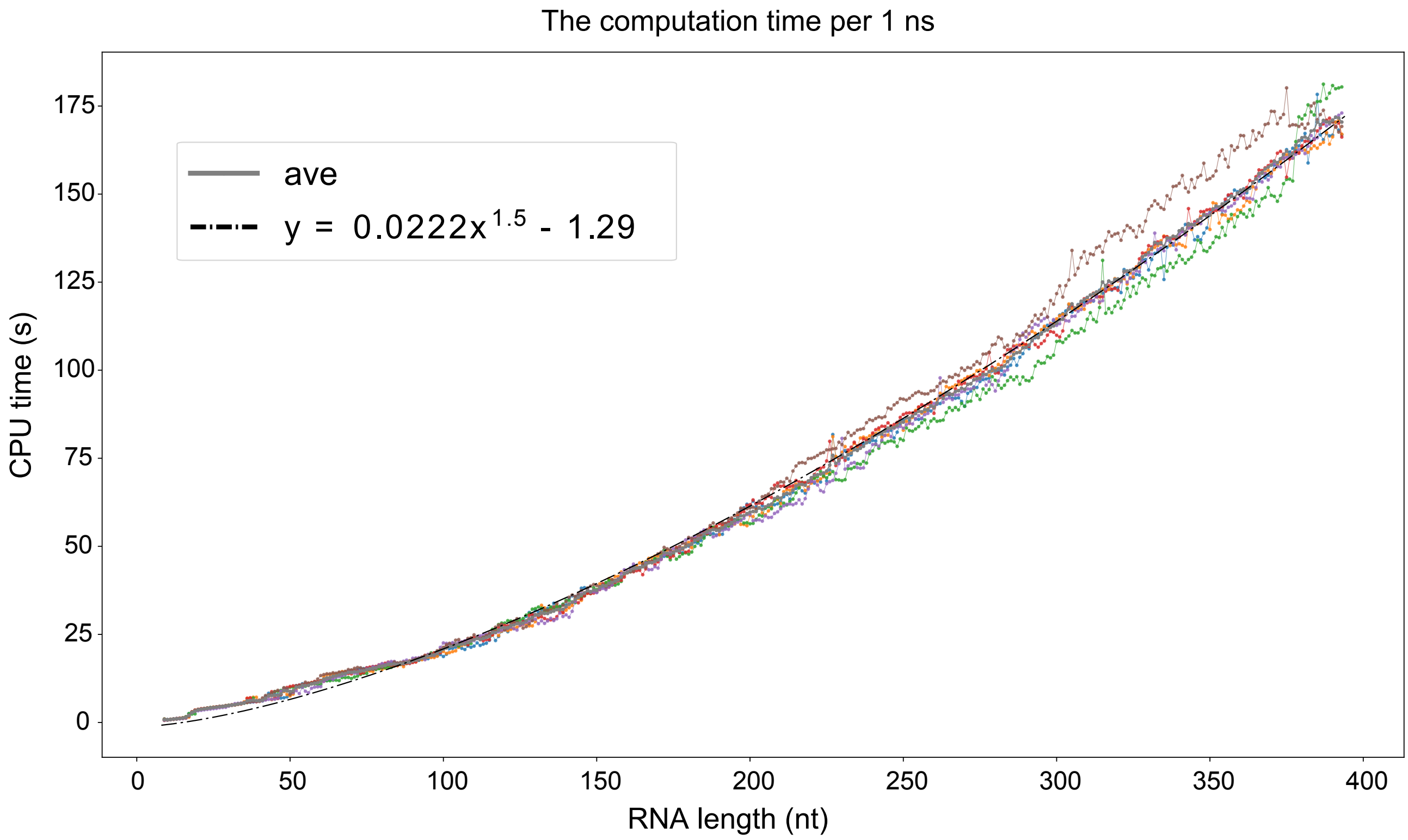

Supplementary Figure 21. Running time versus RNA length.

CPU time as a function of RNA length. The plot shows six co-transcriptional folding trajectories. Each line indicates an RNA chain gradually extending by 1 nt. Each point indicates the CPU time for a simulation duration of 1 ns. The prominent time variations observed for RNAs longer than 200 nt may be attributed to running multiple parallel jobs on a single server. All simulations are performed on AMD Ryzen 9 3900X 12-Core processor (12 cores and 24 threads, 3.8 GHz), equipped with 64 GB of DDR4 memory.

Supplementary tables

Supplementary Table 1. Datasets used for deep-learning based predictors.

The number of RNAs in each dataset is indicated in brackets. The 216 RNAs from PlanarFold's training dataset are collected from the TR1, TS1, TR2, and VL1 of SPOT-RNA. The datasets are named by the original paper, and some datasets are added with a suffix (Rfam or Data) for better distinction. The datasets marked with asterisk indicates currently not accessible thus are not used in this study.

	Original study	Training datasets	Testing datasets
SPOT-RNA	Initial learning	bpRNA-Rfam12.2 (TR0,10814), VL0(1300))	bpRNA-Rfam12.2 (TS0(1305))
	Transfer learning	PDB-2019.3.2 (TR1(120), VL1(31))	PDB-2019.3.2 (TS1(67), TS2(39))
MXfold2	TORNADO set	TrainSetA (3166), TrainSetB (1094)	TestSetA(592), TestSetB(430)
	SPOT-RNA set	bpRNA-Rfam12.2 (TR0(10814), VL0(1300))	bpRNA-Rfam14.2-new(5041), bpRNA-Rfam12.2 (TS0(1305))
	E2efold set	RNAStrAlign(20923)	bpRNA-Rfam14.2-new(5041), ArchiveII(3966)
UFold	Study A	RNAStrAlign (30451)	RNAStrAlign (2826) ArchiveII (3966)
	Study B	bpRNA-Rfam12.2 (TR0)(10814) bpRNA-mut* (Contrafold)(2768)	bpRNA-Rfam14.2-new(5041), bpRNA-Rfam12.2 (TS0)(1305)
	PDB finetune	bpRNA1m-PDB*(669)	PDB-2019.3.2 (TS1(60), TS2(38)) PDB-2020.4.9 (TS3(18))

Supplementary Table 2. F1-score of eleven predictors on testing dataset.

The median and average F1-score of 11 predictors. The "*PlanarFold (sampling)*" indicates the highest F1-score sampled in the conformation pools. The "*PlanarFold (predict)*" indicates the F1-score predicted from the conformers of the lowest energy. P-values are calculated using one-tailed paired t-test using MXfold2 as the reference.

TS (58 RNAs)	PlanarFold (sampling)	MXfold2	SPOT-RNA	BPfold	PlanarFold (predict)	mfold
Median F1-score	0.935	0.818	0.778	0.773	0.770	0.761
Average F1-score	0.876	0.747	0.713	0.692	0.681	0.658
P-value	—	—	0.0197	0.0041	0.0158	0.0032
**TS (58 RNAs)**	**ContraFold**	**IPknot**	**RNAstructure**	**UFold**	**pKiss**	**KnotFold**
Median F1-score	0.758	0.755	0.745	0.672	0.586	0.582
Average F1-score	0.687	0.666	0.673	0.673	0.601	0.563
P-value	3.4×10^{-15}	0.0183	1.2×10^{-13}	1.7×10^{-14}	5.9×10^{-16}	2.1×10^{-14}

Supplementary Table 3. The base-stacking parameters for 36 bp-steps.

This table lists the $k_{stacking}$ term of the base-stacking potential for 36 bp-steps. A bp-step is two continuous (canonical) base-pairs. The vertical and parallel column indicate the 5' and 3' base pair respectively. The values are adopted from 2004 Turner's parameters, but are the negative values (lower free energy corresponds to stronger base-stacking force). E.g., the free energy of AU-AU and GU-UG is -0.9 and 1.3 kcal mol^{-1} respectively in Turner's rules. Units are in kcal mol^{-1} rad^{-2}.

5' \ 3'	AU	UA	GC	CG	GU	UG
AU	0.9	1.1	2.1	2.2	0.6	1.4
UA	1.3	0.9	2.1	2.4	1.0	1.3
GC	2.4	2.2	3.3	3.4	1.5	2.5
CG	2.1	2.1	2.4	3.3	1.4	2.1
GU	1.3	1.4	2.1	2.5	0.5	-1.3
UG	1.0	0.6	1.4	1.5	-0.3	0.5

Supplementary Table 4. Number of parameters of different models.

The 45 parameters of PlanarFold comprises 36 Turner's parameters and 9 force field parameters.

Model	KnotFold	UFold	BPfold	SPOT-RNA	MXfold2	PlanarFold
Number of parameters	~48,100,000	8,641,377	7,962,416	7,759,445	47,346	45

Supplementary Table 5. Force field parameters of PlanarFold.

More detailed description of these parameters can be found in the user manual. Only nine of these parameters are optimized by genetic algorithm, and five (p_{angle}, $p_{stacking}$, $p_{pairing}$, ε, and Q) out of nine are identified to be significant for GA optimization. Consequently, the remaining four (k_{bond}, k_{angle}, k_{stk}, and $k_{pairing}$) out of nine parameters are fixed during final GA optimizations. Other parameters are either equilibrium values or common values that are decided empirically. The parameters marked with asterisk indicates optimized results from genetic algorithm .

	Force field parameters		
Bond	k_{bond}* = 20.0 (kcal mol^{-1} Å^{-2})	r_0 = 5.5 (Å)	
Angle	k_{angle}* = 4.0 (kcal mol^{-1} rad^{-2})	$\theta_0 = \pi$	p_{angle}* = -3.7 (kcal mol^{-1})
Base-stacking	k_{stk}* = 1.5	$\psi_0 = \pi/2$	$p_{stacking}$* = -3.0
Base-pairing	$k_{pairing}$* = 0.4 (kcal mol^{-1} Å^{-2})	d_0 = 10.0 (Å)	$p_{pairing}$* = 9.6 (kcal mol^{-1})
Van der Waals	ε* = 1.1 (kcal mol^{-1})	σ = 9.8 (Å)	
Electrostatic	Q* = -2.93 (kcal$^{1/2}$ mol$^{-1/2}$ Å$^{1/2}$)	λ = 10.0 (Å)	

Supplementary Table 6. Fine-tuning energy difference by adjusting the force-scaling factor. The population of excited state (ES) of T1 hairpin decreases as scaling factor decreases. In this table, only the data of T1 are shown, but this trend applies to other hairpins too. Larger scaling factor stabilizes the ground state (GS) and destabilizes the ES. Each simulation samples 100,000 conformers, and n(Total) = n(GS) + n(ES). The population of GS: p(GS) = n(GS) / n(Total).
The free energy difference is calculated as:

$$\Delta\Delta G = -8.3145 \cdot Temp \cdot ln \frac{p(ES)}{p(GS)} \div 4184$$

The temperature is 283 K, and 4184 is the conversion rate from kcal to Joule.

scaling factor	n(ES)	n(GS)	n(Total)	p(ES)	p(GS)	nES / nGS	ΔΔG (kcal mol^{-1})
0.3	7033	92754	99787	0.070	0.930	0.076	**1.451**
0.33	5855	93821	99676	0.059	0.941	0.062	**1.560**
0.35	4988	94916	99904	0.050	0.950	0.053	**1.657**
0.4	3591	96331	99922	0.036	0.964	0.037	**1.850**
0.5	1947	97985	99932	0.019	0.981	0.020	**2.204**
0.7	536	99398	99934	0.005	0.995	0.005	**2.937**
1.0	116	99845	99961	0.001	0.999	0.001	**3.800**

Supplementary Table 7. Predicted free energy differences of the hairpins with register shift.

	Unit (kcal mol^{-1})	T1-short	T1	T1-GAAA	T1-UUCG	T1-delAU	T1-add1	T1-add2	T2	T2-mirror	T3	T4
RNAstructure	ΔG(GS)	-8.7	-24.6	-24.6	-22.8	-23.2	-26.5	-27.6	-24.9	-25.2	-20.3	-30.0
	ΔG(ES)	-8.8	-23.1	-23.1	-22.3	-21.7	-25.0	-26.6	-23.6	-22.6	-15.8	-26.9
	ΔΔG (ES->GS)	-0.10	1.50	1.50	0.50	1.50	1.50	1.00	1.30	2.60	4.50	3.10
Mfold v2.3	ΔG(GS)	-11.43	-25.82	-25.82	-24.27	-24.42	-28.14	-27.94	-24.95	-23.96	-22.56	-28.27
	ΔG(ES)	-8.11	-21.23	-21.23	-20.33	-19.83	-23.34	-24.02	-25.71	-21.76	-14.75	-26.55
	ΔΔG (ES->GS)	3.32	4.59	4.59	3.94	4.59	4.80	3.92	-0.76	2.20	7.81	1.72
MC-fold	ΔG(GS)	-12.46	-29.69	-30.09	-29.31	-27.08	-31.76	-33.53	-29.18	-29.92	-27.06	-35.80
	ΔG(ES)	-11.00	-28.23	-28.23	-28.23	-25.62	-30.30	-32.10	-28.34	-28.11	-23.45	-31.77
	ΔΔG (ES->GS)	1.46	1.46	1.86	1.08	1.46	1.46	1.43	0.84	1.81	3.61	4.03
MC-fold (non-cano)	ΔG(GS)	-16.15	-35.62	-35.92	-35.45	-33.01	-37.41	-39.18	-35.24	-35.02	-34.56	-36.24
	ΔG(ES)	-15.55	-34.15	-34.34	-31.68	-31.54	-35.95	-37.74	-31.69	-34.74	-33.43	-34.62
	ΔΔG (ES->GS)	0.60	1.47	1.58	3.77	1.47	1.46	1.44	3.55	0.28	1.13	1.62
PlanarFold	ΔΔG (ES->GS)	0.34	1.55	1.55	1.56	1.47	1.67	1.67	1.17	1.51	2.05	2.51
Experiment	ΔΔG (ES->GS)	3.31	1.57	1.58	1.92	1.56	1.41	1.60	1.35	2.92	3.00	2.90

Supplementary Table 8. Predicted free energy differences of transitions with diverse modes.

	Unit (kcal mol⁻¹)	Bistable_911 (313K)	Bistable_55 (298K)	Bistable_46 (298K)	SL1 (288K)	Tri-stable (289K)	SL3 (e-a,298K)
RNAstructure	ΔG(GS)	-12.9	-7.0	-9.7	-29.8	-16.2	-17.6
	ΔG(ES)	-13.8	-9.3	-7.2	-29.5	-15.1	-16.4
	ΔΔG (ES->GS)	-0.90	-2.30	2.50	0.30	1.10	1.20
Mfold v2.3	ΔG(GS)	-13.03	-10.87	-10.89	-30.72	-17.15	-16.13
	ΔG(ES)	-13.01	-8.12	-9.08	-26.17	-15.29	-16.98
	ΔΔG (ES->GS)	0.02	2.75	1.81	4.55	1.86	-0.85
MC-fold	ΔG(GS)	-25.48	-14.29	-17.02	-38.39	-23.36	-34.24
	ΔG(ES)	-30.70	-14.35	-11.43	-35.97	-21.03	-36.61
	ΔΔG (ES->GS)	-5.22	-0.06	5.59	2.42	2.33	-2.37
MC-fold (non-cano)	ΔG(GS)	-27.92	-14.79	-19.11	-42.58	-32.33	-41.53
	ΔG(ES)	-33.23	-16.17	-12.65	-40.96	-33.35	-40.94
	ΔΔG (ES->GS)	-5.31	-1.38	6.46	1.62	-1.02	0.59
PlanarFold	ΔΔG (ES->GS)	1.31	0.53	2.14	1.10	1.00	2.03
Experiment	ΔΔG (ES->GS)	0.17	-0.006	0.86	1.35	-0.13	0.00

Supplementary Table 9. Temperature calibration based on T1-GAAA.

We performed constant temperature equilibrium simulation at several temperatures ranging from 200K to 550K for T1-GAAA, where the free energy difference simulated at 233K, 283K, and 333K match closely with the experimental measurements at 278K, 283K, and 288K respectively.

Temp / Kelvin	n(ES)	n(GS)	n(Total)	p(ES)	p(GS)	nES / nGS	ΔΔG (kcal mol^{-1})
200	2138	97816	99954	0.021	0.979	0.022	2.150
210	2695	97202	99897	0.027	0.973	0.028	2.016
220	2990	96374	99364	0.030	0.970	0.031	1.953
230	3530	96360	99890	0.035	0.965	0.037	1.860
233	3596	96346	99942	0.036	0.964	0.037	1.849
240	4007	95950	99957	0.040	0.960	0.042	1.786
250	4313	95539	99852	0.043	0.957	0.045	1.742
260	4734	95197	99931	0.047	0.953	0.050	1.688
270	5208	94642	99850	0.052	0.948	0.055	1.631
278	5453	94451	99904	0.055	0.945	0.058	1.604
280	5634	94266	99900	0.056	0.944	0.060	1.584
283	5855	93821	99676	0.059	0.941	0.062	1.560
290	6264	93634	99898	0.063	0.937	0.067	1.521
300	6617	93226	99843	0.066	0.934	0.071	1.488
310	6944	92307	99251	0.070	0.930	0.075	1.455
320	7488	92253	99741	0.075	0.925	0.081	1.412
330	7699	92033	99732	0.077	0.923	0.084	1.395
333	8337	91412	99749	0.084	0.916	0.091	1.347
340	8087	91570	99657	0.081	0.919	0.088	1.365
350	8683	90928	99611	0.087	0.913	0.095	1.321
360	9179	90404	99583	0.092	0.908	0.102	1.286
370	9832	89132	98964	0.099	0.901	0.110	1.240
380	10027	89137	99164	0.101	0.899	0.112	1.229
390	10593	88613	99206	0.107	0.893	0.120	1.195
400	11124	87982	99106	0.112	0.888	0.126	1.163
410	11421	87425	98846	0.116	0.884	0.131	1.145
420	12216	86480	98696	0.124	0.876	0.141	1.101
430	12540	85888	98428	0.127	0.873	0.146	1.082
440	12770	85585	98355	0.130	0.870	0.149	1.070
450	13364	84519	97883	0.137	0.863	0.158	1.037
460	13709	83961	97670	0.140	0.860	0.163	1.019
470	13979	82942	96921	0.144	0.856	0.169	1.001
480	9495	55199	64694	0.147	0.853	0.172	0.990
490	14417	81467	95884	0.150	0.850	0.177	0.974
500	15059	80198	95257	0.158	0.842	0.188	0.941
510	15349	79708	95057	0.161	0.839	0.193	0.926
520	15768	78560	94328	0.167	0.833	0.201	0.903
530	15789	77886	93675	0.169	0.831	0.203	0.898
540	16115	76911	93026	0.173	0.827	0.210	0.879
550	16310	75546	91856	0.178	0.822	0.216	0.862

Supplementary Table 10. Kinetic rates of diverse transition modes across large timescale. The default equivalent time can be assumed after linear fitting: 0.047 s (real) = 1 *μ*s (PlanarFold) or 1 s (real) = 20.3 μs (PlanarFold), with a variation of one order of magnitude (Fig.3f). The exchange rate of bistable-46 at 293K is mostly over-estimated, where1 s (real) = 245.2 μs (PlanarFold), conversely, the exchange rate of bistable-55 at 283K is mostly under-estimated, where 1 s (real) = 3 μs (PlanarFold). The free energy difference is calculated as:

$$\Delta\Delta G = -8.3145 \cdot Temp \cdot ln \frac{k_{forward}}{k_{backward}} \div 4184$$

The equivalent ratio X is defined as: 1 s (real) = X μs (PlanarFold). By default, X = 20.

	**Exp $k_{exchange}$ (s^{-1})**	**PlanarFold $k_{exchange}$ (μs^{-1})**	**Equivalent time ratio X**	**Exp $k_{forward}$ (s^{-1})**	**Exp $k_{backward}$ (s^{-1})**	**PlanarFold $k_{forward}$ (μs^{-1})**	**PlanarFold $k_{backward}$ (μs^{-1})**	**Exp ΔΔG (kcal mol^{-1})**	**PlanarFold ΔΔG (kcal mol^{-1})**	**Temp (K)**
**Bistable55**	0.010	0.030	**3.0**	0.0054	0.0047	0.010	0.020	-0.08	0.38	283
	0.014	0.109	**7.6**	0.0077	0.0066	0.021	0.088	-0.09	0.82	288
	0.045	0.399	**9.0**	0.0230	0.0215	0.100	0.299	-0.04	0.64	293
	0.181	1.889	**10.4**	0.0909	0.0900	0.556	1.333	-0.01	0.52	298
**T1-GAAA**	380	31194	**82.1**	13.7	366.3	1122	30072	1.82	1.82	278
	504	34347	**68.1**	30.7	473.3	2018	32329	1.54	1.56	283
	587	39612	**67.5**	45.8	541.2	3242	36370	1.41	1.38	288
**T4**	1157	26218	**22.7**	5.6	1151.4	171	26047	2.95	2.78	278
	1716	29776	**17.4**	10.0	1706.0	479	29297	2.89	2.31	283
	2752	36140	**13.1**	17.1	2734.9	909	35231	2.91	2.09	288
**T2-mirror**	451	31071	**68.9**	1.8	449.2	1237	29834	3.06	1.76	278
	1151	35423	**30.8**	11.3	1139.7	2206	33217	2.60	1.53	283
	1821	38400	**21.1**	23.9	1797.1	3265	35135	2.47	1.36	288
**Tri-stable**	0.7	14.5	**20.6**	0.38	0.30	12.3	2.1	-0.13	-1.00	289
	1.2	15.3	**12.8**	0.59	0.60	13.4	1.9	0.01	-1.14	293
	2.7	25.9	**9.6**	1.16	1.58	23.5	2.4	0.19	-1.36	298
	4.6	24.0	**5.2**	1.57	3.04	22.1	2.0	0.39	-1.44	300
	5.85	29.3	**5.0**	1.74	4.11	27.1	2.2	0.52	-1.52	303
**Bistable-46**	0.074	18.1	**245.2**	0.012	0.062	0.004	18.1	0.95	4.90	293
	0.162	8.9	**55.0**	0.031	0.131	0.024	8.9	0.86	3.50	298
**Bistable-911**	0.03	0.280	**9.3**	0.013	0.017	0.258	0.022	0.17	-1.52	313
**T1-UUCG**	406	36215	**89.2**	13.4	392.6	2033	34182	1.90	1.59	283
**T1-delAU**	347	44881	**129.3**	21.9	325.1	3162	41719	1.52	1.45	283
**T1-add1bp**	393	24498	**62.3**	32.2	360.8	1296	23202	1.36	1.62	283
**T1-add2bp**	351	17598	**50.1**	20.4	330.6	891	16707	1.57	1.65	283
**SL1-wt**	9200	38555	**4.2**	800.0	8400.0	4909	33646	1.35	1.10	288

Supplementary Table 11. End products of co-transcriptional folding and renaturing folding. The percentage of branched conformer is listed. Given the transcription speed for T7 RNA polymerase is approximately 200 to 400 nt/s at 37 °C, we assumed an average speed of 250 nt/s (or 4 ms/nt). By adopting the default equivalent time (20 μs converts to 1 s in real time, see Fig.3f), each transcript should be simulated with 80 ns. However, the population of branched conformers is underestimated using an equivalent ratio of 20 (X=20, see definition of equivalent ratio X at Supplementary Table 10). Of note, the uncertainty of equivalent time ratio can reach up to one order of magnitude (Fig.3f, Supplementary Table 10). We also noticed that Pb-to-Pc transition in the reverse switch shares the same switching mode (nucleation-site-assisted transition) with tri-stable RNA (Supplementary Fig.12e). For tri-stable RNA, the equivalent time ratio gradually decreases from X=20.6 at 16 °C to X=5.0 at 30 °C (Supplementary Table 10). This suggests that the equivalent time of Pb-to-Pc exchange might adopt a lower equivalent time ratio (X≤5) at 37 °C. Hence, we tested another 4 sets of equivalent time ratio (X=1, 2, 5, 10). Clearly, the equivalent time ratio that matches experiment populations best should lie between X=2 and X=5, in agreement with the extrapolation of tri-stable RNA's equivalent time ratio at 37 °C.

		Transcription speed	Direct	Reverse	U36C	Repeats
Co-transcriptional folding	Experiment	~250 nt/s (4 ms/nt)	~100%	~10%	~50%	-
	PlanarFold	250 nt/μs (4 ns/nt, X=1)	100%	30%	90%	40
		125 nt/μs (8 ns/nt, X=2)	**100%**	**15%**	**70%**	40
		50 nt/μs (20 ns/nt, X=5)	100%	3%	48%	40
		25 nt/μs (40 ns/nt, X=10)	100%	0%	33%	40
		12.5 nt/μs (80 ns/nt, X=20)	100%	0%	10%	40
	KineFold	250 nt/s (4 ms/nt)	100%	30%	40%	40
Renaturing folding (Simulated annealing)	Experiment		~50%	~50%	~100%	-
	PlanarFold		~60%	~70%	~90%	200

Supplementary Table 12. Loading conditions of SMD simulations and optical tweezers experiments.

The pulling speed of 20-200 nm/μs and loading rate of 1-20 pN/μs in PlanarFold translate to 400-4000 nm/s and 20-400 pN/s, respectively, by using default equivalent time (1 *μ*s in PlanarFold equals to 0.05 s in real time). The trap stiffness of PlanarFold is chosen to match with the corresponding value of each case study. Loading rate = Pulling speed × Trap stiffness.

	PlanarFold	All-atom MD		OT Experiment
		Study 1 (*Niu, et al.*)[10]	Study 2 (*Baker, et al.*)[11]	
Pulling speed	20-200 nm/μs	0.5-0.05 nm/ns	0.1-0.5 nm/ns	20-200 nm/s
Trap stiffness	0.05-0.1 pN/nm	166.1 pN/nm	6950 pN/nm	0.01-0.1 pN/nm
Loading rate	1-20 pN/μs	83-8.3 pN/ns	695-3475 pN/ns	1-20 pN/s

Supplementary Table 13. Sequences and secondary structures of benchmarked shuffling RNAs.

T1-short	GGGAAGGGCAACUUUCA<br>..(((((....))))). or .(((((.....)))))
T1	GGCGCGAAAGGAAGGGCAACUUUCAAAACGCGCC<br>((((((....(((((....)))))....)))))) or ((((((...(((((.....)))))....))))))
T1-GAAA	GGCGCGAAAGGAAGGGAAACUUUCAAAACGCGCC<br>((((((....(((((....)))))....)))))) or ((((((...(((((.....)))))....))))))
T1-UUCG	GGCGCGAAAGGAAGGUUCGCUUUCAAAACGCGCC<br>((((((....(((((....)))))....)))))) or ((((((...(((((.....)))))....))))))
T1-delAU	GGCGCGAAAGGAGGGCAACUUCAAAACGCGCC<br>((((((....((((....))))....)))))) or ((((((...((((.....))))....))))))
T1-add1bp	GGCGCGAAAGGGAAGGGCAACUUUCUAAAACGCGCC<br>((((((....((((((....))))))....)))))) or ((((((...((((((.....))))))....))))))
T1-add2bp	GGCGCGAAAGGGGAAGGGCAACUUUUCUAAAACGCGCC<br>((((((....(((((((....)))))))....)))))) or ((((((...(((((((.....)))))))....))))))
T2-mirror	GGCGCGAAAACUUUCGCAAGGAAGGAAACGCGCC<br>((((((....(((((....)))))....)))))) or ((((((....(((((.....)))))...))))))
T2	GGCGCGAAAGUUCCUGCAAAGGGAAAAACGCGCC<br>((((((....(((((....)))))....)))))) or ((((((....(((((.....)))))...))))))
T3	GGCGCGAAAGGGUAUGGCAAUGUACAAAACGCGCC<br>((((((.....(((((....)))))....)))))) or ((((((.....((((....))))......))))))
T4	GGGAAGUGCGCGCUUCGGCGCGCCUUUCC<br>((((((.((((((....)))))))))))) or .(((((.((((((....)))))).)))))

Supplementary Table 14. Sequences and secondary structures of benchmarked RNAs.

SL1-wt	GGACUCGGCUUGCUGGAGACGGCAAGAGGCGAGUCC<br>(((((((.(((((((….)))))))...))))))) or (((((((.(((((((....)))))..)).)))))))
Bistable-911	ACAGGUUCGCCUGUGUUGCGAACCUGCGGGUUCG<br>(((((….)))))....((((((....)))))) or .(((((((((.......)))))))))........
Bistable-46	GACCGGAAGGUCCGCCUUCC<br>….((((((....)))))) or ((((....))))........
Bistable-55	GAAGGGCAACCUUCGGGUUG<br>(((((….)))))...... or ......(((((....)))))
Tri-stable	CACUUGAGGCGACUCAGGUGAGGCGACUCACCUG<br>((((((((….)))))))).............. or ..............((((((((....))))))))
SL3	CCCUGCUAGAACCUCCAAACAAGCUCUCAAGGUCCAUUUGUAGGAGAACGUAGGG<br>((((((.....(((.((((...(((.....)))...)))).))).....)))))) or ((((((......((((..(((((.((....))....))))).))))...))))))
P5abc<br>(G176A)	GGCAGUACCAAGUCGCGAAAGCGAUGGCCUUGCAAAGGAUAUGGUAAUAAGCUGCC<br>((((((((((.(((((….)))))...(((....)))...)))).....)))))) or ((((((((((.(((((....)))))..((((...))))...)))).....))))))
P5abc	GGCAGUACCAAGUCGCGAAAGCGAUGGCCUUGCAAAGGGUAUGGUAAUAAGCUGCC<br>(((((.((((.(((((….))))).(((((....))))).)))).......))))) or (((((.((((..((((….))))...((((...))))...)))).......)))))
P5abc<br>(U167C)	GGCAGUACCAAGUCGCGAAAGCGAUGGCCCUGCAAAGGGUAUGGUAAUAAGCUGCC<br>(((((((((..(((((….))))).(((((....)))))..))).....)))))) or (((((((((..(((((....)))))...((.......))...))).....))))))
Direct<br>switch	GGAACCGUCUCCCUCUGCCAAAAGGUAGAGGGAGAUGGAGCAUCUCUCUCUACGAAGCAGAG<br>AGAGACGAAGG<br>....((((((((((((((....))))))))))))))....(((((((((.......)))))))))...... or …..((((((.((((((......((((((((((((...))))))))))))...)))))).)))))).....
Reverse<br>switch	GGAAGCAGAGAGAGACGAAGCAUCUCUCUCUACGAGGUAGAGGGAGAUGGAAAACCGUCUCC<br>CUCUGCCAAGG<br>……(((((((((.......)))))))))....((((((((((((((....)))))))))))))).... or ….((((((.((((((…((((((((((((...))))))))))))......)))))).))))))...
Reverse<br>U36C	GGAAGCAGAGAGAGACGAAGCAUCUCUCUCUACGAGGCAGAGGGAGAUGGAAAACCGUCUCC<br>CUCUGCCAAGG<br>……(((((((((.......)))))))))....((((((((((((((....)))))))))))))).... or ....((((((.((((((...((((((((((.......))))))))))......)))))).)))))).....
SRP	AUCGGGGGCUCUGUUGGUUCCCCCGCAACGCUACUCUGUUUACCAGGUCAGGUCCGGAAGG<br>AAGCAGCCAAGGCAGAUGACGCGUGUGCCGGGAUGUAGCUGGCAGGGCCCCCACCCGGGUC<br>GGCAUGGCA
P5ab	ACAGCCGUUCAGUACCAAGUCUCAGGGGAAACUUUGAGAUGGGGUGCUGACGGACA<br>....(((.((((((((..((((((((....))))))))..)))))))))))...
pri-miR-30c	GUGUAAACAUCCUACACUCUCAGCUGUGAGCUCAAGGUGGCUGGGAGAGGGUUGUUUACUC<br>..(((((((.(((…(((((((((((.........))))))))))).))).))))))).
180-bp<br>hairpin	GUAGUAGUAGUAGUAGUGGUUGUUGUGGUAGUCGUUGUUGUGGUGGUCGUAGUCGUGGUCG<br>UGGUGGUUGUUGUAGUUGUUGUUGUUGUUGUAGUGGUUGUGGUAGUGGUAGUCGUGGUGG<br>UUGUUGUAGUGGUUGUUGUAGUGGUGGUGGGCCACGCGGCGAAAGCCUCAUUGGUCUCGUU<br>UUCGAGACCAAUGAGGCUUUCGCCGCGUGGCCCACCACCACUACAACAACCACUACAACAACC<br>ACCACGACUACCACUACCACAACCACUACAACAACAACAACAACUACAACAACCACCACGACCA<br>CGACUACGACCACCACAACAACGACUACCACAACAACCACUACUACUACUACUAC<br>((<br>((((((((….))<br>))))))))))))))))))))
P4-P6<br>(1GID)	GAAUUGCGGGAAAGGGGUCAACAGCCGUUCAGUACCAAGUCUCAGGGGAAACUUUGAGAUGG<br>CCUUGCAAAGGGUAUGGUAAUAAGCUGACGGACAUGGUCCUAACCACGCAGCCAAGUCCUAA<br>GUCAACAGAUCUUCUGUUGAUAUGGAUGCAGUUC<br>((((((((((…((((((....(((.(((((((((.(((((((((....))))))))).(((((....))))).))))).....)))))))...))))))..)).))))......((((...(((((((((...)))))))))..))))...))))
M-box<br>(2QBZ)	GGGCUUCGUUAGGUGAGGCUCCUGUAUGGAGAUACGCUGCUGCCCAAAAAUGUCCAAAGACG<br>CCAAUGGGUCAACAGAAAUCAUCGACAUAAGGUGAUUUUUAAUGCAGCUGGAUGCUUGUCCU<br>AUGCCAUACAGUGCUAAAGCUCUACGAUUGAAGCCCA<br>((((((((..(((..(((..((((((((..(((.(((((.(((((….((((....))))....)))))....((((((((((.......))))))))))...))))).((((....)))))).))))))).)))...)))..))....))))))).

Supplementary notes

The complete user manual for PlanarFold, including installation instructions, command-line parameters, input/output file formats, and usage examples, is provided as a separate PDF file (PlanarFold_UserManual.pdf) available at Zenodo (https://zenodo.org/records/19397229) and GitHub (https://github.com/Lanxiang-thu/PlanarFold/blob/main/benchmark/PlanarFold_UserManual.pdf). For quick reference, key parameters and example commands are listed below:

1. Basic usage:

The *PlanarFold* is a Python executable script.

```
$ PlanarFold   -i controls.in  -f fparm.in
               -p ssRNA.prmtop  -c ssRNA.inpcrd
               -o run.out  -x run.nc  -r run.rst
               -ntr restraint_ntr.in  -dpr  restraint_dpr.in
```

or

```
$ PlanarFold
```

See more description about the input and output files:

```
$ PlanarFold -h
```

The first command line explicitly describes all the default input and output files. The second command line executes simulation using all default files implicitly.

2. Parameter list:

2.1. Control parameter file

The control parameter file (*control.in*) contains several frequency related parameters, parameters of restraints, the length and time step of simulations, etc. Several basic control parameters follow the name of *AMBER*'s corresponding parameters.

- Parameters are explained as follows:

—*dt* The time step, unit in picosecond (ps). Default = 0.004. Due to single-bead coarse-graining, the time step can be chosen larger than 1 fs and 2 fs. The longer time step is not recommended, as the length of the time step affects the calibrated temperature. A time step of 4 fs is tested through all case studies in the PlanarFold paper, and has been carefully calibrated with experimental temperature and its thermostats. Changing this parameter, then all the quantitative results from the original paper might may no longer apply. Do not change this parameter unless with expertise.

—*nstlim* Number of MD steps to be performed. Default = 1000. Since the parameter *nstlim* is 32-bit (C language int type), the maximum of *nstlim* is 2^{31}-1 (2,147,483,647).

—*ntpr* Every *ntpr* steps, energy information will be printed in a human-readable energy output file (*run.out*). Default = 1000.

—*ntwx* Every *ntwx* steps, coordinates will be printed in NetCDF-formatted coordinate output file (*run.nc*). Default = 1000.

—*nscm* Every *nscm* steps, translational and rotational momentum are removed. Default = 1000. The removal of rotational momentum is extremely important for simulation of small RNAs. Compared to all-atom simulation, the synchronization of all atoms' motion is nearly impossible, so the rotation of the entire system will not happen. However, for our small system, such as a 12-nt hairpin, it will rotate very fast, which contributes to all the kinetic energy, and the RNA will be stuck in one particular conformation.

- Parameters for constant harmonic restraints:

—*ntr* Flag for applying constant harmonic restraints, default = 0. If ntr > 0, the restrained pair-wise residues will be determined from "restraint file" (*restraint_ntr.in*). If ntr = 1, constant harmonic restraints will be applied throughout the simulation. If ntr = 2, constant harmonic restraints will be applied only after the target base pairs approach and form spontaneously. If ntr = 2, extra lines will be written into the energy output file describing the successfully "targeted" base pairs.

—*rcut* The cutoff for applying constant harmonic restraints, unit in Å. Default = 11.5. Only when target residue pairs are in a distance larger than *rcut*, harmonic restraints are applied. In this way, when systems are cooled to 0 Kelvin, most harmonic restraints are released to yield target secondary structure without restraining energy. Setting *rcut* to a large value can be useful for plotting of prescribed secondary structure. However, a large *rcut* can cause futile restraints because target base pairs will not be stretched too close to contact (larger than *vcut*).

—*restraint_wt* The strength of constant harmonic restraints, unit in kcal·mol^{-1}·Å^{-2}. Default = 0.1.

—*restraint_eq* The equilibrium distance of constant harmonic restraints, unit in Å. Default = 10.0.

- Parameters for temperature control:

—*ntt* The option for temperature controlling. Default = 0. If *ntt* = 1, Berendsen thermostat will be used. More sophisticated thermostats will be implemented for specific usage.

—*tautp* Thermostat coupling constant, unit in ps. Default = 0.005. Tight coupling is necessary for PlanarFold as the temperature fluctuation is much stronger than all-atom system.

—*iCollide* The option for mimicking random water collision. Default = 0. If *iCollide* = 1, water with velocity following Maxwell-Boltzmann distribution is assumed to collide with each residue every *nscm* steps. It is important to introduce random collision for simulating RNAs with long handles (SMD). This functionality can be replaced with thermostats considering random fluctuations.

—*ntemp* The number of temperature controlling stages for simulated annealing. Default = 1. If *ntemp* > 1, simulated tempering or annealing is performed.

—*tempi* Initial temperature(s), unit in Kelvin. If more than one *tempi* is described or *tempi* ≠ *temp0*, simulated tempering or annealing is performed.

—*temp0* Reference or final temperature(s), unit in Kelvin. If more than one *temp0* is described or *tempi* ≠ *temp0*, simulated tempering or annealing is performed. The temperature is linearly interpolated from *tempi* to *temp0* within *nstlim* steps.

—*duration* The number of MD steps at each temperature controlling stage. If *tempi* = *temp0, duration* can be set to any non-zero number, indicating a constant temperature simulation. If *tempi* ≠ *temp0, duration* must be set to *nstlim*, such that the temperature can be regulated from *tempi* to *temp0*. When multiple *duration* is assumed, and the sum of all *duration* is smaller than *nstlim*, simulated annealing cycles are performed.

- Parameters for neighbor list:

—*verlet_cut* The cutoff for creating Verlet neighbor lists, unit in Å. Default = 30.0. For residue i, all other residues within *verlet_cut* are stored into its neighbor list. Then, the pair-wise calculation of vdW, electrostatic, and base pairing interactions between residue i and other residues can be searched from its neighbor list, avoiding the search for all the residues. Instead, *Amber* uses box list in combination with periodic boundary condition, which is inappropriate for our coarse-grained small system of non-periodic boundary. V*erlet_cut* must be larger than *vcut*, *bcut*, and *ecut*. When a small *verlet_cut* is chosen, a small *nVerlet* should be employed as well.

—*nVerlet* Every *nVerlet* step, the Verlet neighbor lists are updated. Default = 1000. The choice of a proper *nVerlet* is determined by the velocity of the residue. At least in *nVerlet* step, no residue beyond the Verlet neighbor list of residue i should enter into the range such that it can interact with residue i.

- Parameters for steered MD:

—*ismd* The option for performing steered MD. Default = 0. If *ismd* > 0, steering force will be applied to the 5' and 3' termini of the RNA molecule. Currently, only terminal residues can be steered. The functionality of steering of any pair of residues can be readily achievable in the future. If *ismd* = 1, constant force (c.f.) mode of steering is employed, and the steering force will be kept at *stretch_force* (without any fluctuation). In the future, more realistic c.f. mode, where fluctuation should be allowed as a result of the feedback frequency limited by wet experiment (optical tweezer or AFM), should be implemented. If *ismd* = 2, constant velocity (c.v.) mode of steering is employed. To achieve this, a harmonic restraint is applied to the RNA of interest. By gradually changing the equilibrium value of this harmonic spring, a virtual manipulation of optical trap is mimicked. The c.v. mode of PlanarFold is similar to *Amber*'s SMD mode, which is non-directional steering. To simulate the direction steering, two dumb beads can be added, following *NAMD*'s c.v. mode. The stretching or relaxing speed is determined as (*dist_final* - *dist_init*) / (*nstlim**dt*). If *dist_final* = *dist_init*, passive mode is employed.

—*dumpfreq* Every *dumpfreq* steps, the SMD-related arguments (equilibrium value of steering spring, terminal distance, force, and work) will be written. The

equilibrium values of the steering spring interpolate between *dist_init* and *dist_final* every *dumpfreq* steps.

—*stretch_force* The stretching force in c.f. mode, unit in kcal·mol^{-1}·Å^{-2}. Default = 1.0. 1 kcal·mol^{-1}·Å^{-2} converts to 69.5 pN·Å^{-1}. Of note, the *stretch_force* should be set as the half of the experiment stiffness, as in

—*stretch_spring* The spring constant (or stiffness) in c.v. mode, unit in kcal·mol^{-1}·Å^{-2}. Default = 0.01. 1 kcal·mol^{-1}·Å^{-2} converts to 69.5 pN·Å^{-1}. Of note, the *stretch_spring* should be set as the half of the experiment stiffness, as PlanarFold follows *Amber*'s format, that the Force = 2·*stretch_spring*·(X-X(t)), where X and X(t) is the extension of the RNA and the equilibrium value of the virtual spring, respectively. Careful calculation of the *stretch_spring* is very important to match with experimental trap stiffness.

—*dist_init* The initial equilibrium value of the virtual spring in c.v. mode, unit in Å. Default = 10.0.

—*dist_final* The final equilibrium value of the virtual spring in c.v. mode, unit in Å. Default = 100.0. When *dist_init* < *dist_final*, the RNA is stretched to unfold. When *dist_init* > *dist_final*, the RNA is relaxed to refold. When *dist_init* = *dist_final*, passive mode is simulated.

- Parameters for pseudoknot (un-nested base pairs) simulation:

—*iPK* The option for simulation of pseudoknotted (or un-nested) base pairs. Default = 0. If *iPK* = 0, the pseudoknotted base pairs are excluded during dynamic programming and the nested secondary structure with most base pair will be retained. If *iPK* = 1, both nested and un-nested base pairs are retained (but multiplets are still excluded).

2.2. Force parameter file

The force parameter file (*fparm.in*) contains parameters related to all the potential function and dynamic programming. The most unique design of PlanarFold force field is the three terms in the potential (stem angle, base pairing, and base stacking) are not applied to all residues but are selectively applied to residues identified to form stems. Specifically, these stems are not pre-defined but are determined in real time at each step by the dynamic programming (DP) algorithm.

- Parameters for bond:

—*bond_eq* The equilibrium value (r_0) of bond potential, unit in Å. Default = 5.5.

—*bond_k* The spring constant (k_{bond}) of bond potential, unit in kcal·mol^{-1}·Å^{-2}. Default = 20.0.

- Parameters for stem angle (rigidity):

—*angle_eq* The equilibrium value (θ_0) of stem angle potential, unit in radian. Default = 3.1415926535898 (180° in degree).

—*angle_k* The spring constant (k_{angle}) of stem angle potential, unit in kcal·mol^{-1}·radian^{-2}. Default = 4.0.

—*angle_p* The penalty/bonus (p_{angle}) of stem angle potential, unit in kcal·mol^{-1}. Default = -3.7.

- Parameters for base-stacking (harmonic):

—*stk_ks* The uniform scaling factor (k_{stk}) for base-stacking potential. Default = 1.5.

—*stk_eq* The equilibrium value (ψ_0) of base-stacking potential, unit in radian. Default = 1.57079633 (90° in degree).

—*stk_k* The spring constant ($k_{stacking}[i]$) of base-stacking potential, unit in kcal·mol^{-1}·radian^{-2}. There are 36 stk_*k* parameter for different bp-steps (Fig.2), which is adopted 36 Turner's(2004) parameters for Watson-Crick Helix.

—*stk_p* The penalty/bonus ($p_{stacking}$) of base-stacking potential, unit in radian^2. Default = -3.0.

- Parameters for base pairing (harmonic):

—*bphm_eq* The equilibrium value (d_0) of base pairing potential, unit in Å. Default = 10.0.

—*bphm_k* The spring constant ($k_{pairing}$) of base pairing potential, unit in kcal·mol^{-1}·Å^{-2}. Default = 0.4.

—*bphm_p* The penalty/bonus ($p_{pairing}$) of base pairing potential, unit in kcal·mol^{-1}. Default = 9.6.

—*nHB* The number of hydrogen bonds (nHB[i]). The GC/CG, AU/UA, and GU/UG base pair are assumed to form 3, 2, and 2 hydrogen bonds, respectively. Other non-canonical base pairs are excluded from base pairing potential, even though they are considered in dynamic programming.

- Parameters for van der Waals potential:

—*sigma* The distance (σ) where vdW potential is zero, unit in Å. Default = 9.8.

—*epsilon* The well depth (ε) of vdW potential, unit in kcal·mol^{-1}. Default = 1.1.

—*vcut* The cutoff for calculating vdW potential, unit in Å. Default = 13.5.

- Parameters for electrostatic potential:

—*charge* The charge (Q) of each residue, unit in kcal$^{0.5}$·mol$^{-0.5}$·Å$^{0.5}$. Default = -2.93. Currently, only uniform charge is supported.

—*len_Debye* The Debye length (λ) of electrostatic potential, unit in Å. Default = 10.0.

—*ecut* The cutoff for calculating electrostatic potential, unit in Å. Default = 20.0.

- Parameters for dynamic programming:

—*bcut* The cutoff for calculating inter-residue contacts, unit in Å. Default = 14.0.

—*bulge_p* The penalty for bulge loop and asymmetric internal loop. Default = 0.02.

—*bpFLAG* The factor for modulating different contact strength. bpFLAG is 6, 4, 4, 2, and 1 for GC/CG, AU/UA, GU/UG, GA/AG, and other base pairs, respectively, which is roughly proportional to the hydrogen bonds involving in them.

—*minbpFLAG* The minimum factor of contact that are allowed to form base pairing. Default = 2. For *bpFLAG* ≥ *minbpFLAG*, such contact is extracted during back-tracking of dynamic programming. By setting *minbpFLAG* = 2, only GC, AU, and GU contact will be extracted.

—*minStembp* The minimum number of continuous base pairing allowed to form base pairing potential. Default = 2. After backtracking in dynamic programming, canonical contacts (GC, AU, and GU) are screened into separate stems. If *minStembp* = 1, lone-pairs are allowed. Lone-pairs only have base pairing energy, but no base-stacking and stem angle energy. If *minStembp* = 2, lone-pairs are excluded.

—*min_Loop* The smallest apical loop allowed. Default = 4 (tetraloop). There are also tri-loops identified, but most of them turn to be pentaloop, as their closing base pair are either unstable or non-canonical. For special case where triloop formation is confident, setting *min_Loop* = 3 might be helpful.

- Parameters for holistic scaling of all potential:

—*f_scale* The scaling factor ($k_{scaling}$) that uniformly re-scales all the potential proportionally. Default = 0.33. A larger *f_scale* generally yields a more stagnant trajectory (with rarer conformation change).

2.3. Topology file

The topology parameter file (*ssRNA.prmtop*) follows *Amber*'s ".prmtop" file format. Compared with *Amber*'s complete description of all SECTIONS in prmtop file, PlanarFold's prmtop file only keeps the SECTIONS that are necessary for visualization (by *VMD*) and analyzing tools (*pytraj*). A full description of prmtop file can be found at *https://ambermd.org/FileFormats.php#topology*.

The SECTIONs being kept are as follows:

—POINTERS This section contains the information about how many parameters are present in each section. Compared to all-atom prmtop, most parameters in PlanarFold's prmtop are obsolete.

—ATOM_NAME This section contains the bead name (nucleotide name in PlanarFold) for each bead in the prmtop. Options are A, U, C, and G.

—CHARGE This section contains the charge for each nucleotide in the prmtop. The charges described in prmtop file are currently obsolete. The parameter *"charge"* in the force parameter file (*fparm.in*) describes the uniform charge of nucleotide. In the future, this section can be activated for non-uniform description of charges.

—ATOMIC_NUMBER This section contains the nucleotide index for each nucleotide in the prmtop. A, U, G, and C are indexed as 1, 2, 3, and 4, respectively.

—MASS This section contains the mass for each nucleotide in the prmtop. Currently, the mass of all nucleotides are uniformly assumed as 321.45 $g \cdot mol^{-1}$ for simplicity. In the future, this section can be activated for non-uniform description of masses.

—ATOM_TYPE_INDEX This section contains the nucleotide index for each nucleotide in the prmtop, identical to ATOMIC_NUMBER section. A, U, G, and C are indexed as 1, 2, 3, and 4, respectively.

—NONBONDED_PARM_INDEX This section contains the pointers for each pair of Lennard-Jones bead types for each nucleotide in the prmtop. Index = NONBONDED_PARM_INDEX [NTYPES × (ATOM_TYPE_INDEX(i) − 1) + ATOM_TYPE_INDEX(j)]. In this way, a pair of same residues (e.g., AU and UA) share the same index for LJ potential. This section would be helpful if different residue pairs share different vdW parameter.

—RESIDUE_LABEL This section contains the residue name (nucleotide name in PlanarFold) for each bead in the prmtop, identical to ATOM_NAME section. Options are A, U, C, and G.

—RESIDUE_POINTER This section lists the first atom in each residue. Since PlanarFold uses single-bead model, they are identical to RNA primary sequence position number (from 5' to 3').

—BOND_FORCE_CONSTANT This section contains the bond force constant (k_{bond}) for each bond in the prmtop. The bond force constant described in prmtop file is currently obsolete. The parameter *"bond_k"* in the force parameter file (*fparm.in*) describes the uniform bond force constant of all bonds. In the future, this section can be activated for non-uniform description of bond force constant.

—BOND_EQUIL_VALUE This section contains the bond equilibrium value (r_0) for each bond in the prmtop. The bond equilibrium value described in prmtop file is currently obsolete. The parameter *"bond_eq"* in the force parameter file (*fparm.in*) describes the uniform bond equilibrium value of all bonds. In the future, this section can be activated for non-uniform description of bond equilibrium value.

—ANGLE_FORCE_CONSTANT This section contains the stem angle force constant (k_{angle}) for each stem angle in the prmtop. The stem angle force constant described in prmtop file is currently obsolete. The parameter *"angle_k"* in the force parameter file (*fparm.in*) describes the uniform stem angle force constant of all stem angles. In the future, this section can be activated for non-uniform description of stem angle force constant.

—ANGLE_EQUIL_VALUE This section contains the stem angle equilibrium value (θ_0) for each stem angle in the prmtop. The stem angle equilibrium value described in prmtop file is currently obsolete. The parameter *"angle_eq"* in the force parameter

	file (*fparm.in*) describes the uniform stem angle equilibrium value of all stem angles. In the future, this section can be activated for non-uniform description of stem angle equilibrium value.
—BOND_WITHOUT_HYDROGEN	This section contains a list of every bond in the system in which neither atom is hydrogen. Since PlanarFold is a coarse-grained model with only A, U, C, and G, all the bonds are bonds without hydrogen. This section is important to describe the connectivity of the RNA chain.

Beyond the above-mentioned sections, all other sections are kept vacant, with only header for file format consistency.

2.4. Coordinate file

The coordinate file (*ssRNA.inpcrd*) follows *Amber*'s ".inpcrd" or ".rst" file format. Both coordinate file formats are identical to *Amber*'s corresponding file format. A full description of inpcrd/rst file can be found at *https://ambermd.org/FileFormats.php#topology*. For inpcrd file, bead coordinates and velocities are written in human-readable txt file format. Different from amber, the velocities in inpcrd file will be read by PlanarFold, instead of reassigning initial velocities using build-in procedure. For rst file, the coordinates and velocities are written in NetCDF format, with data stored in double precision (64-bit) for the precise restart of the simulation. Of note, both inpcrd and rst file store three-dimensional coordinates and velocities, with the coordinates and velocities set to zero, to be compatible with VMD and other analysis tools.

Supplementary references

1. Bell, D.R., Cheng, S.Y., Salazar, H. & Ren, P. Capturing RNA Folding Free Energy with Coarse-Grained Molecular Dynamics Simulations. *Sci Rep* **7**, 45812 (2017).
2. R. Salomon-Ferrer, D.A. Case, R.C. Walker. An overview of the Amber biomolecular simulation package. *WIREs Comput. Mol. Sci.* **3**, 198-210 (2013)
3. Justyna, M., Antczak, M. & Szachniuk, M. Machine learning for RNA 2D structure prediction benchmarked on experimental data. *Briefings in Bioinformatics* **24**, bbad153 (2023).
4. Han, G. & Xue, Y. Rational design of hairpin RNA excited states reveals multi-step transitions. *Nat Commun* **13**, 1523(2022).
5. Camara, M.B., Lange, B., Yesselman, J.D. & Eichhorn, C.D. Visualizing a two-state conformational ensemble in stem-loop 3 of the transcriptional regulator 7SK RNA. *Nucleic Acids Res* **52**, 940-952 (2024)
6. Sweeney, B.A. et al. R2DT is a framework for predicting and visualising RNA secondary structure using templates. *Nat Commun* **12**, 3494 (2021).
7. Darty, K., Denise, A. & Ponty, Y. VARNA: Interactive drawing and editing of the RNA secondary structure. *Bioinformatics* **25**, 1974-1975 (2009).
8. Kerpedjiev, P., Hammer, S. & Hofacker, I.L. Forna (force-directed RNA): Simple and effective online RNA secondary structure diagrams. *Bioinformatics* **31**, 3377-9 (2015).
9. Reuter, J.S. & Mathews, D.H. RNAstructure: software for RNA secondary structure prediction and analysis. *BMC Bioinformatics* **11**, 129 (2010).
10. Niu, X.L. et al. Molecular mechanisms underlying the extreme mechanical anisotropy of the flaviviral exoribonuclease-resistant RNAs (xrRNAs). *Nat Commun* **11**, 5496 (2020)
11. Baker, J.L., Dahlberg, T., Bullitt, E. & Andersson, M. Impact of an alpha helix and a cysteine-cysteine disulfide bond on the resistance of bacterial adhesion pili to stress. *Proc Natl Acad Sci U S A* **118**, e2023595118 (2021).
