# Supplementary material for: PlanarFold: a coarse-grained molecular dynamics model of RNA in two-dimensional space
Source: Nat Commun. 2026 Jun 26;17:7998. doi: 10.1038/s41467-026-74729-y (PMC13447859; doi:10.1038/s41467-026-74729-y)
Supplement: Supplementary file 2 [file 41467_2026_74729_MOESM2_ESM.pdf]

Description of Additional Supplementary Files

Supplementary Data 1: The sequence and secondary structures of the training data of PlanarFold

Supplementary Data 2: The sequence and secondary structures of the testing data of PlanarFold
