# Supplementary material for: PlanarFold: a coarse-grained molecular dynamics model of RNA in two-dimensional space
Source: Nat Commun. 2026 Jun 26;17:7998. doi: 10.1038/s41467-026-74729-y (PMC13447859; doi:10.1038/s41467-026-74729-y)
Supplement: Supplementary file 3 [file 41467_2026_74729_MOESM5_ESM.pdf]

nature portfolio

Corresponding author(s): Yi Xue

Last updated by author(s): Jun 3, 2026

Reporting Summary

Nature Portfolio wishes to improve the reproducibility of the work that we publish. This form provides structure for consistency and transparency in reporting. For further information on Nature Portfolio policies, see our Editorial Policies and the Editorial Policy Checklist.

Statistics

For all statistical analyses, confirm that the following items are present in the figure legend, table legend, main text, or Methods section.

n/a	Confirmed	
☐	☒	The exact sample size (n) for each experimental group/condition, given as a discrete number and unit of measurement
☒	☐	A statement on whether measurements were taken from distinct samples or whether the same sample was measured repeatedly
☒	☐	The statistical test(s) used AND whether they are one- or two-sided<br>*Only common tests should be described solely by name; describe more complex techniques in the Methods section.*
☒	☐	A description of all covariates tested
☒	☐	A description of any assumptions or corrections, such as tests of normality and adjustment for multiple comparisons
☐	☒	A full description of the statistical parameters including central tendency (e.g. means) or other basic estimates (e.g. regression coefficient) AND variation (e.g. standard deviation) or associated estimates of uncertainty (e.g. confidence intervals)
☒	☐	For null hypothesis testing, the test statistic (e.g. F, t, r) with confidence intervals, effect sizes, degrees of freedom and P value noted<br>*Give P values as exact values whenever suitable.*
☒	☐	For Bayesian analysis, information on the choice of priors and Markov chain Monte Carlo settings
☒	☐	For hierarchical and complex designs, identification of the appropriate level for tests and full reporting of outcomes
☒	☐	Estimates of effect sizes (e.g. Cohen's d, Pearson's r), indicating how they were calculated

Our web collection on statistics for biologists contains articles on many of the points above.

Software and code

Policy information about availability of computer code

Data collection	RNAsolo (TS dataset, https://rnasolo.cs.put.poznan.pl/archive), SPOT-RNA archive (TR dataset, http://sparks-lab. org/jaswinder/server/SPOT-RNA/), Turner's thermodynamic paramters (https://rna.urmc.rochester.edu/NNDB/turner04/wc.html), CD-HIT (remove sequence redudancy, https://github.com/weizhongli/cdhit/releases)
Data analysis	dssr (secondary structure annotation for PDB files, https://x3dna.org), bpRNA (remove pseudoknots, https://bprna.cgrb.oregonstate.edu/help.php), VMD (trajectory visualization, https://www.ks.uiuc.edu/Research/vmd/), custom python scripts(Python 3.9, Pytraj 2.02, https://amber-md.github.io/pytraj/latest/index.html). Homemade analysis scripts for PlanarFold (https://github.com/Lanxiang-thu/PlanarFold/tree/main/tools)

For manuscripts utilizing custom algorithms or software that are central to the research but not yet described in published literature, software must be made available to editors and reviewers. We strongly encourage code deposition in a community repository (e.g. GitHub). See the Nature Portfolio guidelines for submitting code & software for further information.

Data

Policy information about availability of data

All manuscripts must include a data availability statement. This statement should provide the following information, where applicable:

- Accession codes, unique identifiers, or web links for publicly available datasets
- A description of any restrictions on data availability
- For clinical datasets or third party data, please ensure that the statement adheres to our policy

The public datasets used for secondary structure benchmark are listed in Supplementary Fig. 2 and Supplementary Table 1, which are originally from PDB (https://www.rcsb.org) or RNAsolo (https://rnasolo.cs.put.poznan.pl/archive). The original datasets of SPOT-RNA29, MXfold230, UFold31 listed in Supplementary Table 1 and Supplementary Fig. 2 are publicly available at http://sparks-lab.org/jaswinder/server/SPOT-RNA/, https://zenodo.org/records/4430150, and https://github.com/uci-cbcl/UFold, respectively. The training dataset (TR, 218 RNAs) and testing dataset (TS, 58 RNAs) of PlanarFold are provided in Supplementary Data 1 and 2, respectively. The detailed secondary structure prediction results are provided in Source Data. The following PDB structures were used in the figures, with their accession codes and DOI links: 1GID (https://doi.org/10.2210/pdb1GID/pdb), 2QBZ (https://doi.org/10.2210/pdb2QBZ/pdb), and 1ZIH (https://doi.org/10.2210/pdb1ZIH/pdb).
The various experimental data (optical tweezers, NMR, etc.) used for validation of PlanarFold were obtained from previously published studies. Specifically, the NMR experimental data for the 11 T1-related hairpin RNAs were obtained from Ref. [38], the SL1 of HIV from Ref. [36], the bistable RNAs from Refs. [39–41], the tri-stable RNA from Ref. [42], the SL3 of 7SK RNA from Ref. [43], and the P5abc from Ref. [44]. The co-transcriptional folding experimental data of direct-reverse switch and SRP RNA were obtained from Refs. [47] and [50], respectively. The optical tweezers experimental data of P5ab, pri-miR-30c, and 180-bp hairpin were obtained from Refs. [57], [60], and [61], respectively.
The source data of trajectories and output files of the main figures are deposited on Zenodo73 (https://doi.org/10.5281/zenodo.19985095). The codes to reproduce the main figures and supplementary figures are deposited on Zenodo74 (https://doi.org/10.5281/zenodo.19397229).
Other numerical data for the main figures and supplementary figures and tables are provided in the Supplementary Information and Source Data.
The parameters and reproduction procedures for benchmark tests of PlanarFold can be found in the Supplementary Methods and Supplementary Notes. More detailed instructions to use the software are described in the User Manual deposited on Zenodo74 (https://doi.org/10.5281/zenodo.19397229) and GitHub (https://github.com/Lanxiang-thu/PlanarFold).

Research involving human participants, their data, or biological material

Policy information about studies with human participants or human data. See also policy information about sex, gender (identity/presentation), and sexual orientation and race, ethnicity and racism.

Reporting on sex and gender	*Use the terms sex (biological attribute) and gender (shaped by social and cultural circumstances) carefully in order to avoid confusing both terms. Indicate if findings apply to only one sex or gender; describe whether sex and gender were considered in study design; whether sex and/or gender was determined based on self-reporting or assigned and methods used. Provide in the source data disaggregated sex and gender data, where this information has been collected, and if consent has been obtained for sharing of individual-level data; provide overall numbers in this Reporting Summary. Please state if this information has not been collected. Report sex- and gender-based analyses where performed, justify reasons for lack of sex- and gender-based analysis.*
Reporting on race, ethnicity, or other socially relevant groupings	*Please specify the socially constructed or socially relevant categorization variable(s) used in your manuscript and explain why they were used. Please note that such variables should not be used as proxies for other socially constructed/relevant variables (for example, race or ethnicity should not be used as a proxy for socioeconomic status). Provide clear definitions of the relevant terms used, how they were provided (by the participants/respondents, the researchers, or third parties), and the method(s) used to classify people into the different categories (e.g. self-report, census or administrative data, social media data, etc.) Please provide details about how you controlled for confounding variables in your analyses.*
Population characteristics	*Describe the covariate-relevant population characteristics of the human research participants (e.g. age, genotypic information, past and current diagnosis and treatment categories). If you filled out the behavioural & social sciences study design questions and have nothing to add here, write "See above."*
Recruitment	*Describe how participants were recruited. Outline any potential self-selection bias or other biases that may be present and how these are likely to impact results.*
Ethics oversight	*Identify the organization(s) that approved the study protocol.*

Note that full information on the approval of the study protocol must also be provided in the manuscript.

Field-specific reporting

Please select the one below that is the best fit for your research. If you are not sure, read the appropriate sections before making your selection.

☒ Life sciences ☐ Behavioural & social sciences ☐ Ecological, evolutionary & environmental sciences

For a reference copy of the document with all sections, see nature.com/documents/nr-reporting-summary-flat.pdf

Life sciences study design

All studies must disclose on these points even when the disclosure is negative.

Sample size	All quantitative simulations has been repeated 3 times or more, given different benchmarks.
Data exclusions	Data with high sequence similarity)are removed by CD-HIT following field-common procedure.
Replication	All sequences benchmarked in this study are listed in supporting table. All the parameters and files required to reproduce the MD simulations using PlanarFold are available at https://github.com/Lanxiang-thu/PlanarFold/.
Randomization	Randomness in simulation is introduced by assigning different initial velocities using random number generator in python (numpy).
Blinding	The benchmark cases tested are collected from limited available experiment measurements suitable for secondary structure-level study of RNA. Those data are measured by diverse techniques and laboratories, published over the last two decades.

Reporting for specific materials, systems and methods

We require information from authors about some types of materials, experimental systems and methods used in many studies. Here, indicate whether each material, system or method listed is relevant to your study. If you are not sure if a list item applies to your research, read the appropriate section before selecting a response.

Materials & experimental systems

n/a	Involved in the study
☒	☐ Antibodies
☒	☐ Eukaryotic cell lines
☒	☐ Palaeontology and archaeology
☒	☐ Animals and other organisms
☒	☐ Clinical data
☒	☐ Dual use research of concern
☒	☐ Plants

Methods

n/a	Involved in the study
☒	☐ ChIP-seq
☒	☐ Flow cytometry
☒	☐ MRI-based neuroimaging

Plants

Seed stocks	*Report on the source of all seed stocks or other plant material used. If applicable, state the seed stock centre and catalogue number. If plant specimens were collected from the field, describe the collection location, date and sampling procedures.*
Novel plant genotypes	*Describe the methods by which all novel plant genotypes were produced. This includes those generated by transgenic approaches, gene editing, chemical/radiation-based mutagenesis and hybridization. For transgenic lines, describe the transformation method, the number of independent lines analyzed and the generation upon which experiments were performed. For gene-edited lines, describe the editor used, the endogenous sequence targeted for editing, the targeting guide RNA sequence (if applicable) and how the editor was applied.*
Authentication	*Describe any authentication procedures for each seed stock used or novel genotype generated. Describe any experiments used to assess the effect of a mutation and, where applicable, how potential secondary effects (e.g. second site T-DNA insertions, mosiacism, off-target gene editing) were examined.*
