# Supplementary material for: PlanarFold: a coarse-grained molecular dynamics model of RNA in two-dimensional space
Source: Nat Commun. 2026 Jun 26;17:7998. doi: 10.1038/s41467-026-74729-y (PMC13447859; doi:10.1038/s41467-026-74729-y)
Supplement: Supplementary file 4 [file 41467_2026_74729_MOESM6_ESM.pdf]

nature portfolio

Peer Review File

PlanarFold: a coarse-grained molecular dynamics model of RNA in two-dimensional space

Corresponding Author: Dr Yi Xue

This file contains all reviewer reports in order by version, followed by all author rebuttals in order by version.

A version of this paper was originally rejected for publication by Nature Communications, however that decision was reconsidered after appeal by the authors.

Parts of this Peer Review File have been redacted as indicated to remove third-party material.

Version 0:

Reviewer comments:

Reviewer #1

(Remarks to the Author)
This manuscript presents PlanarFold, a novel model that integrates coarse-grained molecular dynamics with a dynamic programming algorithm to investigate RNA folding in 2D space. The model demonstrates the ability to predict RNA 2D structures, recapitulate folding thermodynamics and kinetics, predict co-transcriptional folding, and explore folding intermediates. The authors tested the model on various systems to validate its efficacy and compared the results to existing models and experimental data.

The concept of coarse-graining each nucleotide as a single bead and conducting simulations in 2D space is intriguing, as it significantly reduces computational costs. However, this raises concerns about whether such planar simulations and single-bead-based force fields can accurately capture RNA folding dynamics and kinetics. For example, in the 2D structure prediction test, the model does not outperform MXfold2 and SPOT-RNA, and the model cannot handle pseudoknots, kissing loops, and other biologically important RNA motifs that can be represented at the 2D level. Furthermore, when using the model to predict thermodynamics for hairpin RNAs, the parameters need to be optimized using NMR-derived data (Page 8). Aside from these major concerns, my additional comments are listed below:

Major:

The model divides the contact map, obtained from simulations, into sub-matrices and employs a dynamic programming algorithm to extract base pairing and stacking information. Would this approach result in the omission of long-range interactions? Why not use the entire map?

For 2D structure prediction, what are the prediction accuracies for pseudoknots and non-canonical base pairs?

Have the authors attempted to use PlanarFold to construct the free energy landscape of an RNA and compare it to that obtained from all-atom MD simulations or 3D CGMD simulations? This could provide validation for the force field of PlanarFold.

What is the computational speed of PlanarFold compared to other 2D structure prediction models? How does the runtime scale with the length of the RNA?

For co-transcriptional folding prediction, the authors compared the model only to Kinefold. How does the model compare to newer methods, such as R2D2, which is an experiment-guided model for folding pathway prediction?

It is recommended to include some discussions on RNA conformational dynamics and co-transcriptional folding prediction in the Introduction section.

Minor:

Typo: Page 20, line 612. “bese” should be “base”.

Page 8, lines 191-193. “In the field of RNA dynamics, it is well-established that various conformation transitions of RNAs can be elucidated through planar representation”. Please cite references here.

(Remarks on code availability)

Reviewer #2

(Remarks to the Author)
I co-reviewed this manuscript with one of the reviewers who provided the listed reports. This is part of the Nature Communications initiative to facilitate training in peer review and to provide appropriate recognition for Early Career Researchers who co-review manuscripts.

(Remarks on code availability)

Reviewer #3

(Remarks to the Author)
In “PlanarFold: a coarse-grained molecular dynamics model of RNA in two-dimensional space,” Xiang and Xue report a creative solution to modeling RNA secondary structure. They introduce a coarse-grained molecular dynamics method where the beads (one per residue) are confined to a plane. This is a good model for capturing RNA secondary structure, which can usually be drawn in a plane without the backbone crossing (depending on the presence of pseudoknots). Additionally, a dynamic programming algorithm is used to adaptively determine helical regions. The force field (potential energy function) then treats helical regions separately from non-helical regions. This is a creative solution for capturing the differences between helical and loop regions as an RNA folds or changes conformations. The manuscript also reports simulations of conformational switches, co-transcriptional folding, and force-extension curves as compared to experiments. These results support that PlanarFold can provide useful models of RNA structures.

Major Revisions:
The first major concern is that the separation between training and testing sets is inadequate. The training and testing sets include RNAs from the same families, i.e. the testing set contains homologs of the training set (Figure S1c). It is well established that this is a problem because the generalizability is not tested when this is done [RNA. 2012. 18: 193; Bioinformatics. 2022. 38: 3892]. The problem almost certainly originates with the requirement that the sequences have <80% identity. It is common for homologous RNAs to have lower than 80% identity.
The PlanarFold model has 9 force field parameters and a small set of additional meta parameters. My estimate is that the fitting will be robust. However, until the training is done properly, the benchmarks (Figure 2) are not reliable.
The second major concern is that many details are missing to fully understand and evaluate the PlanarFold method. One example is the description of the helical parameters (equations 5, 6, and 7). The terms are not defined. I can assume that d is the distance between two paired residues. I do not know what psi represents or what angles theta are included. Lines 487-488 state that the stacking terms are based on Turner rules (given in Table S3). Those entries are Turner rule stacking free energies in kcal/mol. It is not clear how those free energies can be used to determine the force constants, k, in equation 6. Additionally, the Turner rule stacking free energies would cover stabilities for pairing and stacking. This force field uses a separate pairing term (equation 5). Here much more detail is needed to evaluate the parameters. I did, however, note that this detail was raised as a limitation of PlanarFold on lines 652 and 653.
On lines 526-529, the text states that the contact+t map is partitioned into independent regions. It is not clear how this is done. In particular, I am not sure how PlanarFold handles the scenario where nucleotide i is close to j and k. It is then not clear which region(s) i is then assigned to.
Other additional parameters for the dynamics are not provided. There are no statements for what values are used for the mass of the residues or the timestep. There is no statement for the thermostat that is used (although temperatures are referenced). The manuscript also does not state how the equations of motion are integrated.
It is not totally clear whether the accuracy benchmarks shown for PlanarFold in Figure 2 are de novo structure predictions by PlanarFold or re-ranking of decoys generated by RNAstructure. This needs to be clarified.
On line 212, a scaling factor of 0.33 is introduced to calibrate the free energy differences estimated by PlanaFold with experimental free energy differences. I am not sure what this means. How the scaling is applied needs to be explained, ideally with an equation. It is also not clear to me how the temperature calibration is performed (lines 226 to 230).
The manuscript does not explain how the rates were determined from the trajectories (Fig. 3f).

Minor Revisions:
The description of the dynamic programming algorithm is unclear and incorrect on lines 111-118. The manuscript states that “we minimize the sum of inter-residue distances”. Equation 11, however, shows that algorithm maximizes a score that is a product of the reciprocal distance squared and a base pair weight (equation 12). I think the main text should state that pairs are identified that maximize a score based on sequence-based weights and the proximity of residues.
Line 101 states that the potential function for PlanarFold is differentiable. I do not think this is correct because the potential switches abruptly for residues that change between paired and unpaired in the dynamic programming algorithm step. It would be helpful if the manuscript addressed whether the abrupt change in potential as a residue switches state between

paired and unpaired results in any instabilities in the simulations. For example, I could imagine a situation where a residue could switch abruptly between pairing partners that would cause energy to not be conserved. I could imagine that a thermostat would help with this (but the manuscript does not address how temperature regulation is performed). On the other hand, if I am wrong that this is a problem, the manuscript should provide evidence such as literature precedent or empirical evidence showing energy conservation across trajectories.
I am confident that equation 11 is incorrect. The first line should have the terms S(i-1,j+1) + Cmap(i,j), not S(i-1,j+1) + Cmap(i-1,j+1). In other words, shouldn't the score for S(i,j) depend on Cmap(i,j), not the Cmap value of the preceding pair?
The description of the Nussinov algorithm is not correct. Equation 8 should be:
S(i,j) = S(i+1,j-1) + 1
Equation 9 should be:
S(i,j) = max[S(i+1,j), S(i,j-1)
These changes are needed because line 10 states i < k < j, so i is 5' to j. The shorter sequence fragments are therefore referenced as i+1 and j-1. In equation 11, the description of the dynamic programing algorithm developed for PlanarFold, I see that i-1 and j+1 are used. Because these are sequence fragments, it is clearly OK to refer to nucleotides 5' to i and 3' to j.
Figure 1b shows a contact matrix with a grayscale indicated on the right. The grayscale is not explained and the key does not have units. The meaning of the grayscale is needed.
On lines 20 and 21, the text states that for "most non-canonical base pairs, only one hydrogen bond is present." This statement is incorrect. Leontis & Westhof demonstrated that most non-canonical pairs have two hydrogen bonds [NAR, 2002. 30: 3497].
On line 485, a reference should be provided for the origin of the Turner parameters. On line 589, a reference should be provided for dssr.
The manuscript does not report the time cost of the simulations. Please add a table to indicate the time cost for the simulations.

Typos, etc.:
Overall, the writing is clear and concise. There are some minor errors:
Line 106: "on Nussinov algorithm" -> "on the Nussinov algorithm"
Line 117: "algorothm"-> "algorithm"
Line 151: "utilized genetic algorithm" -> "utilized a genetic algorithm"
Line 474: The closing "}" is missing in equation 3.
Line 477: "lenth" -> "length"
Lines 480, 584: "paring"-> "pairing"

(Remarks on code availability)

Reviewer #4

(Remarks to the Author)
The manuscript by Xiang and Xue presents an outstanding study describing the development of PlanarFold, a coarse-grained, two-dimensional molecular dynamics framework for simulating RNA structure and folding dynamics. Understanding RNA structure and dynamics is essential to elucidating RNA function, and computational approaches such as this one offer critical complements to experimental methods. In the present study, the authors focus on simulations of secondary structures and develop an efficient coarse-grained model that combines dynamic programming with molecular dynamics in 2D space. They rigorously benchmarked and demonstrated PlanarFold in several directions, including secondary structure prediction, thermodynamic stability, and kinetic properties of RNA transitions, co-transcriptional folding, and folding of large RNAs. Overall, this is a well-designed, executed, and presented study. This new platform provides a valuable and efficient approach to simulating RNA secondary structure and its transitions and will be of broad interest to the readership of Nature Communications. I highly recommend the publication of this excellent work.

I have a few minor comments for the authors to consider:

1. The authors benchmarked PlanarFold against several established secondary structure prediction tools. However, some of the top-performing programs from recent RNA CASP challenges are not included in the comparisons. Including these methods, particularly those that have demonstrated strong performance in structure prediction, would provide a more comprehensive view of PlanarFold's relative accuracy and help situate its performance within the current landscape.

2. The comparison between simulated and experimental kinetics is compelling. However, the authors currently compare only the overall exchange rate between two states. Since the overall rate can be dominated by either the forward or backward rate when the equilibrium populations are skewed, it would be more informative to report the forward and backward rates separately.

3. Given the good agreement between simulated and experimental exchange rates, it would be valuable to highlight the transition pathways derived from the simulation. In particular, can the authors identify or characterize the transition state(s) within these pathways? This would enhance the mechanistic insights provided by PlanarFold.

4. Many biologically relevant RNAs exhibit more than two conformational states. While the authors present many two-state exchange examples, it would be helpful to include an example of a system with one dominant state and multiple alternative states. This would showcase PlanarFold's ability to capture complex folding landscapes and the kinetics of multi-state

transitions.

5. In Figure 5d, the authors discussed misfolded states of the p5ab RNA with and without force. To improve clarity, it would be helpful to include the secondary structures of these misfolded conformations.

(Remarks on code availability)

Version 1:

Reviewer comments:

Reviewer #1

(Remarks to the Author)
Response to Question 3: The justification for fine-tuning the model on NMR-derived data is unconvincing. The authors responded that the NMR-derived data used to fine-tune the model "essentially serve the same role as the in vitro protein stability data" used to calibrate BioEmu; however, BioEmu was fine-tuned on a large experimental data (~ 500k), whereas PlanarFold was refined using only a small set of hairpin RNAs, making the two incomparable in scale and representativeness.

Response to Question 5: The authors justify excluding PKs and non-canonical pairs largely by arguing they are rare and hard to model, and by pointing to poor performance of predicting PK/noncanonical base pairs by other models. The authors responded that PK formation “more closely resembles tertiary interactions” and is “unlikely to form on a two-dimensional plane”. This is an oversimplification. Pseudoknots are commonly treated as secondary structure by many methods and can be readily represented in 2D contact maps.

Furthermore, PKs can substantially alter RNA thermodynamics, kinetics, and folding pathways, particularly for larger RNAs, so their exclusion is a major limitation for a dynamic simulation tool. Excluding PKs undermines the model’s applicability to many biologically relevant RNAs and should be clearly acknowledged as a key limitation in the manuscript.

Other issues: Some computational details are still missing (e.g., simulation seed for modeling E.Coli SRP). Full method/simulation details and more rigorous benchmarking are needed to support publication.

(Remarks on code availability)

Reviewer #2

(Remarks to the Author)
I co-reviewed this manuscript with one of the reviewers who provided the listed reports. This is part of the Nature Communications initiative to facilitate training in peer review and to provide appropriate recognition for Early Career Researchers who co-review manuscripts.

(Remarks on code availability)

Reviewer #3

(Remarks to the Author)
The revision of “PlanarFold: a coarse-grained molecular dynamics model of RNA in two-dimensional space,” by Xiang and Xue, addresses at least some of my concerns. Overall, I am convinced that the work is novel and interesting. I would still like all the details of the implementation provided in the main text or supplement (rather than stated in a code manual or only in code).

My principal concern with the prior manuscript was that the testing sets were contaminated with homologs of the training data. This version of the manuscript adds an additional benchmark that directly addresses this concern. This is presented on page 7 (lines 170-173) and in Figures S5e and S6. This benchmark, which is capable of assessing transferability to unseen RNA structures, shows that PlanarFold performs about as well as traditional thermodynamic methods (which is impressive). The machine learning methods cannot be assessed on this benchmark without retraining.
The current manuscript still presents the contaminated benchmark up front with claims that PlanarFold and machine learning methods outperform traditional methods. On the one hand, I see that PlanarFold cannot be benchmarked fairly against machine learning methods without retraining them on the same training and testing sets. But the presentation does not clarify that this benchmark is contaminated (and a poor representation of transferability). In my opinion, this benchmark can be included, but should be the one shown in the supplement. At the least, the problems with contamination need to be explained in the main text.

This revision does a better job explaining how things work in PlanarFold, but I am still not satisfied that enough of the

underlying methods are explained to fully evaluate the implementation. This version addressed my concerns about timestep, mass, and thermostat choices. But those were just examples, and I am still wondering about how the engine integrates the equations of motion (e.g. verlet, leapfrog, etc.) and, very importantly, how initial velocities are chosen for the simulations. Also, because Berendsen thermostat is used, are rotational and translation motions removed from the simulation? If not, those motions could use much of the kinetic energy.
I still do not find an explanation as to how the contact map is divided into independent regions. This is mentioned as something that is done on lines 511-512 on page 18. In my opinion, it would be better to let the dynamic programming algorithm address this by letting it run on the full length sequence and full contact map, allowing for branching.
The dynamic programming algorithm recursions (equation 11) include:
$\max[S(i+1,j-1)+Cmap(i,j), S(i+1,j)+Cmap(i,j)+P, S(i,j-1)+Cmap(i,j)+P]$
but I think they should state:
$\max[S(i+1,j-1)+Cmap(i,j), S(i+1,j) +P, S(i,j-1) +P]$
If Cmap(i,j) is the score of a base pair, then only the first option should include that score because that is the state of adding a base pair between i and j. The second or third options are adding unpaired i or unpaired j nucleotides, respectively.

The limitation that pseudoknots are not included is not adequately addressed in the text. This really needs to be in the Discussion because it is a major limitation with PlanarFold. Potential users should understand this.

I was able to build the package and run an example simulation on Linux. I could follow the manual, but I recommend presenting the applications using more step-by-step tutorials. The text does this to some extent, but some details are missing. The Amber-style approach for input and output helps with understanding.
The figures in the manual are very low resolution, including the example file contexts. This made reading the file examples very difficult.
I also found what I think is a bug. I tried running unrestrained simulations. I set ntr=0 in the controls.in file, which should turn off restraints. However, PlanarFold still halts with the message "FilenotFoundError: restraint_ntr.in".

As a comment, the end of page 7 and start of page 8 (Figure S7) looks at the correlation between F1-score and folding energy. It is not clear to me that this should be correlated. Higher energy alternative folds that are close in energy to the native structure do not need to be close in structure to the native structure.

On line 534, the text states that the dataset includes "low nested pairing ratio (<10%)". I think this should state "low unnested pairing ratio (<10%)" because I think this was to remove structures with a large portion of pseudoknotted pairs, i.e. unnested pairs.

(Remarks on code availability)
I did not review the computer code in detail.
I did, however, install PlanarFold and verify that the code works.
The step-by-step installation instructions in the readme were adequate.

As I noted in my review, I think the manual could provide more step-by-step help and more detail. That being stated, the manual is already much better than I have seen for many other software projects. The current state of the manual is not a barrier to publication.

Reviewer #4

(Remarks to the Author)
The revised manuscript by Xiang and Xue has addressed my concerns about the original manuscript. I recommend publishing this excellent work.

(Remarks on code availability)
The code deposition is appropriate.

Version 2:

Reviewer comments:

Reviewer #1

(Remarks to the Author)
Overall, the authors have addressed my original technical concerns by performing additional calculations and revising the manuscript.

The addition of computational details is satisfactory, as the necessary information has been incorporated into the supplementary material. Furthermore, the authors' justification for fine-tuning with limited NMR data is acceptable. I suggest adding a brief description between lines 198–209 to further clarify the rationale for this fine-tuning.

Regarding pseudoknot prediction: among the 15 selected PK-containing RNAs, hairpin–hairpin kissing, one of the most frequent motifs, is missing. If the model is unable to handle this specific motif, the authors should provide an explicit explanation.

(Remarks on code availability)

Reviewer #2

(Remarks to the Author)
I co-reviewed this manuscript with one of the reviewers who provided the listed reports. This is part of the Nature Communications initiative to facilitate training in peer review and to provide appropriate recognition for Early Career Researchers who co-review manuscripts.

(Remarks on code availability)

Reviewer #3

(Remarks to the Author)
On page 8 of the response to reviewers, the authors rebut my concerns about the benchmarks included in the main text. I still do not agree with the decision by the authors to provide the benchmarks in the main text that have homologs to the training data contaminating the test data. The supplement has the correct benchmarks separating homologs properly. The authors are correct that prior studies separated training and testing sets based on sequence identity alone. Recent papers demonstrate that the sequence identity approach is a mistake [RNA. 2026. 32:428; Bioinformatics. 2022. 38:3892], and prior work has done the split of training and testing sets correctly by separating homologs [RNA. 2012. 18:193]. Certainly, many pairs of RNA homologs have less than 80% sequence identity.

With respect to the computational details, I think it would be better to provide all the details in the Supplement rather than the software manual. It would be nice to have these details documented as part of a publication, and the authors might find the documentation of a publication helpful in the future. That being stated, I did find the manual helpful, and this is a matter of opinion.

I have two suggestions for the main text:
On page 6, the text stated that pseudoknots are “beyond the scope of this study”. This text should be revised given the new pseudoknot work in Figure S19.

On page 15 line 424, “benchmared” should be “benchmarked”.

For the Supplement:
Figure S19 has provides new (and interesting) benchmarks with pseudoknot predictions. This needs additional explanation as to how the changes were made to planarfold. For example, the dynamic programming algorithm step needed revision, but I don’t see the revision detailed in the supplement. There are probably other details that also need to be explained.

(Remarks on code availability)

Open Access This Peer Review File is licensed under a Creative Commons Attribution 4.0 International License, which permits use, sharing, adaptation, distribution and reproduction in any medium or format, as long as you give appropriate credit to the original author(s) and the source, provide a link to the Creative Commons license, and indicate if changes were made.
In cases where reviewers are anonymous, credit should be given to 'Anonymous Referee' and the source.
The images or other third party material in this Peer Review File are included in the article's Creative Commons license, unless indicated otherwise in a credit line to the material. If material is not included in the article's Creative Commons license and your intended use is not permitted by statutory regulation or exceeds the permitted use, you will need to obtain permission directly from the copyright holder.
To view a copy of this license, visit https://creativecommons.org/licenses/by/4.0/

We sincerely thank the reviewers for their comments and suggestions, which have led us to make considerable revisions to the manuscript. During this revision, we have performed several additional simulations and analyses to address the corresponding concerns. The main conclusions of this study remain unchanged. The amendment to the equivalent time and its related contents are discussed in Response #17 and #27.

Reviewer #1

This manuscript presents PlanarFold, a novel model that integrates coarse-grained molecular dynamics with a dynamic programming algorithm to investigate RNA folding in 2D space. The model demonstrates the ability to predict RNA 2D structures, recapitulate folding thermodynamics and kinetics, predict co-transcriptional folding, and explore folding intermediates. The authors tested the model on various systems to validate its efficacy and compared the results to existing models and experimental data.

1. *The concept of coarse-graining each nucleotide as a single bead and conducting simulations in 2D space is intriguing, as it significantly reduces computational costs. However, this raises concerns about whether such planar simulations and single-bead-based force fields can accurately capture RNA folding dynamics and kinetics.*

The concept of performing coarse-grained RNA simulations on a two-dimensional plane is novel, so its utility may reasonably be questioned. The justifications are twofold: 1. Our design is based on the hierarchical folding of RNA (secondary structures generally form prior to tertiary structures) and the high stability of RNA secondary structure motifs (canonical base-pairing and base-stacking), while also leveraging the fact that RNA secondary structures can be well presented on a plane; 2. The core objective of our study focuses on dynamic properties dominated by RNA secondary structures, whereas dynamics governed by tertiary motifs (kissing loops, etc.) fall outside the scope of this study (a 3D model aimed at handling these tasks is currently underway).

The thermodynamics and kinetics of RNA secondary structures are fundamentally governed by their free energy landscapes. Therefore, once the free energies of basic structural elements such as base pairing and stacking are reliably defined, the dynamic behavior at the secondary structure level can be adequately captured, regardless of whether the RNA is represented in three-dimensional or two-dimensional space. Single-bead coarse-graining is able to describe the free energy differences accurately between various secondary structures, as demonstrated by the Turner rules. In this context, our method can be viewed as a form of "visualization" for these rules (and MD force parameters). The substantial sampling enhancement achieved through dimensionality reduction and coarse-graining enables the study of long-timescale dynamics that were previously inaccessible. Although the model is highly simplified in form, we have verified its reliability and effectiveness in successfully predicting the dynamic properties of diverse systems by comparing our results with published experimental data.

2. *For example, in the 2D structure prediction test, the model does not outperform MXfold2 and SPOT-RNA, and the model cannot handle pseudoknots, kissing loops, and other biologically important RNA motifs that can be represented at the 2D level.*

We appreciate this specific concern on secondary structure prediction, which is the extended concern of our planar representation.

First, a direct comparison in accuracy between our physics-based method and deep learning models purported for secondary structure prediction is inappropriate. **Deep learning models are often susceptible to overfitting, particularly when trained on limited data, which is a well-known challenge in the field.** Using the fewest parameters, our physics-based model leads in its category, lagging behind deep-learning-based models only by a narrow margin, which should be considered as a satisfactory outcome (the number of parameters of PlanarFold: < *60*; RNAstructure: *hundreds*; SPOT-RNA: *millions*).

More importantly, PlanarFold is fundamentally a dynamic simulation tool whose value extends far beyond secondary structure prediction. Its primary utility lies in accurately modeling the dynamic behavior of RNA at the secondary structure level (as demonstrated by our multiple applications: predicting thermodynamic and kinetic properties, predicting co-transcriptional folding and de novo folding pathways, and predicting refolding/unfolding behaviors under mechanical force). This fulfills a critical need for innovative approaches, since existing deep learning methods and coarse-grained models struggle with such tasks. Moreover, existing deep-learning-based secondary structure predictors cannot predict alternative structures (i.e., they output just a single structure). In contrast, the prediction performance of PlanarFold dramatically increases if the top-3 outputs are considered (F1-score: 0.835 for PlanarFold Top-3 ensemble, outperforming MXfold2 at 0.818; Figure S6). More detailed explanation for our treatment of pseudoknots and kissing loops is provided in Response #5.

3. *Furthermore, when using the model to predict thermodynamics for hairpin RNAs, the parameters need to be optimized using NMR-derived data (Page 8).*

We appreciate the reviewer's concern about our fine-tuning procedure, but the usage of NMR-derived data to fine-tune the parameters is necessary.

1). There is a simplified scenario to explain the necessity of fine-tuning parameters to predict thermodynamics. Assume there are 3 conformations of an RNA: conformer A, B, and C, with F1-score of 1.0, 0.5, and 0.0, respectively. A is the native state. The aim of our genetic algorithm is to optimize a set of parameters that ensures the energy of A is lower than B and C. Therefore, both parameter sets, whether they result in energies of A/B/C: -100.0/-50.0/0.0 kcal/mol or -2.0/-1.0/0.0 kcal/mol, satisfy this optimization goal. In other words, the parameters optimized based on predicting secondary structures only stipulate the relative energy level of each conformer for a given RNA. However, to accurately predict the absolute energy differences among alternative conformers (for instance, -10.0/-5.0/0.0 kcal/mol for A/B/C), experimentally measured free energy data is required for further calibration. The above is the detailed explanation for Lines 199-200: "*Since the parameters optimized by GA only stipulate the relative energy level of conformers, we fine-tuned the absolute energy difference of PlanarFold by fitting NMR-derived thermodynamic data*". For instance, if the NMR-measured energy difference between conformation A and B is 5.0 (unit in kcal/mol), and if the genetic algorithm optimized parameter gives the energy of A/B/C to be -2.0/-1.0/0.0, then we can use a energy scaling factor (Equation 1) of 5.0 to rescale the energy of A/B/C from -2.0/-1.0/0.0 to -10.0/-5.0/0.0, such that the energy difference between A and B are calibrated from 1.0 to 5.0, matching with experimental energy difference. Remarkably, our model, fitted with minimal NMR data (only T1 RNA and T4 RNA), successfully generalizes to predict diverse conformational switches, which is unattainable for deep learning models.

2). In theory, it is nearly impossible to train a valid model capable of quantitatively predicting the free energy difference (or more complex thermodynamic properties) and dynamic behaviors using only static, ground-state PDB structures. A PDB structure provides the static snapshot of an RNA under specific (often crystalline) conditions, typically capturing the ground state (GS) conformation with the lowest energy. The excited state (ES) with the second-lowest energy is low-populated and generally inaccessible to X-ray crystallography and cryo-EM, while several NMR techniques (CPMG, R1rho, etc.) are able to quantitatively characterize the ES and RNA dynamics, which is the reason why we chose NMR-derived data as the gold standard.

3). In practice, for instance, although AlphaFold2 can successfully predict a large number of protein structures, it so far cannot reliably predict the protein dynamics. A recently-published novel model, BioEmu [*Science.*2025;389(6761):eadv9817.], is developed to predict the functional dynamics of proteins and the relative free energy of different conformations. This model is pre-trained on AlphaFold's database to associate the sequence with corresponding structure, and further trained on a vast dataset of MD simulations of thousands of proteins, and eventually fine-tuned based on a large amount of *in vitro* protein stability experiment data. The NMR-derived data we used to fine-tune PlanarFold essentially serve as the same role as the *in vitro* protein stability data they employed to fine-tune BioEmu, to impart thermodynamic knowledge to the model. Furthermore, the current deep-learning-based RNA structure models, either dedicated to secondary structure prediction (MXFold2, SPOT-RNA, UFold, etc.) or tertiary structure prediction (RhoFold+, DRfold, etc.), are incapable of predicting RNA thermodynamics.

4). The validity of our fine-tuned parameter in capturing thermodynamics can also be supported by the successful simulation of co-transcriptional folding pathway and end products, as the co-transcriptional folding is a process governed by thermodynamics, kinetics, and transcription speed. It should be emphasized that the kinetics in our simulations was not subjected to any fine-tuning. We only adjusted a uniform scaling factor (Equation 1) and a dynamic-programming-related parameter to balance the population of GS and ES (see section *"Dynamic Programming"* in *Supplementary methods*). But still, PlanarFold recapitulated the experimental kinetics (exchange rate of transition) quantitatively, further corroborating the effectiveness of our model.

Aside from these major concerns, my additional comments are listed below:

Major:

4. *The model divides the contact map, obtained from simulations, into sub-matrices and employs a dynamic programming algorithm to extract base pairing and stacking information. Would this approach result in the omission of long-range interactions? Why not use the entire map?*

This approach will not cause omission of long-range interactions, as all stems in RNA secondary structures appear as diagonal patterns in the contact map, regardless of whether their paired bases are close or distant in the primary sequence. If "long-range interactions" refers to tertiary interactions, this is not a concern either, since such interactions are spatially constrained on a plane and unlikely to form (discussed in Responses #2 and #5).

In PlanarFold's dynamic algorithm, coordinate information is employed to exclude most of the invalid contacts (distance > 14 Å), as indicated in Figure 1b. Since all RNA stems correspond to diagonal patterns in the contact map, PlanarFold scans the entire map to identify all relevant sub-matrices, each containing one such diagonal pattern.

5. *For 2D structure prediction, what are the prediction accuracies for pseudoknots and non-canonical base pairs?*

First of all, PlanarFold does not predict pseudoknot and non-canonical base pairs. Although pseudoknots (PKs) can be predicted as secondary structure motifs for some models, their formation more closely resembles tertiary interactions and are unlikely to form on a two-dimensional plane. As such, PlanarFold does not account for them. For non-canonical base pairs (other than G-C, A-U, and G-U), their contribution isonly considered in dynamic programming to distinguish symmetric and asymmetric loops, but are not included in the potential energy (base-pairing and base-stacking). The non-canonical base pairs are ignored for two main reasons: 1) their geometric diversity (186 types of non-canonical base pairs, see response #22) cannot be accommodated by a single-bead model; and 2) there is a lack of parameters for their base-pairing and base-stacking interactions.On the other hand, the performance of current predictors for PK and non-canonical base pairs is unsatisfying. For SPOT-RNA, the F1-score of predicting PK and non-canonical base pairs is 0.239 and 0.255 respectively on its testing dataset [*Nat Commun*.2021.12:941]. For UFold, the F1-score of predicting non-canonical base pairs is 0.413/0.637/0.355 on SPOT-RNA’s three testing datasets [*Nucleic Acids Res*.2022.50(3):e14]. Although UFold reported elevated prediction performance, its performance on our dataset is noticeably worse than other predictors (Figure 2), suggesting an overfitting and insufficient generalization ability of the model. Moreover, we tested another model, KnotFold [*Commun Biol*.2024;7(1):297], that claimed improved overall secondary prediction performance by introducing a novel method to handle PKs. However, when we benchmarked this method on our testing dataset (this new comparison is added in Figure 2a, Table S2), it ranked bottom among 11 competitors (a median/average F1-score of 0.582/0.563), which suggests a severe overfitting and that the excess attention to PK base pairs does not improve overall prediction performance.

Although most RNAs (~1/3 of all) contain PK base pairs, the percentage of PK base pairs in all base pairs is low. For instance, in SPOT-RNA’s datasets, the ratio of PK base pairs is only ~3.3% (12,415 out of 376,296, Table S7 of SPOT-RNA paper). Therefore, our model's exclusion of PK prediction is well-justified.

6. *Have the authors attempted to use PlanarFold to construct the free energy landscape of an RNA and compare it to that obtained from all-atom MD simulations or 3D CGMD simulations? This could provide validation for the force field of PlanarFold.*

We have already used PlanarFold to construct the free energy landscape of several RNAs, as indicated in Figure 5d, 6c, and S17c. We did not compare these energy landscapes with all-atom MD simulations due to the prohibitive computational costs, which currently prevent the simulation of large conformational changes (such as the *de novo* folding of large RNAs) that occur over much longer timescales. We did use a 3D CGMD model (simRNA) to simulate the dynamics and folding of several RNAs, but simRNA failed to fold two large RNAs into their native states (see the section *“The de novo folding of large complex RNAs”* and Figure S17f-g). The relative populations of the GS and ES of bistable RNAs and those exhibiting base-pair register shift also significantly deviate from experimental data (Figure S10 and S12). Both results suggest simRNA cannot recapitulate the free energy landscape at present. Other coarse-grained models are either unavailable or inappropriate for de novo folding (requiring secondary structure restraints).

As a more practical strategy, we compared the excited state (ES) and ground state (GS) populations of several RNAs, given that these populations reflect local features of the free energy landscape. The predicted free energy differences between the ES and GS are in good agreement with NMR measurements (Figure 3), corroborating the validity of the force field.

7. *What is the computational speed of PlanarFold compared to other 2D structure prediction models? How does the runtime scale with the length of the RNA?*

We added a new figure (Figure S19) to illustrate the relationship between the simulation runtime and RNA length. The time required for secondary structure prediction by PlanarFold is much longer than that of other methods, since it is an MD simulation program using a sample-and-select strategy. We added a pipeline diagram (Figure S2) to explain the secondary structure prediction procedure of PlanarFold. To emphasize again, PlanarFold is not dedicated to secondary structure prediction, but to the prediction of dynamic behaviors. It typically takes only seconds, or even sub-seconds to predict the secondary structure of an RNA using traditional or DL-based software, but the sampling of diverse conformers using PlanarFold can take hours for the same RNA.

8. *For co-transcriptional folding prediction, the authors compared the model only to Kinefold. How does the model compare to newer methods, such as R2D2, which is an experiment-guided model for folding pathway prediction?*

We only compared PlanarFold with KineFold, since as far as we know, there are no other available methods to predict the co-transcriptional folding pathway. CoFold [*Nucleic Acids Res*. 2013;41(9):e102] is a tool that predicts secondary structure by accounting for the effects of co-transcriptional folding explicitly, but it cannot predict the co-transcriptional folding pathway. RNAKinetics [*J Bioinform Comput Biol*. 2006;4(2):589-96.] is a software that models the dynamics of RNA secondary structure by the means of kinetic analysis of folding transitions of a growing RNA molecule, but its web server is no longer maintained. We have noticed R2D2 before the initial submission, but we did not compare R2D2 with PlanarFold, as it is a method that combines SHAPE-seq experimental data and a sample-and-select strategy to construct a continuum of co-transcriptional folding intermediates. Apparently, it would be unfair to compare our experimental-data-independent model (PlanarFold) to an experimental-data-assisted one (R2D2) in this regard. More importantly, R2D2 demands SHAPE-seq data as input, which are unavailable for our benchmarked RNAs (direct/reverse switch, Figure 4). To use R2D2, one has to perform the co-transcriptional SHAPE-seq experiment on the corresponding RNAs following R2D2’s protocol.

Importantly, we have added co-transcriptional folding simulations of the RNA (signal recognition particle, SRP) from R2D2 paper. First of all, we would like to emphasize that we were already aware of several recent works using SHAPE-seq methods to study co-transcriptional folding of RNA [*Nat Commun.*2023;14(1):7839; *Nucleic Acids Res*.2017;45(12):e109]. The reason we did not conduct simulations and compare with those studies is that their case studies all involve important pseudoknots or ligands, which cannot be handled by PlanarFold. Since SRP from the R2D2 paper does not involve pseudoknots, we have conducted its simulations in this revision.

The simulation results of SRP and their analyses are presented in Figure S15. Remarkably, without utilizing any experimental data (SHAPE reactivities), PlanarFold has recapitulated all the key intermediates and intermediate hairpins (H1, H2, and H3) of SRP detected by the R2D2

method, strongly corroborating the rationality of PlanarFold's force field. We have added the following sentences to *Results*: "*The validity of the co-transcriptional folding simulated by PlanarFold is further corroborated by another test on the E. coli signal recognition particle (SRP). Without utilizing any experimental data, PlanarFold recapitulated all the key intermediates and strand replacement events in the SRP co-transcriptional folding pathway (Fig. S15), which were detected by a recent method dependent on the SHAPE experiments*".

9. *It is recommended to include some discussions on RNA conformational dynamics and co-transcriptional folding prediction in the Introduction section.*

As the reviewer suggested, the second paragraph has been rewritten: "*The in-depth understanding of RNA conformational dynamics can offer essential insights into the mechanisms by which regulatory RNAs execute their biological functions9, but the high flexibility of RNA poses significant challenges to the high-resolution structure determination through X-ray crystallography or cryogenic electron microscopy (cryo-EM). Nuclear magnetic resonance (NMR) spectroscopy, despite its capability to probe dynamics, can only solve the structure of small RNAs (typically less than 50 nt). Moreover, the heterogeneous dynamic ensembles, formed either post-transcriptionally or co-transcriptionally, resist dissection by most bulk methods that obscure individual molecular details10. Recently, single-molecule technologies such as optical tweezers (OT) and atomic force microscopy (AFM) have been utilized to explore the mechanical properties and folding process of RNAs under applied force11. Nevertheless, the mechanisms by which free RNA molecules fold into their native structures without external force manipulation remain largely unresolved. A high-efficiency computational framework that can resolve such heterogeneous dynamics and reconstruct the underlying energy landscape is therefore urgently needed.*".

10. Minor:

Typo: Page 20, line 612. "bese" should be "base".

Page 8, lines 191-193. "In the field of RNA dynamics, it is well-established that various conformation transitions of RNAs can be elucidated through planar representation". Please cite references here.

Thank you for pointing out our oversight. The typos have been corrected, and the reference has been added.

Reviewer #2

I co-reviewed this manuscript with one of the reviewers who provided the listed reports. This is part of the Nature Communications initiative to facilitate training in peer review and to provide appropriate recognition for Early Career Researchers who co-review manuscripts.

Reviewer #3

In "PlanarFold: a coarse-grained molecular dynamics model of RNA in two-dimensional space," Xiang and Xue report a creative solution to modeling RNA secondary structure. They introduce a coarse-grained molecular dynamics method where the beads (one per residue) are confined to a plane. This is a good model for capturing RNA secondary structure, which can usually be drawn in a plane without the backbone crossing (depending on the presence of pseudoknots). Additionally, a dynamic programming algorithm is used to adaptively determine helical regions. The force field (potential energy function) then treats helical regions separately from non-helical regions. This is a creative solution for capturing the differences between helical and loop regions as an RNA folds or changes conformations. The manuscript also reports simulations of conformational switches, co-transcriptional folding, and force-extension curves as compared to experiments. These results support that PlanarFold can provide useful models of RNA structures.

Major Revisions:

11. *The first major concern is that the separation between training and testing sets is inadequate. The training and testing sets include RNAs from the same families, i.e. the testing set contains homologs of the training set (Figure S1c). It is well established that this is a problem because the generalizability is not tested when this is done [RNA. 2012. 18: 193; Bioinformatics. 2022. 38: 3892]. The problem almost certainly originates with the requirement that the sequences have <80% identity. It is common for homologous RNAs to have lower than 80% identity. The PlanarFold model has 9 force field parameters and a small set of additional meta parameters. My estimate is that the fitting will be robust. However, until the training is done properly, the benchmarks (Figure 2) are not reliable.*

We acknowledge the limitation of using an 80% sequence identity cutoff. This cutoff, albeit somewhat controversial, was currently widely adopted [e.g., *Nat Commun*.2019;10:5407; 2021;12:941; 2025;16(1):5856] following historical conventions in the field and for practical considerations in constructing datasets based on the limited availability of high-resolution RNA structures. We agree that cross-family validation would be more rigorous and convincing than cross-sequence validation if abundant RNA families are available, but most RNAs discovered to date fall into very limited families, such as tRNA, rRNA, and microRNA. In our datasets, a large proportion of RNAs cannot be classified into well-defined families (140 out of 274 RNAs have no Rfam families, Figure S1c).

In our initial submission, we attempted to create a cross-family dataset (Figure S1a). Specifically, following a prior study [*Brief. Bioinform*.2023;24:bbad153], we compiled new families from Rfam 14.9, removed those existing in Rfam 14.2, and applied an 80% similarity filter, resulting in the "Rfam-unique" dataset. However, this dataset proved less diverse than anticipated, with 834 of its 837 sequences being microRNAs (simple short hairpins). We also noticed the paper [*Bioinformatics.*2022;38:3892] mentioned by the reviewer. Its ArchiveII dataset, however, uses comparative sequence analysis (CSA) for RNA secondary structure annotation. In contrast, all RNAs in the PlanarFold TR and TS sets are annotated based on PDB structures, providing more reliable secondary structure information than CSA-based methods.

To address the reviewer's concern, we have now reconstructed our dataset using a strict family-based division based on Rfam classifications, and the revised results demonstrate that PlanarFold

maintains robust performance even under this more rigorous evaluation framework. Specifically, we rearranged the RNAs from the existing TR+TS dataset into new datasets and retrained the model for cross-family validation. According to the Rfam family classification, we rearranged the RNAs into three datasets: NoRfam, CrossFam1, and CrossFam2. The NoRfam contains 140 RNAs that cannot be classified into existing Rfam families. The remaining 134 RNAs (belonging to 47 Rfam families) were split into CrossFam1 and CrossFam2, each containing 67 RNAs. There are no overlapping families between CrossFam1 and CrossFam2. After splitting the data, we retrained the model using either NoRfam+CrossFam1 or NoRfam+CrossFam2, and tested the model on CrossFam2 or CrossFam1, respectively.

As the results show, the F1-scores of CrossFam1 and CrossFam2 are 0.818 and 0.831, respectively (Figure S6a). We then compared the prediction performance against RNAstructure and mfold (Figure S6b). The total average/median F1-scores across the combined CrossFam1 and CrossFam2 of RNAstructure, PlanarFold, and mfold are 0.831/0.750, 0.819/0.753, and 0.774/0.710. PlanarFold achieves a comparable cross-family prediction performance to RNAstructure and better performance than mfold. The DL-based models (SPOT-RNA, UFold, etc.) were not included in this cross-family test since their training data included many RNAs from CrossFam1 and CrossFam2 as their training data. In addition, we performed three repeats of parameter optimization (using the genetic algorithm). The three parameter sets trained on either NoRfam+CrossFam1 or NoRfam+CrossFam2 converge with those trained on TR dataset (Figure S5e, where the PCA is newly added with the three parameter sets trained on NoRfam+CrossFam1 and the three parameter sets trained on NoRfam+CrossFam2).

We have revised the introduction to state: "*This model demonstrated performance in predicting RNA secondary structure on par with state-of-the-art methods based on free energy or deep learning, as validated on both cross-sequence and cross-family tests*", to strengthen the robustness of our model in predicting RNA secondary structure. We also added cross-family validation results to the secondary structure prediction section: "*In contrast, using a plain potential energy function of 6 terms with only 45 parameters in total (Table S5), PlanarFold achieved a highly sequence-independent prediction performance (Fig. 2b), which is comparable to or even better than more complex DL-based methods and traditional methods. To further evaluate PlanarFold's ability to predict unseen RNA families, we rearranged the datasets according to Rfam families and retrained the model. Overall, PlanarFold achieved performance comparable to RNAstructure and superior to mfold in cross-family prediction (Figs. S5e and S6)*"

12. The second major concern is that many details are missing to fully understand and evaluate the PlanarFold method. One example is the description of the helical parameters (equations 5, 6, and 7). The terms are not defined. I can assume that d is the distance between two paired residues. I do not know what psi represents or what angles theta are included. Lines 487-488 state that the stacking terms are based on Turner rules (given in Table S3). Those entries are Turner rule stacking free energies in kcal/mol. It is not clear how those free energies can be used to determine the force constants, k, in equation 6. Additionally, the Turner rule stacking free energies would cover stabilities for pairing and stacking. This force field uses a separate pairing term (equation 5). Here much more detail is needed to evaluate the parameters. I did, however, note that this detail was raised as a limitation of PlanarFold on lines 652 and 653.

We thank the reviewer for pointing out our omission of the variable definitions. To address this issue, we have modified the Lines 468-473 to: "*where k and p are the spring constant and penalty/bonus of each potential. The variables d, ψ, and θ are indicated in Fig. 1a (right). In the*

base pairing potential, nHB is the number of hydrogen bonds of the base pair and d_0 represents the equilibrium distance. In the base stacking potential, $k_{stacking}$ is assigned according to 36 Turner's stacking parameters (Table S3) and ψ_0 is the equilibrium angle. In the stem angle potential, θ_0 denotes the equilibrium angle. The values of all parameters can be found in Tables S3 and S5. For more details, see the Supplementary methods." We also modified Figure 1 and its caption accordingly. Besides, we have added a new section "*Force Field of PlanarFold*" in *Supplementary methods*, explaining the potential function and the associated parameters of the force field.

Regarding the Turner rule stacking free energies, we apologize for not providing detailed information on the optimization of the force constant. In Equation 6, $k_{\mathrm{stacking}}[i]$ takes the negative value of the Turner parameters, with units of $\mathrm{kcal} \cdot \mathrm{mol}^{-1} \cdot \mathrm{radian}^{-2}$ (Table S3), where *i* indexes the 36 types of base-pair steps. Please note that since the coefficient k_{stk} remains to be optimized, k_{stacking} is only required to be proportional to the Turner parameters. Here we have directly adopted the Turner parameters. The coefficient k_{stk} was tuned manually to ensure reasonable dynamic behavior on RNA and then was optimized using a genetic algorithm based on experimental secondary structure data. Detailed information has been added to *Methods* and *Supporting Method*s (*Force field*). Indeed, Turner rule stacking free energies account for both pairing and stacking effects. However, introducing a separate pairing energy term in PlanarFold is essential, as justified below.

1). The energy functions of PlanarFold and RNAstructure are built on totally different philosophies: PlanarFold includes van der Waals, electrostatics, and other energy terms. In contrast, Turner's rules include free energy contribution of various stem and loop motifs. Therefore, it would be inappropriate to directly transplant Turner rule parameters into our force field without any coordination with other potential terms. Introducing a pairing term in PlanarFold helps resolve this incompatibility and creates room for decomposing the composite contributions of stacking and pairing effects.

2). PlanarFold is designed to simulate the dynamic behavior of RNA secondary structures. During RNA folding, explicit base-pairing interactions are required to bring two distal nucleotides into proximity and stabilize stacking. Moreover, PlanarFold needs a term to maintain a suitable distance between the two paired residues. More importantly, a base pairing term can distinguish the anisotropic mechanic property of a bp-step (two continuous base pairs). For instance, a bp-step comprised of a continuous G-C and A-U exhibits different tensile strength at two ends, with a higher mechanic resistance at the G-C end than the A-U end. Such anisotropy cannot be reflected by the base-stacking term alone.

3). Optimizing the 36 stacking free energies as independent parameters, rather than adopting the Turner values directly, may seem preferable. However, during the initial submission, we have attempted to co-optimize these 36 Turner parameters together with force field terms, but this led to non-convergence and overfitting. Consequently, this study was limited to optimizing only 5 force field parameters. In this revision, we further tested whether the 36 Turner parameters could be optimized separately to decouple the base stacking contribution. Both genetic algorithm (GA) and Bayesian optimization were applied. In three GA runs, although the training set F1-score reached ~0.935, the test set score was only ~0.70, indicating severe overfitting compared to the results of optimizing only 5 force field parameters (training: 0.915, test: 0.771, Figure S5). Principal component analysis revealed no convergence among the 1003 parameter sets sampled (Figure R1), in contrast to the convergent parameter sets of optimizing only 5 force field parameters (Figure S5). Bayesian optimization also performed poorly, with a maximum F1-score of ~0.89, underscoring the challenge of navigating the vast 36-dimensional parameter space. Therefore,

refining Turner's parameters to isolate a pure base stacking term for PlanarFold is currently infeasible. Directly incorporating them, although not ideal, remains the most feasible and pragmatic solution.

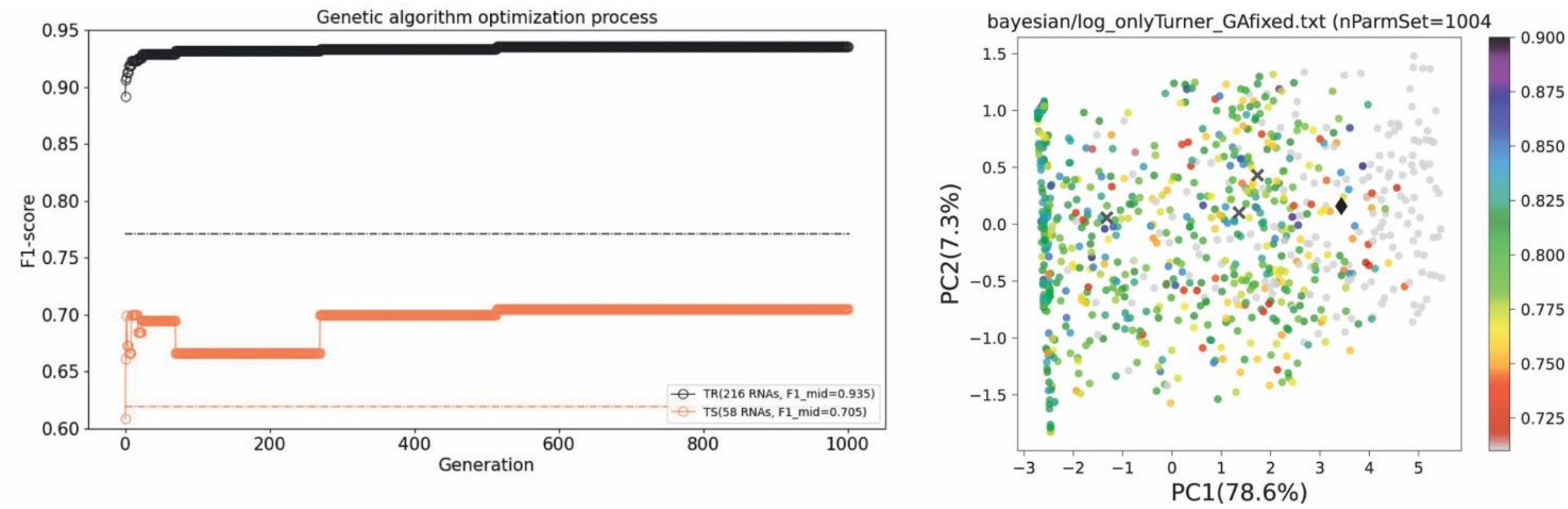

Figure R1. Optimization of 36 Turner helix parameters. **a** The optimization process of genetic algorithm. A population of 1000 evolved for 1000 generations. There are 36 Turner helix parameters being optimized, with each parameter having a value range within the initial value plus or minus 1.0 kcal/mol. The final F1-score (median) of TR and TS is 0.935 and 0.705. **b** Principal component analysis (PCA) of parameter sets from Bayesian optimizations and GA optimizations. There are 1000 parameter sets from Bayesian optimization and 3 parameter sets from GA optimization (black crosses), which are optimized on the identical parameter space (value range of each parameter). The set of original Turner parameters is marked as diamond (black). The color bar indicates the median F1-score of TR under each parameter set.

Although in line 629 we mentioned that the overestimated strength of the base-pairing might account for the discrepancies between the simulation and the OT experiment, it is only one of the possible reasons for these discrepancies. For instance, the buffer conditions (such as cation type and concentration) and the simplified handles that connect the target RNA may also contribute. We also speculate that the loading rates in the simulation may be distorted from the experiment as the equivalent time of PlanarFold has one order of magnitude of uncertainty, since the higher loading rates are well-established to increase the hysteresis in OT experiments.

We have added the following sentences to *Methods:* "*Further independent optimization of the 36 Turner stacking parameters to separate the contribution of base stacking and base pairing may alleviate these issues but has proven impractical due to the large parameter space and scarce data. Therefore, those fine-tuned parameters are employed for the following simulations of pri-miR-30c and the 180-bp hairpin as well, to match the higher pulling speeds (20-fold higher than their corresponding experimental speed).*"

13. On lines 526-529, the text states that the contact+t map is partitioned into independent regions. It is not clear how this is done. In particular, I am not sure how PlanarFold handles the scenario where nucleotide i is close to j and k. It is then not clear which region(s) i is then assigned to.

We have added a detailed description of the dynamic programming algorithm used in PlanarFold in the *Supporting Methods* (see section "*Dynamic programming*"). The complete source code has previously been made available on GitHub (*https://github.com/Lanxiang-thu/PlanarFold*). The DP-related code can be found in "*PlanarFold/src/gradient.c*". For convenience, an explanation of the methods is also provided below.

First of all, the contact map is constructed only with pairwise contacts within the cutoff of 14 Å, which will yield a contact map as Figure 1b (right, upper-right half of the map) depicted. We have devised an algorithm to recursively search for the neighboring valid contacts for a given contact. For instance, starting from Cmap(i,j), the algorithm examines its 8 neighboring contacts. Each of these contacts then recursively searches for its own 8 neighboring contacts, and so on. When a recursion cycle completes (back to Cmap(i,j)), indicating no remaining unvisited neighboring contacts, an independent region is identified. In Figure 1b, four such recursion cycles are performed, identifying four non-neighboring regions. This might seem a heavy computational burden, but we have implemented several optimizations to accelerate this recursion process.

When nucleotide i is within proximity of both j and k (within the cutoff of 14 Å for valid contact formation), two contacts, Cmap(i,j) and Cmap(i,k), will both appear in the contact map. If Cmap(i,j) and Cmap(i,k) belong to the same helix region, dynamic programming will determine which one of them to retain. If Cmap(i,j) and Cmap(i,k) belong to different helix regions, there may (though not necessarily, since nucleotide j and k could be closer to other nucleotides) be two base pairs identified by DP. In such cases, the base pair involving nucleotide i with the larger Cmap value is retained. We prohibit the occurrence of multiplets, as most of multiplets identified in our model are artifacts. Multiplets form more readily in a single-bead model where nucleotides can be readily close to more than 3-4 other nucleotides. In reality, however, the base plane of a nucleotide rarely forms hydrogen bonds with more than 3 nucleotides, as real hydrogen bonds are both distance-dependent and angle-dependent. In contrast, hydrogen bonds are only distance-dependent in PlanarFold. Although a small portion of the triplets does exist in reality, the triplets are also prohibited in PlanarFold as the non-canonical base pairs associated within triplets is currently not considered in PlanarFold. In another view, triplets might best be regarded as tertiary motifs.

14. *Other additional parameters for the dynamics are not provided. There are no statements for what values are used for the mass of the residues or the timestep. There is no statement for the thermostat that is used (although temperatures are referenced). The manuscript also does not state how the equations of motion are integrated.*

We apologize for not having represented the details about MD simulation in *Methods*. Due to the journal's length restrictions for the *Main text* (< 5000 words) and *Methods* (< 3000 words), we summarized all the MD-related parameters (including the force field parameters, mass of the residue, timestep, temperature controlling parameters, other control parameters, etc.) into a comprehensive user manual (46 pages), which was uploaded along with the manuscript during the initial submission. It elaborates on all MD-related details of PlanarFold and provides the procedures for reproducing the benchmark works presented in this study.

Still, we agree that it is necessary to clarify the most important parameters in the manuscript. Therefore, we have added a new section "*Molecular Dynamics Simulation*" to explain all the essential MD-related parameters in *Supplementary Methods*, while more technical details in performing MD were already given in the user manual. All source code has also been deposited on GitHub (*https://github.com/Lanxiang-thu/PlanarFold*).

15. *It is not totally clear whether the accuracy benchmarks shown for PlanarFold in Figure 2 are de novo structure predictions by PlanarFold or re-ranking of decoys generated by RNAstructure. This needs to be clarified.*

The benchmark results shown for PlanarFold in Figure 2 represent the *de novo* structure predictions of PlanarFold aided by additional sampling using RNAstructure, rather than merely re-scoring of decoys generated by RNAstructure.

We indeed used the decoys generated by RNAstructure, but the process involved more than mere re-ranking of scores. Since RNAstructure provides only secondary structures and not the exact coordinates of each conformation, we used RNAstructure's predicted base pairs as structural restraints to sample planar conformations consistent with the proposed secondary structure. Because a single secondary structure can correspond to multiple sets of coordinates (and thus slightly different energies), we generated 5 different decoys for each set of restraints. Aside from restrained decoys, we also used unrestrained sampling to generate 1000 conformations for each RNA through 1000 cycles of heating and cooling. More details on the generation of conformation pools can be found in *Methods: Generation of conformation pools*.

We have added a new figure (Figure S2) to visualize the pipeline for predicting RNA secondary structure in PlanarFold (the reproduction procedures have also been elaborated in the user manual). Both restrained conformations (based on RNAstructure predictions) and unrestrained conformations are combined to generate the decoy pool for each RNA. The overlap between restrained conformations and unrestrained conformations decreases as RNA length increases.

16. On line 212, a scaling factor of 0.33 is introduced to calibrate the free energy differences estimated by PlanaFold with experimental free energy differences. I am not sure what this means. How the scaling is applied needs to be explained, ideally with an equation. It is also not clear to me how the temperature calibration is performed (lines 226 to 230).

1). The necessity of energy rescaling has been detailed in Response #3. In brief, we added the scaling factor in Equation 1 to indicate how the scaling is applied. This adjustment only rescales the absolute energy values but maintains relative differences, thus leaving secondary structure predictions unchanged. In Figure S9b, we observe that the free energy difference between the GS and ES of T1 RNA linearly correlates with the force-scaling factor. A value of 0.33 for this factor calibrates the simulated population ratio of GS/ES to match the experimental result.

Specifically, NMR experiments can measure the populations of the GS and the ES directly. By assuming a two-state equilibrium and using Boltzmann distribution of the two states ($\Delta\Delta G = -8.3145 \times 283 \times ln\frac{p(ES)}{p(GS)} \div 4184$, unit in kcal/mol), the free energy difference between them can be calculated. In MD simulations, the ratio of GS to ES can be acquired from a long equilibrium trajectory. By adjusting the GS and ES populations in MD to match their corresponding experimental populations, the free energy difference derived from MD can be brought into agreement with the experimental value.

We have modified the methods section "*The free energy difference between two conformations can be derived from the Boltzmann distribution, as given in Eq. 14, where R is the gas constant, and T is the temperature in Kelvin. The population of ES and GS is indicated by pES and pGS, respectively. In experiments, the free energy difference between GS and ES is obtained from their populations measured by NMR (or other methods). In MD simulation, the ratio of GS and ES can be determined from a long equilibrium simulation. The scaling factor rescales the conformer energies proportionally. A larger scaling factor (> 1) increases the absolute energy difference*

between GS and ES, while a smaller one (< 1) does the opposite. A value of 0.33 best reproduces the experimental populations of GS and ES for T1 RNA"

2). Given the space limit (both the main text and *Methods*) of the journal, the details of temperature calibration were presented in Figure S9c and its caption. Notably, this temperature calibration is omitted in most (if not all) other RNA coarse-grained models as they generally lack accuracy in predicting thermodynamics and kinetics, but is essential for PlanarFold's diverse applications.

17. The manuscript does not explain how the rates were determined from the trajectories (Fig. 3f).

We thank the reviewer for raising this concern about the important technical detail, which has helped us identify a mistake in the formula used to calculate the exchange rates from simulation. Previously, the exchange rates were calculated by dividing the number of transitions between two states by the total simulation time. By definition, one transition is a cycle from state A to B and back to A, or vice versa. After revision, the correct approach has been provided in the Methods (*Evaluation of thermodynamics and kinetics*): "*In PlanarFold, the free energy differences between two states were calculated using Boltzmann statistics, while the exchange rates were determined as* $k_{ex} = k_{forward} + k_{backward}$*. The forward (backward) rates, defined as the rates from state A to B (state B to A), were determined by dividing the number of forward (backward) transitions with retention time at state A (state B).* "

We apologize for this mistake. Using the corrected formulas, the simulated exchange ratesstill show a strong linear correlation with the experimental exchange rates (even with a slightly higher correlation coefficient, 0.965 versus 0.960). However, the speedup ratio has decreased from ~1×10^6 to ~5×10^4, meaning that 1 μs of simulation time now corresponds to about 0.05 s of real time instead of 1 s.

It is worth mentioning that such a 20-fold reduction of speedup will only affect the applications that require timescale matching with real world. Specifically, the three applications as follows:

i). Co-transcriptional folding (Figure 4): to match the transcription speed of T7 polymerase (250 nt/s or 4 ms/nt), the simulation speed was set to 4 ns/nt using a speedup ratio of 1×10^6. Given that the equivalent time has an error of one order of magnitude, we also performed simulations with a simulation speed of 40 ns/nt. We speculated that a simulation speed between 4 ns/nt and 40 ns/nt should reproduce the experiment results best. After time correction, the simulation speed should be set to 80 ns/nt using a speedup ratio of 5×10^4. In this revision, we also added simulations with a simulation speed of 20 ns/nt and 8 ns/nt, and guaranteed more repeats at each simulation speed. We find that, the simulation speed that reproduces the experiment results best is narrowed down between 8 ns/nt and 20 ns/nt, lying within the interval of 4 ns/nt and 40 ns/nt we assumed before. Therefore, this corrected speedup ratio does not affect the major results and conclusions of co-transcriptional folding simulations.

ii). Mechanical unfolding/refolding (Figure 5): to simulate the experimental pulling speed (1-100 nm/s), we set our simulated pulling speed as 1-100 nm/μs using a speedup ratio of 1×10^6. However, after time correction, the simulation speed should be set as 0.05-5 nm/μs using a speedup ratio of 5×10^4, which means that the simulation time required to simulate the same RNA will be 20 times that of before. Unfortunately, even the simulation of the smallest RNA (P5ab, Figure 5b) in this application takes ~3 days. After speedup ratio correction, such simulation will take ~ 60 days, which is computationally unaffordable. Therefore, when simulating optical tweezers

experiments, we adopted the pulling speed 20 times higher than the corresponding experiment pulling speed.

iii). De novo folding (Figure 6): the P4-P6 can fold as fast as ~90 ns in our simulation; we therefore have speculated the real time scale should be ~90 ms using a speedup ratio of 1×10^6. After time correction, it should be ~4.5 ms using a speedup ratio of 5×10^4.

Other applications such as 2D structure predictions and free energy difference predictions are not affected by this time correction

Minor Revisions:

18. The description of the dynamic programming algorithm is unclear and incorrect on lines 111-118. The manuscript states that "we minimize the sum of inter-residue distances". Equation 11, however, shows that algorithm maximizes a score that is a product of the reciprocal distance squared and a base pair weight (equation 12). I think the main text should state that pairs are identified that maximize a score based on sequence-based weights and the proximity of residues.

We thank the reviewer for pointing out this ambiguity. Following the reviewer's suggestion, we have adopted the phrase "*maximize a score based on basepair-dependent weights and on the proximity between residues*" in the revised version.

19. Line 101 states that the potential function for PlanarFold is differentiable. I do not think this is correct because the potential switches abruptly for residues that change between paired and unpaired in the dynamic programming algorithm step. It would be helpful if the manuscript addressed whether the abrupt change in potential as a residue switches state between paired and unpaired results in any instabilities in the simulations. For example, I could imagine a situation where a residue could switch abruptly between pairing partners that would cause energy to not be conserved. I could imagine that a thermostat would help with this (but the manuscript does not address how temperature regulation is performed). On the other hand, if I am wrong that this is a problem, the manuscript should provide evidence such as literature precedent or empirical evidence showing energy conservation across trajectories.

We have revised the phrasing in lines 91-94 to "*To monitor the time-resolved dynamics of RNA, we designed a physics-based potential function for PlanarFold that is evaluated at each timestep and is differentiable within a fixed secondary structure configuration, making it fully compatible with an MD sampling scheme*".

In line 92 we emphasized that the potential function of PlanarFold is differentiable since we employ MD sampling scheme. As the reviewer pointed out, the potential terms (base pairing, base stacking, stem angle) switches abruptly if the secondary structure changes. Although the potential energy of PlanarFold may be discontinuous across timesteps, it remains differentiable once the secondary structure is determined at each step via dynamic programming (DP). Since DP is applied at every timestep, the abrupt changes in secondary structure are usually modest and do not cause instabilities or crashes in our simulations. More importantly, although the sudden formation or disruption of certain interactions along the trajectory might result in high potential

energies, these energies are rapidly converted into kinetic energy and effectively buffered by the thermostat. To ensure trajectory stability and energy conservation, we have integrated the Berendsen thermostat into PlanarFold, which does not introduce stochasticity (as in the Bussi thermostat). We also recommend using tighter bath coupling in the thermostat, given the greater fluctuations in coarse-grained bead motion compared to all-atom systems. Additionally, translational and rotational momentum of the system is removed to maintain the reliable RNA dynamics. All of these have been described in the user manual.

We did not encounter any simulation crash with a timestep of 4 fs, provided the initial conformation was reasonable. A timestep of 10 fs will face frequent simulation failures; therefore, all simulations in this study employed a 4-fs timestep. It is noteworthy that, although adopting a timestep of 4 fs might seem to be an insignificant acceleration, we have shown that each fs in PlanarFold simulation time is equivalent to 5×10^4 fs in real time (see section "*PlanarFold recapitulates the thermodynamics and kinetics*"). Therefore, a timestep of 4 fs in PlanarFold corresponds to roughly 200 ps in all-atom MD, representing a speedup of more than four orders of magnitude.

It is also worth mentioning that discontinuous potential energies have proven effective in RNA and protein coarse-grained simulations, as exemplified by the discrete MD (DMD) [*Structure*.2008;16(7):1010-8; *RNA*.2008;14(6):1164-73; *JACS*.2009;131(7):2541-6]. In DMD models, the potentials are a series of step functions segmenting the original continuous potential functions. This modeling strategy sacrifices energy continuity to enable enhanced sampling of larger conformational changes.

20. *I am confident that equation 11 is incorrect. The first line should have the terms S(i-1,j+1) + Cmap(i,j), not S(i-1,j+1) + Cmap(i-1,j+1). In other words, shouldn't the score for S(i,j) depend on Cmap(i,j), not the Cmap value of the preceding pair?*

The description of the Nussinov algorithm is not correct. Equation 8 should be:

S(i,j) = S(i+1,j-1) + 1

Equation 9 should be:

S(i,j) = max[S(i+1,j), S(i,j-1)

These changes are needed because line 10 states i < k < j, so i is 5 'to j. The shorter sequence fragments are therefore referenced as i+1 and j-1. In equation 11, the description of the dynamic programing algorithm developed for PlanarFold, I see that i-1 and j+1 are used. Because these are sequence fragments, it is clearly OK to refer to nucleotides 5 'to i and 3 'to j.

We thank the reviewer for pointing out the mistakes in Equations 8 and 9. We have corrected these two equations as suggested. The equation 11 has also been corrected: the indices i-1 and j+1 are replaced with i+1 and j-1, respectively. The reason why equation 11 has several mistakes in indexing is that the practical implementation of dynamic programming uses different indexes for computational efficiency. Our dynamic programming source code has been carefully checked many times during development (the source code remains untouched in this submission for transparency), and we are sure the mistakes in equation 11 are not present in the source code. More details about dynamic programming have been added to *Supporting Methods*.

21. Figure 1b shows a contact matrix with a grayscale indicated on the right. The grayscale is not explained and the key does not have units. The meaning of the grayscale is needed.

The caption of Figure 1b has been updated to include: "*The color bar indicates the value of Cmap(i,j*), with unit in $Å^{-2}$".

22. On lines 20 and 21, the text states that for "most non-canonical base pairs, only one hydrogen bond is present." This statement is incorrect. Leontis & Westhof demonstrated that most non-canonical pairs have two hydrogen bonds [NAR, 2002. 30: 3497].

We thank the reviewer for correcting this misleading statement of the non-canonical base pairs. We have consulted the same paper suggested by the reviewer. Our earlier conclusion was based simply on the average number of hydrogen bonds across all classes of non-canonical base pairs.

To justify this number in a more quantitative manner, we have now collected the hydrogen bond number for 12 classes of base pairing from the original paper (Table R1). These values were extracted from the lower-left corner of the original figures. The three numbers in each table cell are described as follows: "Three numbers are given: (i) the number of observed or potential hydrogen bonds between two nitrogen or oxygen containing groups (i.e. normal hydrogen bonds); (ii) the number of hydrogen bonds involving polarized C-H groups (i.e. AH2, RH8, YHS or YH6); (iii) the number of bridging water molecules". In our analysis, we excluded the hydrogen bonds involving polarized C-H groups due to their lower stability, as well as the hydrogen bonds involving water molecules. For some base pairs, we also added a parenthetical value to indicate the remaining number of hydrogen bonds after removing contributions from protonated states (in A or C).

After these treatments, the distribution of hydrogen bonds across the 12×16 = 192 possible base pair types are as follows: 1 type has four bonds, 11 have three, 73 have two, 50 have one, and 57 have none. As the reviewer correctly noted, most non-canonical pairs (71 out of 192, excluding AU and UA) indeed form two hydrogen bonds. However, there are also 107 that form one or no hydrogen bond. By averaging across the 12 edge families (cWW, tWW, etc.), we estimate that a non-canonical base pair forms approximately 0.8-1.7 hydrogen bonds on average (Table R2). Although this simplified summation is rough and does not account for the varying frequencies of different edge families, it still suggests that non-canonical base pairs generally form fewer hydrogen bonds than canonical ones. Moreover, there are frequent observations of suboptimal non-canonical base pair with only one hydrogen bond in the PDB deposits, consistent with the original paper's statement: "*Thus, while one can generally classify base pairs into one of the 12 families discussed above, a particular base pair may form with a slightly different combination of hydrogen bonds or with the absence of one or more hydrogen bonds, depending on the structural context or on the resolution of the structure*".

Table R1. Number of hydrogen bonds of 12 families of base pairs

cWW	A	C	G	U	tWW	A	C	G	U	cWH	A	C	G	U
A	1-1-0	2(1)-0-0	2-0-0	2-1-0	A	2-0-0	2-0-0	-	2-1-0	A	-	-	2(1)-0-0	1-1-0
C	2(1)-0-0	2(1)-0-0	3-0-0	1-0-1	C	2-0-0	3(2)-0-0	2-0-0	2-0-1	C	-	1-1-0	2(1)-0-0	1-1-0
G	2-0-0	3-0-0	-	2-0-1	G	-	2-0-0	2-0-0	2-0-0	G	2-0-0	-	2-0-0	-
U	2-1-0	1-0-1	2-0-1	2-0-1	U	2-1-0	2-0-1	2-0-0	2-0-0	U	2-1-0	-	1-1-0	1-1-0
tWH	A	C	G	U	cWS	A	C	G	U	tWS	A	C	G	U
A	2-0-0	-	2(1)-0-0	-	A	2-0-0	2-0-0	2-0-0	2-0-0	A	1-1-0	2-0-0	3-0-0	2-0-0
C	2-0-0	1-1-0	2(1)-0-0	-	C	2-0-2	2-0-0	2-0-0	2-0-0	C	2-0-0	2-0-0	3-0-0	2-0-0
G	-	-	2-0-0	1-1-0	G	2-1-1	2-0-0	2-0-1	2-0-0	G	-	2-0-0	-	2-0-0
U	2-0-0	-	1-1-0	1-1-0	U	2-1-1	2-0-0	2-0-0	2-0-0	U	1-1-0	2-0-0	1-0-1	2-0-0
cHH	A	C	G	U	tHH	A	C	G	U	cHS	A	C	G	U
A	-	-	1-0-0	-	A	2-0-0	1-0-0	1-0-0	1-0-0	A	1-2-0	1-1-0	2-1-0	1-1-0
C	-	-	0-1-0	-	C	1-0-0	-	1-0-0	1-0-0	C	-	-	-	-
G	1-0-0	0-1-0	0-1-0	-	G	1-0-0	1-0-0	0-1-0	-	G	3-0-0	2-0-0	4-0-0	2-0-0
U	-	-	-	-	U	1-0-0	1-0-0	-	-	U	-	-	-	-
tHS	A	C	G	U	cSS	A	C	G	U	tSS	A	C	G	U
A	2-1-0	2-0-0	3-0-0	2-0-0	A	2-1-0	2-0-0	3-0-0	2-0-0	A	1-2-0	1-1-0	2-1-0	1-1-0
C	2-0-0	2-0-0	-	2-0-0	C	2-1-1	2-0-0	3-0-0	2-0-0	C	-	-	-	-
G	-	-	1-0-0	-	G	3-1-0	2-0-0	3-0-0	2-0-0	G	3-0-0	2-0-0	4-0-0	2-0-0
U	0-1-0	-	1-0-0	-	U	2-1-0	2-0-1	3-0-0	2-0-0	U	-	-	-	-

According to Leontis&Westhof classification, the base pairs can be classified into 12 families. The number describes the hydrogen bonds. The first is the number of observed or potential hydrogen bonds between two nitrogen- or oxygen-containing groups, i.e. normal hydrogen bonds. The number in the parenthesis is the remaining normal hydrogen bonds after protonation is removed. The second is the number of hydrogen bonds involving polarized C-H groups. The third is the number of bridging water molecules.

Table.R2 Average number of hydrogen bonds of 16 type of base pairs.

	A	C	G	U	A	C	G	U
A	16	14	20	16	1.3	1.2	1.7	1.3
C	15	14	17	14	1.3	1.2	1.4	1.2
G	14	14	17	13	1.2	1.2	1.4	1.1
U	14	10	14	12	1.2	0.8	1.2	1.0

The sum of normal hydrogen bonds from 12 Leontis&Westhof families and the average normal hydrogen bonds.

Another thing worth noting is that, in genuine structures, residues within large internal/bulge loops or multiway loops are frequently observed which do not form any base pairs. Given that PlanarFold's dynamic programming algorithm attempts to pair any two opposite residues (but only A-U, G-U, and G-C are ultimately retained) in the internal loop or bulge loop to avoid asymmetry and optimize the objective function S(i,j) (see equation 11), we chose to limit the contribution of non-canonical base pairs by assigning "0.5 hydrogen bond" for them. We have modified this phrase to "*Since PlanarFold is prone to pair any two opposite residues in the internal loop and*

bulge loop to avoid asymmetry and optimize the objective function S(i,j), we assigned 0.5 hydrogen bond to all non-canonical base pairs except for G-A."

23. On line 485, a reference should be provided for the origin of the Turner parameters. On line 589, a reference should be provided for dssr.

The related reference has been added.

24. The manuscript does not report the time cost of the simulations. Please add a table to indicate the time cost for the simulations.

We have added Figure S19 to show the time cost of the simulation with varying RNA length.

25. Typos, etc.:

Overall, the writing is clear and concise. There are some minor errors:

Line 106: "on Nussinov algorithm" -> "on the Nussinov algorithm"

Line 117: "algorothm"-> "algorithm"

Line 151: "utilized genetic algorithm" -> "utilized a genetic algorithm"

Line 474: The closing "}" is missing in equation 3.

Line 477: "lenth" -> "length"

Lines 480, 584: "paring"-> "pairing"

We sincerely thank the reviewer for identifying these typographical errors. In addition to correcting these, we have performed a full check of the manuscript and resolved all similar issues found.

Reviewer #4

The manuscript by Xiang and Xue presents an outstanding study describing the development of PlanarFold, a coarse-grained, two-dimensional molecular dynamics framework for simulating RNA structure and folding dynamics. Understanding RNA structure and dynamics is essential to elucidating RNA function, and computational approaches such as this one offer critical complements to experimental methods. In the present study, the authors focus on simulations of secondary structures and develop an efficient coarse-grained model that combines dynamic programming with molecular dynamics in 2D space. They rigorously benchmarked and demonstrated PlanarFold in several directions, including secondary structure prediction, thermodynamic stability, and kinetic properties of RNA transitions, co-transcriptional folding, and folding of large RNAs. Overall, this is a well-designed, executed, and presented study. This new platform provides a valuable and efficient approach to simulating RNA secondary structure and its transitions and will be of broad interest to the readership of Nature Communications. I highly recommend the publication of this excellent work.

I have a few minor comments for the authors to consider:

26. *The authors benchmarked PlanarFold against several established secondary structure prediction tools. However, some of the top-performing programs from recent RNA CASP challenges are not included in the comparisons. Including these methods, particularly those that have demonstrated strong performance in structure prediction, would provide a more comprehensive view of PlanarFold's relative accuracy and help situate its performance within the current landscape.*

We appreciate the reviewer's suggestion and agree that incorporating the top-performing programs from recent RNA CASP into the benchmark might be a valuable addition, even though the primary focus of RNA CASP is on 3D structure prediction. Nevertheless, several practical challenges would arise during implementation, as detailed below.

1). The participants of RNA CASP challenges include human groups and server groups. Most top-performing models in the CASP RNA challenge are generated by human groups, which use program and model selection guided by human expertise (e.g., AIchemy_RNA2, Chen, CoMMiT-human). These results cannot be obtained by fully automated programs alone.

2). The top-performing server groups (e.g., Yang-Server, Vfold) in the CASP RNA challenge rely on secondary structures predicted by dedicated secondary structure prediction programs. For instance, Vfold requires the Vfold2D program (developed by Chen's group) to provide secondary structures as restraints for the sampling. Yang-Server (TrRosettaRNA) relies on secondary structure generated by SPOT-RNA (incorporated into trRosettaRNA's pipeline). Although trRosettaRNA is reported to be able to correct some of the missing and wrong base pairs in the final predicted 3D model, the secondary structure annotated from the final structure is generally worse than SPOT-RNA's prediction. As described in the original paper of trRosettaRNA: "*Nevertheless, when considering the entire set of 30 RNAs, trRosettaRNA exhibits a slight drop in the average F1-score of secondary structures, decreasing it from 0.65 to 0.6 (as shown in Fig. S5b)*" [*Nat Commun*.2023;14(1):7266.]. Another newly developed model DRfold2 has used all of our testing data as their training data (58 out of 58), and is therefore inappropriate for comparison.

3). We evaluated a 3D structure predictor, simRNA, which is also a participant of RNA CASP, and found that it can hardly sample the near-native state of large RNAs without secondary structure restraints (Figures 6 and S17).

To further expand the scope of our benchmark, we identified a newly published deep-learning-based secondary structure predictor (BPfold) and have incorporated it into our benchmark (comparison added in Figure 2a). The secondary structure prediction performance of BPfold on our testing dataset lies between SPOT-RNA and PlanarFold, ranking third among 11 predictors we benchmarked.

27. The comparison between simulated and experimental kinetics is compelling. However, the authors currently compare only the overall exchange rate between two states. Since the overall rate can be dominated by either the forward or backward rate when the equilibrium populations are skewed, it would be more informative to report the forward and backward rates separately.

We thank the reviewer for raising this point regarding kinetic rates, which, along with Question #17 from Reviewer #3, helped us identify a mistake of formula in calculating the exchange rates from simulation. An important amendment of the kinetic rates and its related context has been made in this revision, which has decreased the speedup ratio from ~1×10^6 to ~5×10^4. Further details on these modifications are available in Response #17, which we invite the reviewer to consult.

We would also emphasize that, after correcting the formula of calculating forward, backward, and exchange rates, they remain in strong agreement with the corresponding experimental data. The forward and backward rates have been added to Table S10 in this revision, along with free energy differences. The correlation of the forward rates and backward rates with their corresponding experimental values has been analyzed in the newly added Figure S12. In general, both forward rates and backward rates agree well with NMR measurement (Pearson coefficient: 0.830 and 0.933, respectively).

These results indicate that the agreement in overall exchange rates stems from the accurate prediction of both the forward and backward rates individually, rather than from a fortuitous cancellation of errors.

Accordingly, we have revised the results section to state: "*Surprisingly, we observed excellent correlations between the overall exchange rates as well as their individual components (forward and backward rates) from PlanarFold simulations and NMR measurements (Fig. 3f and Fig. S12 and Table S10).*"

28. Given the good agreement between simulated and experimental exchange rates, it would be valuable to highlight the transition pathways derived from the simulation. In particular, can the authors identify or characterize the transition state(s) within these pathways? This would enhance the mechanistic insights provided by PlanarFold.

Transition states are high-energy configurations with an extremely short lifetime. Although PlanarFold can identify intermediates along the transition pathway in many cases, the substantial sampling acceleration it introduces to the simulation renders the transition states difficult to capture. Regarding intermediate states, for example, PlanarFold captured several intermediates of

the tri-stable RNA between its conformer A and B. Moreover, by setting a smaller bulge penalty of 0.005, our model can also capture the intermediates of T1 between its GS and ES (Figure R2).

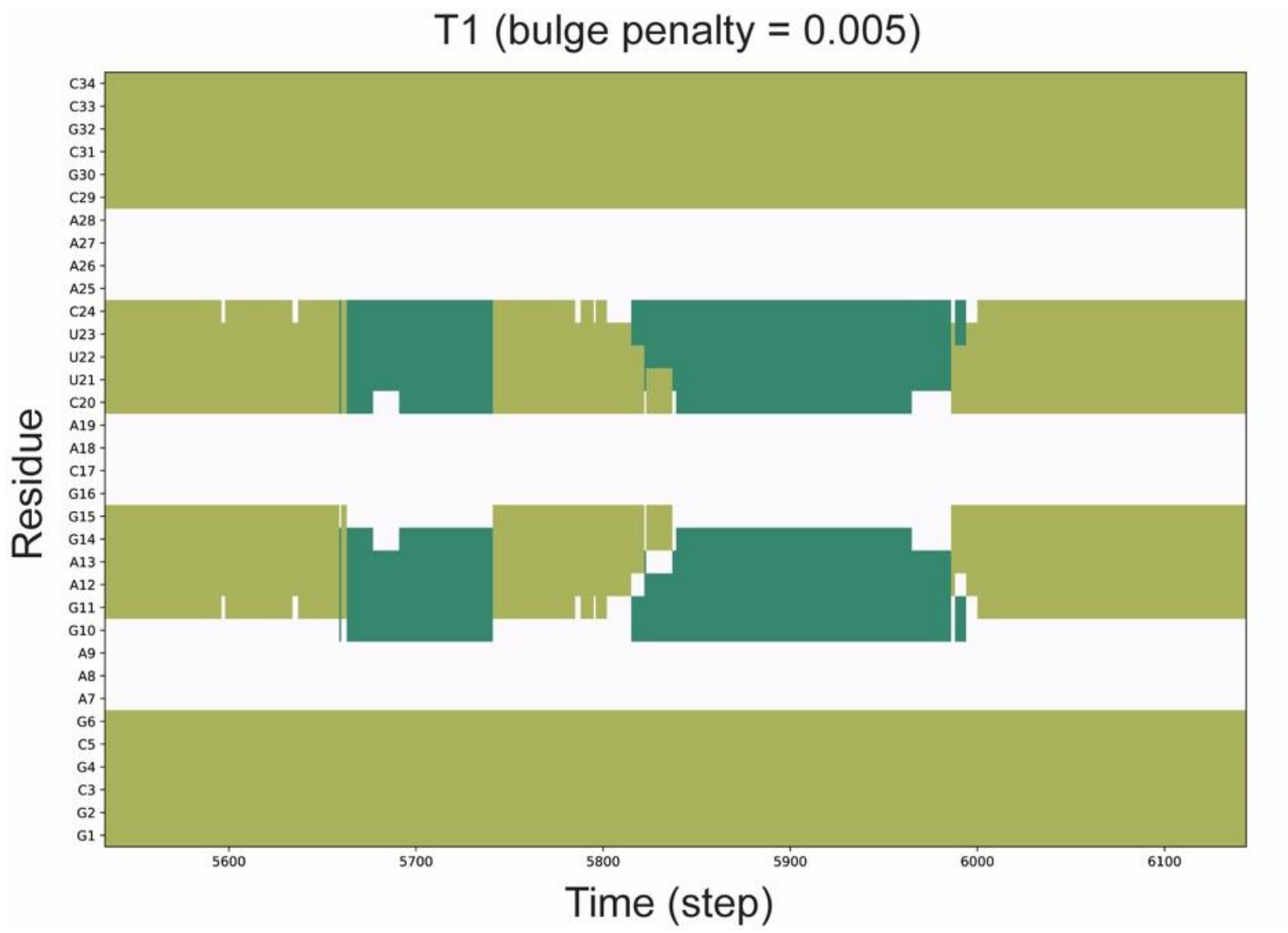

Figure R2. Stepwise transition of T1. The gradual transition by 1-nt register shift between the GS and ES of T1. Each step is 0.4 ps. The bulge penalty is set as 0.005.

Although PlanarFold cannot provide a detailed mechanistic description of the transition states, it has captured the essential factors influencing transition kinetics. We found that the exchange rate of the conformational transition is associated with the probability of forming a nucleation site for the competing conformer. For bistable-55, forming the nucleation site of conformer B within the state of conformer A (or vice versa) involves pseudoknotted base pairs (Figure S10c). The residues forming the closing base pair of conformer B are protected in conformer A, making the transition slow (on the minute timescale). For the tristable RNA, the residues forming the nucleation site of one conformer are exposed in another, making the transition easier and faster (on the second timescale) than in bistable-55 RNA. For T1-series RNA, no new hairpin needs to form; the transition occurs via a 1-nt register shift along a single strand, resulting in significantly faster (on the millisecond timescale) kinetics. The successful recapitulation of transition kinetics by PlanarFold suggests that the essential spatial proximities required for these conformational rearrangements are largely preserved in the two-dimensional representation.

29. Many biologically relevant RNAs exhibit more than two conformational states. While the authors present many two-state exchange examples, it would be helpful to include an example of a system with one dominant state and multiple alternative states. This would showcase PlanarFold's ability to capture complex folding landscapes and the kinetics of multi-state transitions.

We thank the reviewer for raising this good point. Since PlanarFold is a tool designed to study secondary structure dynamics, its analysis of transitions is confined to the secondary structure level. Specifically, PlanarFold is suitable for characterizing the transition involving changes in A-U, G-C, and G-U base pairs. We have searched the literature for multi-state transition studies and identified a few examples; however, virtually none are appropriate for investigation with PlanarFold. These cases fall into three categories [*Cold Spring Harb Perspect Biol*. 2020,12(1):a032425; *Mol Cell*.2018, 70(2):274-286.e7]: 1) transitions only involving minor changes in tertiary interactions or

structure; 2) transitions induced by binding with ligands, proteins, or other molecules; and 3) transitions involving conformational changes of only non-canonical base pairs. Additionally, transitions with excessively large conformational changes in long RNAs that exceed the upper limit of PlanarFold's simulation time are also unsuitable.

The only suitable case we found is a tristable RNA with three interconverting states, which has already been investigated in the current work. However, the prior study only characterized the exchange between two of its three states (conformer A and B, Figure S10e). The exchange between conformer A and B with the third state (conformer C) is estimated to be at least one order of magnitude slower than the exchange of conformer A and B. We attempted to simulate the transition of conformer A or B to conformer C by extending the simulation time, but we did not observe the appearance of conformer C even after 2000 μs of simulation at 40 ºC. We also launched the simulation using conformer C as the starting conformation, but it rapidly transitioned to conformer A or B and never reverted (Figure R3). This instability of conformer C in our simulations may stem from PlanarFold's current inability to account for non-canonical base pairing within it. Incorporating non-canonical base pairing into PlanarFold has been discussed in the *Discussion*.

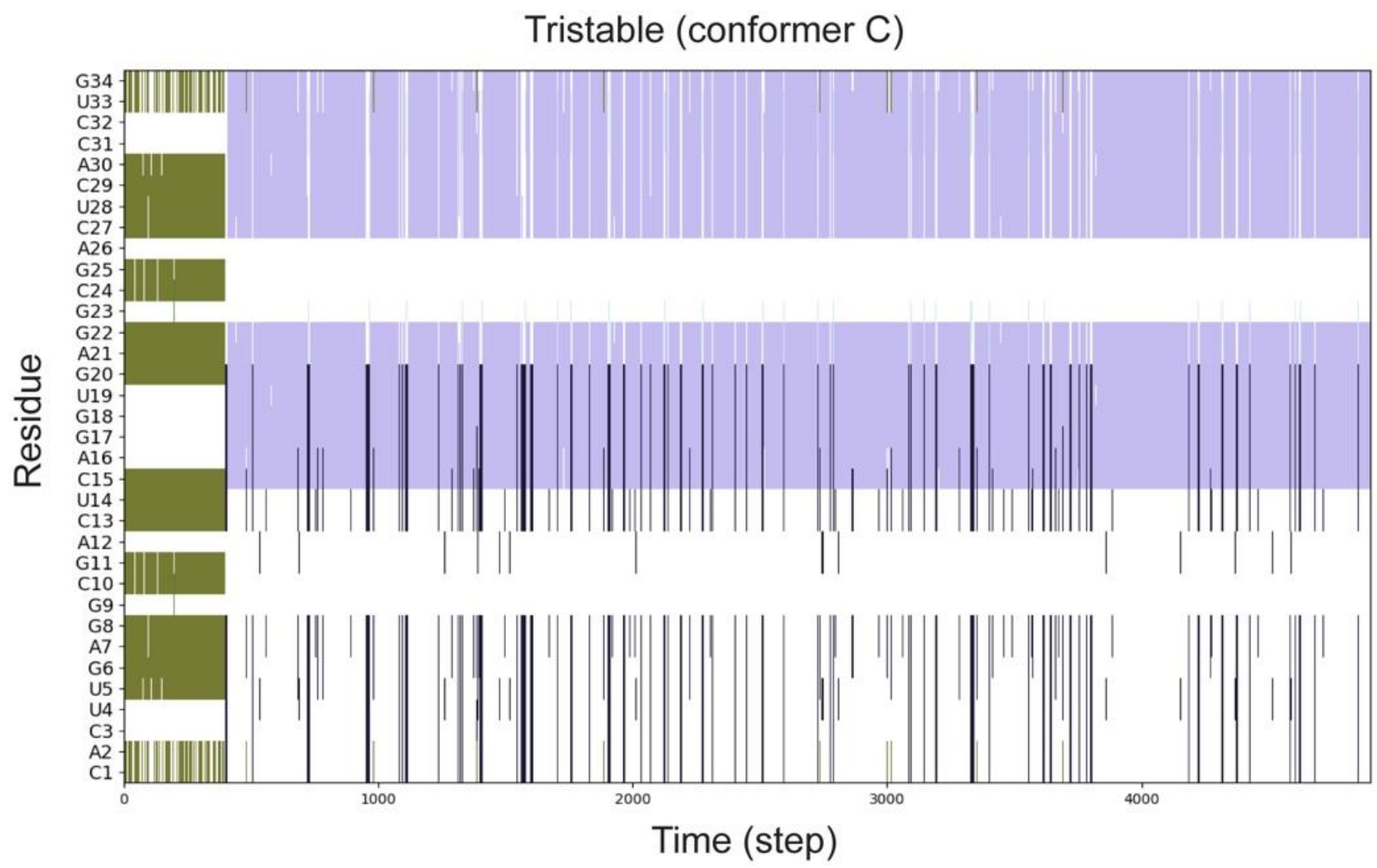

Figure R3. Transition from state C to state A/B of tri-stable RNA.

30. In Figure 5d, the authors discussed misfolded states of the p5ab RNA with and without force. To improve clarity, it would be helpful to include the secondary structures of these misfolded conformations.

As suggested, representative structures of the misfolded states (IM, M) have been included in Figure 5d, and the following sentence has been added to the caption: "*In the lower panel, two kinetically trapped states are denoted as M and IM, and the representative structures of F, IM, and M are displayed.*"

The previously labeled misfolded states M1 and M2 are renamed as IM and M, respectively. The state labeled IM represents an intermediate with a misfolded region, featuring one domain in the native state (F) and another in the misfolded state (M). We have reclassified it as an intermediate because it eventually folds into the native state (F) in all simulations (each 2 μs in length). It should be noted that the misfolded state (M) represents not a single conformer but an ensemble of

structurally similar conformers. Although their secondary structures exhibit minor variations, their contact maps are highly similar, which accounts for their clustering in the principal component analysis (Figure 5d, lower).

We sincerely thank the reviewers for their comments and suggestions again, which have led us refine our manuscript. During this revision, we have performed several additional simulations and analyses for pseudoknot RNAs. More detailed discussion of pseudoknot simulations is made in the Discussion of the manuscript as well as in Response #2 and #3.

Reviewer #1

1. *Response to Question 3: The justification for fine-tuning the model on NMR-derived data is unconvincing. The authors responded that the NMR-derived data used to fine-tune the model "essentially serve the same role as the in vitro protein stability data" used to calibrate BioEmu; however, BioEmu was fine-tuned on a large experimental data (~ 500k), whereas PlanarFold was refined using only a small set of hairpin RNAs, making the two incomparable in scale and representativeness.*

The context we mentioned BioEmu as comparison is that, training a model capable of predicting alternative states (and the free energy differences between them) solely based on low-energy state conformations from PDB structures is fundamentally impractical; therefore, it is necessary to "feed" the model thermodynamics-related knowledge and information. For the BioEmu model, the thermodynamic input is protein stability data, while for PlanarFold, the thermodynamic input is NMR-derived data. In this sense, both aim to equip the model with the ability to predict thermodynamic properties. Our original statement, "The NMR-derived data we used to fine-tune PlanarFold essentially serve as the same role as the in vitro protein stability data they employed to fine-tune BioEmu, to impart thermodynamic knowledge to the model," may have been imprecise. What we intended to express was: "In terms of imparting thermodynamic knowledge to the model, the NMR-derived data we used to fine-tune PlanarFold essentially serve the same role as the in vitro protein stability data they employed to fine-tune BioEmu."

More specifically, the way BioEmu uses protein stability data, however, differs from how PlanarFold uses NMR-derived data. For deep learning models like BioEmu, which have millions of parameters (specifically, 31 million), a vast amount of thermodynamic data (such as ~ 500 K) is required to guide model fitting and parameter adjustment. In contrast, PlanarFold's potential energy function (or scoring function) is a very simple physical potential with very few parameters (less than 50) that are intuitive in meaning (e.g. base pairing, base stacking). Moreover, after genetic algorithm optimization, we use NMR-derived data to adjust only one parameter, namely $f_{scaling}$ (Equation 1, Fig.S9b), not millions of parameters, so consequently, a large amount of data is not necessary.

It is worth emphasizing that NMR methods offer a unique advantage: relaxation dispersion techniques enable the direct measurement of energy differences between alternative states. Our NMR-derived data consist precisely of such experimental measurements. The implementation of this fine-tuning involves a global rescaling of the potential energy (Equation 1), without any further adjustments to individual energy terms in the potential function. Consequently, during this step of fine-tuning, the relative proportions among the various energy terms remain unchanged, as their parameters have already been optimized through the previous GA steps. Using NMR-derived data, we essentially adjust the overall offset of the predicted ΔΔG relative to the experimental ΔΔG. The complexity of this parameter optimization task is significantly lower than fine-tuning tens of millions

(31 M) of parameters in a deep learning model like BioEmu, which explains the substantial difference in the amount of data required.

In fact, RNAstructure's ability to predict the free energy of different secondary structures and their differences also stems from its utilization of thermodynamic data, which are obtained from UV melting experiments on RNA duplexes. Specifically, they employed only 90 RNA UV melting datasets to fit 24 model parameters (Nearest Neighbor parameters [https://pubs.acs.org/doi/10.1021/bi9809425]). Their model is a physically more interpretable scoring function rather than being deep-learning-based, and numerous secondary structure prediction benchmarks have demonstrated that RNAstructure's model parameters possess good generalization capability. Similarly, PlanarFold also uses only NMR-derived data from a few simple hairpins to adjust only one parameter ($f_{scaling}$). Its concise physical potential also exhibits a certain degree of generalization ability. This generalization capability is further evidenced in our current revision by the satisfactory prediction performance on several pseudoknot RNAs (PlanarFold achieved good prediction results on 15 pseudoknot RNAs that were previously completely untested and unfitted).

In terms of results, after fine-tuning with NMR data from just a few simple hairpin RNAs (T1-series), we achieved satisfactory performance in predicting free energy differences for subsequent bistable RNAs and other RNAs (Fig.S10g). This also verifies that models based on physical potentials generally exhibit stronger generalization capabilities and lower risks of overfitting.

2. *Response to Question 5: The authors justify excluding PKs and non-canonical pairs largely by arguing they are rare and hard to model, and by pointing to poor performance of predicting PK/noncanonical base pairs by other models. The authors responded that PK formation "more closely resembles tertiary interactions" and is "unlikely to form on a two-dimensional plane". This is an oversimplification. Pseudoknots are commonly treated as secondary structure by many methods and can be readily represented in 2D contact maps.*

We are not attempting to evade the issue by "justifying excluding PKs and non-canonical pairs largely by arguing they are rare and hard to model, and by pointing to poor performance of predicting PK/noncanonical base pairs by other models." Rather, we initially believed that the manuscript had already made it clear in Fig. S1a ("The pipeline of collecting the TR and TS datasets. The TR and TS dataset are obtained by filtering RNAs of high pairing ratio (>40%), low nested pairing ratio (<10%), and of diverse topologies.") and also in the Discussion that PlanarFold cannot predict pseudoknots or non-canonical pairs. Therefore, we were uncertain whether the reviewer's question referred to PlanarFold's performance in predicting PKs/non-canonical pairs, or to the current state of PK/non-canonical prediction in the broader field of RNA secondary structure prediction. Consequently, we addressed both aspects separately—namely, PlanarFold's capability regarding PK/non-canonical prediction and the current status of PK/non-canonical prediction in the field. Unfortunately, our intended message was not conveyed clearly, and we should have explicitly stated in that passage that these two parts were meant to address two possible interpretations of the question.

Our full response was: "Although pseudoknots (PKs) can be predicted as secondary structure motifs for some models, their formation more closely resembles tertiary interactions and are unlikely to form on a two-dimensional plane." Taken out of context, the latter part of the sentence appears misleading. Moreover, the phrase "pseudoknots (PKs) can be predicted as secondary structure motifs for some models" indicates that we also acknowledge the field-wide consensus

that pseudoknots are considered secondary structure. However, unlike conventional dynamic programming approaches that directly predict secondary structure, we predict secondary structure by performing dynamic programming using coordinate information derived from planar MD simulations. There is a substantial difference between these two approaches in how they handle PKs (and traditional DP algorithms also face great difficulties in handling PKs).

We agree with the statement “Pseudoknots are commonly treated as secondary structure by many methods and can be readily represented in 2D contact maps.” However, our discussion pertains to **whether pseudoknots can be represented in the “plane”**, not **whether they can be represented in the “contact map”**. **“Planar representation”** and “**representation in a contact map”** are two entirely different concepts. Both tertiary and secondary interactions can be represented on a contact map, as long as they are close enough to form a “contact”. What we stated before is that many long-range pseudoknotted base pairs are impossible to be close enough in the first place to form a “contact” in the plane, and therefore they certainly cannot appear in the contact map (see newly added Fig. S19c). Although many pseudoknot RNAs are displayed on a two-dimensional plane, they cannot be drawn in close proximity like nested stems; instead, pseudoknot stems require elongated lines to be drawn manually to connect them.

"This figure has been redacted because it contains elements sourced from another publication. Ple ase see Figure 2 in Staple DW, Butcher SE. Pseudoknots: RNA structures with diverse functions. P Lo S Biol. 2005 Jun;3(6):e213. doi: 10.1371/journal.pbio.0030213. Epub 2005 Jun 14. PMID: 15941 36 0; PMCID: PMC1149493."

What we intended to convey is that “the formation of PKs more closely resembles that of tertiary interactions (because they are long-range interactions and, unlike nested stems, do not form readily)”. Hence, the RNAs studied in this work either contain no pseudoknots or have a low PK content. Additionally, not all PKs are long-range; some short-range PKs, as we have verified, can indeed form (newly added Fig. S19). Therefore, in this revision, we have modified the PlanarFold code by introducing a switch to control whether PK formation is allowed. By enabling this switch (iPK = 1), we selected 15 PK-containing RNAs to benchmark secondary structure prediction performance. PlanarFold achieves remarkable prediction performance on these short PK RNAs, significantly outperforming traditional methods in this task, which further demonstrates the strong generalization capability of the PlanarFold force field.

As for why non-canonical base pairs are not currently considered, we have supplemented the Supplementary methodsSupplementary methods with the following explanation: “The non-canonical base pairs are only used to calculate S(i,j) but not used to draw the final secondary structure. Due to the lack of their experimentally derived stacking and pairing parameters, but also their complexity and unpredictability in internal and bulge loops. For instance, all symmetric N×N (e.g. 1x1, 2x2) and asymmetric N×M (N<M, e.g, 1×2, 3×4) internal loop will form N non-canonical base pair if non-canonical base pairing is considered in PlanarFold. This might be true for some short symmetric internal loops such as 1×1 and 2×2. Nonetheless, based on PDB statistics, many

larger symmetric N×N (N>2) and asymmetric N×M (N<M) internal loops do not form N continuous base pairing as short internal loops do. Instead, residues in those larger internal loop form intricate base pairs and stacks networks, and some residues are unpaired or facing outward the helix. Hence, only AU, GC, and GU base pairs are allowed to calculated base-pairing, base-stacking, and stem-angle potential terms, to avoid the aforementioned complications caused by considering non-canonical base pairs

3. *Furthermore, PKs can substantially alter RNA thermodynamics, kinetics, and folding pathways, particularly for larger RNAs, so their exclusion is a major limitation for a dynamic simulation tool. Excluding PKs undermines the model's applicability to many biologically relevant RNAs and should be clearly acknowledged as a key limitation in the manuscript.*

First, we agree with the reviewer that pseudoknots (PKs) have a substantial impact on various dynamic properties of RNAs, and excluding PKs from simulations would limit PlanarFold's applicability to many functional RNAs. Although, many natural RNAs (e.g., RNase P, riboswitches) do contain pseudoknotted base pairs, as long as the overall PK content remains low (<10%), PlanarFold can still be effectively applied to study the formation of the dominant nested secondary structure in these RNAs, since their PKs typically form after the core secondary structure has already been established. The Discussion section has been expanded to include a more detailed treatment of PK RNA (pseudoknot RNA) simulations.

Our primary research focus is on nested secondary structures and their associated dynamic properties. For many large RNAs, the majority of PKs are long-range interactions that typically form after the basic secondary structure has already been established—for example, the kissing loop in the purine riboswitch.

In our previous Discussion, we mentioned that PlanarFold is currently not suitable for studying PKs, but this statement was insufficiently elaborated. In this revision, we have taken two major steps: first, we modified the source code by introducing a switch to enable PK formation, and second, we performed additional simulations on multiple PK-containing RNAs to examine the impact of allowing PK formation on both PK and non-PK RNAs. The results are summarized as follows:

I). New function implementation of PK formation. Our previous DP code contain a module that explicitly disallowed pseudoknots. We have now introduced a control parameter (iPK). When iPK = 0, PK formation is prohibited (the default setting used in all previous simulations). When iPK = 1, PK formation is allowed. All supplementary simulations allowing PK formation in this revision were conducted with iPK = 1.

II). Secondary structure prediction of PK RNAs. We selected 15 PK-containing RNAs with high-resolution PDB structures and performed secondary structure prediction using PlanarFold. This benchmark is presented in the newly added Figure S19. Notably, none of these 15 RNAs are present in our training set (or testing set), and PlanarFold has never been trained on pseudoknotted base pair prediction. Therefore, this benchmark serves as an excellent test set for evaluating the generalization capability of the PlanarFold force field. We observed that for short PK RNAs with short-range pseudoknotted base pairs, PlanarFold achieves remarkable prediction performance, significantly outperforming traditional methods such as RNAstructure and IPknot, and even reaching the training performance of deep-learning-based methods like SPOT-RNA and UFold. However, for larger RNAs such as 1SJ3 and 5JUP, certain pseudoknotted stems cannot be

sampled or predicted by PlanarFold because they are geometrically inaccessible in the plane. For longer PK RNAs, the majority of pseudoknotted base pairs are long-range and thus cannot be simulated by PlanarFold. Of note, those RNAs containing long-range PKs are also challenging for other software packages: all five benchmarked methods exhibited substantially lower F1-scores on 1SJ3 and 5JUP compared to simple PK RNAs.

III). Thermodynamics and kinetics. We examined the effect of allowing PK formation (iPK = 1) on conformational switching of the bistable RNA studied previously. After enabling PK formation, the exchange rate of Bistable55 RNA at 293 K increased from 0.399 μs-1 to 3.608 μs-1—an approximately 9-fold increase—and the free energy difference decreased from 0.64 kcal/mol to 0.39 kcal/mol. These results indicate that allowing PK formation does influence the predicted free energy differences and kinetic rates of bistable RNAs. However, we have previously noted that kinetic rates are subject to approximately one order of magnitude uncertainty; the kinetic rate obtained under the default PK-free setting was likely an underestimate. Moreover, the free energy difference of 0.39 kcal/mol indicates that this bistable RNA still populates two conformations with comparable probabilities when PK formation is permitted. For the T1 series of RNAs (involving 1-nt register shifts), enabling PK formation had no impact, as their transitions do not involve PK formation.

"This figure has been redacted because it was sourced from another publication. Please see Figure 4 in Szyjka CE, Strobel EJ. Observation of coordinated RNA folding events by systematic cotranscriptional RNA structure probing. Nat Commun. 2023 Nov 29;14(1):7839. doi: 10.1038/s41467-023-43395-9. PMID: 38030633; PMCID: PMC10687018."

IV). Co-transcriptional folding. We performed co-transcriptional folding simulations of a fluoride riboswitch, which is not studied before as it contains pseudoknot. Experimental studies on this riboswitch have demonstrated that, in the absence of fluoride ions, the preformed PK1 is progressively replaced by the terminator stem [Nat Commun. 2023.29;14(1):7839]. Our simulations successfully recapitulated this strand displacement phenomenon. However, due to planar geometric constraints, PK1 appeared less stable in our simulations; typically, only one of the two competing helices (PK1 or P1) remained stably formed within the 47–67 nt region. This again reflects the difficulty of simulating certain pseudoknotted stems in a planar representation. In addition, we performed co-transcriptional folding simulations of the reverse riboswitch at a pulling rate of 125 nt/μs with PK formation enabled, and repeated the simulation 40 times. Among these replicates, only 3 trajectories yielded the branched conformer (Pa+Pb) as the final product, while the remaining 37 trajectories resulted in the rod-like conformer (Pc+Pd). Compared to our previous PK-free simulations (where the branched:rod-like ratio was 6:34), the proportion of branched conformers decreased from 15% to 7.5%. This reduction is attributable to the fact that allowing PK formation increases the probability of switching from Pb to Pc during transcription, thereby

increasing the population of the rod-like conformer. Nevertheless, this change remains within an acceptable range and does not lead to the complete disappearance of the branched conformer.

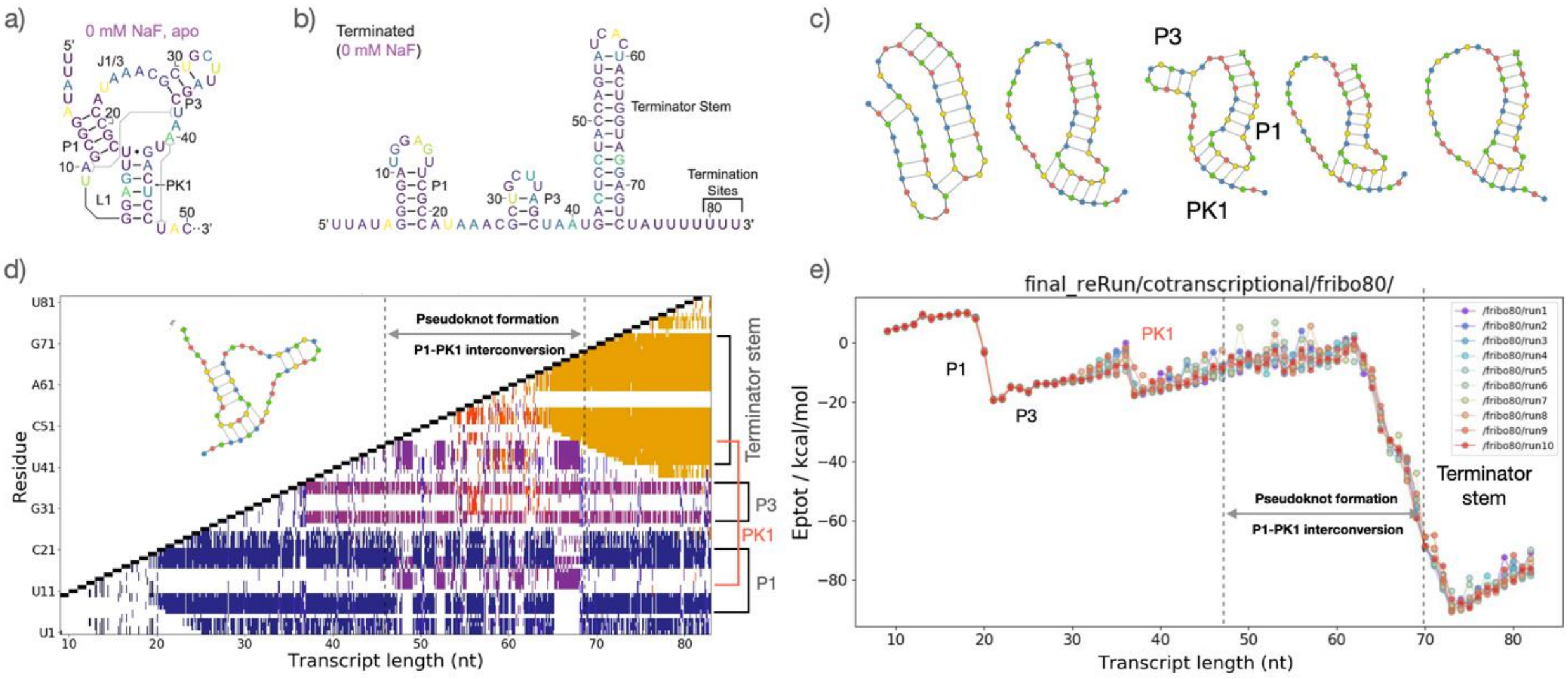

Figure R3. Co-transcriptional folding of a fluoride riboswitch

a The conformation of the 50-nt transcript of fluoride riboswitch without fluoride binding. **b** The conformation of the 82-nt transcript of fluoride riboswitch without fluoride binding. [Nat Commun. 2023.29;14(1):7839] **c** Some pseudoknotted conformations sampled by PlanarFold during co-transcriptional folding simulation. **d** The secondary structure evolvement during co-transcriptional folding. During 47-73 nt window, the P1 and PK1 is competing with each other. **e** The potential energy evolvement during co-transcriptional folding. Ten repeats are displayed.

V). *De novo* folding of large RNAs. We also performed *de novo* folding simulations of two large RNAs, 1GID and 2QBZ, with PK formation enabled. Under force-free conditions (iPK=1), the successful folding rates for 2QBZ and 1GID are 5/10 and 8/10, respectively. In our previous PK-free simulations (iPK=0), the success rates for 2QBZ and 1GID were 5/10 and 5/10, respectively (Figure 6b and S18b). These results indicate that allowing PK formation does not compromise PlanarFold's folding ability for these two large RNAs. Furthermore, we performed mechanical refolding simulations of 1GID with PK formation enabled; under external force, 1GID achieved a 10/10 successful folding rate, identical to the 100% success rate observed under PK-free conditions.

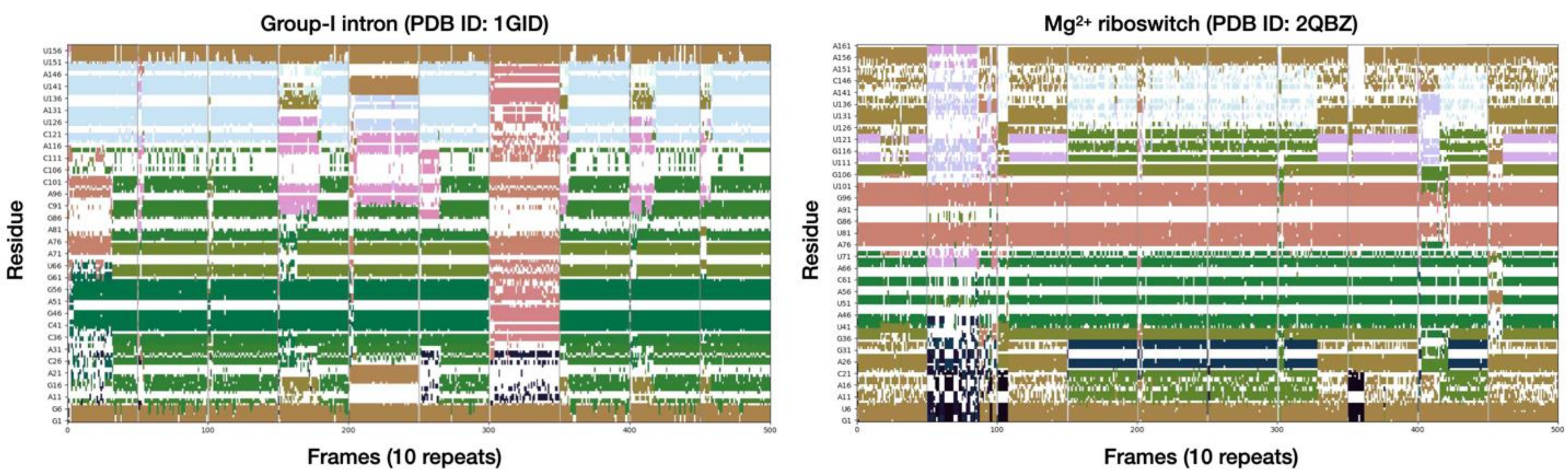

Figure R4. *De novo* folding of two large RNAs

In summary, PlanarFold is indeed incapable of simulating many long-range pseudoknotted stems due to its intrinsic 2D planar constraint. However, it demonstrates strong predictive performance for short-range pseudoknotted stems. In this revision, we have introduced a user-controllable option to enable PK simulation. Nevertheless, owing to the geometric limitations of planar representation, many long-range PKs remain inaccessible even when iPK = 1. We have verified that PlanarFold

achieves excellent secondary structure prediction accuracy for short PK-containing RNAs. For non-PK RNAs, allowing PK formation in MD simulations has measurable effects on conformational exchange thermodynamics, kinetics, and folding pathways, but these effects do not markedly alter the overall simulation outcomes or change our major conclusions. Given that our parameter optimization and refinement were conducted exclusively on PK-free RNAs, PlanarFold is still recommended primarily for studying the dynamic properties of RNAs with low pseudoknotted base pair content. In the future, we plan to further refine the force field parameters based on short PK RNAs to expand PlanarFold's applicability and predictive capacity. Concurrently, we are actively developing a 3D version of our CGMD simulation software, as we believe that PK RNAs are more appropriately studied using 3D CGMD rather than the 2D PlanarFold framework.

4. *Other issues: Some computational details are still missing (e.g., simulation seed for modeling E.Coli SRP). Full method/simulation details and more rigorous benchmarking are needed to support publication.*

Given the word limits of the main text and Methods section, and the extensive range of applications involved in this work, it is difficult to present all details in full within the main text or methods. Therefore, more specific MD simulation details have been added to the Supplementary methods. We have refined the three previously existing sections (Force field of PlanarFold, Molecular dynamics simulation: general parameters, Dynamic programming) and newly added four methodological sections: MD simulations for predicting secondary structures, MD simulations for predicting free energy differences and kinetic rates, MD simulations for co-transcriptional folding, and MD simulations for mechanical unfolding and refolding, to better assist others in reproducing the results of this paper. In addition, we have added Figure 1a and Figure S1 to better illustrate the design workflow of the PlanarFold model. However, since this MD tool involves many control parameters, we still recommend that users refer to the User Manual for more detailed simulation guidance.

Regarding the co-transcriptional folding simulations of E. coli SRP, providing the random seed for the simulations is unnecessary because the random seed in our simulations is determined by the system clock. Moreover, to validate the results of the E. coli SRP co-transcriptional folding simulations, it is less instructive to reproduce simulations that are exactly identical to our 10 trajectories. Instead, it is more meaningful that 10 simulations initiated with arbitrarily chosen random seeds should yield statistical results similar to those of our 10 trajectories; otherwise, it would indicate insufficient sampling in the MD simulations. This is also one of the main reasons why almost all current MD simulation articles do not provide random seeds in their methods sections.

Reviewer #2

I co-reviewed this manuscript with one of the reviewers who provided the listed reports. This is part of the Nature Communications initiative to facilitate training in peer review and to provide appropriate recognition for Early Career Researchers who co-review manuscripts.

Reviewer #3

The revision of "PlanarFold: a coarse-grained molecular dynamics model of RNA in two-dimensional space," by Xiang and Xue, addresses at least some of my concerns. Overall, I am convinced that the work is novel and interesting. I would still like all the details of the implementation provided in the main text or supplement (rather than stated in a code manual or only in code).

My principal concern with the prior manuscript was that the testing sets were contaminated with homologs of the training data. This version of the manuscript adds an additional benchmark that directly addresses this concern. This is presented on page 7 (lines 170-173) and in Figures S5e and S6. This benchmark, which is capable of assessing transferability to unseen RNA structures, shows that PlanarFold performs about as well as traditional thermodynamic methods (which is impressive). The machine learning methods cannot be assessed on this benchmark without retraining.

5. *The current manuscript still presents the contaminated benchmark up front with claims that PlanarFold and machine learning methods outperform traditional methods. On the one hand, I see that PlanarFold cannot be benchmarked fairly against machine learning methods without retraining them on the same training and testing sets. But the presentation does not clarify that this benchmark is contaminated (and a poor representation of transferability). In my opinion, this benchmark can be included, but should be the one shown in the supplement. At the least, the problems with contamination need to be explained in the main text.*

We have replaced the results with Figure 2b to demonstrate the cross-sequence and cross-family performance of PlanarFold.

However, we'd like to clarify that our cross-sequence validation in Fig. 2a was conducted using a testing dataset (TS) collected according to the widely adopted filtering standard in the field and also by the DL-based methods benchmarked in this study (80% is also the minimum cutoff allowed by the CD-HIT-EST software). We believe this benchmark aligns with the evaluation criteria commonly used in the field. The revised main text reads as follows:

"For an unbiased comparison, we first gathered all the RNA sequences that have been used as training data for SPOT-RNA, MXfold2, and UFold, and merged them into a large dataset (approximately 40,000 sequences, Table S1), termed DL-nonRedundant. From this dataset, we selected 216 RNAs with high-resolution PDB structures as the training set (TR). Then we carefully assembled a testing set of newly released, high-resolution RNA PDB structure. These RNAs were further filtered by removing those with ⩾80% sequence similarity to the DL-nonRedundant dataset (Fig. S1a), yielding a final set of 58 RNAs (TS) that can serve as an independent cross-sequence testing dataset for these DL-based predictors."

As the reviewer pointed out, although we did not retrain the machine learning methods, we strictly followed the same testing dataset selection criteria used by DL-based tools to obtain a testing dataset with less than 80% sequence similarity to their training datasets. The TS dataset we

curated is entirely equivalent to and independent of the testing datasets used in their respective studies.

For example, in the case of SPOT-RNA, they first selected TR0, VL0, and TS0 from bpRNA, and then further obtained TR1, VL1, TS1, and TS2 from the PDB (as of March 2, 2019) (Table S1). In their paper, they used TR0, VL0, TR1, and VL1 for parameter tuning and ensured that the RNAs in TS1 shared less than 80% sequence similarity to those in TR0 and TR1. They reported the prediction performance on TS1 as the test performance of their tool. For PlanarFold, we collected all RNA sequences used by SPOT-RNA (including TR0, VL0, TS0, TR1, VL1, TS1, and TS2), as well as the training data from the other two deep learning models, to form a large non-redundant dataset called DL-nonRedundant. We then selected our TS from the latest PDB entries based on the criterion of "less than 80% sequence similarity to any RNA in the DL-nonRedundant dataset", which means that every RNA in our TS shares less than 80% sequence similarity to any RNA in the training sets of SPOT-RNA (as well as UFold and MXfold2). Now that SPOT-RNA used its TS1 to demonstrate its predictive performance, our TS dataset following the same filtering standard can fully serve as an independent and valid test set for evaluating the cross-sequence prediction performance of SPOT-RNA. Similarly, because our DL-nonRedundant also includes all training data used by MXfold2 and UFold, TS is equally valid as a cross-sequence test set for these predictors. As for BPfold, since it did not create its own new dataset but entirely adopted the datasets from SPOT-RNA, MXfold2, and UFold, our TS is also an effective and independent dataset for evaluating BPfold.

Additionally, from a practical perspective, current deep learning–based predictors still perform reasonably well on RNAs from known families, so their intra-family prediction retains practical value. The performance of deep learning predictors on tasks involving cross-family prediction is indeed concerning, however, it is not what can be solved by cross-seqeunce validation.

6. *This revision does a better job explaining how things work in PlanarFold, but I am still not satisfied that enough of the underlying methods are explained to fully evaluate the implementation. This version addressed my concerns about timestep, mass, and thermostat choices. But those were just examples, and I am still wondering about how the engine integrates the equations of motion (e.g. verlet, leapfrog, etc.) and, very importantly, how initial velocities are chosen for the simulations. Also, because Berendsen thermostat is used, are rotational and translation motions removed from the simulation? If not, those motions could use much of the kinetic energy.*

Given the word limits of the main text and methods section, and considering the wide range of applications involved in this work, it is difficult to fully present all details in the main text or Methods. Therefore, more specific details of the MD simulations have been added to the Supplementary methods. We have refined the three existing sections (Force field of PlanarFold, Molecular dynamics simulation: general parameters, Dynamic programming) and newly added four methodological sections: MD simulations for predicting secondary structures, MD simulations for predicting free energy differences and kinetic rates, MD simulations for co-transcriptional folding, and MD simulations for mechanical unfolding and refolding, to better assist others in reproducing the results of this paper. Moreover, Figure 1a and Figure S1 are added to the main text and supporting materials to better illustrate the pipeline of PlanarFold's model. However, since this MD tool involves many control parameters, we still recommend users refer to the User Manual for more detailed simulation guidance.

Since we adopted the same fundamental framework and many file formats as AMBER, we also use the same Verlet algorithm as AMBER: modified velocity Verlet integration. The formulas first update the half-step positions and full-step velocities （$V(t+\frac{1}{2}\Delta t)=V(t)+a(t)\cdot\frac{1}{2}\cdot\Delta t$, $X(t+\Delta t)=X(t)+V(t+\frac{1}{2}\Delta t)\cdot\Delta t$）, then compute energy and gradients, and finally update the full-step positions ($V(t+\Delta t)=V(t+\frac{1}{2}\Delta t)+a(t+\Delta t)\cdot\frac{1}{2}\cdot\Delta t$). It is called "modified" velocity Verlet integration because, unlike the traditional velocity Verlet integration, our algorithm explicitly calculates the half-step velocity, resembling a hybrid of leapfrog Verlet and velocity Verlet integration methods, following AMBER's framework. It is worth mentioning that PlanarFold was not written from scratch by imitating AMBER. Before developing PlanarFold, we have developed an all-atom simulation software in C based on the code framework of AMBER's mdgx program for in-house educational use only (with only basic functionality implemented), and our calculation results were highly consistent with AMBER's programs (pmemd and sander). PlanarFold was developed upon this all-atom simulation software by removing redundant modules and functions, and adding new potential energy functions and functional modules (such as DP). Therefore, the MD framework of PlanarFold is highly consistent with AMBER, and its input/output files as well as the naming of many variables follow AMBER's format.

Regarding the selection of initial velocities, velocity initialization is far less critical for coarse-grained simulations than for all-atom simulations. Sampling in all-atom simulations is highly dependent on the starting structure, while for our coarse-grained simulations, the influence of the starting structure and velocities is minimal, for two main reasons. First, we chose a very small temperature coupling constant (5 fs), so PlanarFold uses very tight heating bath. The reason for choosing such a tight thermostat coupling constant is that we found the temperature fluctuations in CGMD with the same coupling constant are much higher than in all-atom simulations. After testing a series of coupling constants, we found that 5 fs allows the temperature fluctuations in our CGMD to approximate those in all-atom MD simulation systems, a detail often overlooked by many other CGMD software packages.

Second, while the starting structure is very important in all-atom MD simulations as it determines whether certain low-frequency/slow conformational changes can be accessed, for our CGMD, the initial structure and velocities are almost negligible relative to the scale of the conformational changes we aim to study. For example, for T1, as long as the starting conformation does not have beads too close or overlapping, simulations starting from any initial conformation and velocities at 283 K can capture both its GS and ES, and the ratio between ES and GS remains essentially consistent over 1 µs of simulation (Fig.3e). Multiple parallel repeats in Figure 3e, 4b, 5c, 5f, 5g, and 6b can serve as direct evidence. Therefore, initializing velocities according to the Boltzmann distribution is unnecessary for our coarse-grained simulations because, regardless of the initial velocities, the thermostat quickly adjusts them to the user-set simulation temperature.

As for removing translational and rotational momentum, we already stated in the Supplementary methods ("MD simulation" section) previously: "Translation and rotational momentum of the system are removed every 1000 steps." This removal frequency ensures the system does not develop significant translational or rotational motion.

7. *I still do not find an explanation as to how the contact map is divided into independent regions. This is mentioned as something that is done on lines 511-512 on page 18. In my opinion, it would be better to let the dynamic programming algorithm address this by letting it run on the full length sequence and full contact map, allowing for branching.*

In lines 512-517 of the main text methods, we mentioned that the DP algorithm of PlanarFold first divides the entire RNA into several sub-regions and then performs independent DP calculations for each. Due to the word limit of the methods section, more details of contact map division were stated in the DP method module in Supplementary methods, which has now further been refined as: "2. Select the stem blocks according to contact map. Only the upper-triangular part of the map is used for this process given the symmetry of the contact map. We employed a recursive algorithm to search for the neighboring contact of each contact to determine the independent 'block'. For each contact in the contact map, this algorithm searches its neighboring valid contacts recursively. Therefore, each recursive search will identify a continuous block of contacts, which suggests an individual helix region. As indicated in Figure.1b, there are 4 blocks, hence 4 helix regions (R1-R4) will be identified by this recursive search. By splitting the contact map into sub-matrices, the computationally heavy enumeration of bifurcated helices ('branching') is also avoided in dynamic programming. Moreover, independent calculation of each region is also designed to facilitate future GPU parallelization."

Additionally, the reasons why we did not initially consider performing DP calculations with branching over the entire contact map are as follows. First, for the Nussinov algorithm, even if branching occurs, it does not assign any bonus/penalty to branching—unlike Zuker's MFE algorithm, which applies different free energy bonuses/penalties to different multi-way junctions. Therefore, in our DP algorithm based on Nussinov, determining the specific type of branching is pointless. On the other hand, if branching were allowed, the complexity would increase from $O(N^2)$ (without considering branching) to $O(N^3)$ (since each recursion would require additional computation of possible branches), which would be a significant overhead for our MD simulation software that performs dynamic programming calculations at each integration step.

However, it is worth discussing that although PlanarFold avoided the computational cost of branching, it also introduced additional overhead for dividing the contact map into blocks. Yet, dividing into blocks also reduces the $O(N^2)$ complexity to computing m smaller blocks, i.e., $O(N^2/m)$. Therefore, we are currently uncertain whether considering branching would improve computational efficiency. In terms of determining secondary structure results, there is no difference between the two approaches unless a residue-i belongs to two blocks simultaneously (we have already addressed how to handle such cases in the previous round of revisions). In the future, our DP development will involve testing the differences in computational efficiency and junction structures between "DP with branching considered" and "block-partitioned DP".

8. *The dynamic programming algorithm recursions (equation 11) include: max[S(i+1,j-1)+Cmap(i,j), S(i+1,j)+Cmap(i,j)+P, S(i,j-1)+Cmap(i,j)+P]*
   but I think they should state:
   max[S(i+1,j-1)+Cmap(i,j), S(i+1,j) +P, S(i,j-1) +P]
   If Cmap(i,j) is the score of a base pair, then only the first option should include that score because that is the state of adding a base pair between i and j. The second or third options are adding unpaired i or unpaired j nucleotides, respectively.

We sincerely thank the reviewer for pointing out this error in the formula. We apologize for the mistakes in describing the DP algorithm in the manuscript and have corrected them. We'd like to clarify that this error only exists in the formula in the manuscript but do not exist in the codes, and we have once again verified that our DP algorithm code is indeed implemented as the reviewer noted (see lines 954-965 in gradient.c).   This issue occurred because there was a two-year gap between our implementation of the DP algorithm and the preparation of the manuscript.

9. The limitation that pseudoknots are not included is not adequately addressed in the text. This really needs to be in the Discussion because it is a major limitation with PlanarFold. Potential users should understand this.

Since this question is the same as Reviewer #1's Q2 and Q3, please refer to our responses in 2&3.

10. I was able to build the package and run an example simulation on Linux. I could follow the manual, but I recommend presenting the applications using more step-by-step tutorials. The text does this to some extent, but some details are missing. The Amber-style approach for input and output helps with understanding.
The figures in the manual are very low resolution, including the example file contexts. This made reading the file examples very difficult.
I also found what I think is a bug. I tried running unrestrained simulations. I set ntr=0 in the controls.in file, which should turn off restraints. However, PlanarFold still halts with the message "FilenotFoundError: restraint_ntr.in".

We thank the reviewer for testing our software and providing valuable feedback. We have further refined some details in the User Manual for benchmarking and results reproduction. We apologize for previously submitting a compressed PDF version, which resulted in lower image clarity. We have now uploaded a high-resolution PDF, and the realtime updated User Manual has been made available on GitHub.

Regarding the earlier issue with the restraint input file, it was due to the code mandating a restraint file. This limitation has been resolved. The current version of PlanarFold can now run successfully without specifying any constraint file (restraint_ntr.in) when no restraints are required (ntr=0), and similarly, it can run without a confinement file (restraint_dpr.in) when confinement is not needed (dpr=0).

11. As a comment, the end of page 7 and start of page 8 (Figure S7) looks at the correlation between F1-score and folding energy. It is not clear to me that this should be correlated. Higher energy alternative folds that are close in energy to the native structure do not need to be close in structure to the native structure.

The decision to analyze plots of F1-score versus folding energy was informed by similar RMSD versus scoring/energy plots commonly used in prior studies of RNA 3D modeling and predictions, as exemplified by BRiQ (Fig. 3 & Fig. 5, https://www.nature.com/articles/s41467-021-23100-4), simRNA (Fig. 4, https://academic.oup.com/nar/article/44/7/e63/2467805?login=true), and ARES (Fig. S4, https://www.science.org/doi/10.1126/science.abe5650). While we also predict folding, our model operates in 2D plane, sampling secondary structures on a plane rather than 3D conformations. Consequently, we employed the F1-score, which quantifies secondary-structure similarity, instead of RMSD, which measures tertiary-structural deviation. Furthermore, in the aforementioned RMSD-energy plots from previous works, seeming correlations exist for some RNAs (i.e., lower model scoring corresponds to lower RMSD), although they were not explicitly analyzed.

In our case, we observed that PlanarFold's energy and F1-score exhibit a noticeable correlation for several RNAs, which we therefore analyzed and compared. It is important to note that this correlation is merely an observed trend and was not a target during our parameter optimization.

We speculate that for some RNAs lacking low-energy alternative states, their energy landscape exhibits a funnel shape. In such cases, the conformational search gradually approaches the global minimum and the native state, resulting in lower energies for structures closer to the native conformation. Overall, however, this correlation is not strong, with a median (for 58 RNAs) Pearson coefficient of 0.591.

12. On line 534, the text states that the dataset includes "low nested pairing ratio (<10%)". I think this should state "low unnested pairing ratio (<10%)" because I think this was to remove structures with a large portion of pseudoknotted pairs, i.e. unnested pairs.

We thank the reviewer for pointing out this mistake and has corrected it.

13. I did not review the computer code in detail.
I did, however, install PlanarFold and verify that the code works.
The step-by-step installation instructions in the readme were adequate.
As I noted in my review, I think the manual could provide more step-by-step help and more detail. That being stated, the manual is already much better than I have seen for many other software projects. The current state of the manual is not a barrier to publication

We would like to express our sincere gratitude to the reviewer for the patient testing and evaluation of our code. Currently, we have updated the GitHub repository with more comprehensive benchmark tests and a more complete user manual.

Reviewer #1

1. *Overall, the authors have addressed my original technical concerns by performing additional calculations and revising the manuscript.*
   The addition of computational details is satisfactory, as the necessary information has been incorporated into the supplementary material. Furthermore, the authors' justification for fine-tuning with limited NMR data is acceptable. I suggest adding a brief description between lines 198–209 to further clarify the rationale for this fine-tuning.

We have modified the description between lines 198–209 as: "*We compared PlanarFold's prediction performance on thermodynamics with that of three conventional methods on 11 hairpin RNAs displaying µs-ms timescale base-pair reshuffling (Fig. S10a). Since the parameters optimized by GA only stipulate the relative energy level of conformers, we fine-tuned the absolute energy difference of PlanarFold by fitting a single global scaling factor (Equation 1, see Methods) to NMR-derived thermodynamic data. In PlanarFold, the population of different states can be counted from a long, equilibrated trajectory (Fig. 3b), and can be further adjusted to match the corresponding experimental populations by tuning this factor that uniformly rescales the strength of all six potential terms (Equation 1, see Methods). As only one parameter is fitted, a limited set of high-quality NMR measurements suffices for calibration—a strategy analogous to that used by RNAstructure, whose nearest-neighbor thermodynamic parameters were fitted using limited UV-melting experiments. Ultimately, we chose a value of 0.33 for this scaling factor based on the thermodynamics of T1 RNA (Table S6 and Fig. S10b, see Methods). Importantly, such rescaling preserves the relative energies among conformers, thereby maintaining PlanarFold's secondary structure prediction performance.*"

2. *Regarding pseudoknot prediction: among the 15 selected PK-containing RNAs, hairpin–hairpin kissing, one of the most frequent motifs, is missing. If the model is unable to handle this specific motif, the authors should provide an explicit explanation.*

We thank the reviewer for raising this concern. Indeed, most kissing loops, some of which also belong to pseudoknots, cannot be simulated by PlanarFold due to planar geometric constraints. In Fig.S19d, we present one kissing loop (PK1 of 1SJ3) that cannot be sampled in the plane. Furthermore, kissing loops are also prevalent in tRNA and purine riboswitch, and can only form by out-of-plane folding and approaching. We have explicitly included kissing loops in lines 132-135 in the revised manuscript as "*It is important to note that PlanarFold is specifically designed to investigate RNA secondary structure dynamics in the plane; thus, tertiary interactions (e.g., pseudoknots and kissing loops) are beyond the scope of this study.*". We also modified the caption of Fig. S19 as "*…d. For 1SJ3, neither its PK1 (kissing loop) nor PK2 can be simulated in a*

plane, restricted by the persistence length of RNA single-stranded chain (5.5 Å in PlanarFold). Of note, most kissing loops (e.g. those in tRNAs and purine riboswitches) cannot be simulated by PlanarFold…"

Reviewer #2

I co-reviewed this manuscript with one of the reviewers who provided the listed reports. This is part of the Nature Communications initiative to facilitate training in peer review and to provide appropriate recognition for Early Career Researchers who co-review manuscripts.

Reviewer #3

3. *On page 8 of the response to reviewers, the authors rebut my concerns about the benchmarks included in the main text. I still do not agree with the decision by the authors to provide the benchmarks in the main text that have homologs to the training data contaminating the test data. The supplement has the correct benchmarks separating homologs properly. The authors are correct that prior studies separated training and testing sets based on sequence identity alone. Recent papers demonstrate that the sequence identity approach is a mistake [RNA. 2026. 32:428; Bioinformatics. 2022.38:3892], and prior work has done the split of training and testing sets correctly by separating homologs [RNA. 2012. 18:193]. Certainly, many pairs of RNA homologs have less than 80% sequence identity.*

We thank the reviewer for reiterating this methodological concern. We fully appreciate the importance of rigorous benchmark design to avoid overestimation of model performance.

We would like to clarify that our choice to split based on sequence identity (rather than strict homology) was made, on the one hand, to follow the prevailing practice in the RNA secondary structure prediction, on the other hand, to facilitate direct comparison with existing tools and to maintain consistency with the established literature. Regarding the references cited by the reviewer, we agree that the question of optimal benchmark splitting is an active area of discussion. While we note that the current consensus in the RNA secondary structure community still predominantly relies on sequence-identity-based splits—as reflected in the high-impact methods benchmarked in this work—we have taken the reviewer's concern seriously and ensured that our manuscript presents both forms of evaluation (cross-sequence and cross-family, Fig.2). And we have explicitly indicated the risk of simple cross-sequence validation and rephrased the lines 170-177 as "*PlanarFold achieved a highly sequence-independent prediction performance (Fig. S7a), which is comparable to or even better than more complex DL-based methods and traditional methods. Cross-sequence validation is common practice in the field; nonetheless, the risk of RNA homolog leakage exists in such tests. To further evaluate PlanarFold's ability to predict unseen RNA families under a more stringent*

condition, we rearranged the datasets using the a homology-based split according to Rfam34 families and retrained the model (Fig. S7b). Overall, PlanarFold achieved performance comparable to RNAstructure and superior to mfold in cross-family prediction (Fig. 2b), demonstrating its generalization capability across RNA families" with reference [*RNA.2026.32:428*] cited within. We believe this dual presentation provides readers with full transparency and demonstrates that PlanarFold's performance holds under both evaluation frameworks.

4. *With respect to the computational details, I think it would be better to provide all the details in the Supplement rather than the software manual. It would be nice to have these details documented as part of a publication, and the authors might find the documentation of a publication helpful in the future. That being stated, I did find the manual helpful, and this is a matter of opinion.*

We thank the reviewer for this constructive suggestion about our material organization. We agree that documenting computational details as part of the publication enhances reproducibility and long-term accessibility. That said, considering the different language style of Supplementary Information and user manual, we have kept the user manual as a separate document, as it is designed for practical use by researchers who wish to run PlanarFold. The manual is formatted as a hands-on guide with command-line examples, troubleshooting tips, and usage workflows, which we believe would be less convenient if embedded within the Supplementary Information. To ensure both accessibility and citability, we have deposited the manual in a public repository (*https://github.com/Lanxiang-thu/PlanarFold/tree/main/benchmark*) and provided the link in the **Data Availability** section of the manuscript.

We believe this approach—core protocols in the Supplement for archival purposes, and a separate user manual for practical usability (as a living document)—strikes a balance that serves both the publication record and the broader user community. We appreciate the reviewer's recognition that the manual was helpful, and we hope this dual format satisfactorily addresses the reviewer's concern..

5. *I have two suggestions for the main text:*
   On page 6, the text stated that pseudoknots are "beyond the scope of this study". This text should be revised given the new pseudoknot work in Figure S19.

We sincerely appreciate the reviewer for pointing out this negligence during our revision of manuscript. Before the second round of revision, we assumed that most pseudoknots are not accessible to a 2D-MD engine like PlanarFold. During the second round of revision, the newly added PK simulation function demonstrated that some short-ranged pseudoknotted base pairs can be accurately sampled by PlanarFold, while other short-ranged pseudoknotted base pairs are still inaccessible given the restriction of plane geometry.

We have revised the related lines (132-135) as "*It is important to note that PlanarFold is specifically designed to investigate RNA secondary structure dynamics* in the plane*; thus, tertiary interactions (especially long-range pseudoknots and kissing loops and* kissing loops*) are beyond the scope of this study due to geometric constraints.*".

6. *On page 15 line 424, "benchmared" should be "benchmarked".*

We thank the reviewer for pointing out this typo in the revised text, which has been corrected. Other revised text has been carefully checked.

7. *For the Supplement:*
   Figure S19 has provides new (and interesting) benchmarks with pseudoknot predictions. This needs additional explanation as to how the changes were made to planarfold. For example, the dynamic programming algorithm step needed revision, but I don't see the revision detailed in the supplement. There are probably other details that also need to be explained.

We actually updated the related *Supplement methods* in the *Dynamic Programming* section in the last revision about the modifications related to the newly added functionality of simulating pseudoknot. The specific statement (black underlined phrases) about this newly added functionality is "*4. Exclude parallel stem, trans-isomer, and pseudoknotted base pairs. First, occasionally formed "parallel" stems are excluded, as all most naturally-occurring RNA helices are "anti-parallel". Second, "trans-isomer" is a phenomenon that occurs when two stems are in two opposite planar topologies, which is an issue only existing in planar structures (analogous to cis-trans isoform of carbon-carbon double bond) but not in 3D space. We devised a vector-based method to eliminate trans-isomer and pseudoknotted base pairs simultaneously. Third, since our model is designed to study secondary structure and its related dynamics, pseudoknotted base pairs are also prohibited. Of note, in the contact map, the pattern of pseudoknotted (un-nested) base pairs is no different from the pattern of nested base pairs. While in planar topology, many long-range pseudoknotted stems cannot be easily formed, restricted by the persistent length of RNA chain (5.5 Å in PlanarFold). However, for some short pseudoknot RNAs, their un-nested base pairs can form in our planar representation (Fig. S20). To permit the formation of pseudoknotted base pairs, the procedure of PK exclusion should be ignored by setting the control parameter iPK=1*".

This modification can be easily neglected as it is probably much smaller than the reviewer would expect for such an important new function (simulating pseudoknot). However, as we have specifically designed an algorithm to exclude the formation of pseudoknot in the very early version of PlanarFold's codes, during the last revision, we only needed very simple modifications to add a switch (iPK) to toggle pseudoknot exclusion. Specifically, this new

functionality (iPK=1) is readily achieved by: (1) adding two lines in the "*gradient.c*" source code file (see **Fig.R1**, lines 1006 and 1139) to provide an option for excluding the pseudoknot base pairs (iPK=0) or not (iPK=1); and (2) creating/defining the variable for iPK in other source code files. Beyond that, no other changes were made to the source code.

gradient.c — Edited

gradient

gradient 〉 eBasePair(top, sdt, E_basepair, tim, dpr, fp, nstep)

```c
587  void eBasePair(prmtop *top, state *sdt, double* E_basepair, timer *tim, int dpr, FILE *fp, int nstep){
1104         //--------- Step 4.2. Check the trans-isomer and Pseudoknots -----------
1105         //-----------------------------------------------------------------
1106         if (sdt->iPK == 0) {
1107             // 1. translation to superimpose 'bp5-5' onto the origin
1108             moveX = sdt->crd[3*aBPlist5[n]];
1109             moveY = sdt->crd[3*aBPlist5[n]+1];
1110             // translate the crd of "bp5-3"
1111             crdTx = sdt->crd[3*aBPlist3[n]] - moveX;
1112             crdTy = sdt->crd[3*aBPlist3[n]+1] - moveY;
1113             // get and translate the middle of "second basepair"
1114             // NOTICE: 'second-bp' is replace by the adjacent [i+1] and [j-1]
1115             //            for the harmony of stems and lonepairs (without 2nd bp)
1116             crdMx = (sdt->crd[3* (aBPlist5[n]+1)    ] + sdt->crd[3* (aBPlist3[n]-1)    ])/2 - moveX;
1117             crdMy = (sdt->crd[3* (aBPlist5[n]+1) +1] + sdt->crd[3* (aBPlist3[n]-1) +1])/2 - moveY;
1118             // 2. rotation 'bp5' to align with "x+" axis
1119             theta = acos(crdTx / sqrt(crdTx*crdTx + crdTy*crdTy));  //rotation angle
1120             if (crdTy>0){   //change to clockwise rotation!
1121                 theta = -theta;
1122             }
1123             // Rotation Matrix = [[cos(theta), -sin(theta)],[sin(theta), cos(theta)]]
1124             crdMrotY = crdMx*sin(theta) + crdMy*cos(theta);
1125             crdMrotY = (crdMrotY<-1.0) ? -1.0 : (crdMrotY>1.0) ? 1.0 : crdMrotY;
1126             // 3. determine the orientation of stem
1127             if (crdMrotY>0.0){ // upwards
1128                 orient[i] = 1;
1129                 upward += aStem[i];
1130             }
1131             else{       // downwards
1132                 orient[i] = -1;
1133                 downward += aStem[i];
1134             }
1135         }
1136         else{
1137             orient[i] = 1;
1138             upward += aStem[i];
1139         }
1140
1141         n += aStem[i];
1142     }
1143     //-----------------------------------------------------------------
```

Figure R1. Adding a switch (iPK) to control pseudoknot formation

The key source codes related to the newly added functionality that allows pseudoknot formation ("*gradient.c*"). A new control variable named as "iPK" (C *int* type) is created in "sdt" (C *struct* type) variable (see above figure, line 1106) to control the pseudoknot formation. If iPK = 0, the pseudoknotted base pairs are excluded by this algorithm. If iPK = 1, all the base pairs (regardless of pseudoknot) are kept and processed by ensuing codes.
